# A Cognitive-Based Board Game With Augmented Reality for Older Adults: Development and Usability Study

**DOI:** 10.2196/22007

**Published:** 2020-12-14

**Authors:** Yen-Fu Chen, Sylvia Janicki

**Affiliations:** 1 Department of Media Design Tatung University Taipei Taiwan

**Keywords:** cognitive-based, augmented reality, board game, older adults, cognitive health, serious game

## Abstract

**Background:**

Older adults in Taiwan are advised to adopt regular physical and social activities for the maintenance of their cognitive and physical health. Games offer a means of engaging older individuals in these activities. For this study, a collaborative cognitive-based board game, Nostalgic Seekers, was designed and developed with augmented reality technology to support cognitive engagement in older adults.

**Objective:**

A user study of the board game was conducted to understand how the game facilitates communication, problem solving, and emotional response in older players and whether augmented reality is a suitable technology in game design for these players.

**Methods:**

A total of 23 participants aged 50 to 59 years were recruited to play and evaluate the game. In each session, participants’ interactions were observed and recorded, then analyzed through Bales’ interaction process analysis. Following each session, participants were interviewed to provide feedback on their experience.

**Results:**

The quantitative analysis results showed that the participants engaged in task-oriented communication more frequently than social-emotional communication during the game. In particular, there was a high number of answers relative to questions. The analysis also showed a significant positive correlation between task-oriented acts and the game score. Qualitative analysis indicated that participants found the experience of playing the game enjoyable, nostalgic objects triggered positive emotional responses, and augmented reality technology was widely accepted by participants and provided effective engagement in the game.

**Conclusions:**

Nostalgic Seekers provided cognitive exercise and social engagement to players and demonstrated the positive potential of integrating augmented reality technology into cognitive-based games for older adults. Future game designs could explore strategies for regular and continuous engagement.

## Introduction

### Background

The growing older population has become a major source of concern in the past decade due to the demographic transition occurring in many developed countries [1]. One of the Sustainable Development Goals (SDGs) listed by the United Nations is to ensure healthy lives and promote well-being for all ages [2]. To support healthy aging and well-being, a variety of factors should be considered, for example, preventing injury or illness, maintaining physical and cognitive function, retaining intimate human relationships, and actively participating in social activities [3]. Studies have shown that the cognitive health of older populations is related to their performance in daily activities and that the presence of cognitive impairments strongly impacts older individuals’ level of independence [4-6]. Thus, it is important to assist older adults in maintaining their cognitive health in order to support their ability to perform daily activities and live independently. Taiwan is one of the fastest-aging societies in the world [1]. The Taiwanese government has undertaken measures to address issues relating to the growing older population. Some public policies that support older persons in Taiwan focus on the development of a society for active aging [7]. These policies suggest that Taiwanese older adults adopt regular physical and social activities for the maintenance of their cognition and health. However, active and continuous engagement in physical and social activities can be challenging for older adults.

Games may offer a means of engaging older persons in regular health-promoting social and physical activities because they can also provide a source of entertainment and engagement. Studies have shown that gaming can produce general cognitive and perceptual improvements, making games a promising approach to preventing or slowing down cognitive decline [8-11]. In recent years, different types of games have been used to investigate the effects of game play on the cognition of older populations [12-14]. These studies have demonstrated that games have the potential to serve as effective cognitive training tools that can benefit cognitive and perceptual abilities in older adults. With growing technological development, digital games could also be easily incorporated as a daily activity for older players. Recent reports from the United States and the United Kingdom have shown that the number of older players playing digital games is on the rise [15,16].

As older populations continue to grow, older players could potentially become a very large customer base in the gaming market. However, the older population has not typically been targeted as an audience in the design of digital games [17]. Currently, digital games are mainly designed for adolescents and young adults and do not consider the physical limitations or psychological preferences of older people. As a result, many digital games are unenjoyable or unsuitable for older adults because design considerations ignore the needs of older populations. For example, the size of the objects on the screen may be too small or the movements and reactions required of the user may be too rapid [18]. Nevertheless, there have been some studies that have investigated digital game preferences of older adults. For example, older adults dislike violence in digital games [15] and prefer intellectually stimulating games, such as puzzle games [19]. Games and game system designs that ignore the needs of older gamers may exclude a large number of potential players [20]. Therefore, it is important for game designers and developers to understand the capabilities, limitations, and interests of older gamers.

In addition to understanding older adults’ limitations and preferences in games, an equally important aspect of developing digital games for them is understanding their acceptance of new technology in games and their willingness to learn a new form of technology to play games. The technology of mobile devices (such as smartphones and tablets) is well established, commonly used, and affordable for the general public. Mobile devices seem to be readily adopted by older adults in their daily lives [21]. Digital technology, such as augmented reality (AR), has been widely applied to mobile devices, and its applications are on the rise [22]. AR integrates virtual information with information in the physical environment [23]. Like digital games, AR is usually associated with younger user groups, and older adults are often excluded in the development of AR technology. However, AR applications have the potential to improve older adults’ social relationships, overall well-being, and quality of life and to support their independence [23,24]. Studies show that even though interacting with digital technology may present unique challenges to older users [25], employing new technology in games can enhance older players’ immersion and flow [26]. As such, mobile devices with AR technology used for gaming should offer older adults a new and enhanced gaming experience. However, there have been limited studies examining the effects of AR technology in gaming for older players.

For this study, we designed, built, and evaluated a cognitive-based cooperative board game that integrates AR technology and targets Taiwanese older players. We examined how the game facilitates communication and evokes emotional responses and how players responded to the use of new technology in the game. We studied Taiwanese individuals aged 50 to 59 years, as they represent the next generation of older adults in Taiwan and because of their familiarity with mobile devices in daily life [21]. The content of the AR game was developed to reflect Taiwanese culture and history.

### Literature Review

#### Cognitive Health Games

Cognitive performance in working memory, executive function, semantic memory, and logical reasoning could be enhanced by playing games [27,28]. Serious games are games designed to achieve purposes beyond entertainment [29]. They are designed to be used as an educational tool to inform or as a training tool to encourage behavioral changes. Cognitive health games are a form of serious games that support cognitive function by integrating tasks that may help to prevent or slow down cognitive decline and providing players with information relevant to maintaining cognitive health [8,9]. In general, cognitive health games have three main purposes: (1) a preventative purpose intended for healthy players to prevent cognitive decline, (2) a therapeutic purpose intended for cognitively impaired players to maintain mental activity and slow down cognitive decline, and (3) an informational purpose intended to provide the player with formal or informal cognitive assessments and educate the player about different cognitive disorders [30]. Cognitive health games include cognitive training games, which aim to improve a player’s cognitive abilities through cognitive tasks, and cognitive screening games, which are used to assess the player’s performance in cognitive tasks [31].

Though digital games are becoming more prolific, studies seem to suggest that board games can attain emotional satisfaction more effectively. Using Norman’s 3 emotional design levels—the visceral level, behavioral level, and reflective level—as a measure [32], a study comparing video games to board games found that the satisfaction levels declined on all 3 levels for study participants when playing digital games [33]. Another study found that older populations specifically are less drawn to video games than young adults and are more inclined to play board games [26]. The physical appearance and tangibility of board games may make them more legible and memorable to players and reduce errors in operation. Players can experience intimacy, vivid imagery, sympathetic responses, and social satisfaction while playing board games with others in person [33]. Because of the face-to-face interaction during play, board games give players more social connection and social exchange than virtual or digital games. This may explain why playing board games not only decreases cognitive decline in older adults but reduces the rate of depression as well [34]. Because of these advantages, board games appear to be an appropriate means to support cognitive health in older populations. As such, our study focuses on the development of a cognitive-based board game for older adults for cognitive training.

#### Nostalgia as the Cognitive Element of Older Adults’ Gameplay

Cognitive responses are commonly shown to be affected by feelings of nostalgia [35,36]. Nostalgia refers to a general liking or favorable affect toward objects or events of the past [36]. It differs from memory in that memory refers to the ability to remember information, experiences, and people, whereas nostalgia consists of remembering and reflecting [37], which includes emotional reactions that can be positive (ie, warmth, tenderness, joy, elation) or negative (ie, loss, fear, sadness) [38].

Although nostalgia can be triggered by negative emotions such as loneliness and depression [37], it typically serves as a repository of positive affect [39] and has also been characterized as a joyous experience that provide “a feeling of elation” [40]. Nostalgia may provide a positive view of the past that can help increase players’ feelings of social connectedness and enhance meaning in their lives [41]. For populations especially vulnerable to social isolation, such as first-year boarding students, immigrants, and older adults, experiencing feelings of nostalgia may help overcome feelings of loneliness [42]. In general, there are two different types of nostalgic reactions: personal and historical nostalgia [43]. Personal nostalgia refers to reactions generated from a memory of one’s past (“the way I was”), whereas historical nostalgic reactions are related to a general period of time in history that could include a time before one was born (also known as historical or communal nostalgia, or “the way it was”) [43,44]. The occurrence of nostalgia appears to vary with age: nostalgia levels tend to be higher among young adults, then drop in middle age and rise again during old age [45]. In general, nostalgia is set off by two types of triggers. One type occurs internally and usually arises from feelings of psychological distress, such as loneliness [39], meaninglessness [46], and boredom [47]. When people have negative feelings, they may turn their thoughts toward the past to deal with their current discomfort. Another type of trigger occurs externally through sensory stimuli in one’s environment that reminds an individual of their past, such as smells [48], tastes [49], the sight of different objects (often childhood related) [50], and music [51].

For this study, we incorporated imagery of nostalgic objects into the visual materials of the game to evoke memories of players’ childhoods that might lead to positive emotions and stimulate cognitive response in participants. Nostalgic objects were selected to be external visual triggers to elicit both a historic nostalgic reaction, in which participants are reminded of a time period in the past that they lived through, and a personal nostalgic reaction, in which the objects might also remind participants of their own stories from childhood. The details of the selection of nostalgic objects for the game is discussed in a later section.

#### AR Technology for Older Adults

Although AR has existed for about three decades, AR systems have only become affordable and available to the general public because of the recent proliferation of mobile devices [52]. AR is defined as a system or visualization technique that fulfils three main criteria: (1) a combination of real and virtual worlds, (2) real-time interaction, and (3) accurate three-dimensional (3D) registration of virtual and real objects [53]. AR differs from virtual reality (VR) in that VR allows the user to be completely immersed inside an artificial environment, whereas AR is a real-time technology, whereby a physical environment is augmented by adding or embedding virtual information [54]. It allows for a form of information presentation that can more efficiently convey abstract concepts and in turn help improve the spatial and cognitive abilities of its users [55].

Most applications with AR are available using mobile devices that have built-in cameras, GPS sensors, and internet access to embed real-world environments with dynamic, context-aware, and interactive digital content. Currently, 2 forms of AR are available to app developers: vision-based and location-aware AR [56]. Vision-based AR shows the digital content on the screen of mobile devices when users point the built-in camera at an object, such as a two-dimensional (2D) graphic or quick response code. Location-aware AR shows the digital content on the screen when users move through a physical area with the GPS-enabled mobile device. Many studies in the areas of gerontology and human-computer interaction have focused on the use of AR technology to support aging-related needs. For example, Korn et al [57] developed a system combining AR and gamification to support older adults’ rehabilitation activities. Kim and Dey [58] developed an AR navigation display system on vehicle windshields to assist older drivers, showing a significant reduction in the number of navigational errors made by users. These studies suggest that AR technology can be a supportive tool to respond to age-related changes in cognitive and physical abilities when designing for older populations. In recent years, AR technology has been applied to the design of various board games to enhance players’ gaming experiences [59,60]. Boletsis and McCallum [61] developed the cognitive augmented reality cubes system, which is a collection of cognitive-based mini-games combining AR and physical objects that is designed to prevent cognitive decline in older players through frequent play. Their research has shown that integrating AR technology into games can enhance engagement for older adults [61].

In this study, we integrated vision-based AR technology into the design of a cognitive-based collaborative board game to explore how older adults react to the use of AR technology in games and examine whether AR technology can enhance their gaming experience.

### The Design of a Cognitive-Based Board Game With AR

In response to the rapidly growing older population in Taiwan and the rising need to promote active aging and cognitive health in older populations, we designed Nostalgic Seekers, a cognitive-based collaborative board game that integrates AR technology and targets Taiwanese players aged 50 and older. The game consists of physical puzzle pieces designed to be assembled on a table and vision-based AR technology that responds to combinations of puzzle pieces that form specific 2D graphics. An AR app was developed for the game so players could point the built-in camera of their mobile device at a 2D graphic of the board game to conjure a digital 3D model. These 3D AR models take the form of various objects from a period that the targeted players might associate with their childhood. The game is designed to be a cognitive training tool for older adults. Following its design and development, we conducted a user test to examine the cognitive effects of the game, presented in the “Methods” section. With the development of this game, we wanted to examine if a board game integrating AR technology was an appropriate tool to engage older Taiwanese adults and how our game supports the cognitive health of this population.

#### The Selection of Nostalgic Objects for the Board Game

Nostalgic Seekers was designed for Taiwanese individuals aged 50 to 59, considered the next generation of Taiwanese older adults. To elicit feelings of nostalgia in these players, the AR-projected artifacts in the game resemble various products from the 1960s, 1970s, and 1980s, covering the period that represents the childhood of our target players. These 3 decades also represent an era that brought about rapid industrialization and economic growth in Taiwan. During this time, many national development projects were proposed and undertaken. With prolific government planning efforts and the rise of universal education, Taiwan made huge advances in industry and agriculture and raised living standards substantially [62]. Taiwanese people began to experience the convenience of industrialization as families started to buy and use diverse industrialized products, including home electronic products and private vehicles. This era marked a rapid change in the lifestyles of Taiwanese people, representing a critical period in Taiwanese modern history that is referred to as the ‘Taiwan Economic Miracle” [62]. As a result, many products from the 1960s, 1970s, and 1980s remain memorable and iconic of a unique time in history to Taiwanese individuals older than 50 years. We selected a range of products from this era to represent the nostalgic objects in Nostalgic Seekers. Two approaches were used to determine which products were most suitable to incorporate into the game: (1) a Likert-scale questionnaire showing images of a wide range of products from the 1960s, 1970s, and 1980s, as seen in Multimedia Appendices 1 and 2, and (2) a brief individual interview following the questionnaire.

Through snowball sampling, a total of 30 participants aged 50 to 59 (10 men and 20 women) were recruited to take part in the initial questionnaire and interviews for the selection of nostalgic objects. The questionnaire was conducted electronically, and images were shown to participants on a screen. In the questionnaire, 93 images of products from the 1960s, 1970s, and 1980s were presented, including home electronic products, vehicles, toys, furniture, accessories, and daily use products. Participants were asked to observe the image, then rate their familiarity with the object on a Likert scale from 1 to 7 (1=strongly disagree; 2=disagree; 3=slightly disagree; 4=neutral; 5=slightly agree; 6=agree; 7=strongly agree). Each participant was given 15 minutes to fill out the questionnaire. After completing the questionnaire, a brief interview was conducted with each participant in which participants provided their thoughts on the objects. Each individual interview lasted 25 to 30 minutes. Interviews were recorded with a voice recorder with participants’ consent.

Average scores for each object were calculated following the questionnaire. Scores ranged from 4.50 to 6.63, with a mean score of 5.83. Moreover, the interviews revealed the different emotional reactions participants had to the various objects. Images of toys reminded participants of sentimental memories from childhood, bringing some participants back to the good times they shared with their childhood friends. For example, one male participant said:

Seeing these toys gives me the feeling of joy. They remind me of the fun times I had with my old friends. I was so happy.

Another participant said:

When I see these toys, I feel like I am back in my childhood and playing with other children.

Images of toys reminded participants of carefree memories of their childhood playing with others and brought a positive feeling of nostalgia. In addition, images of daily use products, such as toiletries, kitchenware, and cleaning products, though seemingly mundane, reminded participants of the details in their everyday lives, bringing them back to a time in their past. As one participant stated:

I still remember the smell of Ming Sing Floral Water because my family always used it in the morning. It reminds me of the wonderful time I had with my mom and sisters.

Another participant said:

These daily use products were seen in every family 30 years ago. They were cheap but useful.

These daily necessities represent different facets of people’s lives during the 1960s, 1970s, and 1980s, eliciting nostalgic feelings in participants. Other objects, such as home electronic products, vehicles, furniture, and accessories, also evoked memories in participants. However, they had to be especially iconic and representative of the time for participants to have specific associations with them and consider them nostalgic objects.

**Figure 1:**
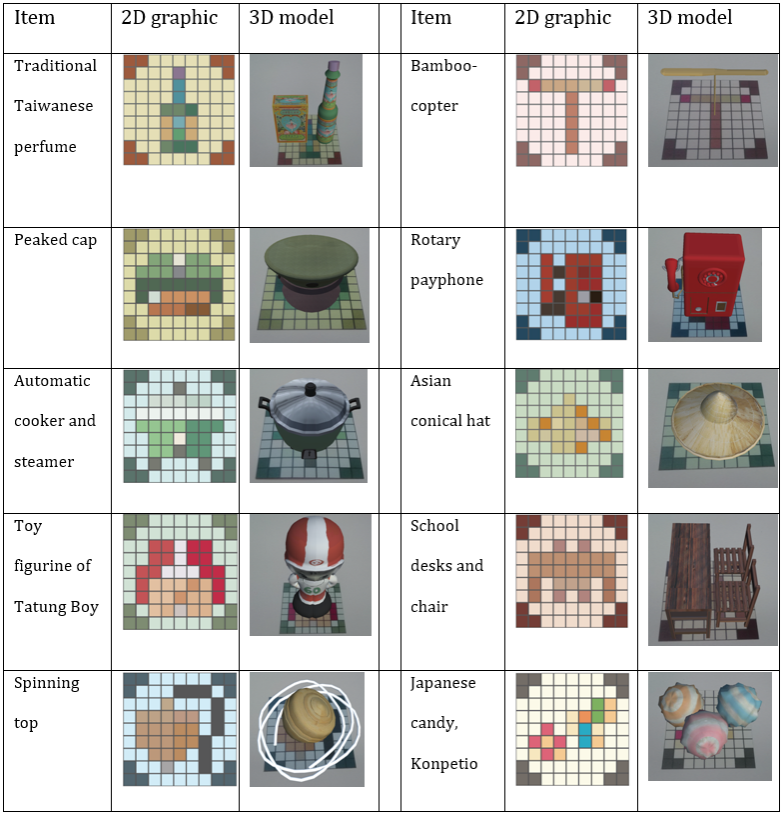
The graphics in pixel art and the 3D models of the 10 objects in Nostalgic Seekers. 2D: two-dimensional; 3D: three-dimensional.

Based on this investigation, the following 10 objects were selected for the board game: a traditional Taiwanese perfume, Ming Sing Floral Water (average score of 6.63); a peaked cap (average score of 6.56), an automatic cooker and steamer (average score of 6.56), a toy figurine of Tatung Boy (average score of 6.50); a spinning top (average score of 6.46); a bamboo-copter (average score of 6.43); a rotary phone (average score of 6.40); an Asian conical hat (average score of 6.40); school desks and chairs (average score of 6.40); and a Japanese candy, Konpetio (average score of 6.40). The 10 selected objects were modeled in Autodesk Maya (Autodesk Inc), a 3D modeling software, and incorporated into the game as the AR images. Using the shape, form, and color of each nostalgic object as guidelines, a 2D pixel art graphic was created to represent each of the 10 objects in an abstracted form. Collectively, these 2D graphics became the puzzle pieces in the game. Each completed 2D graphic represents a 2D target for the vision-based AR in the game. Figure 1 shows the graphics of the 2D pixel art and the 3D models of the 10 objects.

#### The Rules of the Board Game

**Figure 2:**
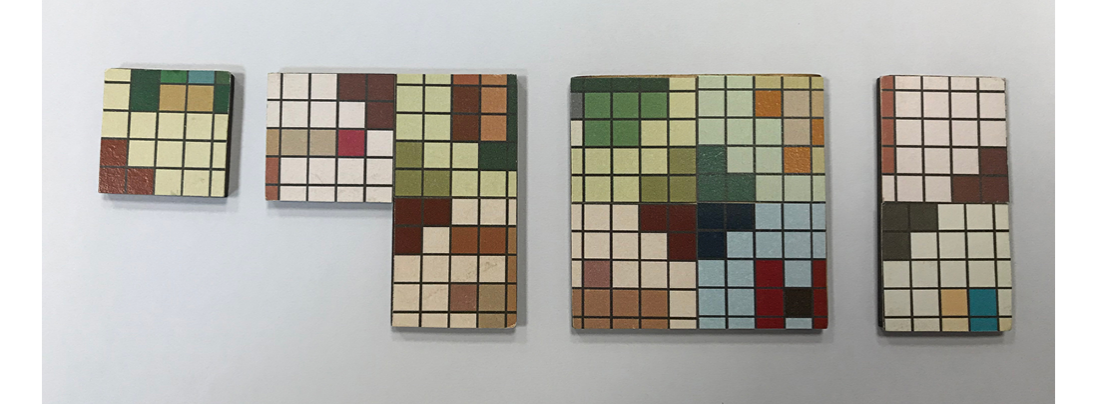
The 4 different types of puzzle tiles and the pixel graphics on the tiles.

**Figure 3:**
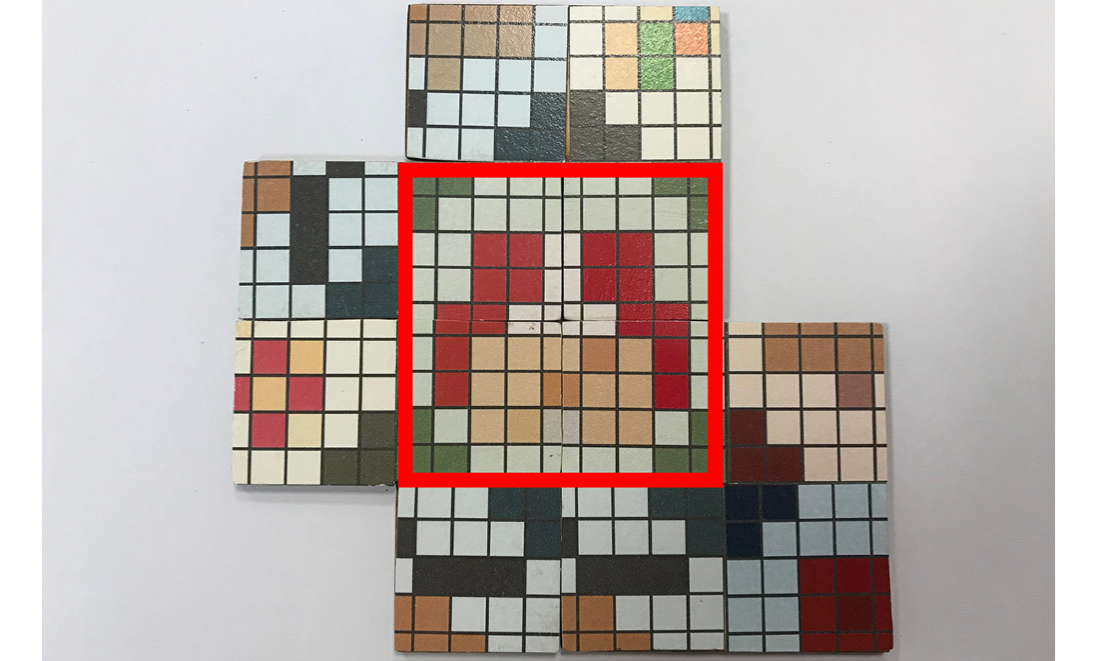
The red square area shows a complete pattern that was constructed using different types of puzzle tiles.

Nostalgic Seekers was designed to be a collaborative puzzle game for 4 to 5 players. The components of the game include 5 player tokens, 1 base tile, and 60 puzzle tiles. As shown in Figure 2, these 60 puzzle tiles come in 4 different shapes: the small square tile, the rectangle tile, the *L*-shaped tile, and the large square tile. Each puzzle tile includes part of the 2D pixel graphic for one or more of the nostalgic objects, so the players have to put the puzzle tiles together to construct the complete 2D pixel image of a nostalgic object (Figure 3).

Each player has one player token representing a treasure seeker looking for nostalgic artifacts in the game. The player tokens are placed on top of the base tile, which sits at the center of the play area. The puzzle tiles are placed in piles at the edge of the play area away from the base tile. Puzzle tiles of the same shape should be put in respective piles. For each turn, a player can perform 2 of the following 4 actions: (1) move the player token by 1 position, (2) pick 1 puzzle tile from one of the 4 piles and put the puzzle tile next to the tile that has the player token, (3) move a puzzle tile in the play area to complete the pattern, and (4) inspect a completed pattern of the puzzle tiles via a mobile device, such as a mobile phone or tablet, using the associated app.

**Figure 4:**
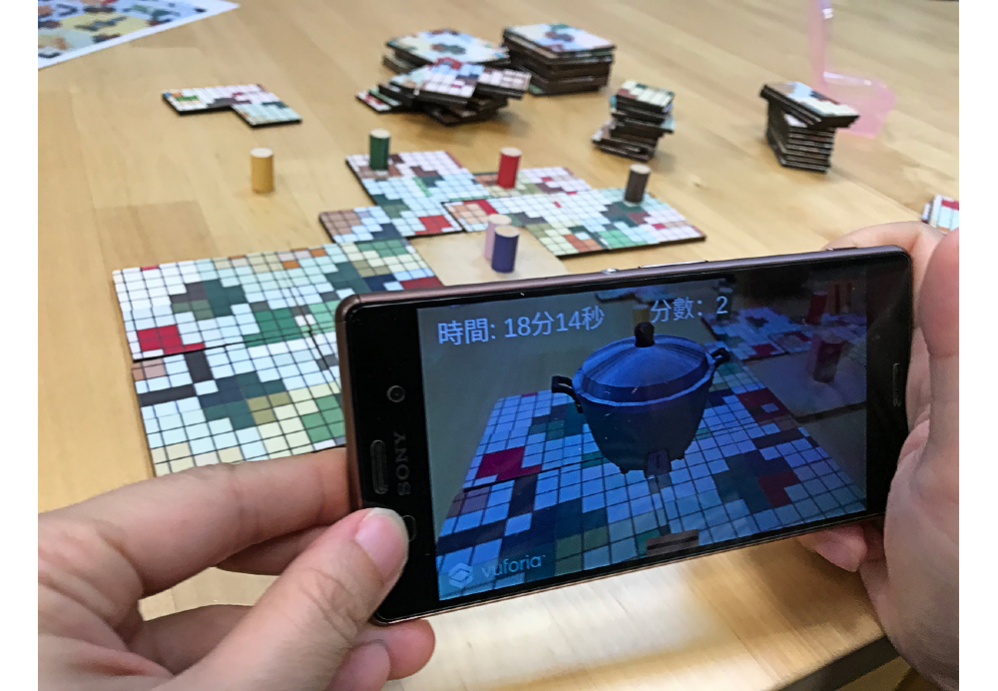
The three-dimensional object of one of the nostalgic artifacts, an automatic cooker and steamer, is presented on the screen of a mobile device when the pattern of the puzzle tiles is assembled correctly.

After a player has taken 2 actions in their turn, the next player goes. During the game, players have to put the tiles together, then inspect the combined tiles via the AR technology app with a mobile device, such as a mobile phone or tablet. If the tiles are assembled correctly and form the accurate 2D pixel art, a 3D object will appear on the screen along with the game score (Figure 4). Players can discuss strategies and moves with each other during the game, but only 1 player can take an action at one time. Players have 20 minutes to complete the game.

## Methods

**Figure 5:**
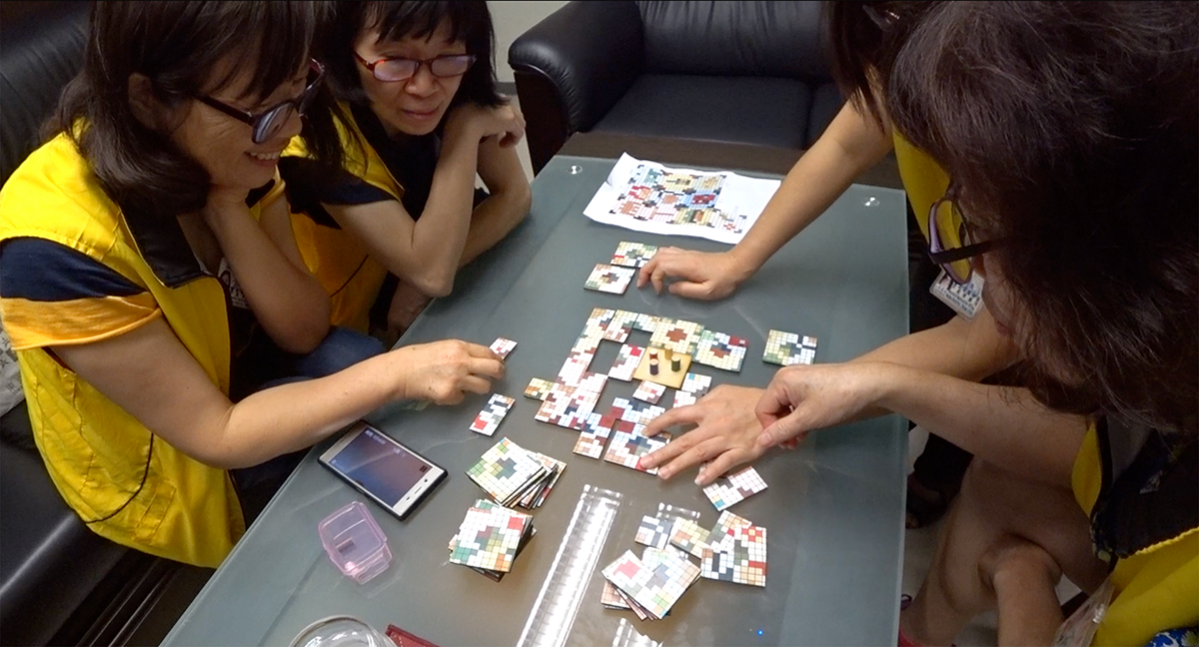
Four participants played the game in one of the game-testing workshops.

To evaluate Nostalgic Seekers, 23 of the original participants who gave feedback on the nostalgic objects volunteered to participate. A total of 13 women and 10 men aged 50 to 59 years participated in the evaluation workshops. In consideration of participants’ variable availability, 7 game-testing workshops were arranged on different dates, and each workshop had 4 participants (Figure 5). To keep the number of participants in each workshop the same, 5 participants were invited to participate in 2 different workshops. However, they did not play with any other participant twice. Each workshop lasted 1 hour, including 30 minutes for playing the game. This study was approved by the research ethics committee. We obtained written permission to use images of individuals included in this paper.

At the beginning of the game-testing workshops, the research team explained the rules of Nostalgic Seekers in detail to the participants. Two digital cameras and one sound recorder were set up in each workshop to record the participants’ interactions during the game. After the game ended, each participant gave their feedback on the game through structured interviews, which were recorded with a voice recorder.

Observing players’ interactions with each other in the game play is a useful approach to evaluating the game design because players engage in both verbal and nonverbal communication. Bales’ interaction process analysis (IPA) is a method for the analysis of communication processes among small groups [63]. IPA consists of 12 complementary paired process categories for communication acts [63,64]. These 12 process categories are further subdivided into 4 major functions: social-emotional acts for positive reaction, social-emotional acts for negative reaction, task-oriented acts for questions, and task-oriented acts for attempted answers. Table 1 shows the system of process categories in the IPA.

**Table 1:** System of process categories in the interaction process analysis.

Function and process categories		Paired processes and central problems addressed
**Social-emotional: positive reaction**		
	1. Shows solidarity, raises other's status, gives help, rewards	1 & 12; integration
	2. Shows tension release, jokes, laughs, shows satisfaction	2 & 11; tension management
	3. Agrees, shows passive acceptance, understands, concurs, complies	3 & 10; decision
**Task area: attempted answers**		
	4. Gives suggestion or direction, implying autonomy for other	4 & 9; control
	5. Gives opinion, evaluation, or analysis, expresses feeling or wish	5 & 8; evaluation
	6. Gives orientation or information, repeats, clarifies, confirms	6 & 7; orientation
**Task area: questions**		
	7. Asks for orientation, information repetition, or confirmation	7 & 6; orientation
	8. Asks for opinion, evaluation, analysis, or expression of feeling	8 & 5; evaluation
	9. Asks for suggestion, direction, or possible action	9 & 4; control
**Social-emotional: negative reaction**		
	10. Disagrees, shows passive rejection or formality, withholds help	10 & 3; decision
	11. Shows tension, asks for help, withdraws out of field	11 & 2; tension management
	12. Shows antagonism, deflates other's status or defends or asserts self	12 & 1; integration

A total of 6 of the 12 process categories are related to social-emotional acts, with 3 positive and 3 negative types of expressions representing sociability and affect. Positive social-emotional content expresses solidarity or friendliness (category 1), tension relief or dramatizing (category 2), or agreement and understanding (category 3). Negative social-emotional content shows disagreement and passive rejection (category 10), tension (category 11), and antagonism (category 12). Categories 1 and 12 are related to issues of integration. Categories 2 and 11 are related to issues of tension management. Categories 3 and 10 are related to issues of decision making.

The other 6 process categories are task-oriented acts, including the exchange of questions and responses to complete a task. The 3 types of questions in task-oriented acts are asking for task information or orientation (category 7), asking for an opinion (category 8), and asking for a suggestion (category 9). The 3 types of answers in task-oriented acts are giving a suggestion or command (category 4), giving an opinion (category 5), and giving task information or orientation (category 6). Categories 4 and 9 relate to problems of control. Categories 5 and 8 relate to problems of evaluation. Categories 6 and 7 relate to problems of orientation. The IPA uses a single act as the unit for coding and analysis. An act is a single sentence or its equivalent, such as nonverbal communication or an exchange that may be understood by other members.

Sometimes participants’ verbal communication was also accompanied by nonverbal behavior. To avoid coding the same acts twice, when both verbal and nonverbal communication occurred, the coding only counted verbal communication. Two coders were trained in accordance with the IPA coding scheme. Cohen κ was used to assess the interrater reliability for the agreement between the 2 coders. The Cohen κ values were greater than 7 for all 7 workshops and thus satisfactory in all instances. Specifically, the kappa index (κ) for each workshop, in order of the workshop, was 0.76, 0.86, 0.81, 0.88, 0.83, 0.79, and 0.89.

Structured interviews were conducted with each participant following each game and recorded with a voice recorder. Participants were asked to answer questions related to their experience of the puzzle game, specifically how they felt about the design and goals of the board game, the process of collaborative problem solving, the use of AR technology, and their reactions to the nostalgic objects.

## Results

### Participants’ Communication Acts

After the coding was complete, a total of 2021 communication acts were recorded from these 7 workshops; 278 total communication acts were observed in the first workshop, 144 in the second workshop, 213 in the third workshop, 132 in the fourth workshop, 349 in the fifth workshop, 465 in the sixth workshop, and 431 in the seventh workshop. Table 2 shows the observed frequencies of participants’ communication acts in these 7 game-testing workshops.

**Table 2:** The observed frequencies of participants’ communication acts in the game-testing workshops.

Game test workshop	Social-emotional acts		Task-oriented acts		Game score
	Positive	Negative	Attempted answers	Questions	
1st	20	27	165	66	2
2nd	12	15	78	39	0
3rd	10	2	139	62	2
4th	27	4	75	26	0
5th	42	4	208	95	2
6th	33	9	293	130	3
7th	60	10	254	107	3

Participants’ social-emotional acts and task-oriented acts were analyzed with a chi-square goodness-of-fit test to assess the interpersonal interaction during the game test. The value (χ^2^_3_=7.81; α=.05) on the chi-square distribution table indicated that when a chi-square value was greater than 7.81, there was a statistically significant difference in the data. The chi-square tests testing the observed frequencies of participants' social-emotional acts and task-oriented acts achieved statistical significance in all workshops (first workshop: χ^2^_3_=162.65 > 7.81; second workshop: χ^2^_3_=77.5 > 7.81; third workshop: χ^2^_3_=223.98 > 7.81; fourth workshop: χ^2^_3_=81.52 > 7.81; fifth workshop: χ^2^_3_=270.7 > 7.81; sixth workshop: χ^2^_3_=428.93 > 7.81; seventh workshop: χ^2^_3_=286.6 > 7.81).

These results indicate that participants performed task-oriented acts much more frequently than social-emotional acts when playing the game. For the frequency of participants’ task-oriented acts, the frequency of attempted answers was more than 2 times greater than the frequency of questions in each workshop.

The analysis results shown in Table 3 indicate that the frequency of task-oriented acts, namely the communication acts of questions (*R*^2^=0.834; *F*_1_=25.184) and attempted answers (*R*^2^=0.870; *F*_1_=33.535), significantly predicted the game score, with greater frequencies of each correlating with higher scores. However, social-emotional acts of positive reactions (*R*^2^=0.303; *F*_1_=2.172) and negative reactions (*R*^2^=0.000; *F*_1_=0.002) did not correlate to the game score. Communication acts of questions accounted for 83% and attempted answers accounted for 87% of the total variance in the game score, showing that there was a significant relationship between the game score and participants’ task-oriented acts. This suggests that the game encourages participant discussion and cooperation for problem solving, providing participants a chance to practice their cognitive skills. In particular, the high number of answers relative to questions signals that participants were collaborating to find solutions together. Specifically, in the game-testing workshops, we saw participants not only communicating to solve the puzzle together but also collaborating to figure out the use of the AR technology.

**Table 3:** Simple linear regression analysis results of participants’ communication acts related to the game scores.

Dependent variable and independent variables		*R*	*R* ^2^	β	*t* test (*df*)	*F* test (*df*)	*P* value
**Game score^a^**							
	Positive reactions	0.550	0.303	0.039	1.474 (1)	2.172 (1)	.20
	Negative reactions	0.020	0.000	0.003	0.044 (1)	0.002 (1)	.97
	Questions	0.913	0.834	0.031	5.018 (1)	25.184 (1)	.004
	Attempted answers	0.932	0.870	0.012	5.491 (1)	33.535 (1)	.002

^a^Dependent variable.

On the other hand, for participants’ social-emotional acts, the relative frequencies of negative acts and positive acts varied. In the first and second game-testing workshops, the number of negative acts exceeded positive ones. However, in the remaining workshops, the frequency of positive acts was substantially higher than the frequency of negative acts. In total, across all workshops, the number of positive acts accounted for almost triple the number negative acts. The low number of social-emotional acts, particularly negative ones, signals that the collaborative problem-solving process in the game, including learning a new technology, was largely a rational process and did not trigger many negative emotions or frustrations in participants.

### Participants’ Feedback on the Game

Following the game in each workshop, participants were asked to provide feedback on the process and experience of playing Nostalgic Seekers through structured interviews. The participant interviews were recorded with a voice recorder and transcribed, then analyzed with deductive analysis to examine the participants’ reactions to the puzzle game and collaborative process, their acceptance of AR technology, and their reactions to the theme of nostalgia.

Overall, participants expressed positive experiences with the game. They enjoyed playing the game (16/23, 70%), found the game design intuitive (20/23, 87%), and felt positively about the collaborative process (16/23, 70%). Over half of the participants were especially intrigued by the use of AR technology (12/23, 52%). One female participant said:

The goal of this game is easy to understand. It’s a collaborative puzzle game for us, and we need to discuss. I think the experience of gameplay is great because it encourages us to talk. It also reminds me that we are a team… I was surprised by the use of AR technology in the game. It is stunning because AR is the main part of this game. We may be aware of something on the tiles, but we still have no idea what it is without AR.

This shows that the discussions and communication that occurred in the game through the collaborative game-playing process can have a positive social impact and that the AR component added an element of excitement and engagement that was attractive to participants. Another male participant stated:

For me, AR technology is a new technology that I am interested in… Even though this game is a board game, it cannot be played without AR. This game gave me a good experience with AR and makes me want to try other applications on mobile devices with AR technology!

Participants’ feedback revealed that, overall, they not only were willing to learn AR and accepting of the technology but also seemed excited and fascinated by the AR component in the game.

Additionally, participants also described some of the challenges in playing Nostalgic Seekers that were overcome through the collaborative process. In the game, participants had to put puzzle tiles together to construct different graphics of nostalgic objects in pixel art form before they could inspect the graphics via the AR technology with their mobile devices. Although participants were given reference graphics for the different images they were asked to construct, identifying the graphics on the puzzle tiles and assembling them was challenging for some participants. One female participant shared her experience with the pixel art graphics:

These graphics were not easy to identify, even though I knew these graphics were the abstracted form of some artifacts that I should be familiar with. We spent a lot of time putting the right tiles together… It was a big challenge in the game for us.

The level of difficulty in assembling the puzzle required participants to discuss, collaborate, and use trial and error to problem solve, which was the intention of the game. The same participant continued to say:

But when we inspected the graphics via mobile phone and got a 3D object on the screen and scored one point, I can’t describe the feeling…a sense of achievement! I am so proud of my team.

This participant reveals that even though constructing the puzzle was challenging and time-consuming, the difficulty made the accomplishment more rewarding. Another female participant described a similar experience:

It seems the graphics in pixel art should not be difficult to recognize, but it was difficult to put these tiles together correctly. The color, the shape… both were challenging for us. However, I got the sense of achievement when we got the 3D object on the screen… it was worth the effort.

Putting the pixel graphics together correctly with the puzzle tiles is central to the game mechanics, and it is the main cognitive task of the game that serves as cognitive exercise. The challenge in the game not only provides cognitive training but also gives participants a feeling of reward and sense of achievement when they eventually solve the problem and score points. In addition, some participants suggested that more specific directions should be given to assist players in playing the game.

Regarding the use of the 3D nostalgic objects with AR technology, participants agreed that these 3D nostalgic objects were a novel and appropriate reflection of the theme of the game. One male participant said:

I am familiar with these 3D objects on the screen, and they were a part of my life…. no wonder the name of this game is Nostalgic Seekers!

Another female participant gave her feedback:

Using AR technology to present these objects is interesting. Yes, we are the seekers and trying to find these nostalgic objects. I like the idea of the game, but a fly in the ointment was that we didn’t have the chance to talk about the objects.

This participant suggested that a facilitated or intentional discussion of the 3D objects could be a positive component to add in the game.

## Discussion

### Summary of Results

Through the design, testing, and both the quantitative and qualitative analyses of the game Nostalgic Seekers, this study examined how 50- to 59-year-old Taiwanese adults interacted and communicated with each other while playing a collaborative board game with AR technology. Our game was designed to be a cognitive training game that provides cognitive tasks [31], which are achieved through solving a puzzle and using a digital app with AR technology. Overall, we saw that participants’ communication during the game heavily focused on task-oriented exchanges rather than social-emotional ones, and the number of task-oriented acts positively correlated with higher scores. This finding indicates that the collaborative process of the game was able to facilitate and reward problem-solving communication; thus, it successfully engages players in cognitive exercise. Specifically, we observed participants not only problem solving for the puzzle itself but also troubleshooting the use of AR technology with each other. We also saw that for social-emotional communication, the overall number of positive acts significantly outnumbered negative ones. However, the number of negative acts exceeded positive ones in workshops 1 and 2. This may in part be due to the “alpha player problem,” in which a dominant player takes control of collective decision making and creates a negative atmosphere in the game [65]. In addition, we saw that nostalgic objects brought back specific memories and positive emotions in participants, which they were eager to share and discuss with each other, and helped strengthen the interest and engagement of the players during game play. Finally, we saw that the inclusion of AR objects in the game was engaging and exciting for the players rather than intimidating.

### Implications

The overall low number of negative social-emotional acts coupled with qualitative findings that showed that players enjoyed the collaborative problem-solving process and were excited by the AR app in the game indicate that the users embraced the use of AR technology in the game and that the learning of it became a part of the collaborative process. As such, we found that introducing a new technology in games, in particular in a collaborative game, can encourage supportive peer learning of the new technology in a fun way and is a cognitively engaging task in itself. Subsequently, there is strong potential in integrating applications of new technologies in board games as a tool to support cognitive health and active aging. There are also implications of using interactive and entertaining processes, such as collaborative games, as a means to teach new technology to older adults and help them become more comfortable with the use of digital technologies.

Consequently, our game shows potential for being used as a tool to support the Taiwanese government's active aging agenda [7], as well as the United Nation's SDGs for healthy living and well-being for all ages [2]. Consistent with other findings we previously referred to in the “Literature Review” section, our study also demonstrated that new technology can in fact enhance “immersion and flow” for older adults during game play [26], despite also presenting some challenges [25]. In fact, we also found that challenges that arose in navigating the technology became a part of the collaborative problem-solving process in the cognitive-based game. Our findings reiterate the potential and importance of digital games marketed to older adults [15,16], which are currently lacking [17], and could serve as a step to filling this gap.

### Limitations and Future Work

Some factors related to our study sample, such as participants’ experiences playing board games or their familiarity with AR and other digital applications, could have played a role in the gameplay and affected player interactions. However, these characteristics were not distinguished in our study sample. Future studies could explore if the game has different impacts on older adults with variable familiarity with technology or variable experiences with board games, for example.

In addition, a major concern with the active aging agenda is related to continuous engagement. Some critical considerations we have yet to address include how to motivate older individuals to engage in this form of game play regularly to achieve the desired cognitive benefits and how to design games that can be engaging after repeated use.

Some additional questions arose during the study that could be further examined in future studies. For example, how might communication style, interaction, and association to different nostalgic objects in the game differ between male and female players or players from different geographic regions in Taiwan? Moreover, how might acts of communication and interaction vary in a different cultural setting (such as in a Western culture)? Additional studies could also explore how task-oriented acts and social-emotional acts in games affect cognitive function in older adults differently and how the two types of acts may complement each other in supporting cognitive health in older adults. These questions have strong implications on modifications to the game design to maximize support for cognitive health in older populations. Future studies could also explore how integrating different virtual components, such as AR, into a physical board game might support cognitive health in unique ways.

Feedback from participants provided valuable suggestions on ways to improve Nostalgic Seekers for greater clarity and enjoyment. For example, some participants expressed that the 2D graphics were too difficult to recognize. Incorporating multiple levels of difficulty that can accommodate players with different cognitive abilities would provide greater accessibility and cognitive benefits to all players. Participants also expressed their interest in having more discussions about the nostalgic objects that they found. Incorporating a component of storytelling or facilitated conversation about the nostalgic objects that players find can encourage participants to engage in active remembering and enhance social and emotional interaction and communication, which are currently less prominent in the game. Finally, in its current design, Nostalgic Seekers does not necessarily appeal to repeated use, as the novelty of discovering the nostalgic objects would wear off once players are aware of the objects. Future iterations could consider ways to modify the game so that it remains exciting and entertaining for continuous use.

### Conclusions

Games offer a means of engaging older adults in social and physical activities for the maintenance of their cognition and health. As the number of older adults increases, active aging is becoming a growing priority for community well-being. Older players are also becoming a growing customer base in the game market. Mobile devices with AR technology used for gaming can offer a new gaming experience for these players and enhance their immersion in the gameplay. In this study, we designed and tested Nostalgic Seekers, a cognitive-based collaborative board game integrating AR technology for Taiwanese players 50 years and older. This game provides players a chance to use cognitive skills because it requires discussion to problem solve and achieve tasks. The study found that the use of AR technology was enticing to participants; assembling the puzzles was challenging, but participants felt a sense of achievement when they completed a puzzle and were able to see their prize in the AR model.

## Appendix

Multimedia Appendix 1 Original survey form in Chinese.

Multimedia Appendix 2 Translated survey form.

## Abbreviations

2D: two-dimensional
3D: three-dimensional
AR: augmented reality
IPA: interaction process analysis
SDG: Sustainable Development Goals
VR: virtual reality
