# Supplementary material for: A Cognitive-Based Board Game With Augmented Reality for Older Adults: Development and Usability Study
Source: JMIR Serious Games. 2020 Dec 14;8(4):e22007. doi: 10.2196/22007 (PMC7769693; doi:10.2196/22007)
Supplement: Multimedia Appendix 1 [file games_v8i4e22007_app1.pdf]

60年代印象之問卷調查

親愛的朋友，您好：
感謝您撥空填寫這一份問卷，本問卷目的是在探討50歲~59歲的人對於60年代有那些印象及看法。問卷採匿名的方式，而且您在問卷中所提供的資訊，僅作為學術研究之用，不會提供其他單位，敬請安心填寫。 您的意見對我們非常重要，衷心期盼您依自己的實際感受填答。感謝您的熱情支持與協助！
敬祝　平安快樂，萬事如意！

*必填

基本資料

性別 *

- 男
- 女

請問您的教育程度？ *

- 國中以下
- 國中
- 高中
- 大學
- 研究所以上

請問您的年齡？ *

您的回答

請問您的職業？ *

您的回答

60年代印象

1. 對於這張圖片有熟悉感。 *

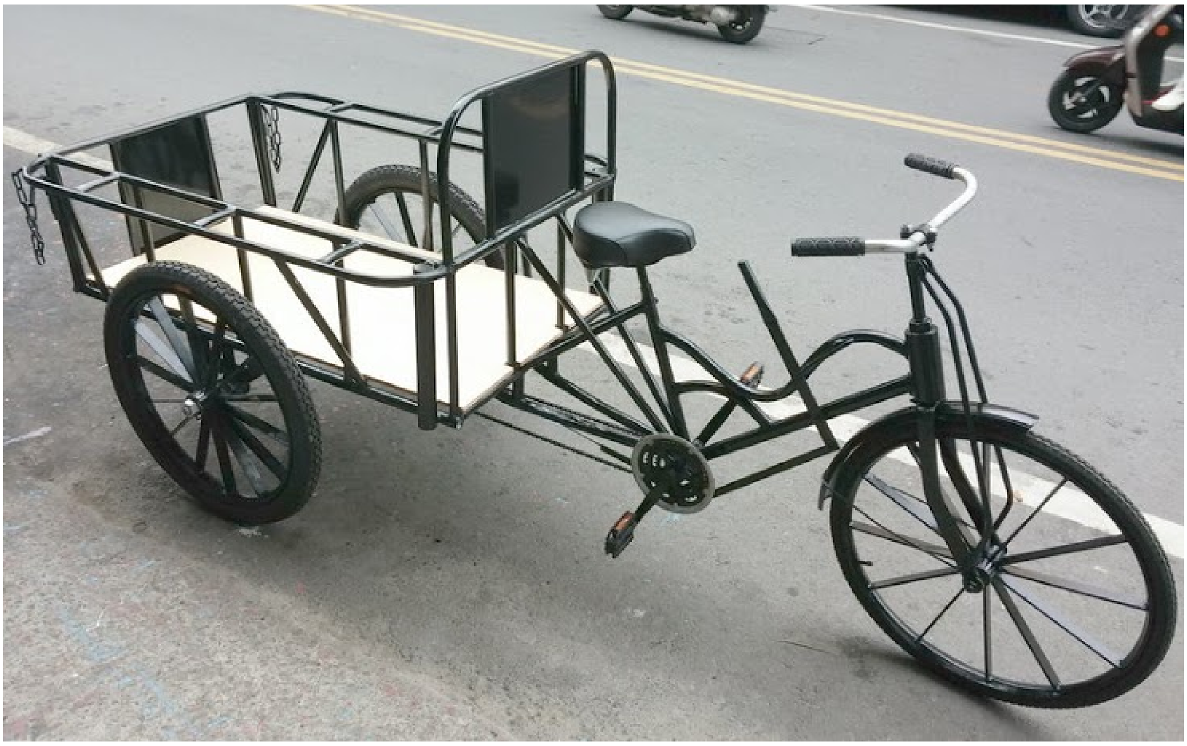

三輪車

- ◯ 非常不同意
- ◯ 不同意
- ◯ 有點不同意
- ◯ 普通
- ◯ 有點同意
- ◯ 同意
- ◯ 非常同意

2. 對於這張圖片有熟悉感。 *

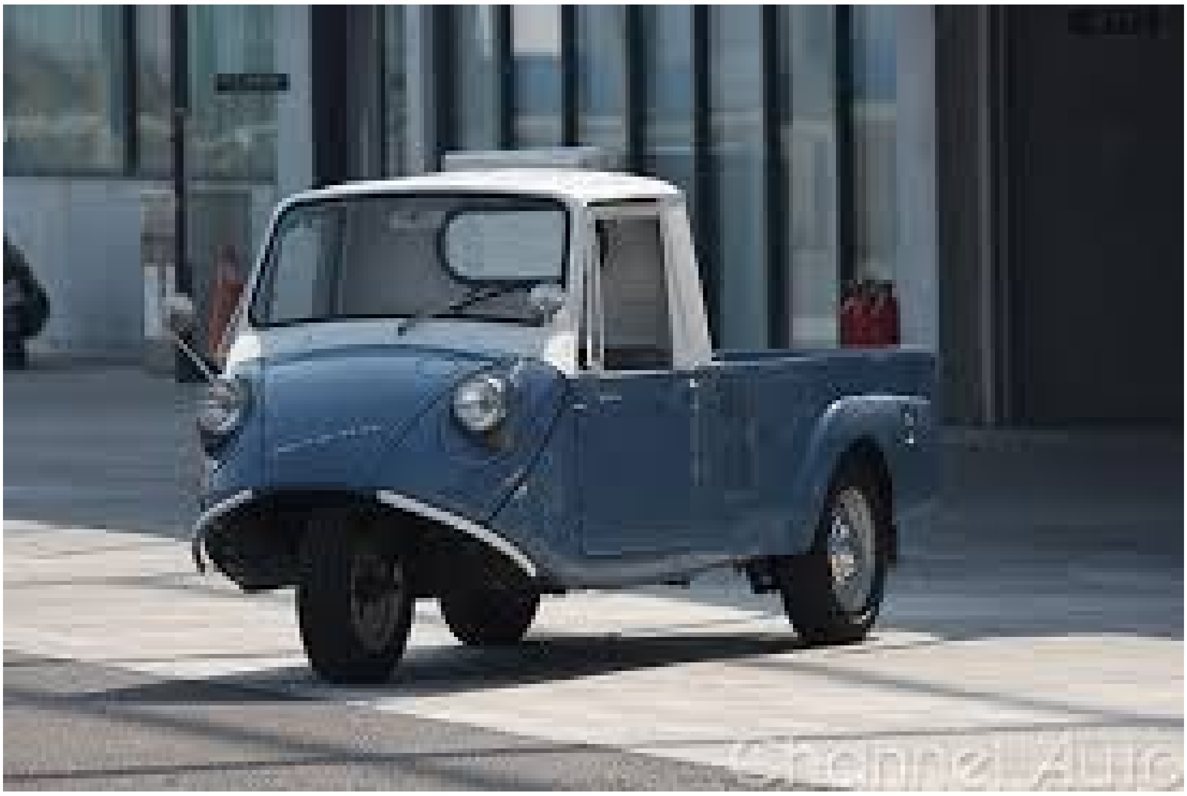

三輪貨車

- ◯ 非常不同意
- ◯ 不同意
- ◯ 有點不同意
- ◯ 普通
- ◯ 有點同意
- ◯ 同意
- ◯ 非常同意

3. 對於這張圖片有熟悉感。 *

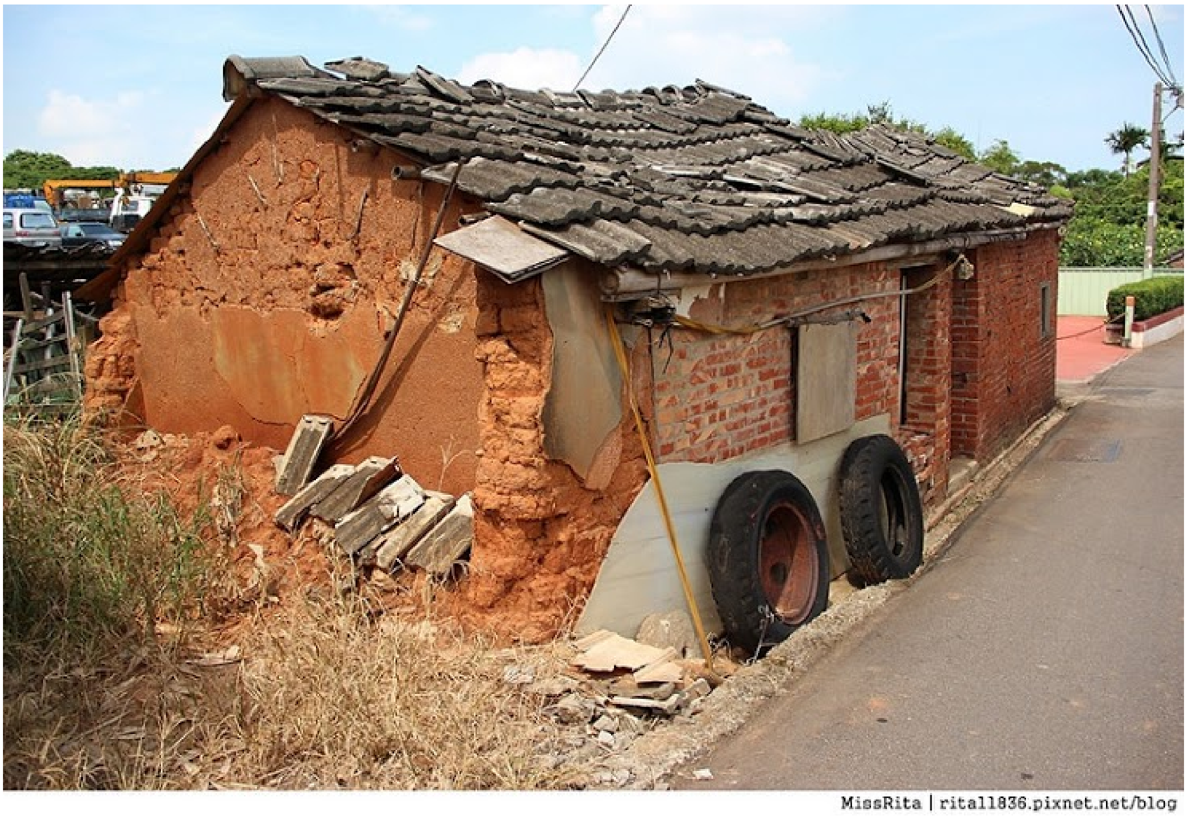

土角厝

- ○ 非常不同意
- ○ 不同意
- ○ 有點不同意
- ○ 普通
- ○ 有點同意
- ○ 同意
- ○ 非常同意

4. 對於這張圖片有熟悉感。 *

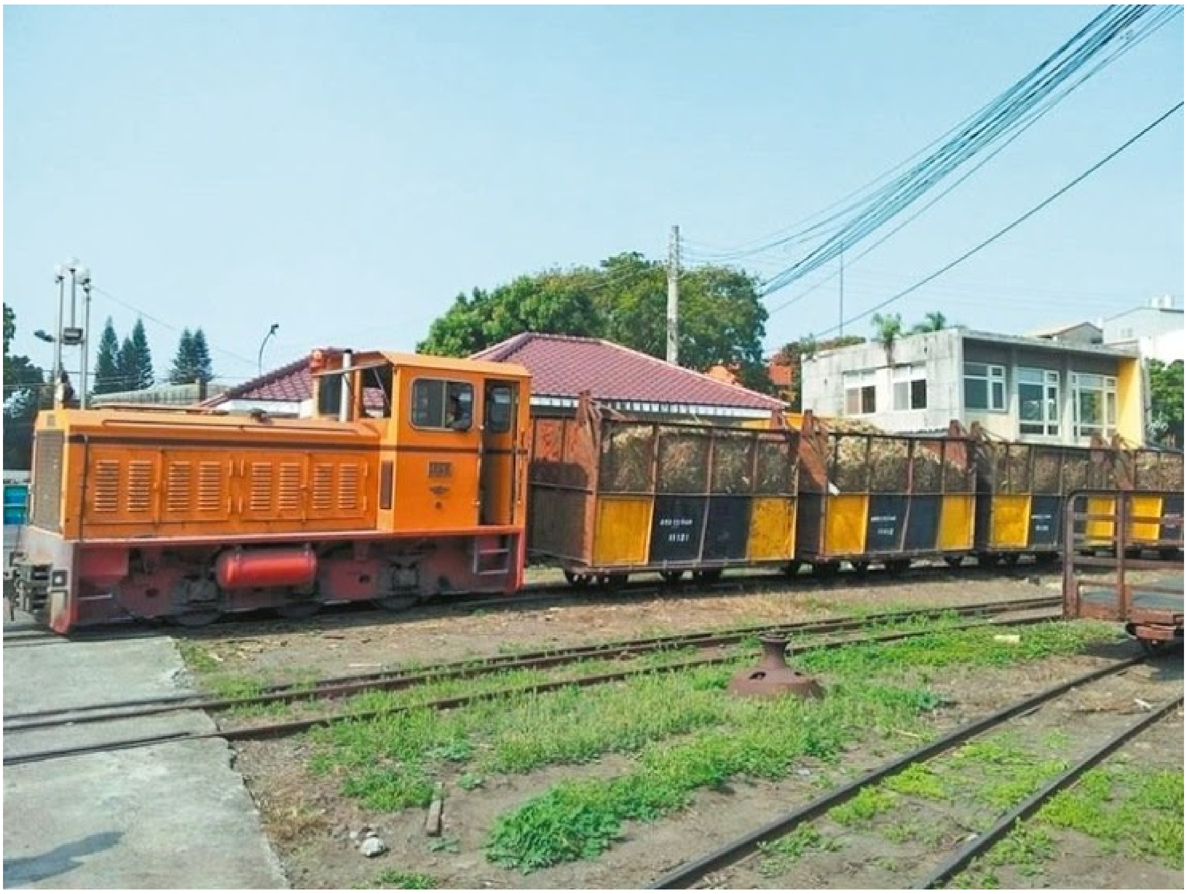

五分車

- ◯ 非常不同意
- ◯ 不同意
- ◯ 有點不同意
- ◯ 普通
- ◯ 有點同意
- ◯ 同意
- ◯ 非常同意

5. 對於這張圖片有熟悉感。 *

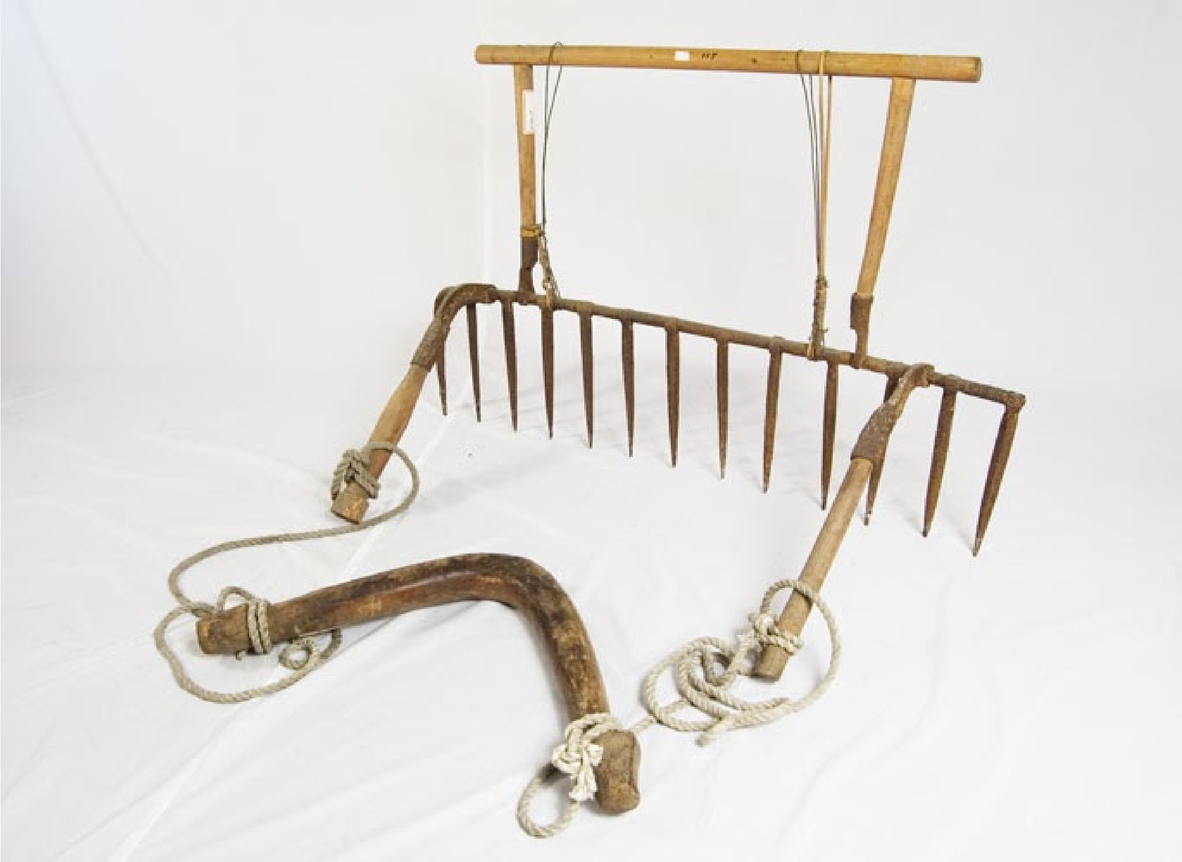

手耙

- 非常不同意
- 不同意
- 有點不同意
- 普通
- 有點同意
- 同意
- 非常同意

6. 對於這張圖片有熟悉感。 *

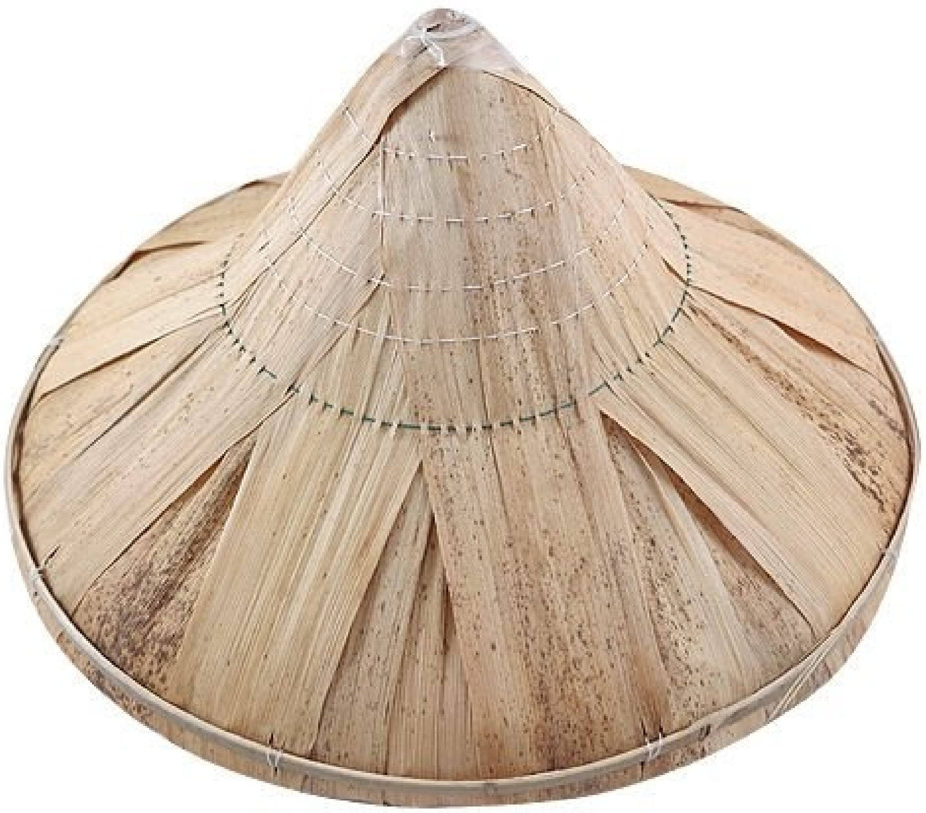

斗笠

- 非常不同意
- 不同意
- 有點不同意
- 普通
- 有點同意
- 同意
- 非常同意

7. 對於這張圖片有熟悉感。 *

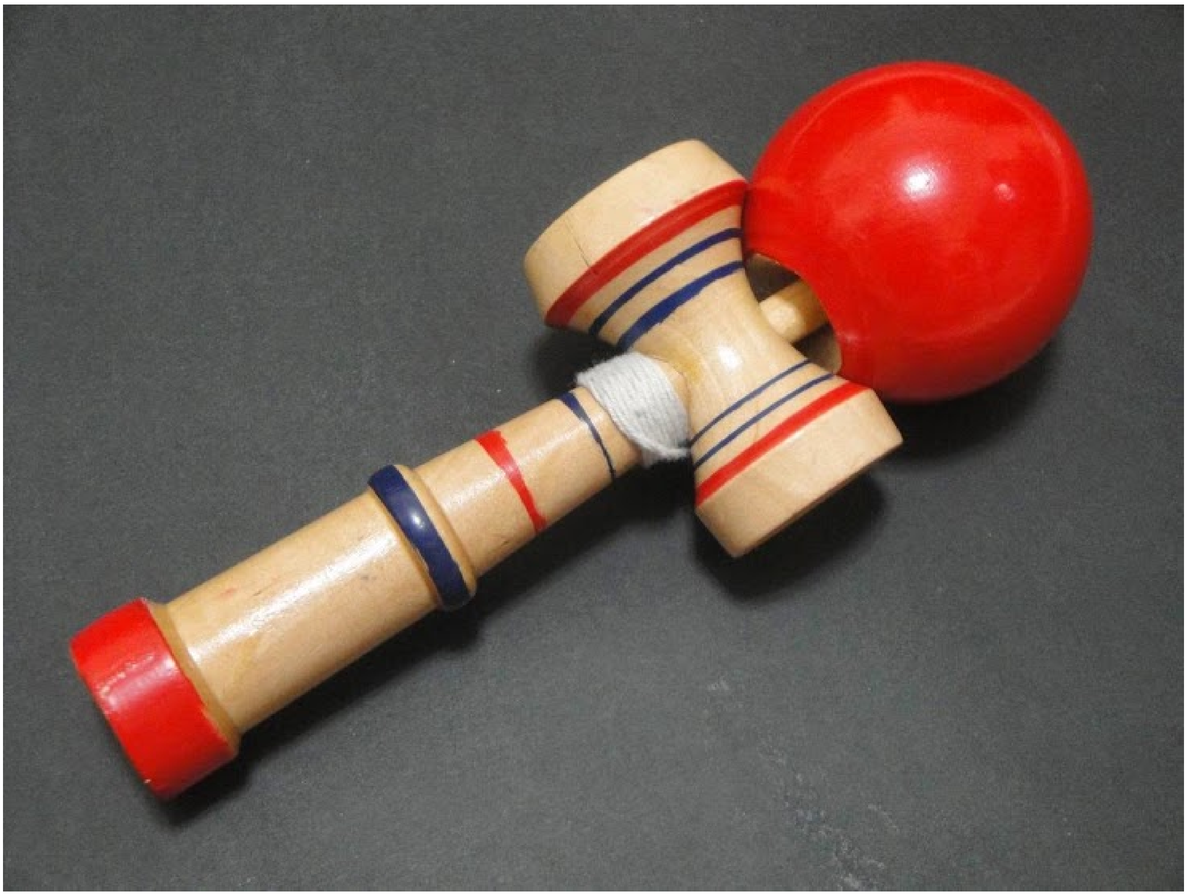

日月球

- ◯ 非常不同意
- ◯ 不同意
- ◯ 有點不同意
- ◯ 普通
- ◯ 有點同意
- ◯ 同意
- ◯ 非常同意

8. 對於這張圖片有熟悉感。 *

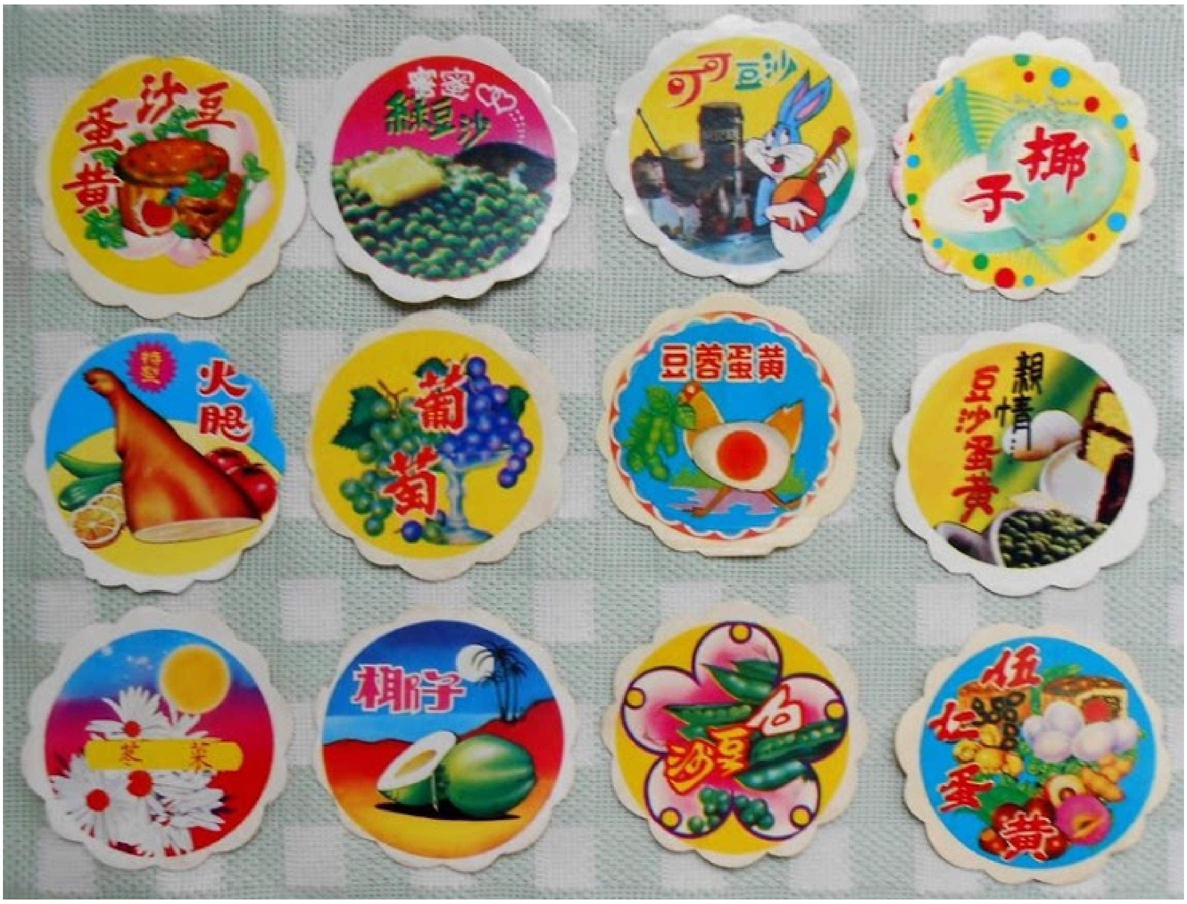

月餅標籤紙

- ◯ 非常不同意
- ◯ 不同意
- ◯ 有點不同意
- ◯ 普通
- ◯ 有點同意
- ◯ 同意
- ◯ 非常同意

9. 對於這張圖片有熟悉感。 *

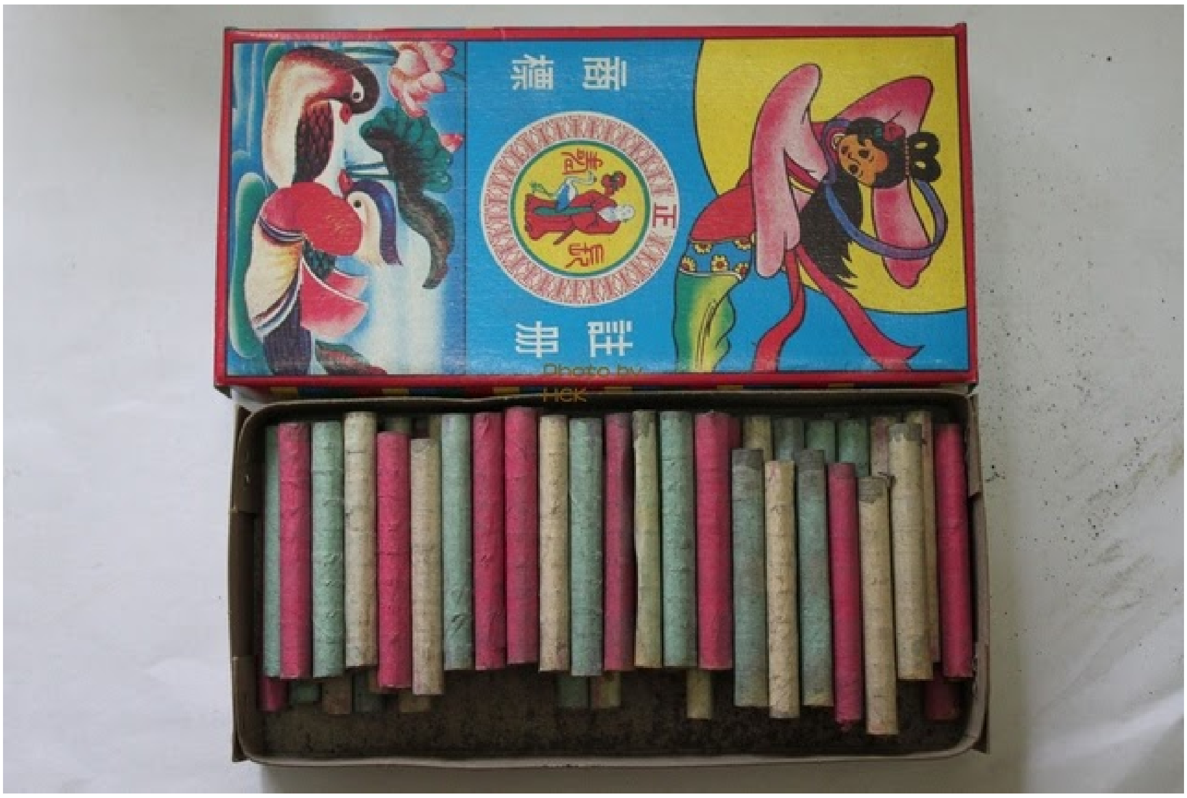

水鴛鴦

- 非常不同意
- 不同意
- 有點不同意
- 普通
- 有點同意
- 同意
- 非常同意

10. 對於這張圖片有熟悉感。 *

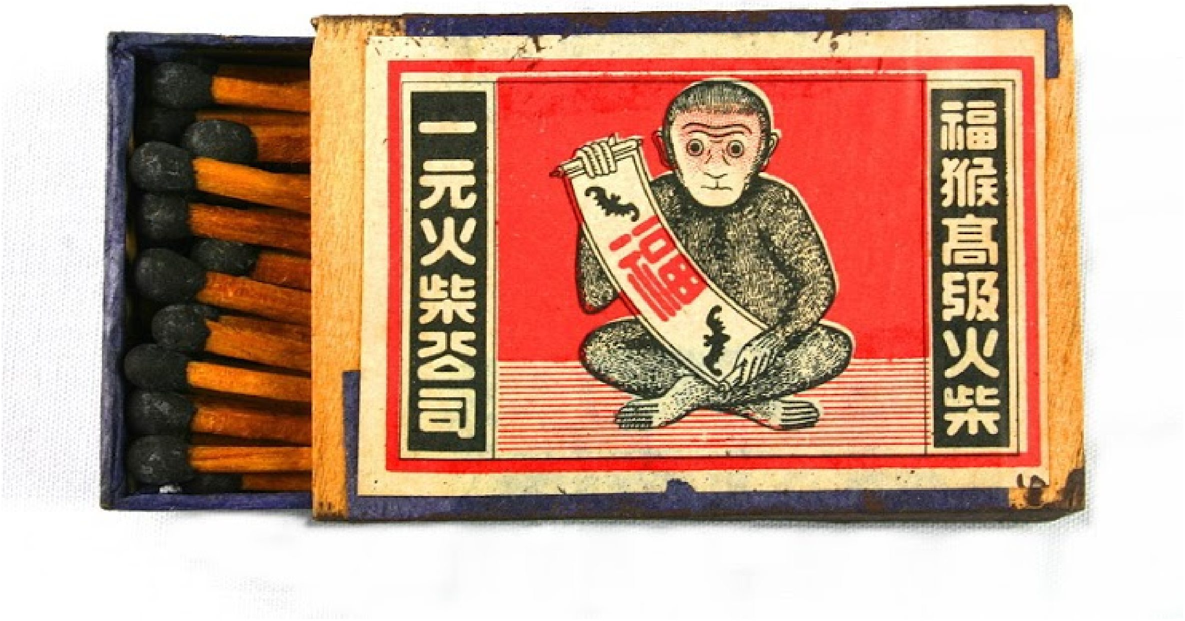

火柴盒

- ◯ 非常不同意
- ◯ 不同意
- ◯ 有點不同意
- ◯ 普通
- ◯ 有點同意
- ◯ 同意
- ◯ 非常同意

11. 對於這張圖片有熟悉感。 *

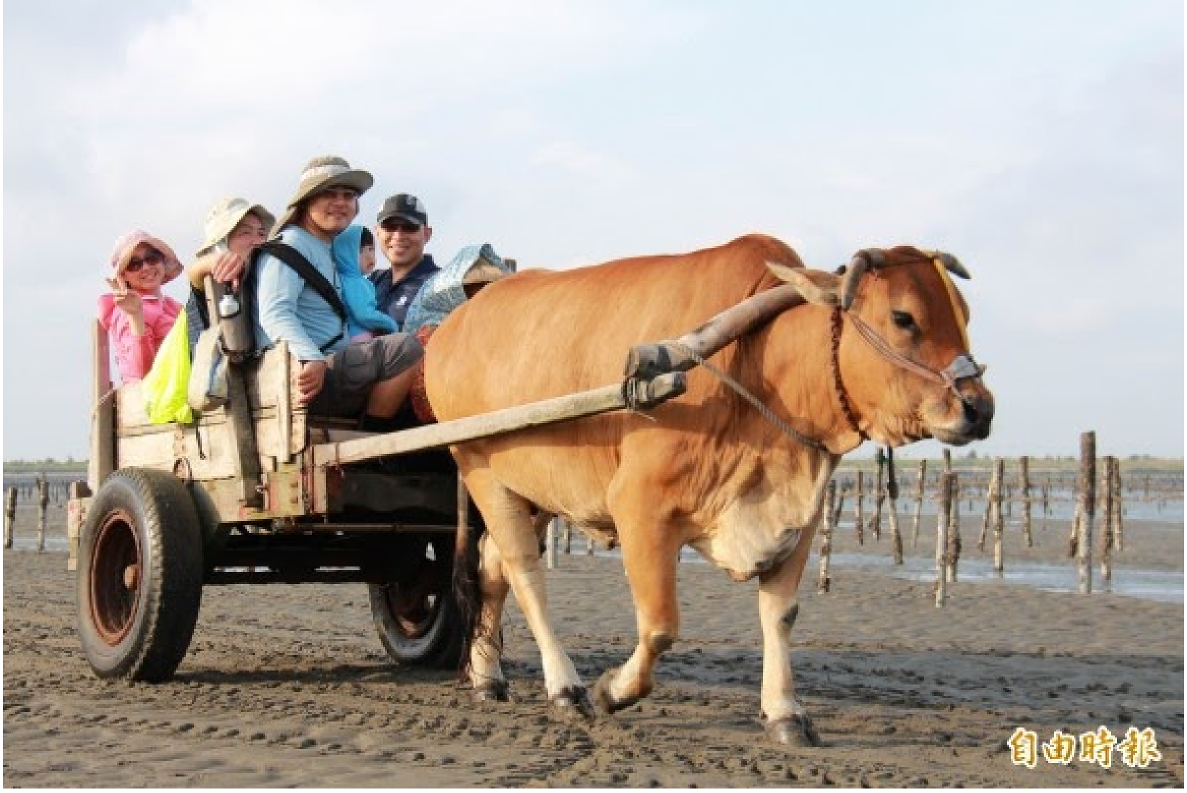

牛車

- ○ 非常不同意
- ○ 不同意
- ○ 有點不同意
- ○ 普通
- ○ 有點同意
- ○ 同意
- ○ 非常同意

12. 對於這張圖片有熟悉感。 *

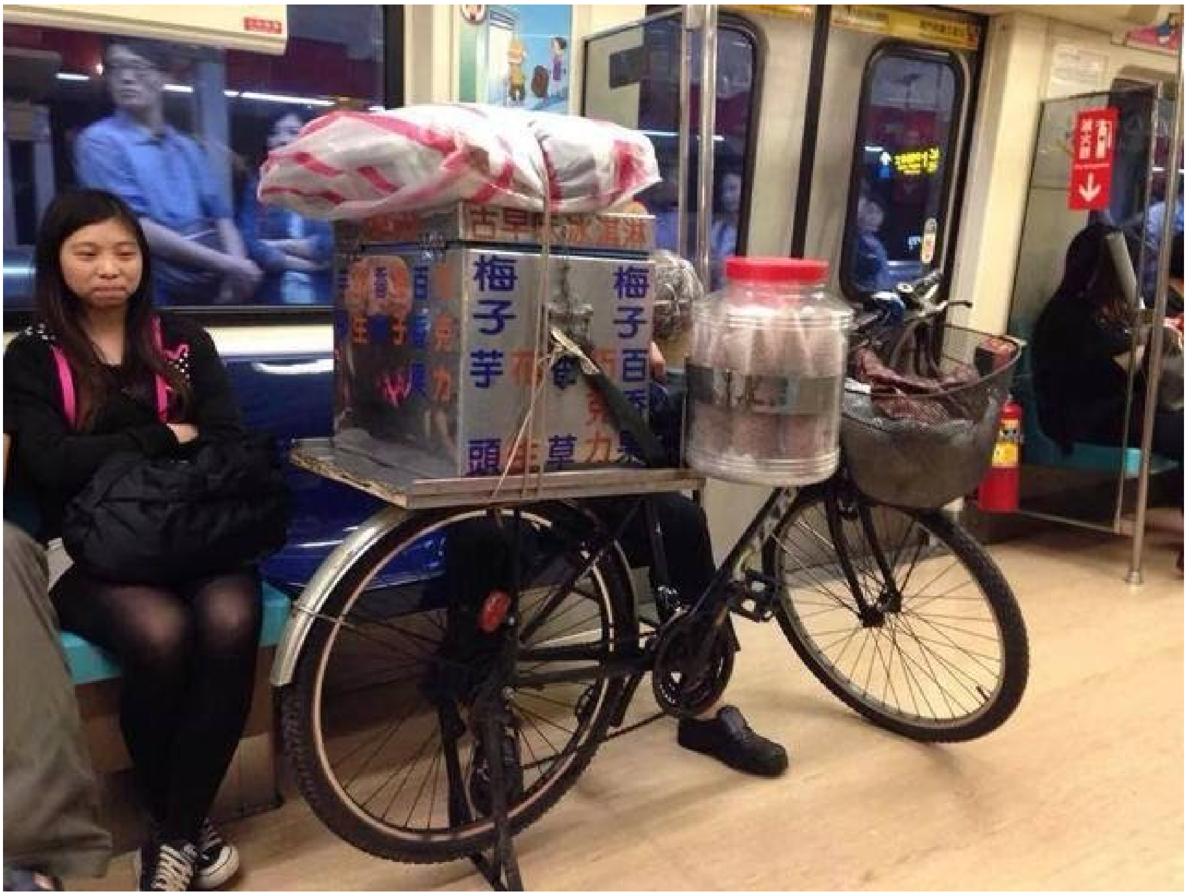

叭噗車

- 非常不同意
- 不同意
- 有點不同意
- 普通
- 有點同意
- 同意
- 非常同意

13. 對於這張圖片有熟悉感。 *

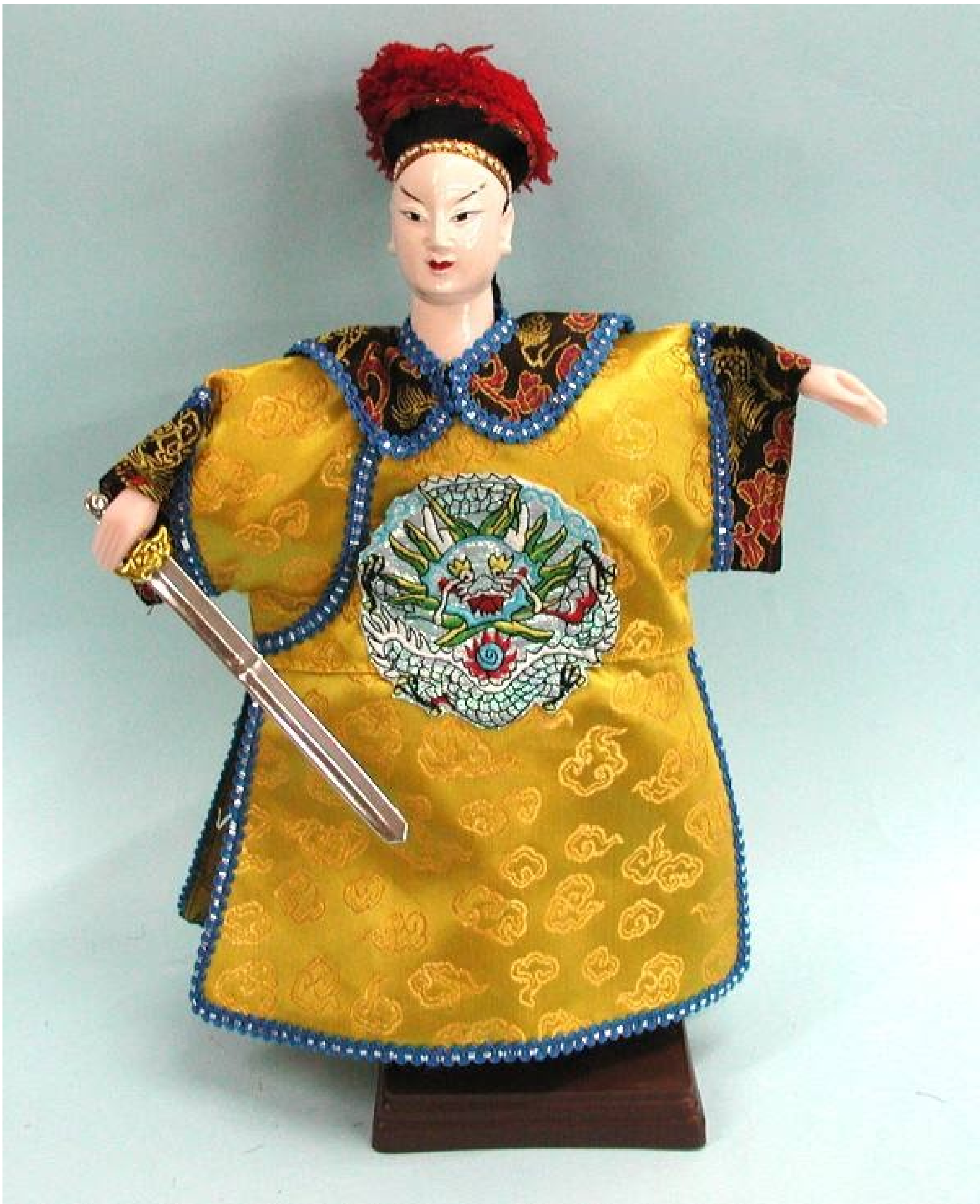

布袋戲布偶

- 非常不同意
- 不同意
- 有點不同意
- 普通
- 有點同意
- 同意

同意

非常同意

14. 對於這張圖片有熟悉感。 *

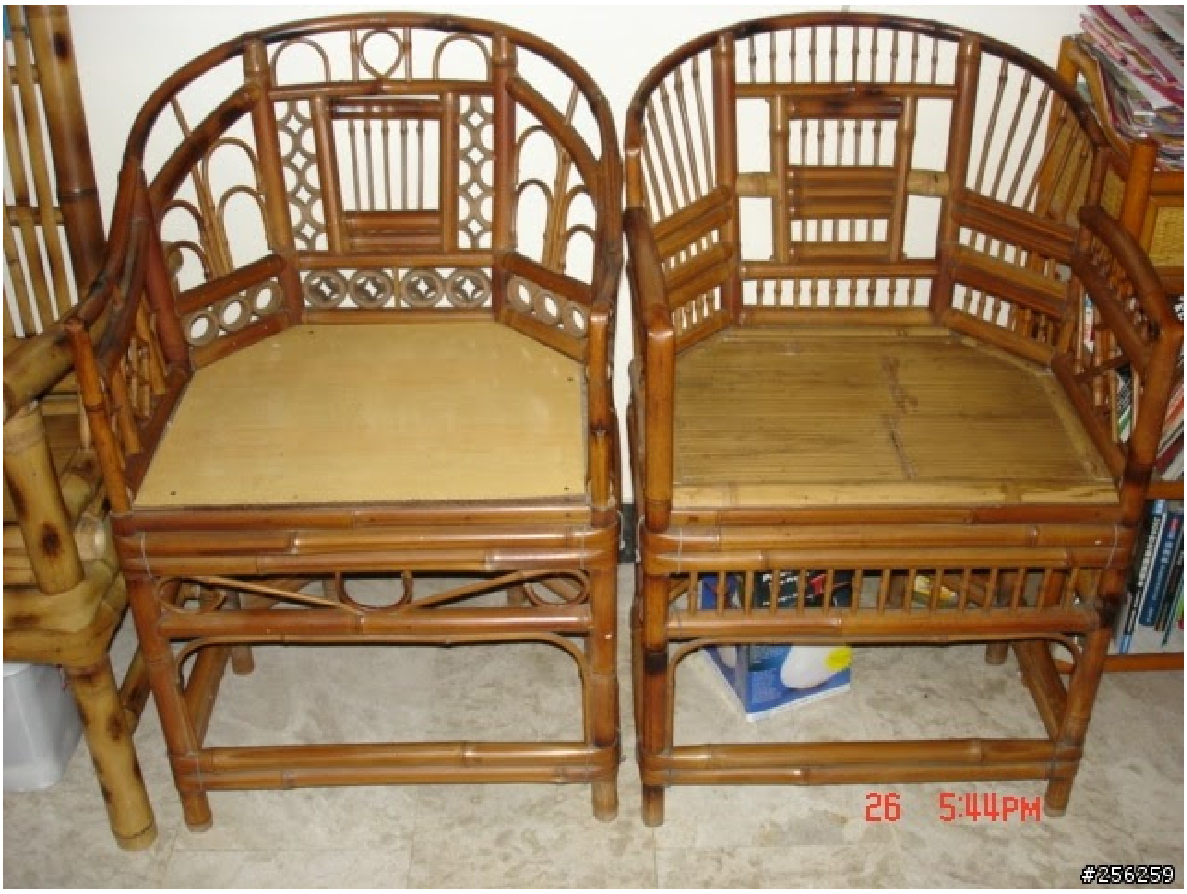

竹椅

- 非常不同意
- 不同意
- 有點不同意
- 普通
- 有點同意
- 同意
- 非常同意

15. 對於這張圖片有熟悉感。 *

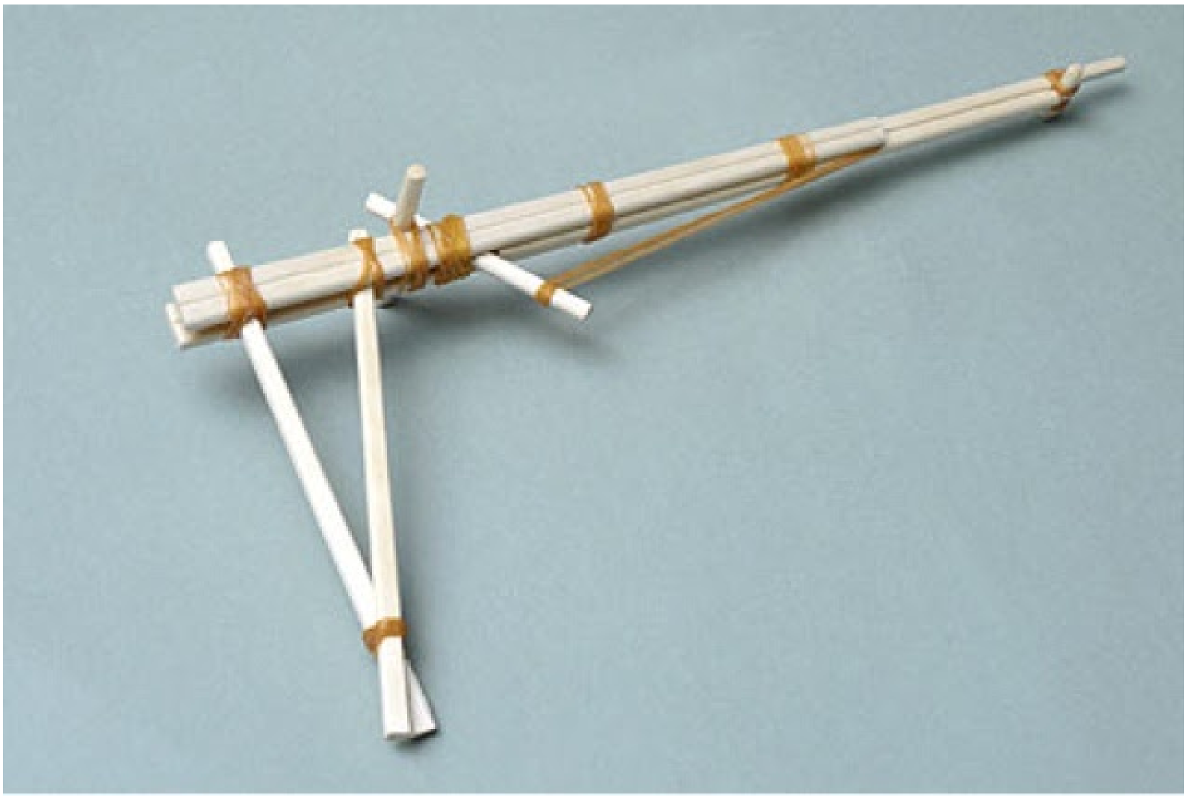

竹槍

◯ 非常不同意

◯ 不同意

◯ 有點不同意

◯ 普通

◯ 有點同意

◯ 同意

◯ 非常同意

16. 對於這張圖片有熟悉感。 *

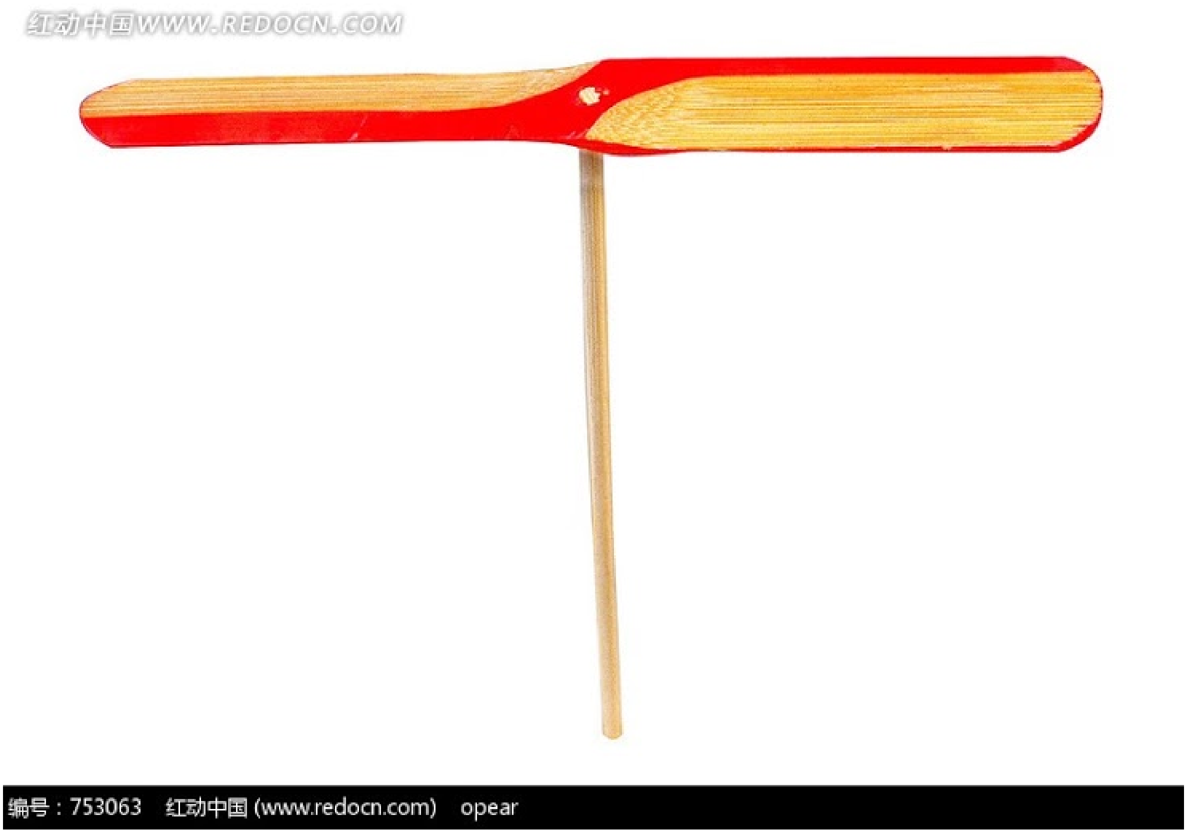

竹蜻蜓

- 非常不同意
- 不同意
- 有點不同意
- 普通
- 有點同意
- 同意
- 非常同意

17. 對於這張圖片有熟悉感。 *

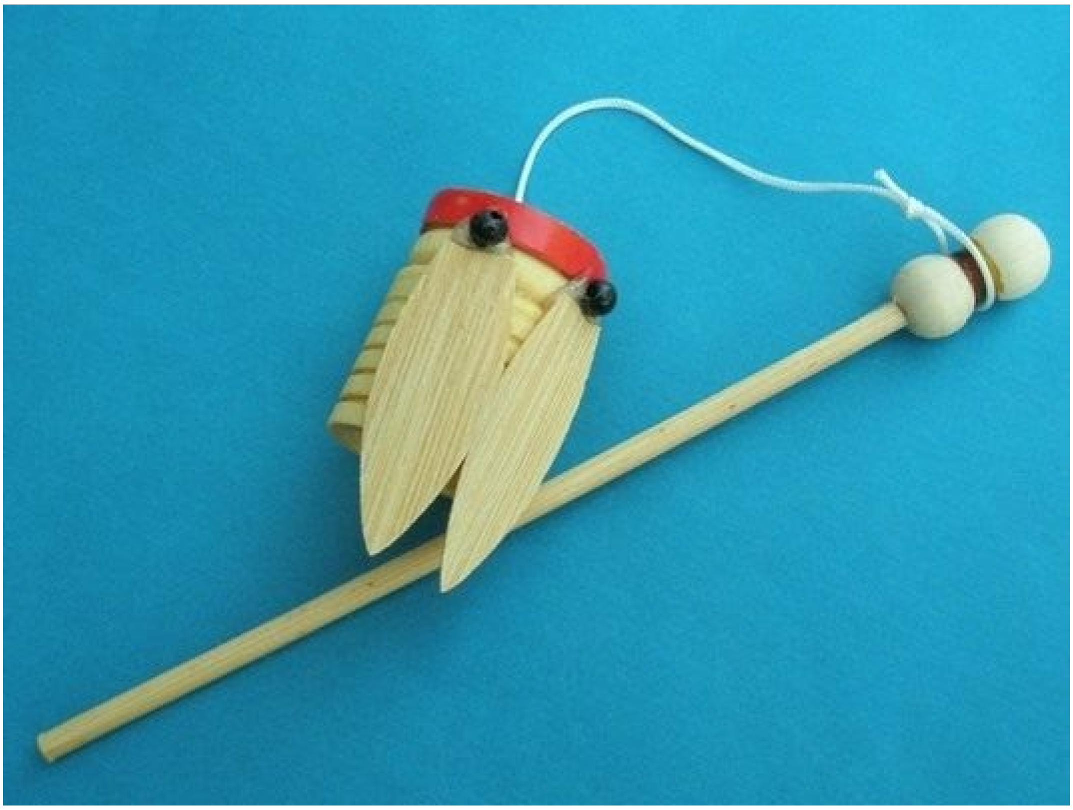

竹蟬

- ◯ 非常不同意
- ◯ 不同意
- ◯ 有點不同意
- ◯ 普通
- ◯ 有點同意
- ◯ 同意
- ◯ 非常同意

18. 對於這張圖片有熟悉感。 *

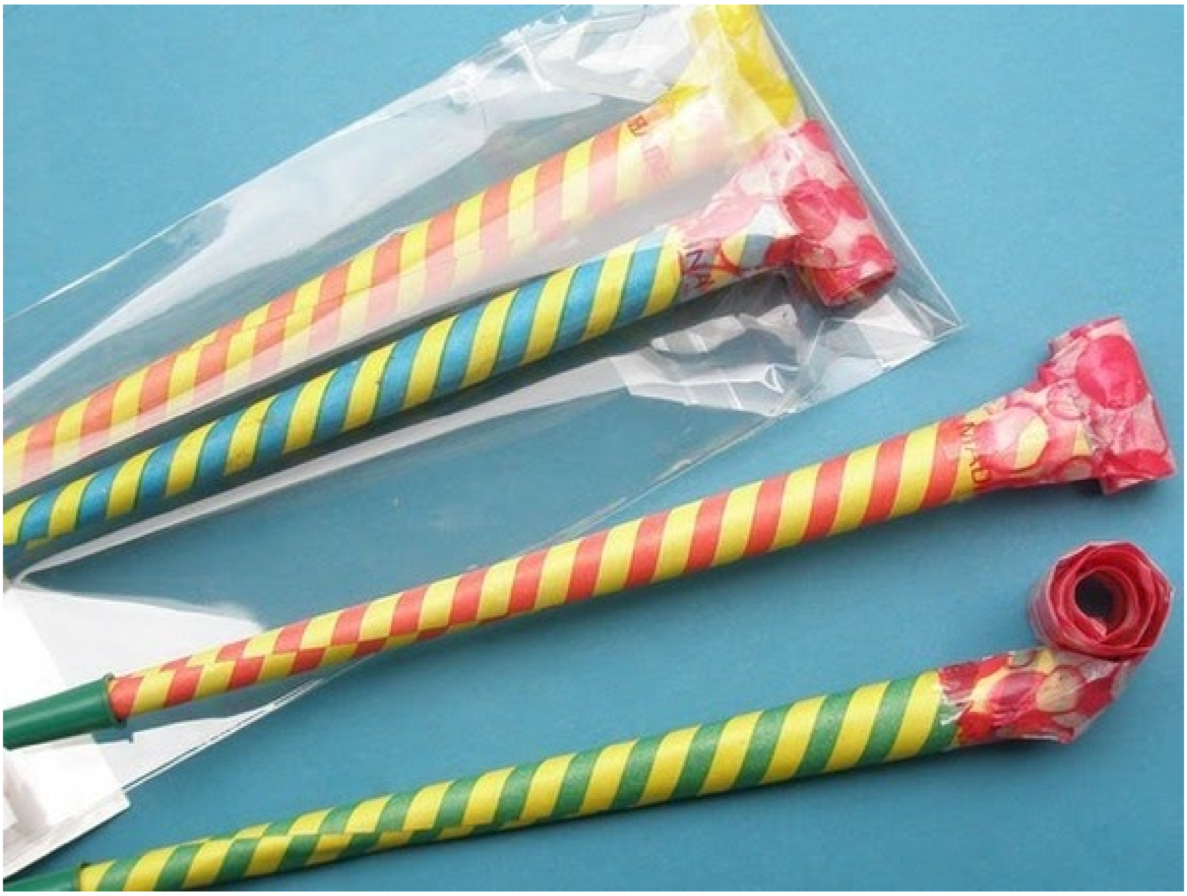

伸縮象鼻

- ○ 非常不同意
- ○ 不同意
- ○ 有點不同意
- ○ 普通
- ○ 有點同意
- ○ 同意
- ○ 非常同意

19. 對於這張圖片有熟悉感。 *

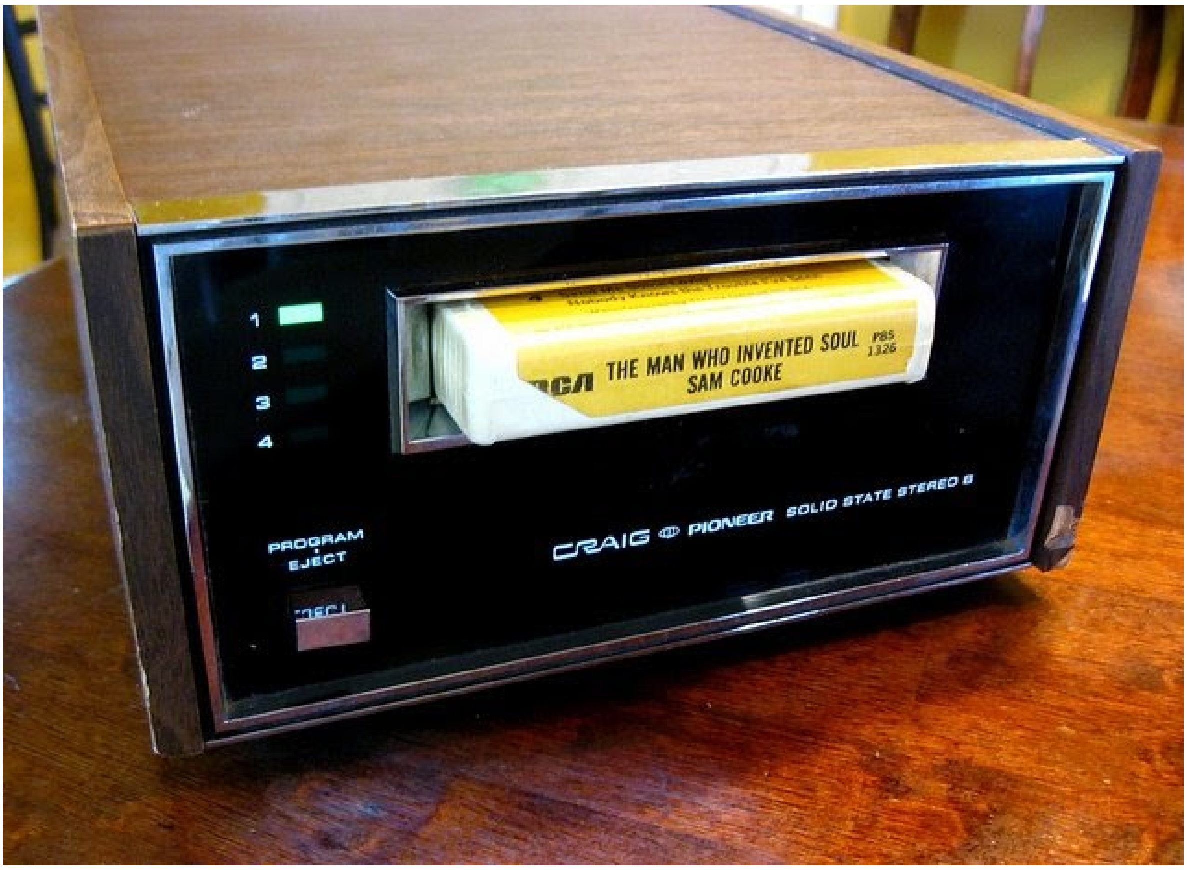

匣式錄音帶

- ◯ 非常不同意
- ◯ 不同意
- ◯ 有點不同意
- ◯ 普通
- ◯ 有點同意
- ◯ 同意
- ◯ 非常同意

20. 對於這張圖片有熟悉感。 *

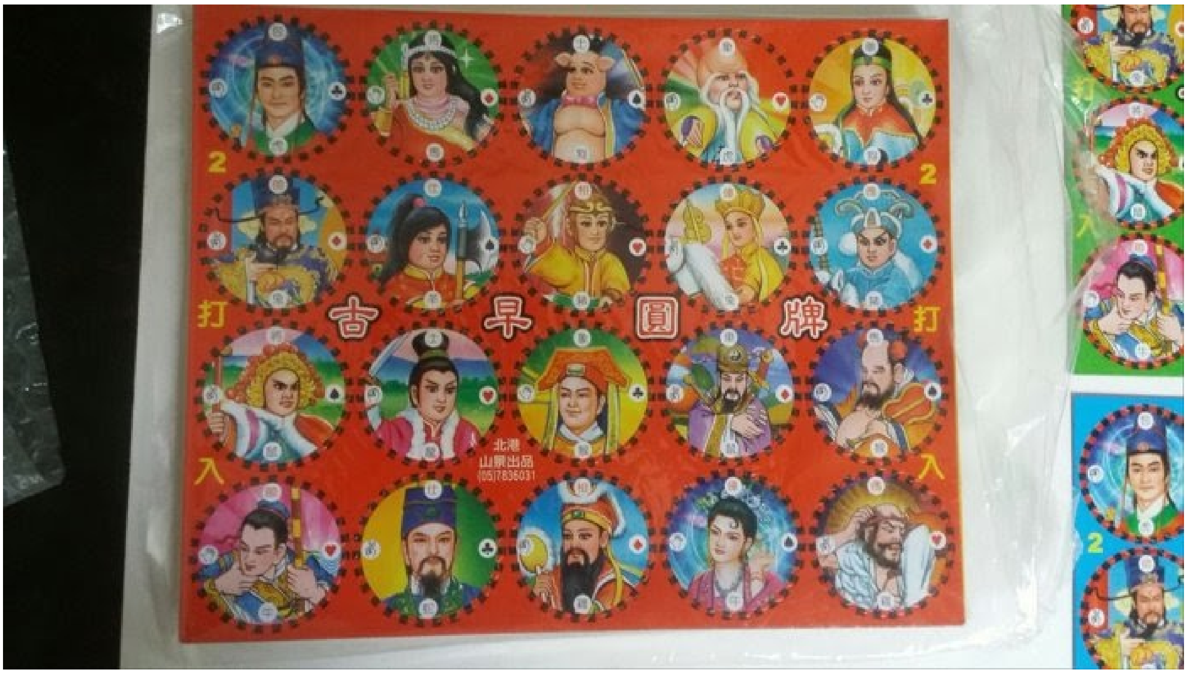

尪仔標

- ◯ 非常不同意
- ◯ 不同意
- ◯ 有點不同意
- ◯ 普通
- ◯ 有點同意
- ◯ 同意
- ◯ 非常同意

21. 對於這張圖片有熟悉感。 *

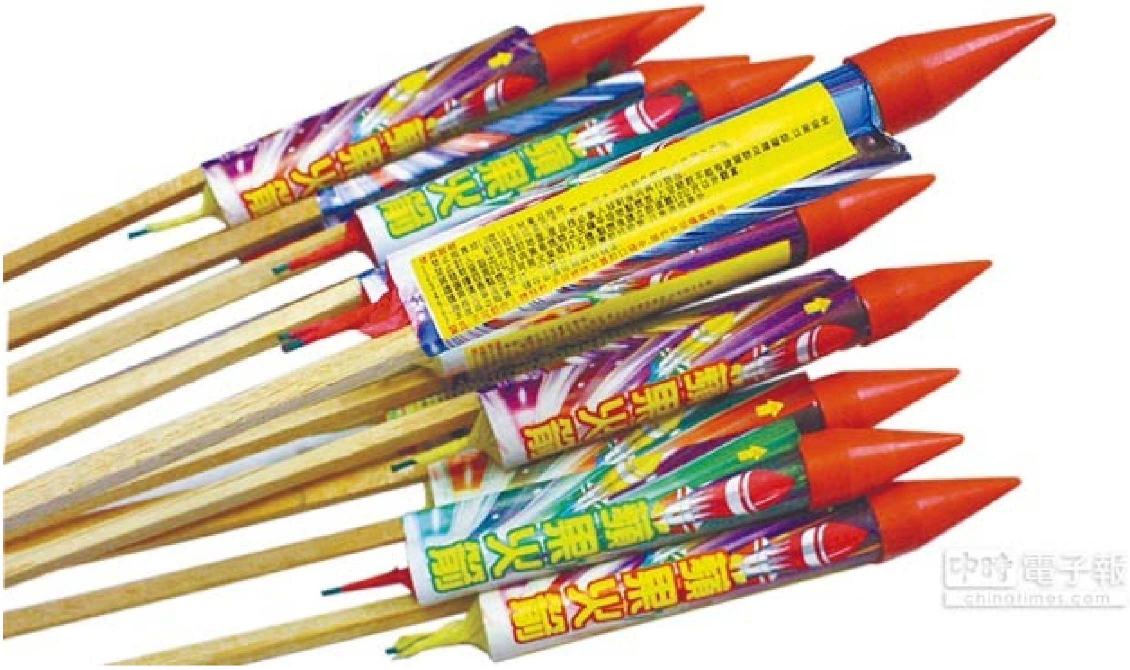

沖天炮

- 非常不同意
- 不同意
- 有點不同意
- 普通
- 有點同意
- 同意
- 非常同意

22. 對於這張圖片有熟悉感。 *

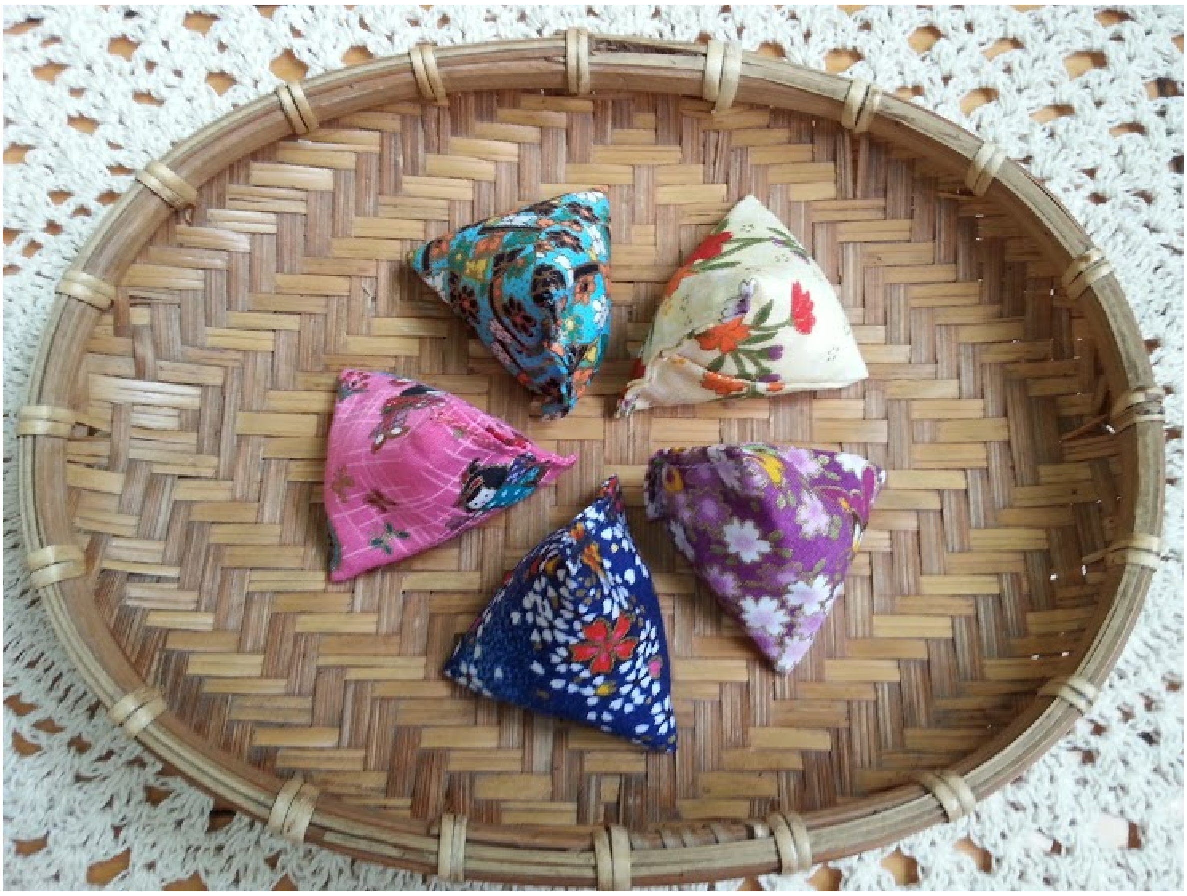

沙包

- 非常不同意
- 不同意
- 有點不同意
- 普通
- 有點同意
- 同意
- 非常同意

23. 對於這張圖片有熟悉感。 *

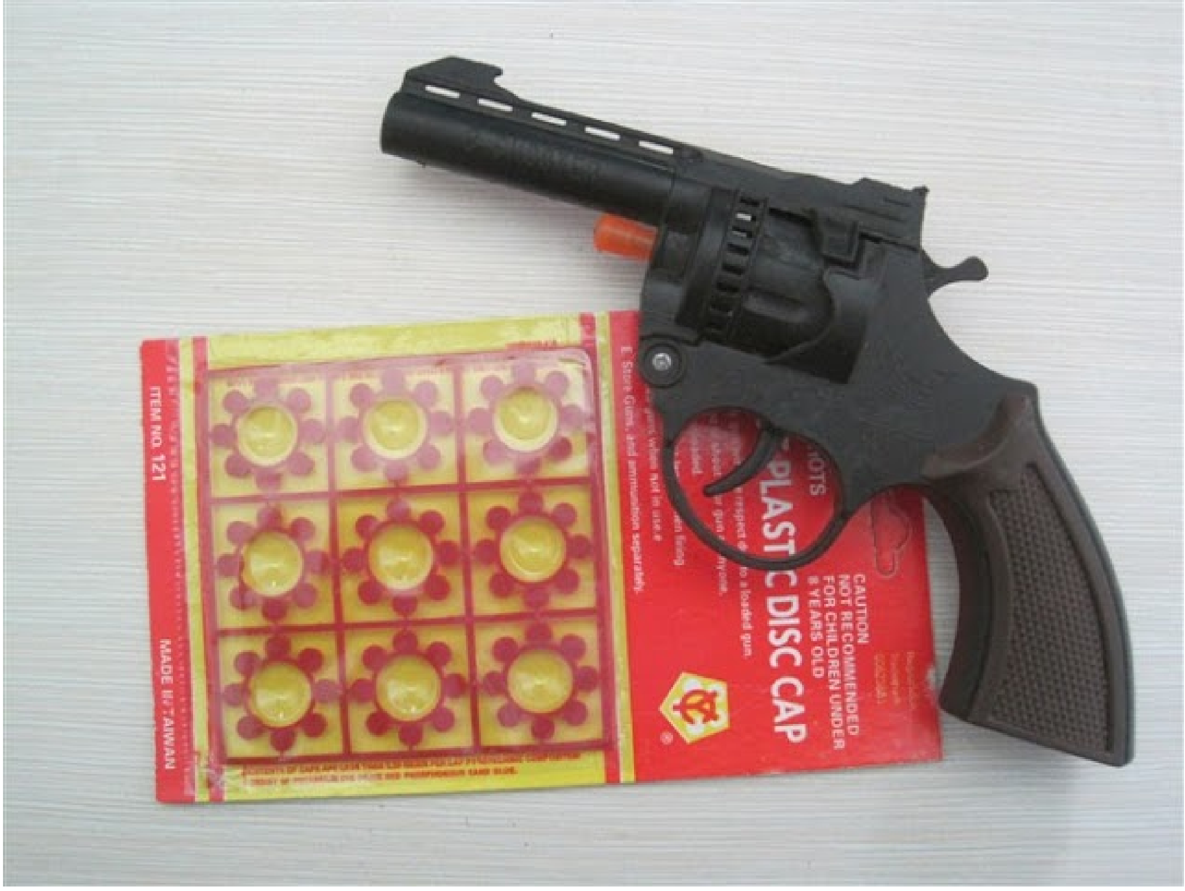

玩具左輪手槍

- ◯ 非常不同意
- ◯ 不同意
- ◯ 有點不同意
- ◯ 普通
- ◯ 有點同意
- ◯ 同意
- ◯ 非常同意

24. 對於這張圖片有熟悉感。 *

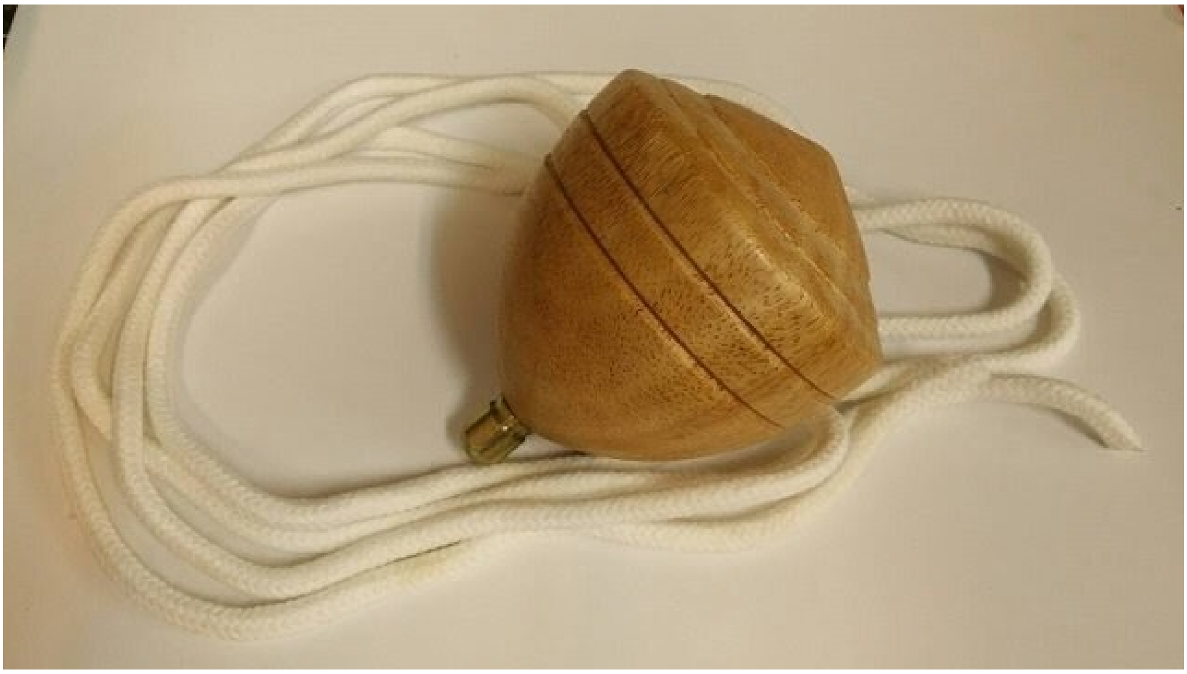

陀螺

- 非常不同意
- 不同意
- 有點不同意
- 普通
- 有點同意
- 同意
- 非常同意

25. 對於這張圖片有熟悉感。 *

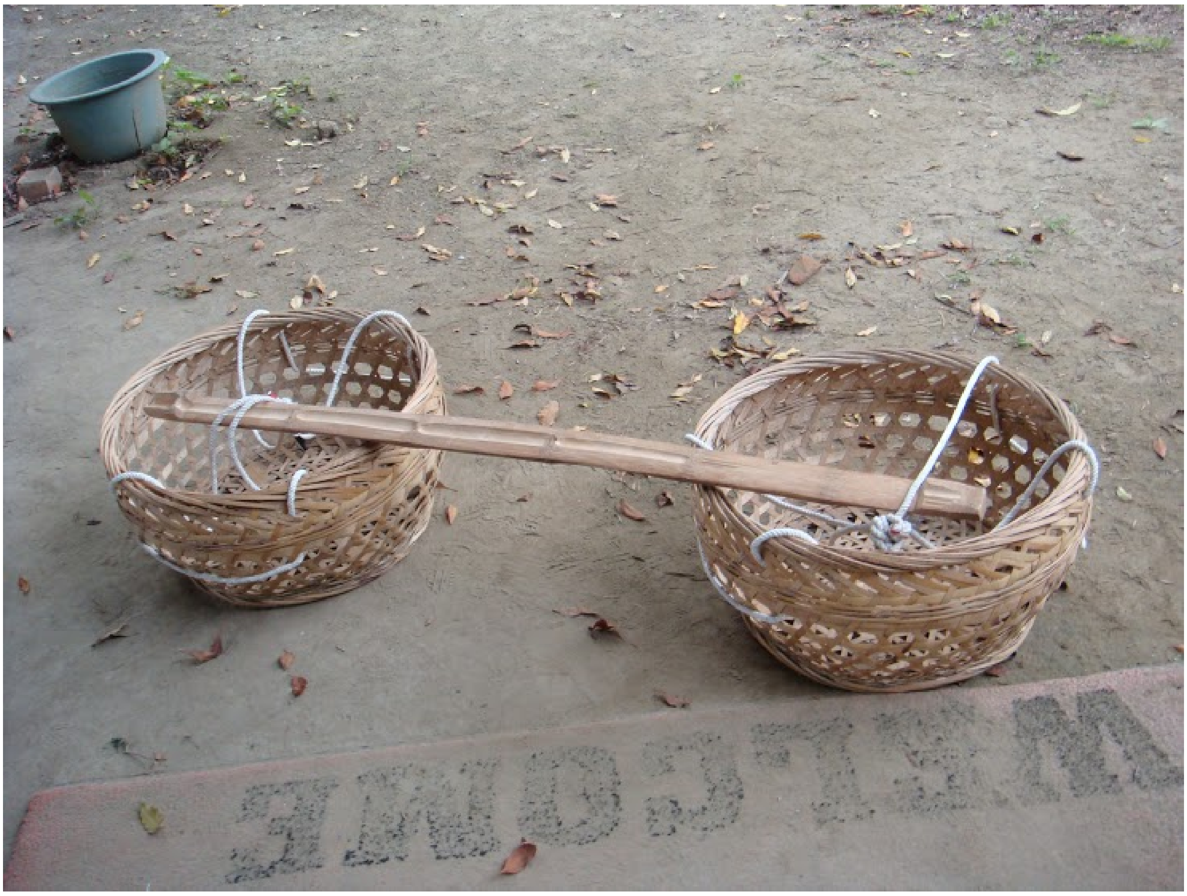

扁擔

- ◯ 非常不同意
- ◯ 不同意
- ◯ 有點不同意
- ◯ 普通
- ◯ 有點同意
- ◯ 同意
- ◯ 非常同意

26. 對於這張圖片有熟悉感。 *

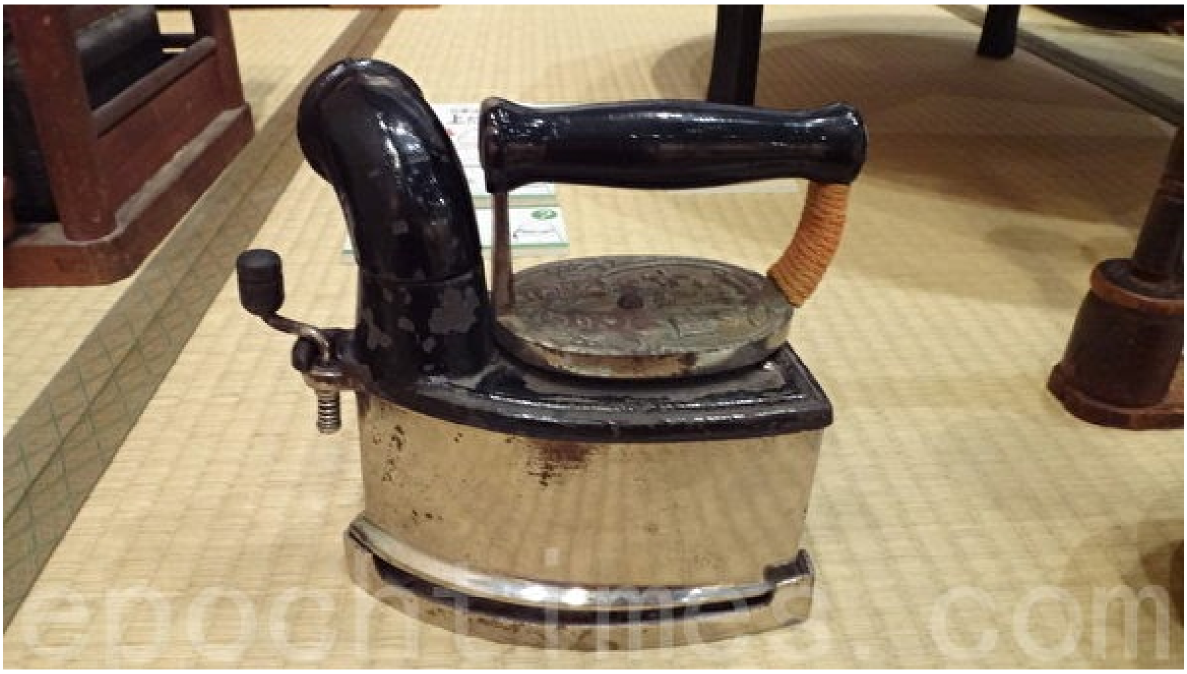

炭火熨斗

- 非常不同意
- 不同意
- 有點不同意
- 普通
- 有點同意
- 同意
- 非常同意

27. 對於這張圖片有熟悉感。 *

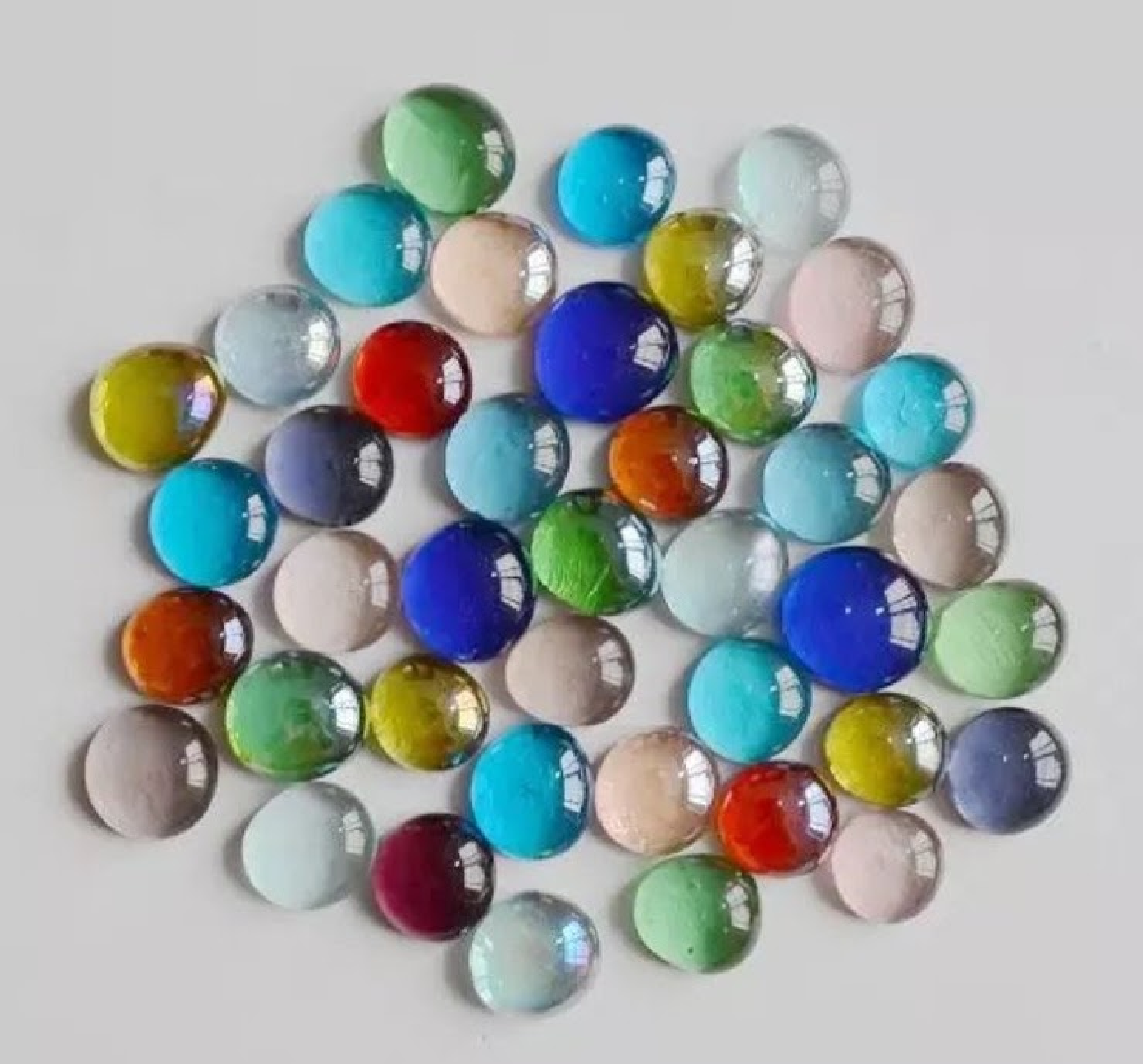

玻璃珠

- ◯ 非常不同意
- ◯ 不同意
- ◯ 有點不同意
- ◯ 普通
- ◯ 有點同意
- ◯ 同意
- ◯ 非常同意

28. 對於這張圖片有熟悉感。 *

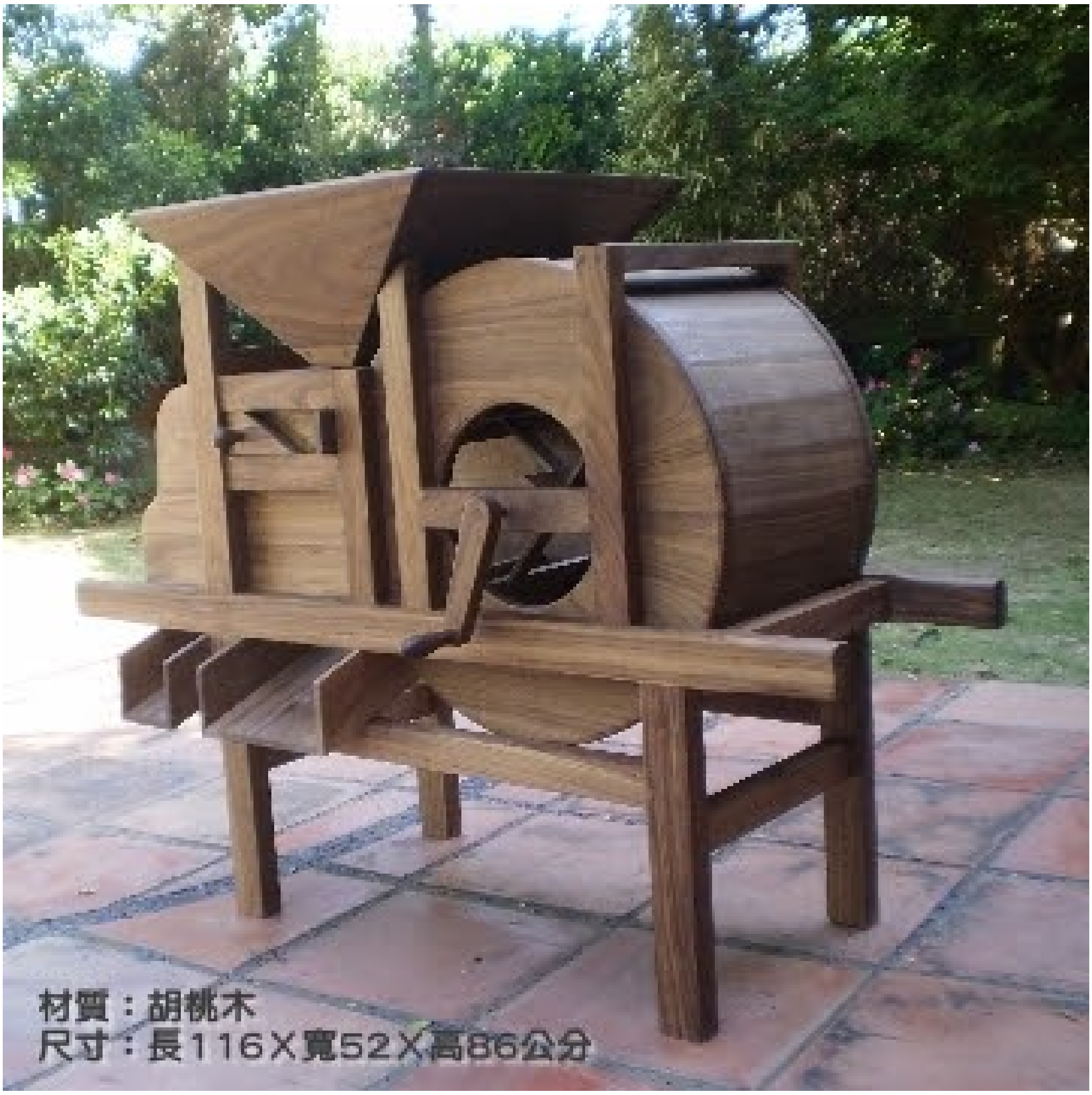

風鼓機

- 非常不同意
- 不同意
- 有點不同意
- 普通
- 有點同意
- 同意
- 非常同意

29. 對於這張圖片有熟悉感。 *

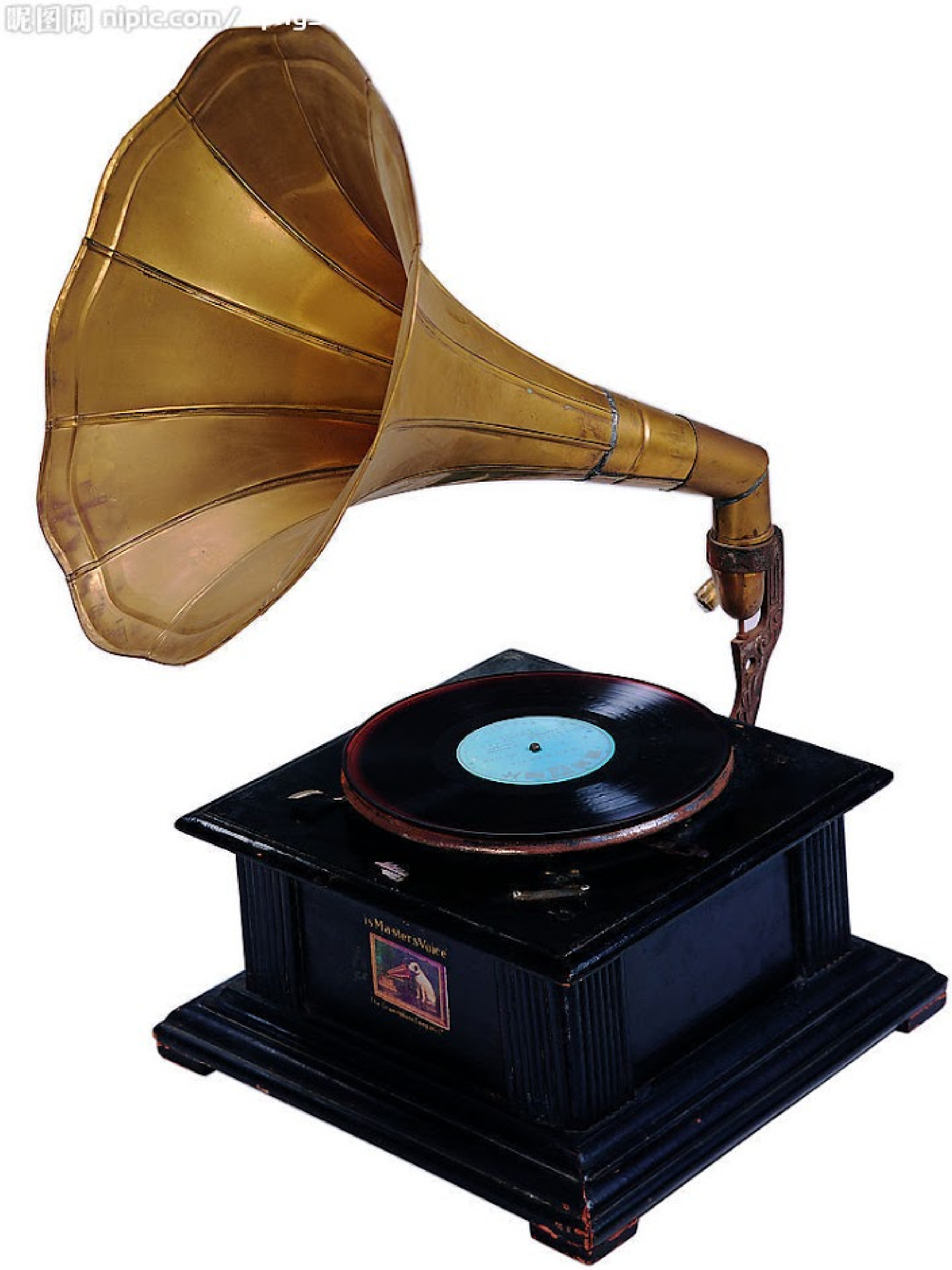

留聲機

- ◯ 非常不同意
- ◯ 不同意
- ◯ 有點不同意
- ◯ 普通

有點同意

同意

非常同意

30. 對於這張圖片有熟悉感。 *

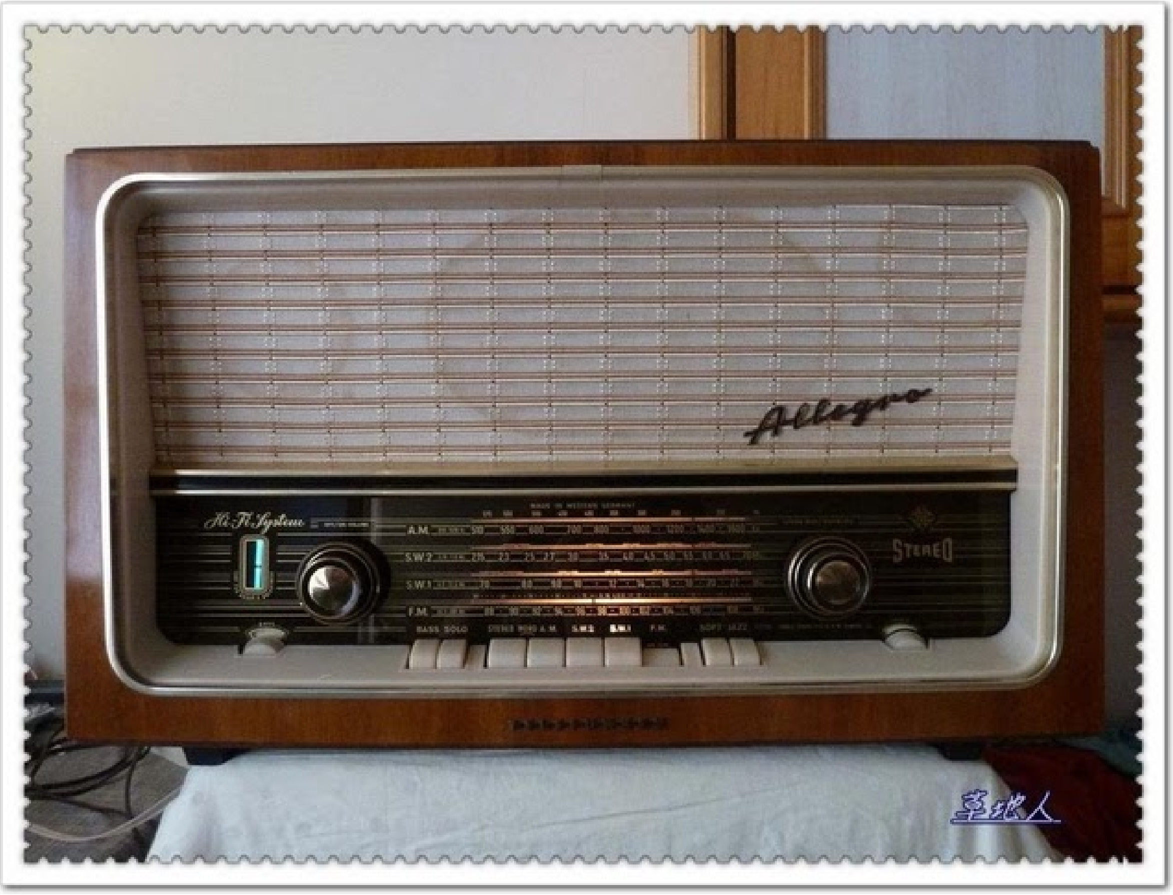

真空管收音機

非常不同意

不同意

有點不同意

普通

有點同意

同意

非常同意

31. 對於這張圖片有熟悉感。 *

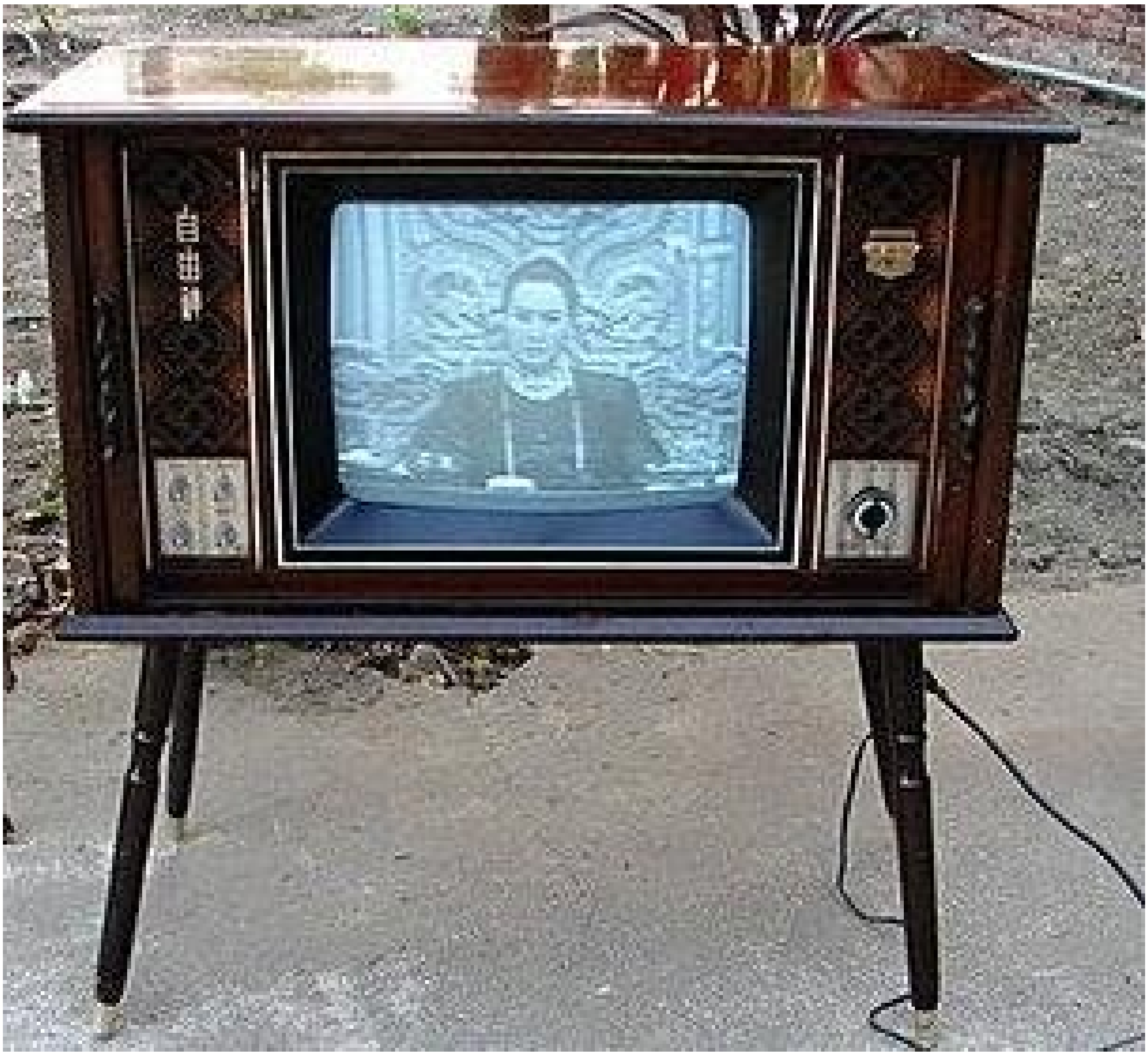

真空管電視

- ◯ 非常不同意
- ◯ 不同意
- ◯ 有點不同意
- ◯ 普通
- ◯ 有點同意
- ◯ 同意
- ◯ 非常同意

32. 對於這張圖片有熟悉感。 *

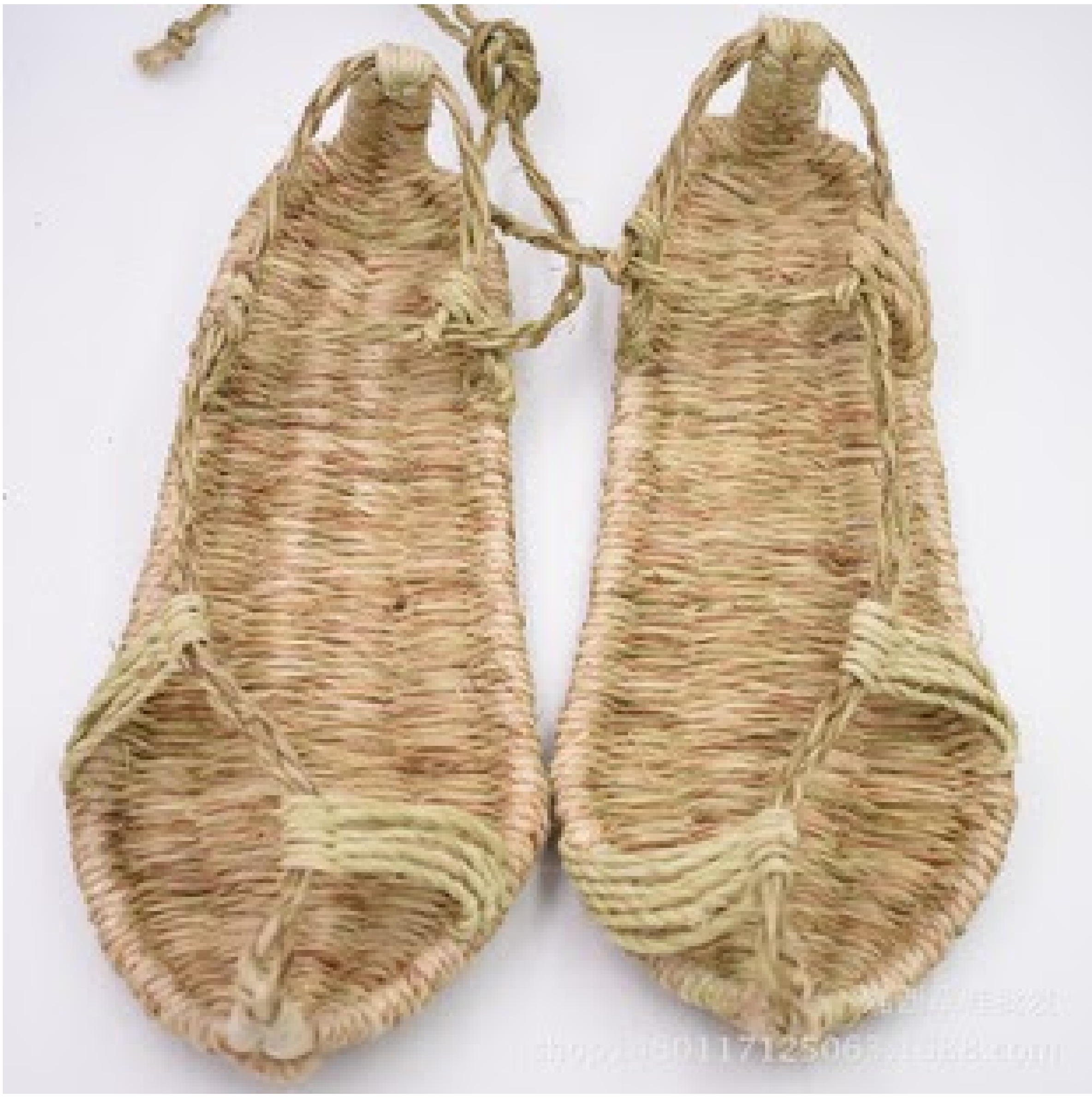

草鞋

- ◯ 非常不同意
- ◯ 不同意
- ◯ 有點不同意
- ◯ 普通
- ◯ 有點同意
- ◯ 同意
- ◯ 非常同意

33. 對於這張圖片有熟悉感。 *

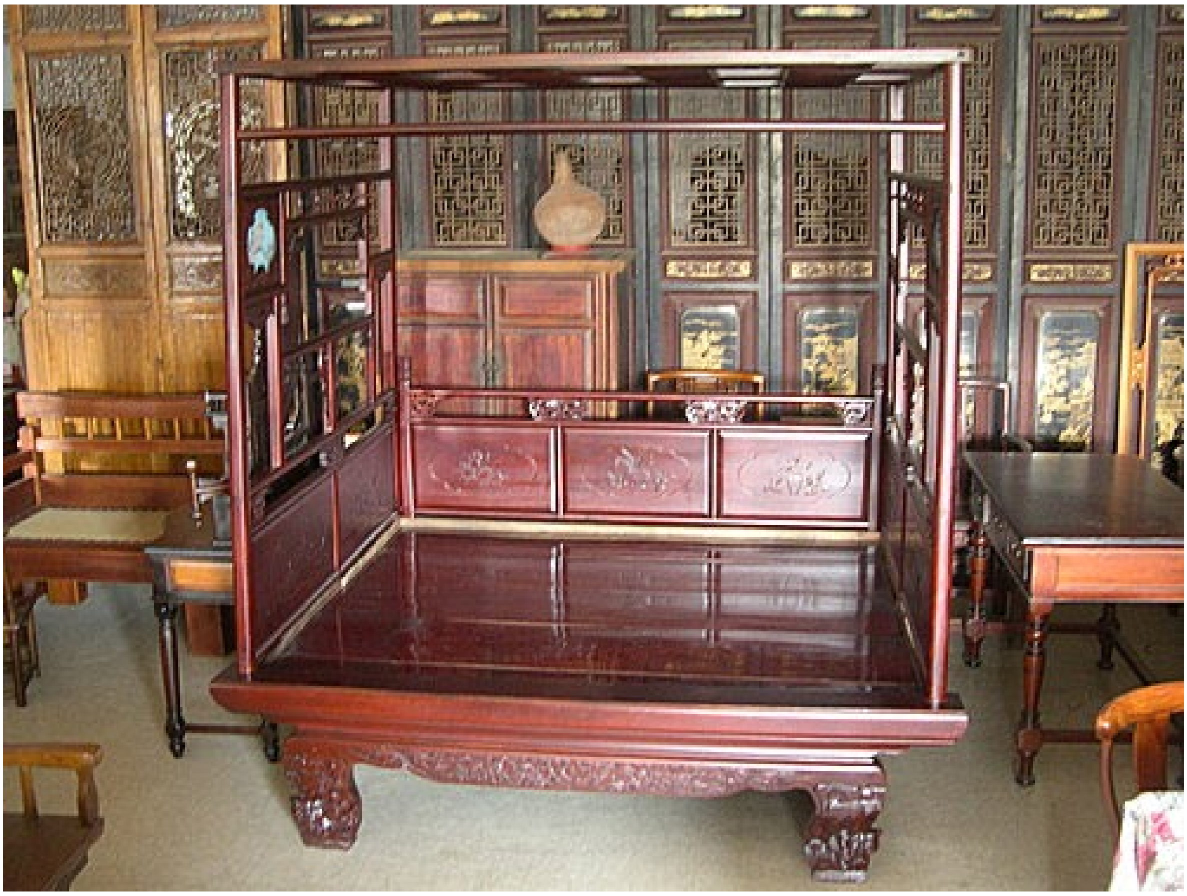

高架床

- 非常不同意
- 不同意
- 有點不同意
- 普通
- 有點同意
- 同意
- 非常同意

34. 對於這張圖片有熟悉感。 *

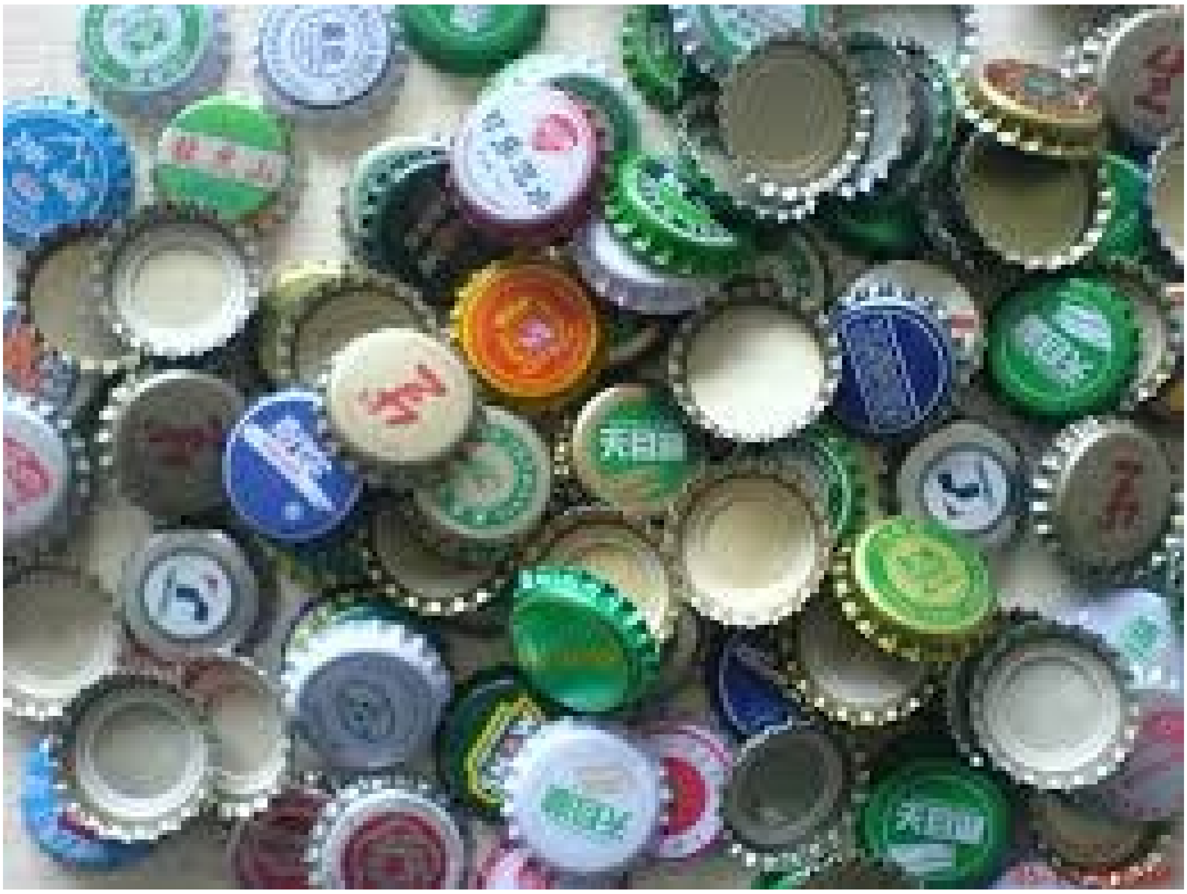

瓶蓋

- ◯ 非常不同意
- ◯ 不同意
- ◯ 有點不同意
- ◯ 普通
- ◯ 有點同意
- ◯ 同意
- ◯ 非常同意

35. 對於這張圖片有熟悉感。 *

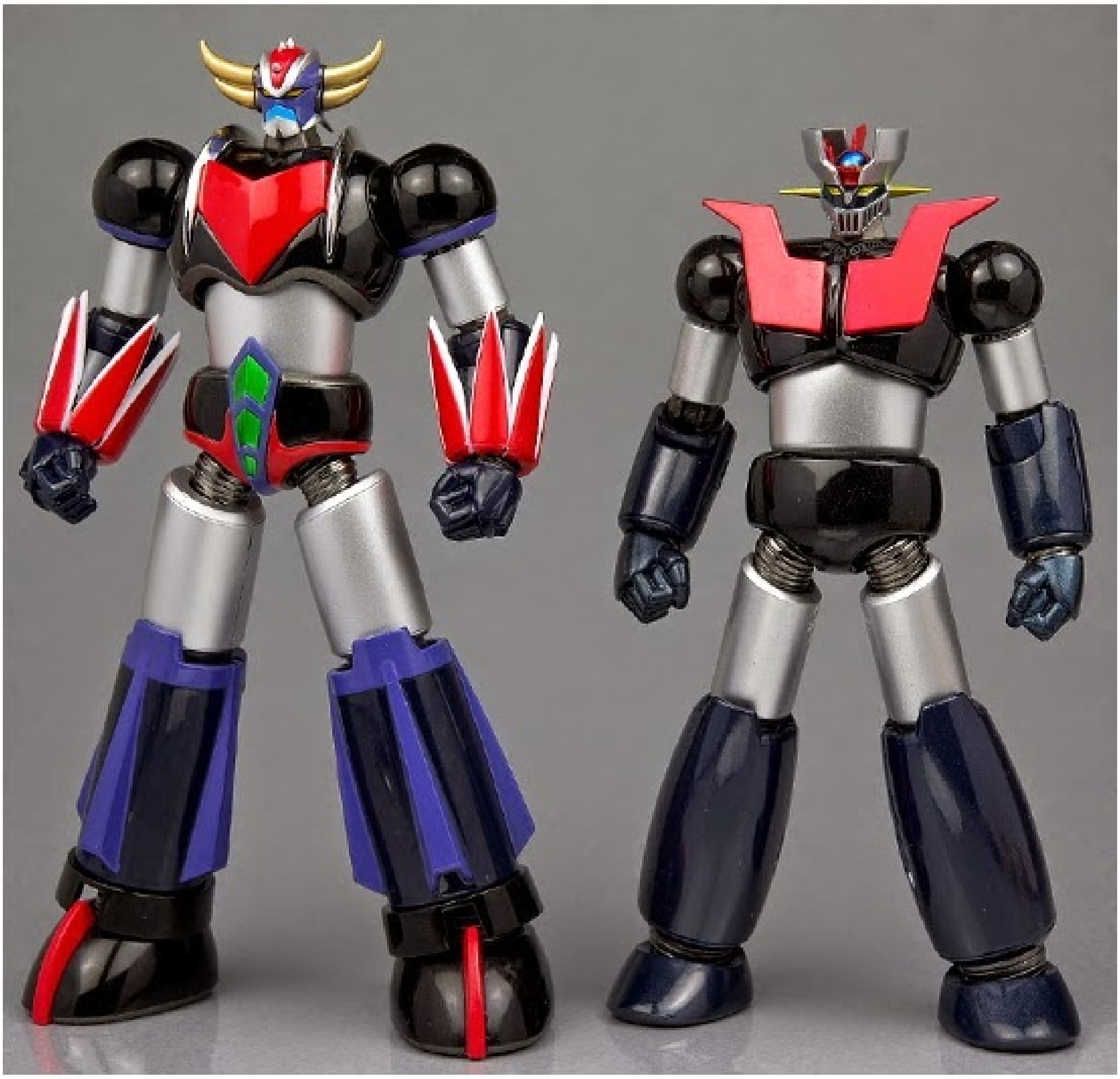

無敵鐵金剛

- 非常不同意
- 不同意
- 有點不同意
- 普通
- 有點同意
- 同意
- 非常同意

36. 對於這張圖片有熟悉感。 *

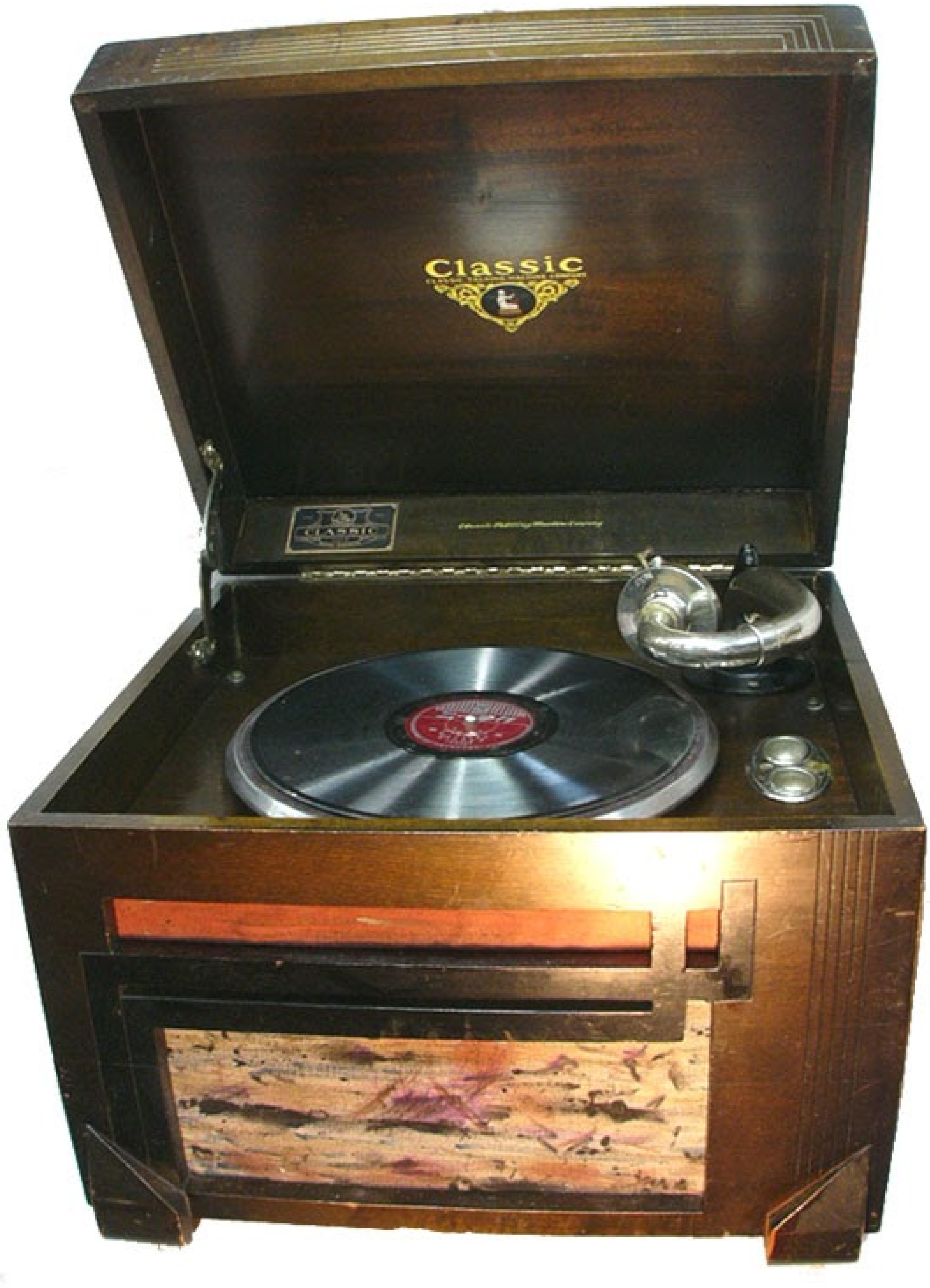

發條留聲機

◯ 非常不同意

◯ 不同意

◯ 有點不同意

◯ 普通

◯ 有點同意

○ 同意

○ 非常同意

37. 對於這張圖片有熟悉感。 *

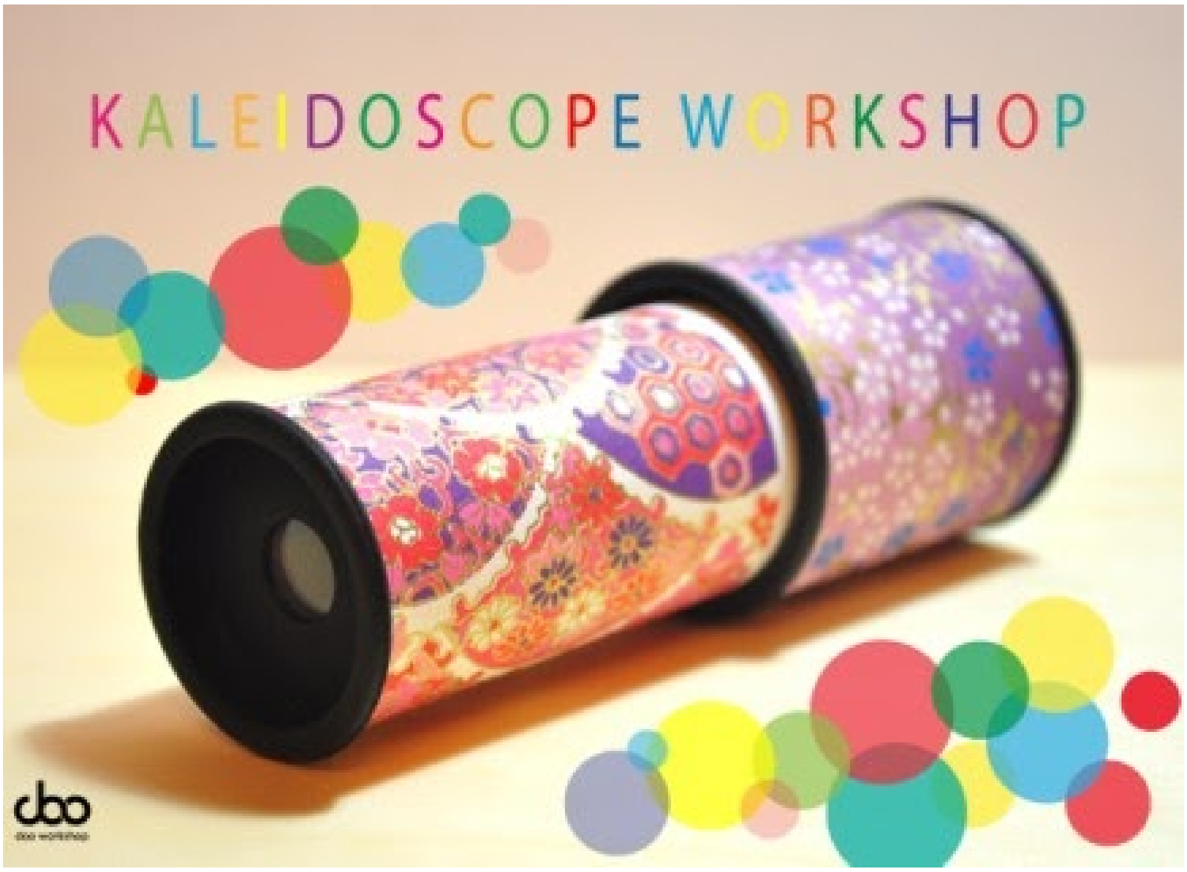

萬花筒

○ 非常不同意

○ 不同意

○ 有點不同意

○ 普通

○ 有點同意

○ 同意

○ 非常同意

38. 對於這張圖片有熟悉感。 *

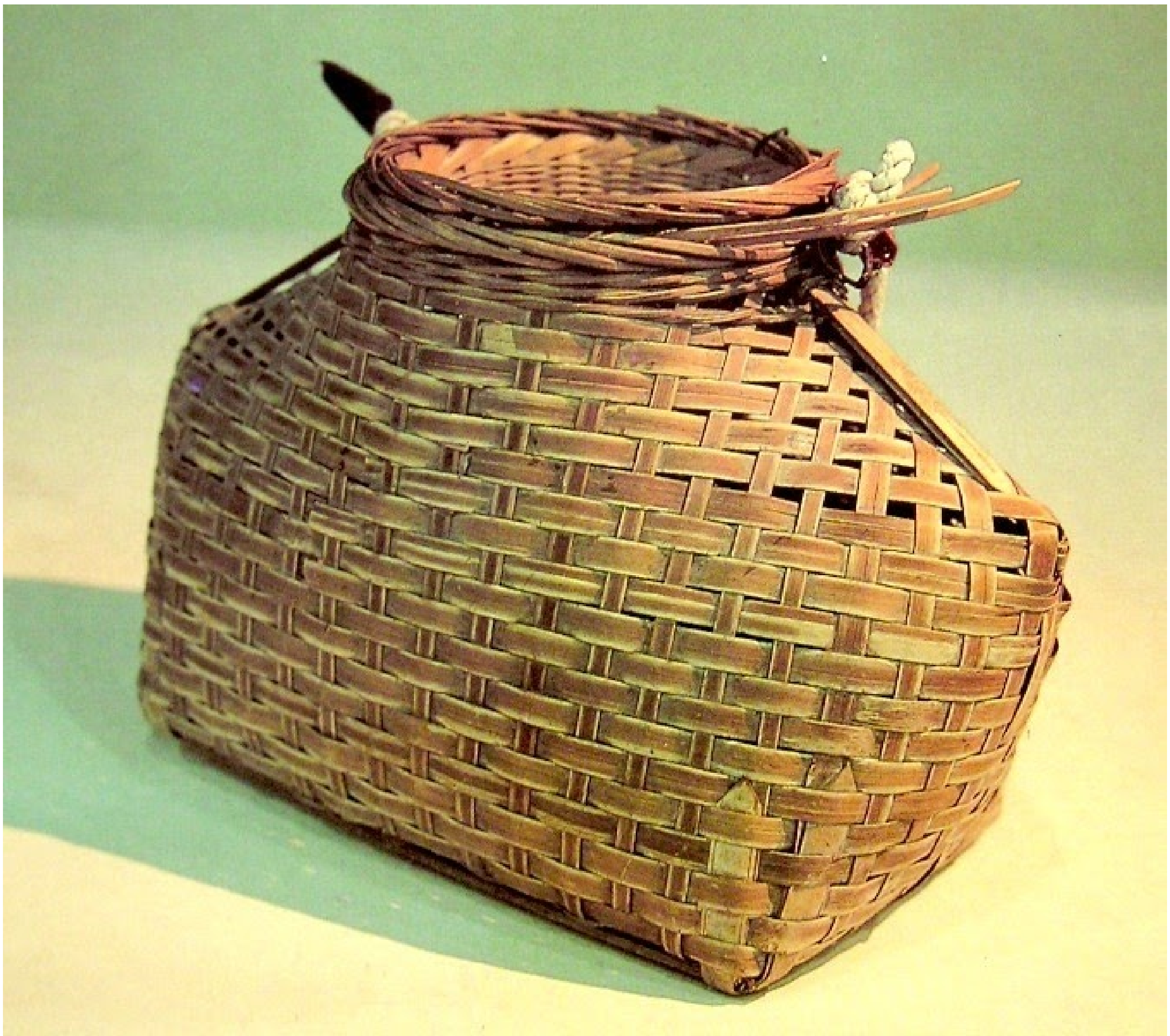

補魚簍

- 非常不同意
- 不同意
- 有點不同意
- 普通
- 有點同意
- 同意
- 非常同意

39. 對於這張圖片有熟悉感。 *

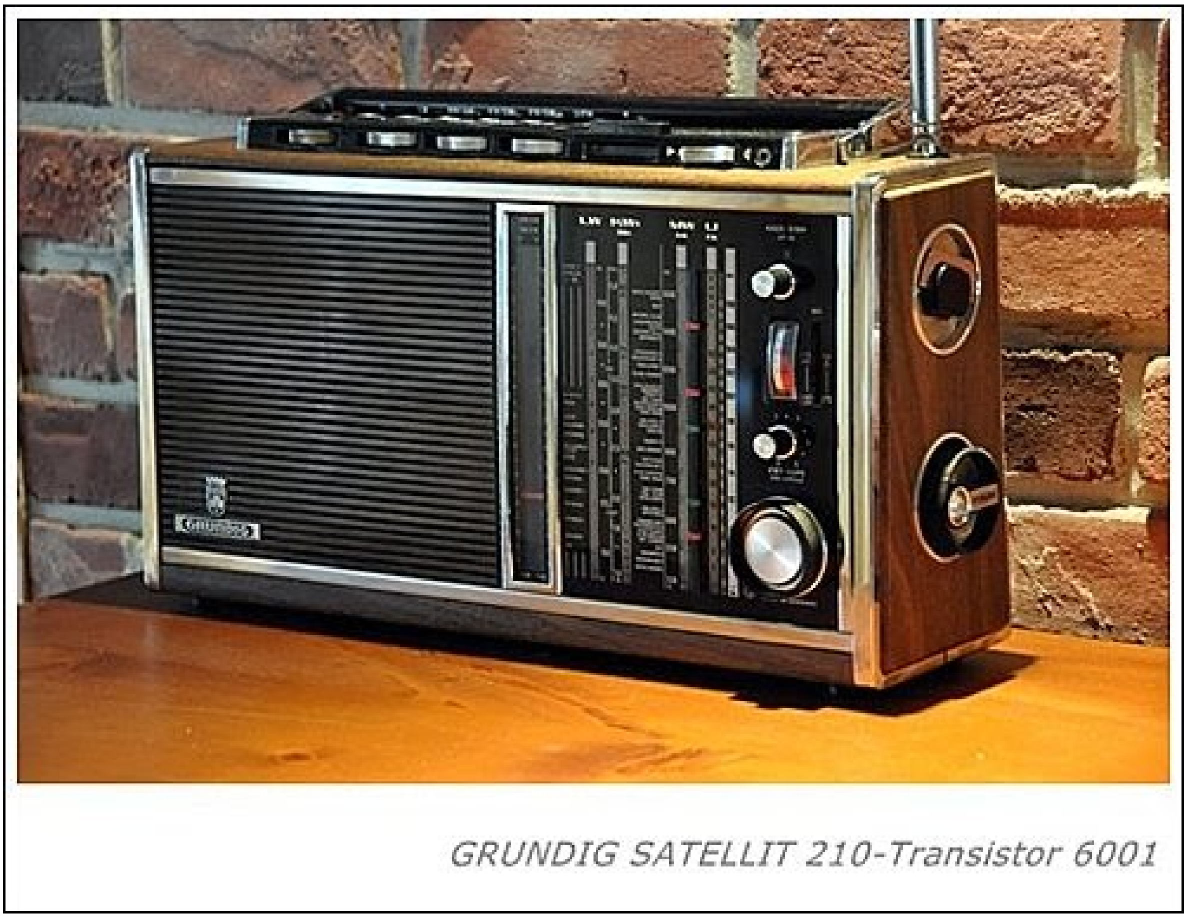

電晶體收音機

- ◯ 非常不同意
- ◯ 不同意
- ◯ 有點不同意
- ◯ 普通
- ◯ 有點同意
- ◯ 同意
- ◯ 非常同意

40. 對於這張圖片有熟悉感。 *

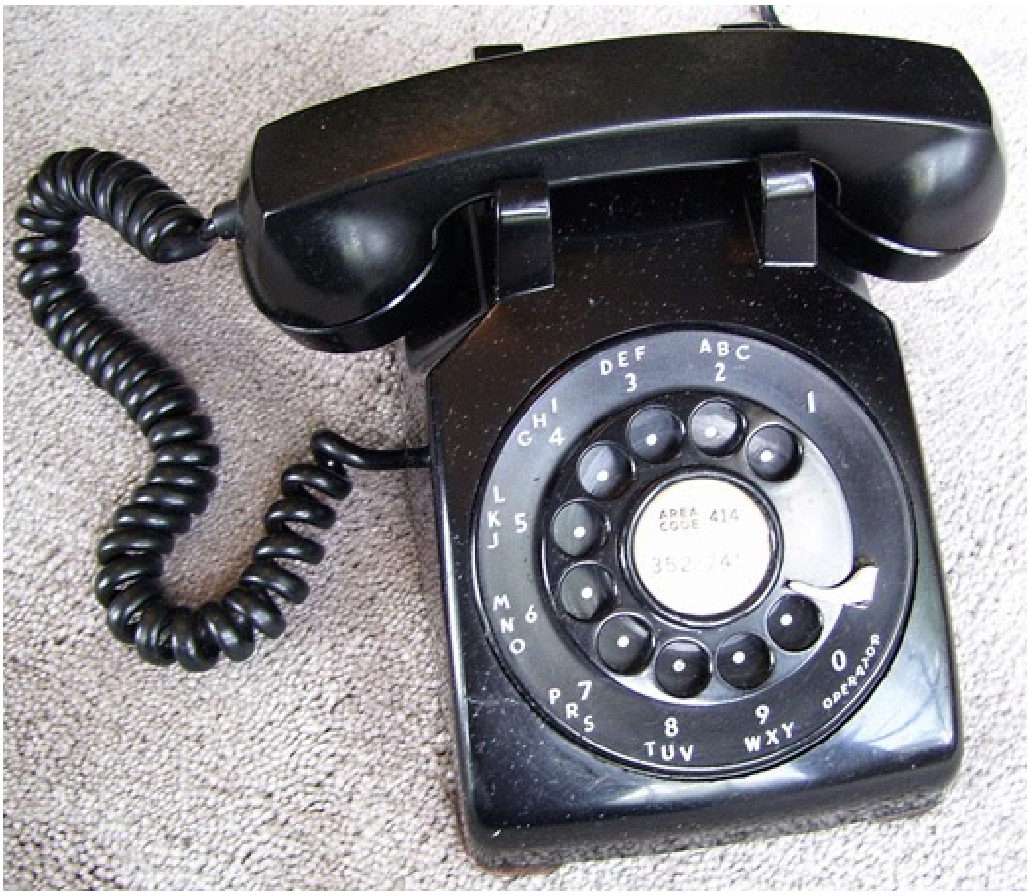

電話

- ◯ 非常不同意
- ◯ 不同意
- ◯ 有點不同意
- ◯ 普通
- ◯ 有點同意
- ◯ 同意
- ◯ 非常同意

41. 對於這張圖片有熟悉感。 *

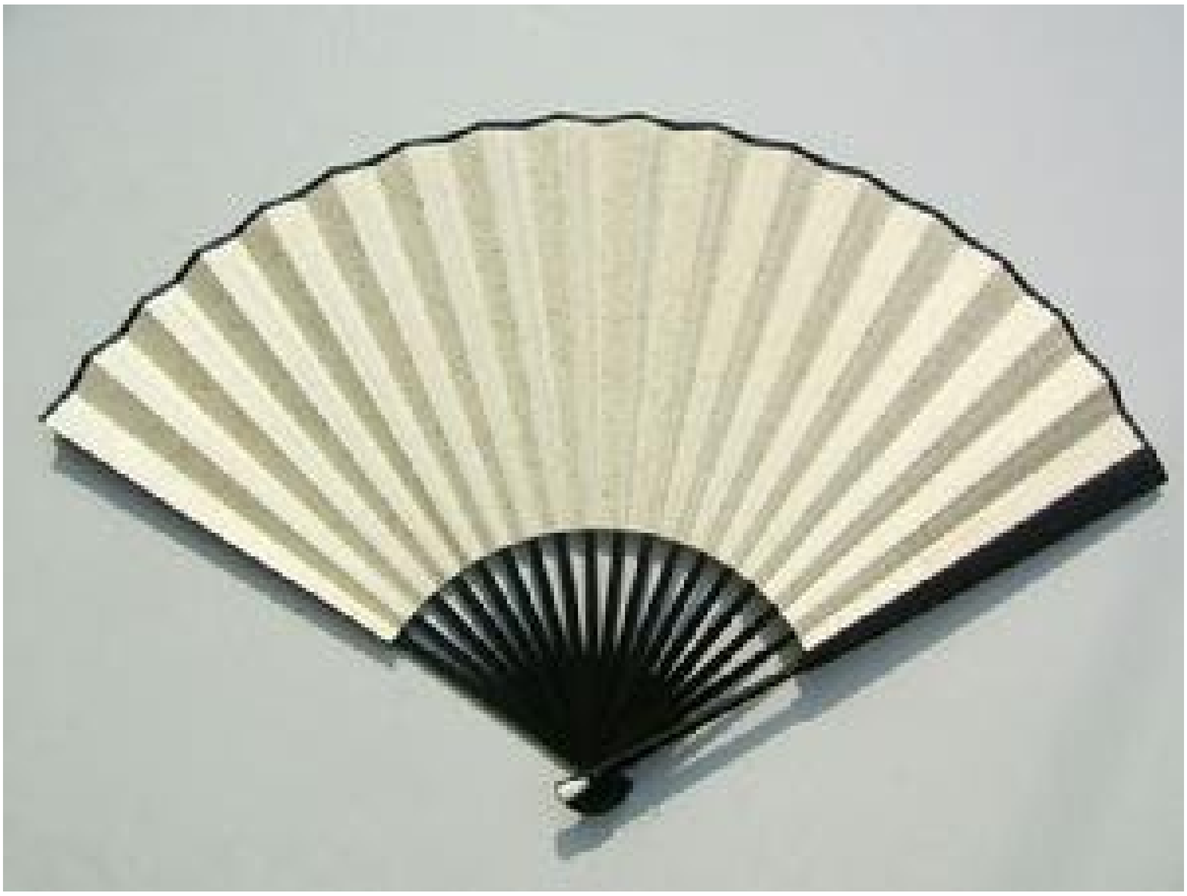

摺扇

- 非常不同意
- 不同意
- 有點不同意
- 普通
- 有點同意
- 同意
- 非常同意

42. 對於這張圖片有熟悉感。 *

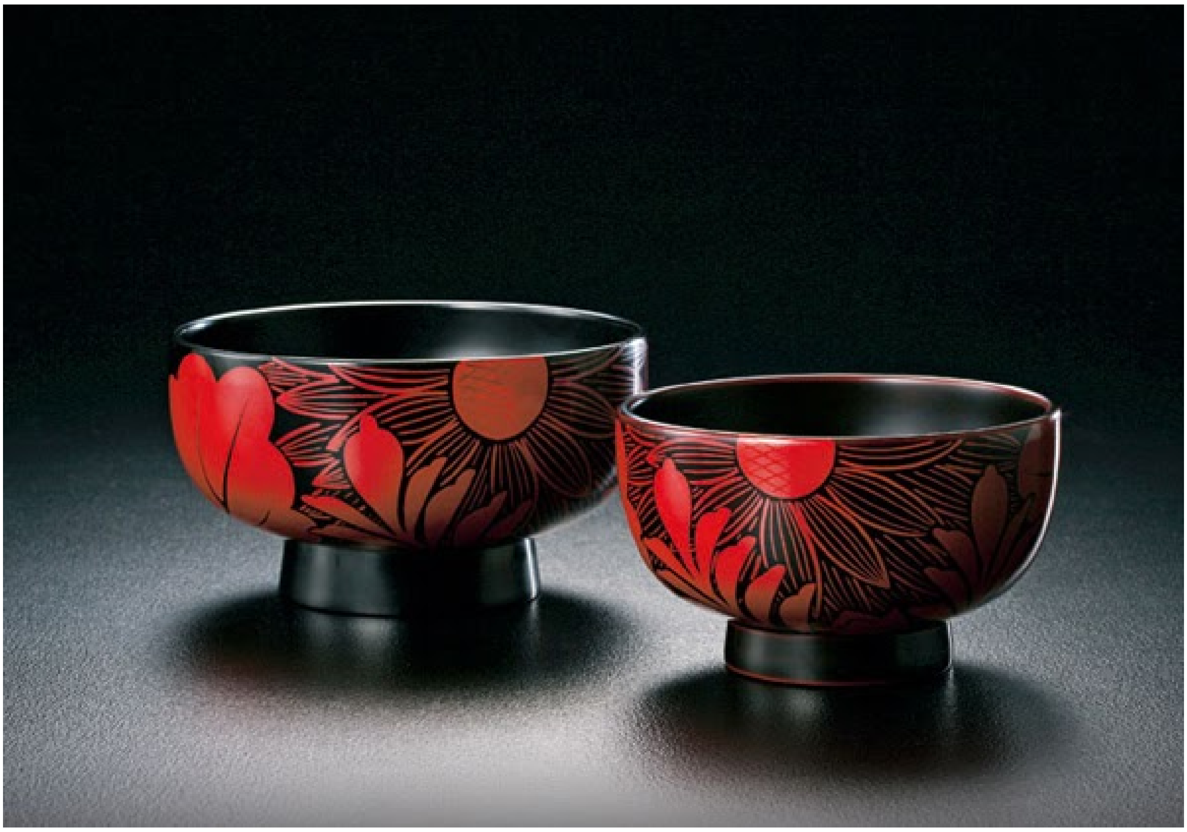

漆器

- 非常不同意
- 不同意
- 有點不同意
- 普通
- 有點同意
- 同意
- 非常同意

43. 對於這張圖片有熟悉感。 *

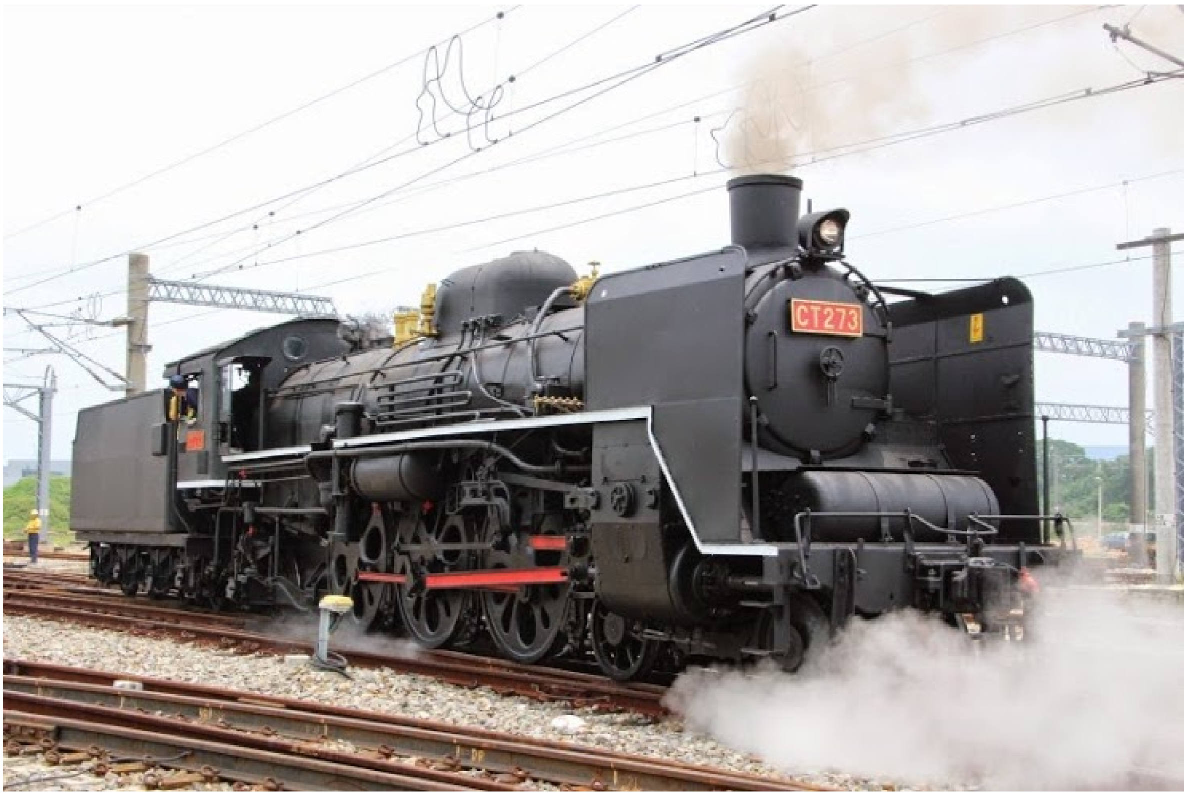

蒸汽火車

- ◯ 非常不同意
- ◯ 不同意
- ◯ 有點不同意
- ◯ 普通
- ◯ 有點同意
- ◯ 同意
- ◯ 非常同意

44. 對於這張圖片有熟悉感。 *

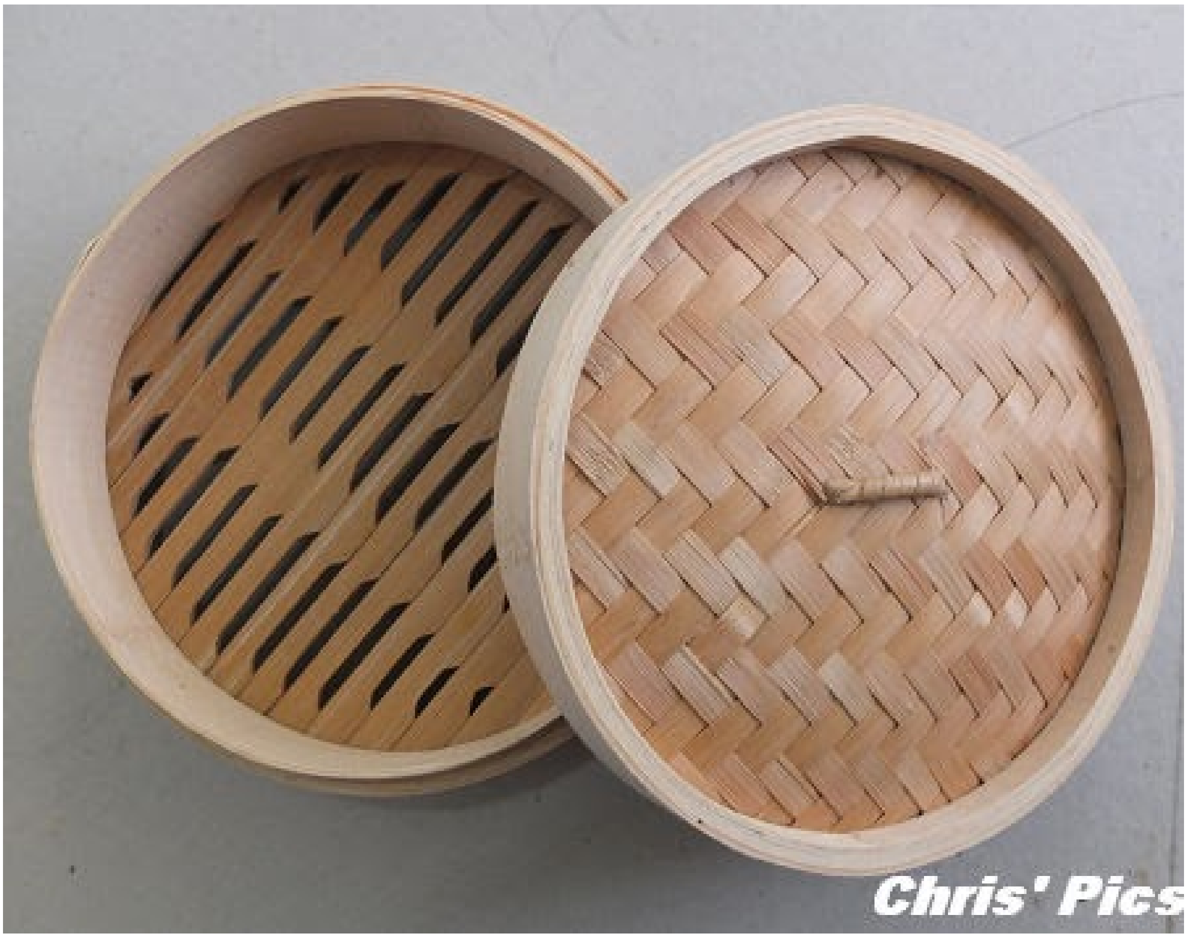

蒸籠

- 非常不同意
- 不同意
- 有點不同意
- 普通
- 有點同意
- 同意
- 非常同意

45. 對於這張圖片有熟悉感。 *

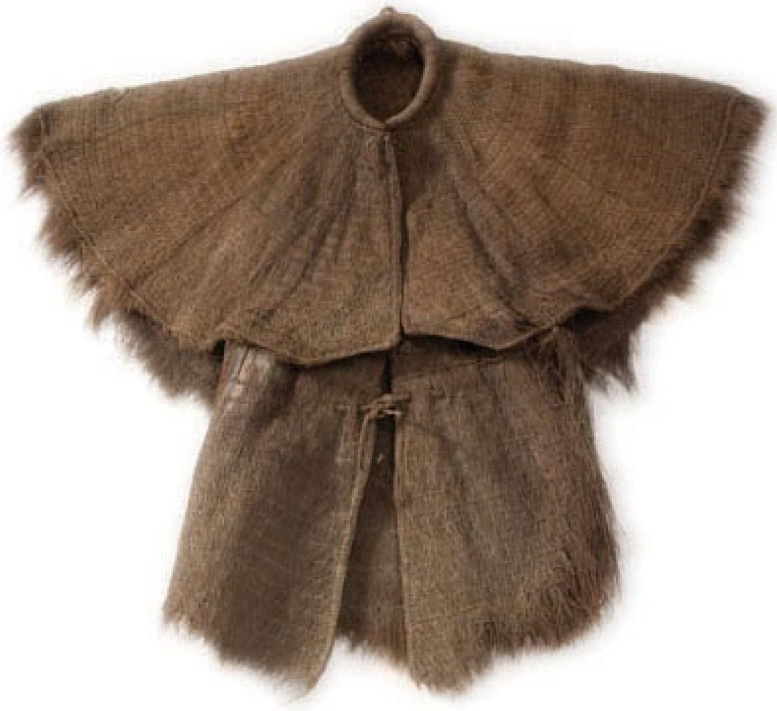

蓑衣

- 非常不同意
- 不同意
- 有點不同意
- 普通
- 有點同意
- 同意
- 非常同意

46. 對於這張圖片有熟悉感。 *

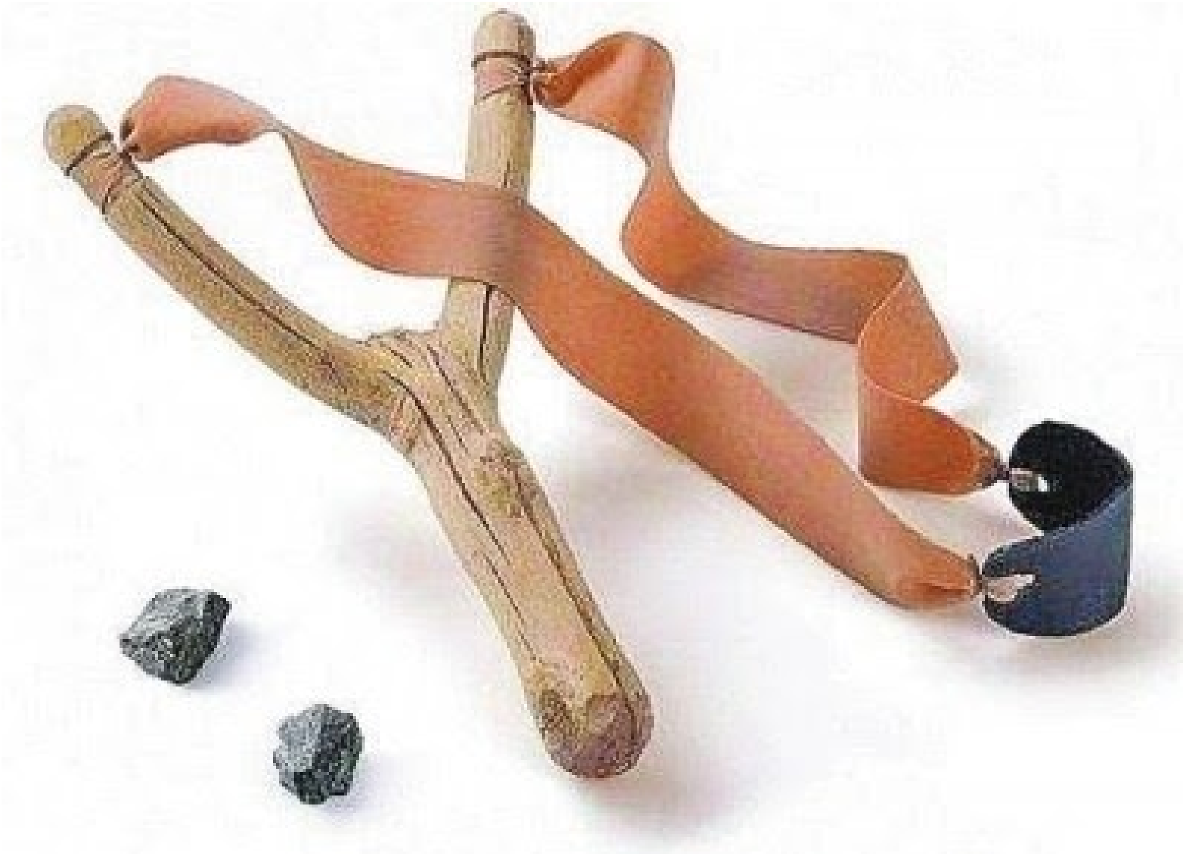

彈弓

- 非常不同意
- 不同意
- 有點不同意
- 普通
- 有點同意
- 同意
- 非常同意

47. 對於這張圖片有熟悉感。 *

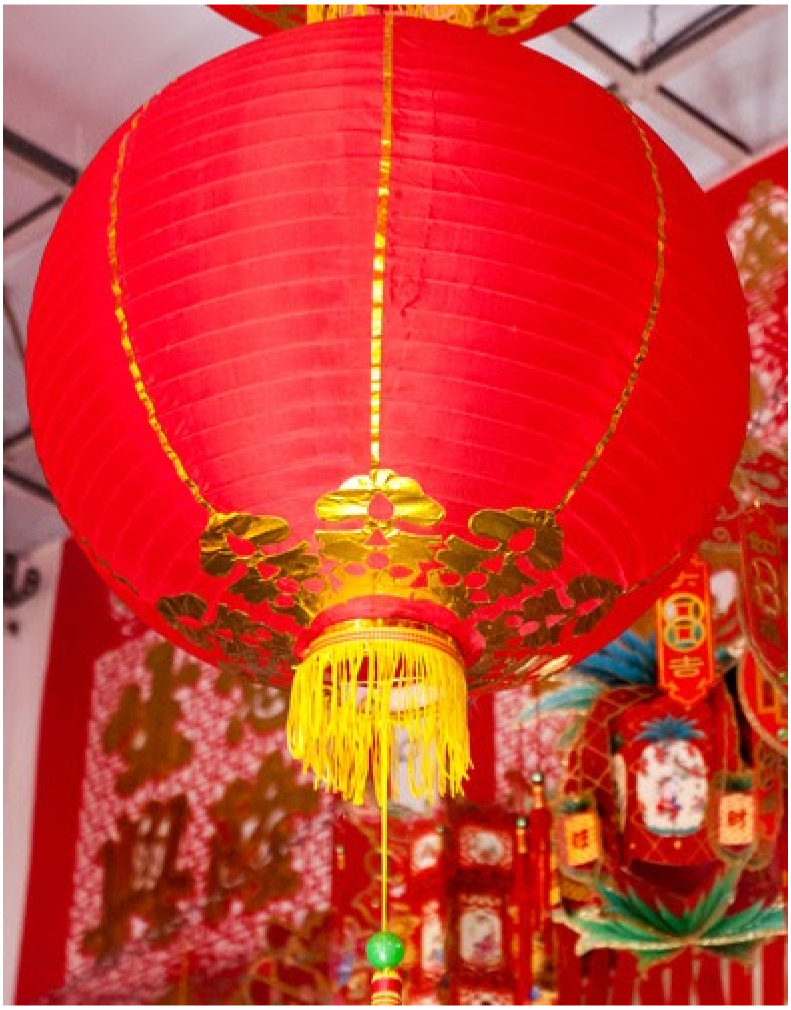

燈籠

- ◯ 非常不同意
- ◯ 不同意
- ◯ 有點不同意
- ◯ 普通
- ◯ 有點同意
- ◯ 同意
- ◯ 非常同意

48. 對於這張圖片有熟悉感。 *

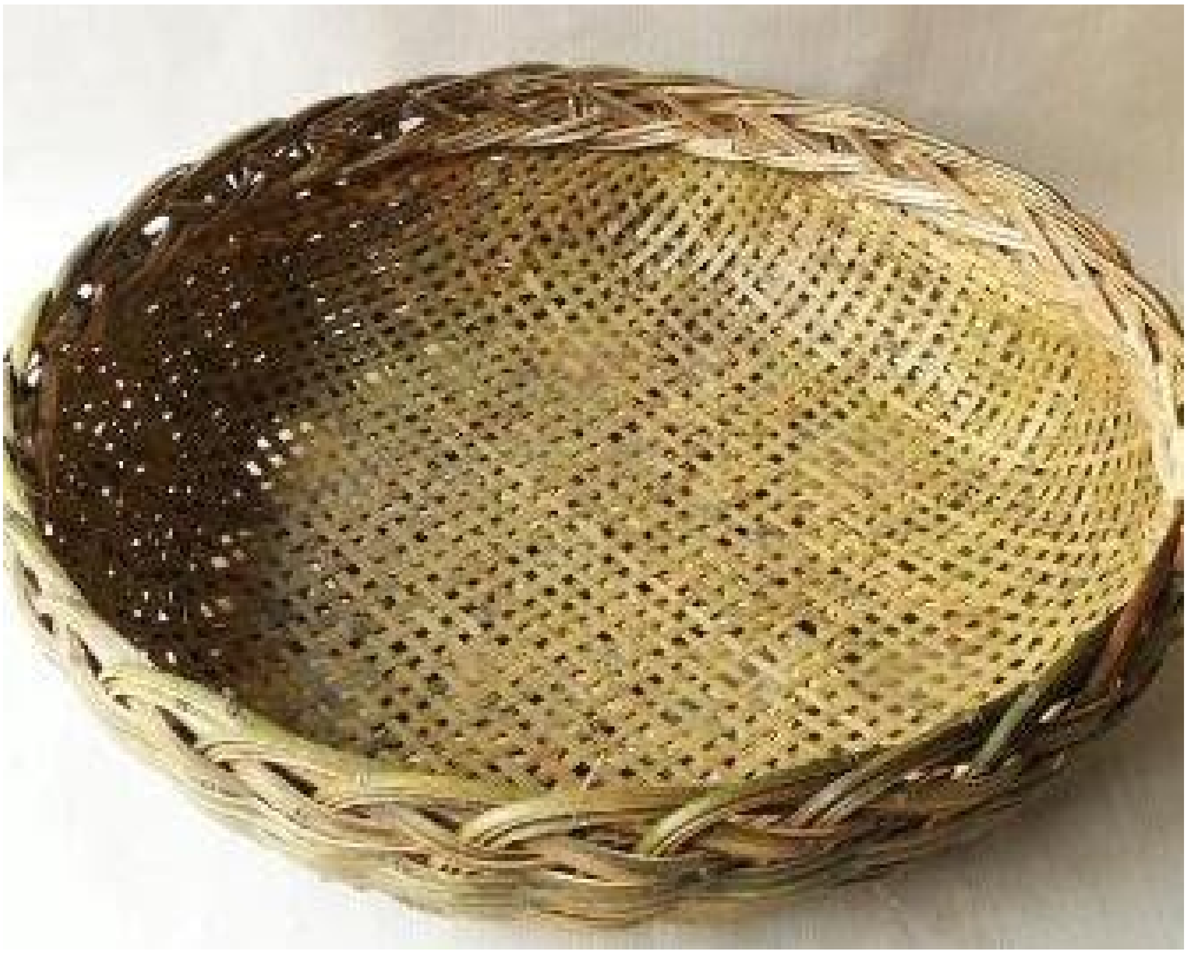

篩子

- 非常不同意
- 不同意
- 有點不同意
- 普通
- 有點同意
- 同意
- 非常同意

49. 對於這張圖片有熟悉感。 *

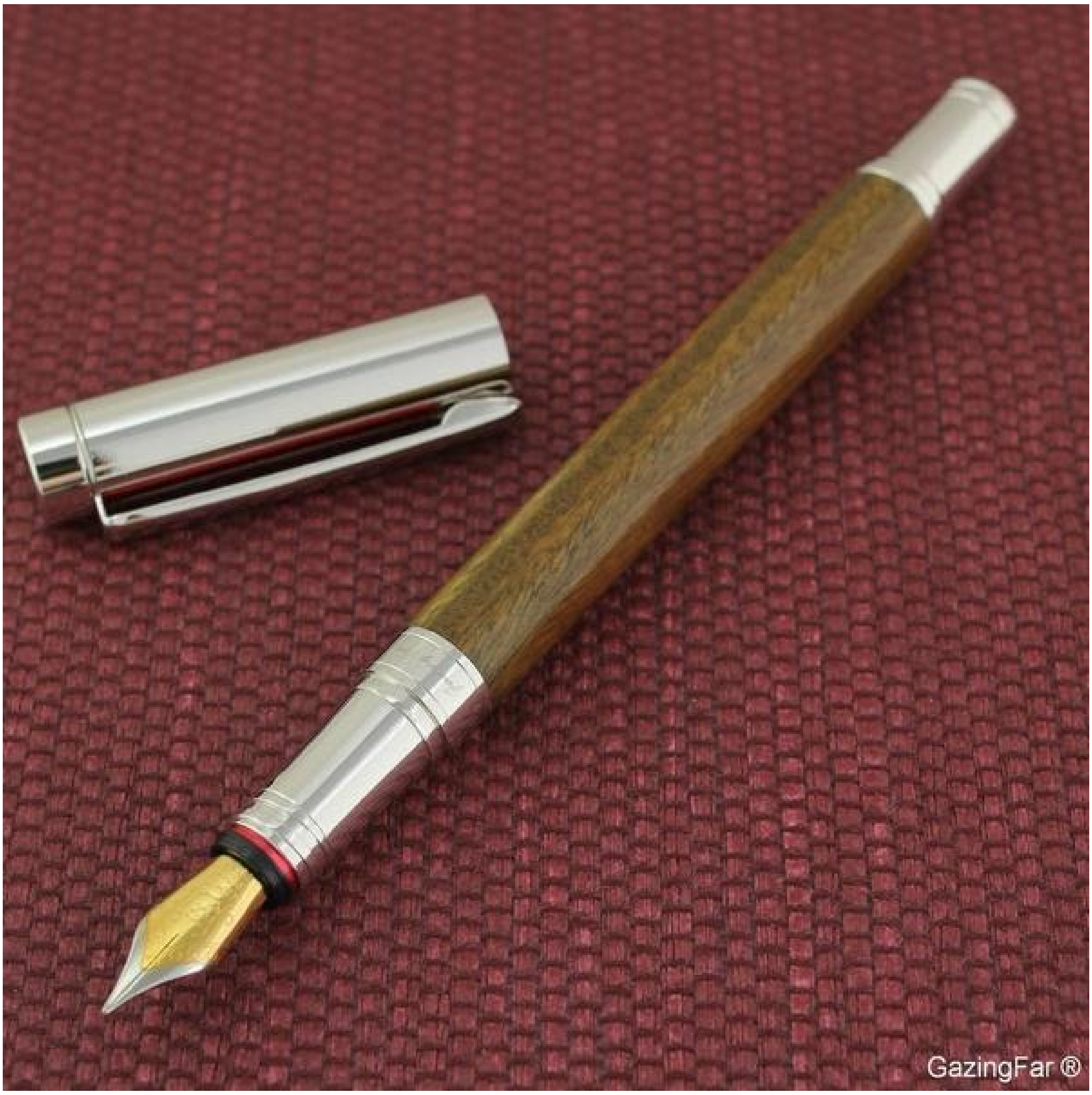

鋼筆

- 非常不同意
- 不同意
- 有點不同意
- 普通
- 有點同意
- 同意
- 非常同意

50. 對於這張圖片有熟悉感。 *

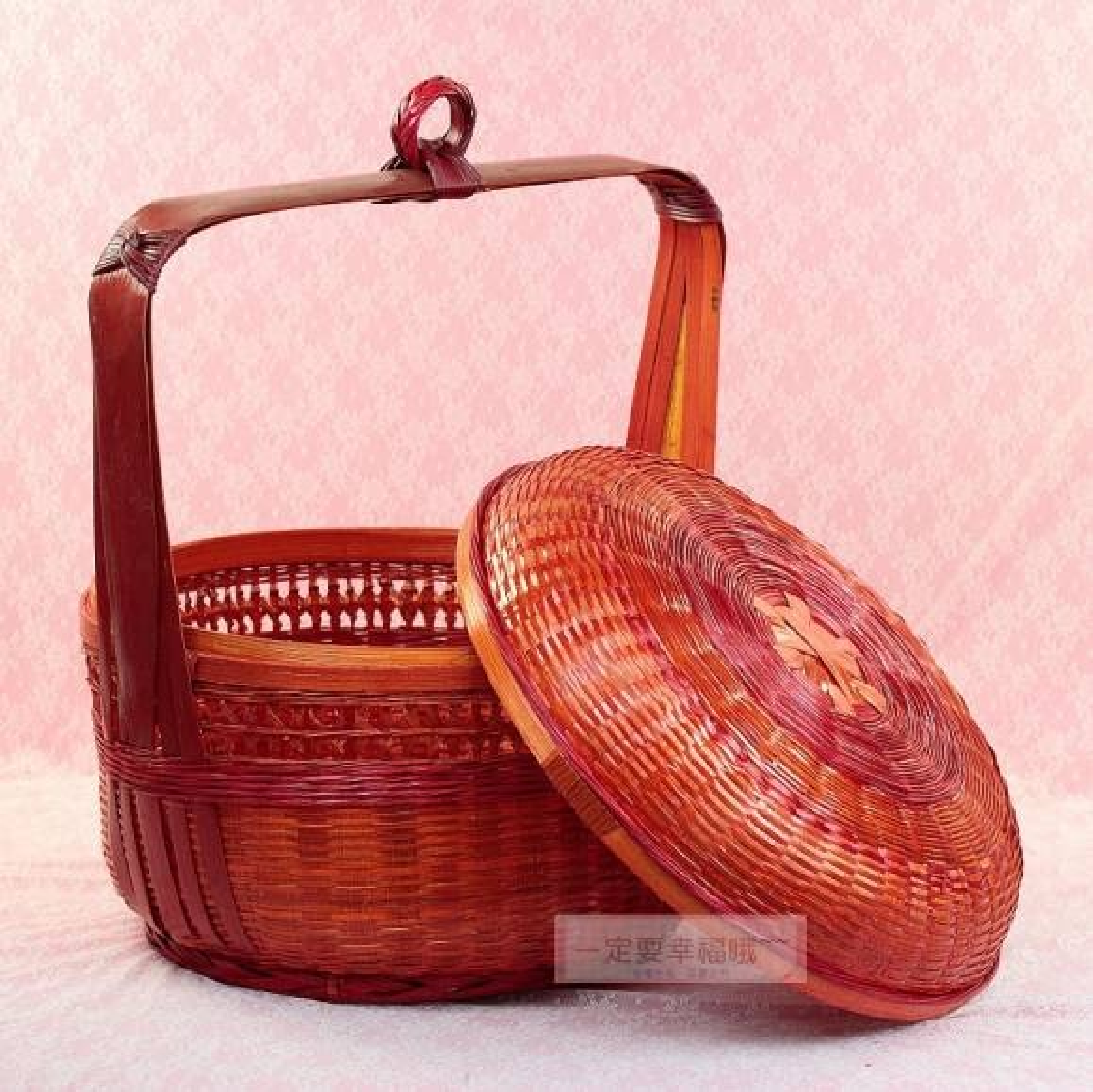

謝籃

- 非常不同意
- 不同意
- 有點不同意
- 普通
- 有點同意
- 同意
- 非常同意

51. 對於這張圖片有熟悉感。 *

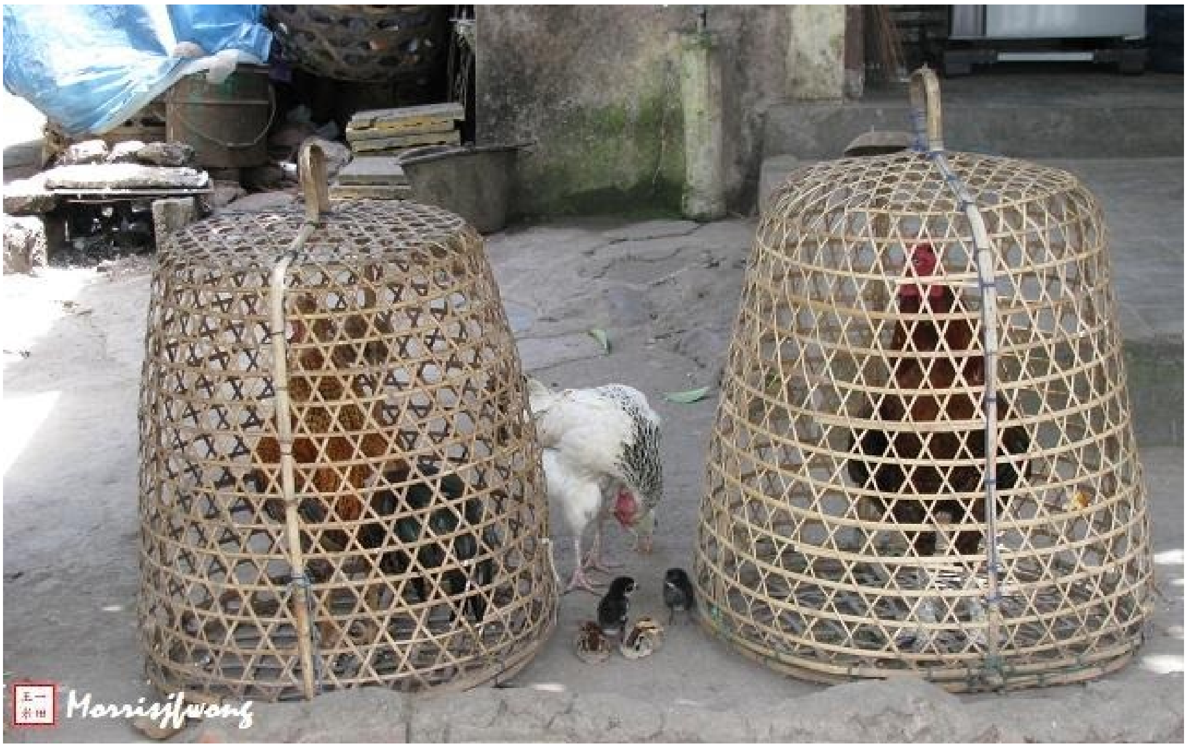

雞籠

- 非常不同意
- 不同意
- 有點不同意
- 普通
- 有點同意
- 同意
- 非常同意

52. 對於這張圖片有熟悉感。 *

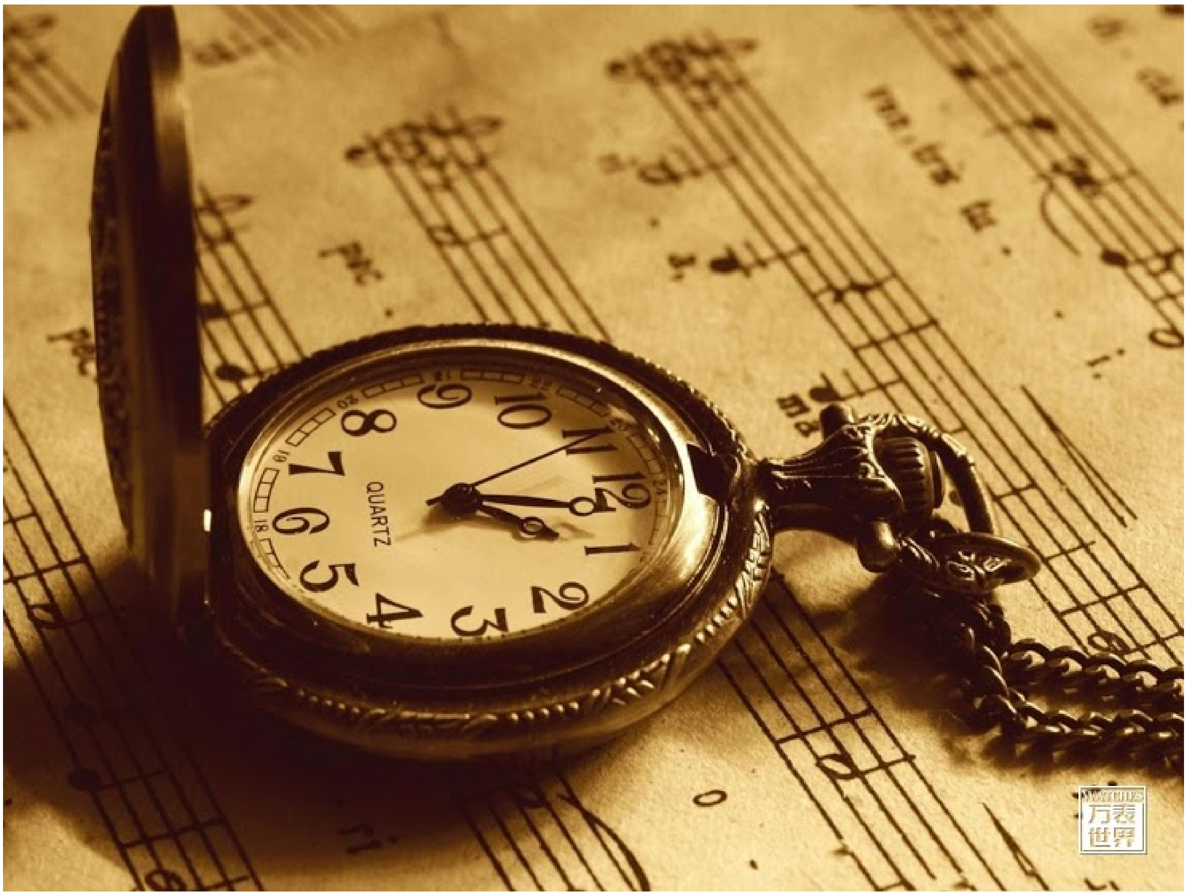

懷錶

- ◯ 非常不同意
- ◯ 不同意
- ◯ 有點不同意
- ◯ 普通
- ◯ 有點同意
- ◯ 同意
- ◯ 非常同意

53. 對於這張圖片有熟悉感。 *

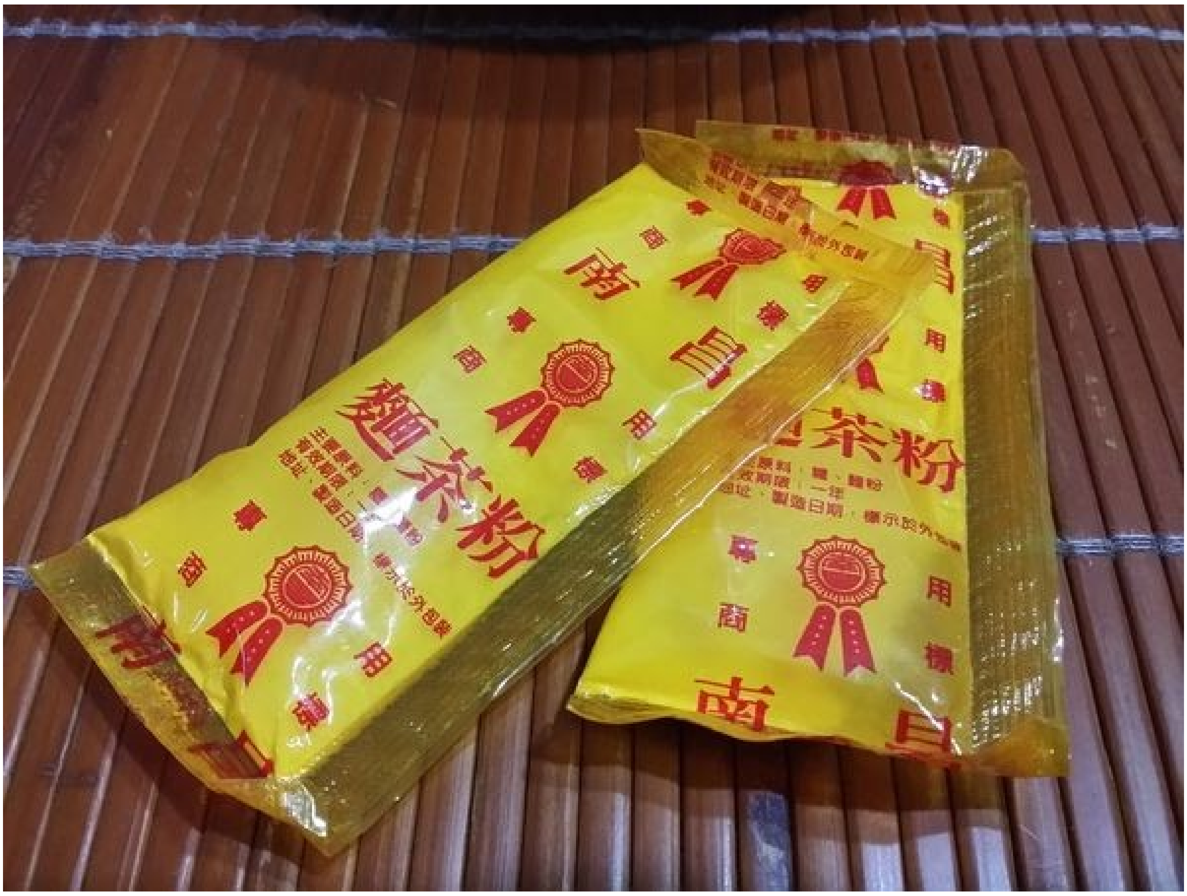

麵茶粉

- ◯ 非常不同意
- ◯ 不同意
- ◯ 有點不同意
- ◯ 普通
- ◯ 有點同意
- ◯ 同意
- ◯ 非常同意

54. 對於這張圖片有熟悉感。 *

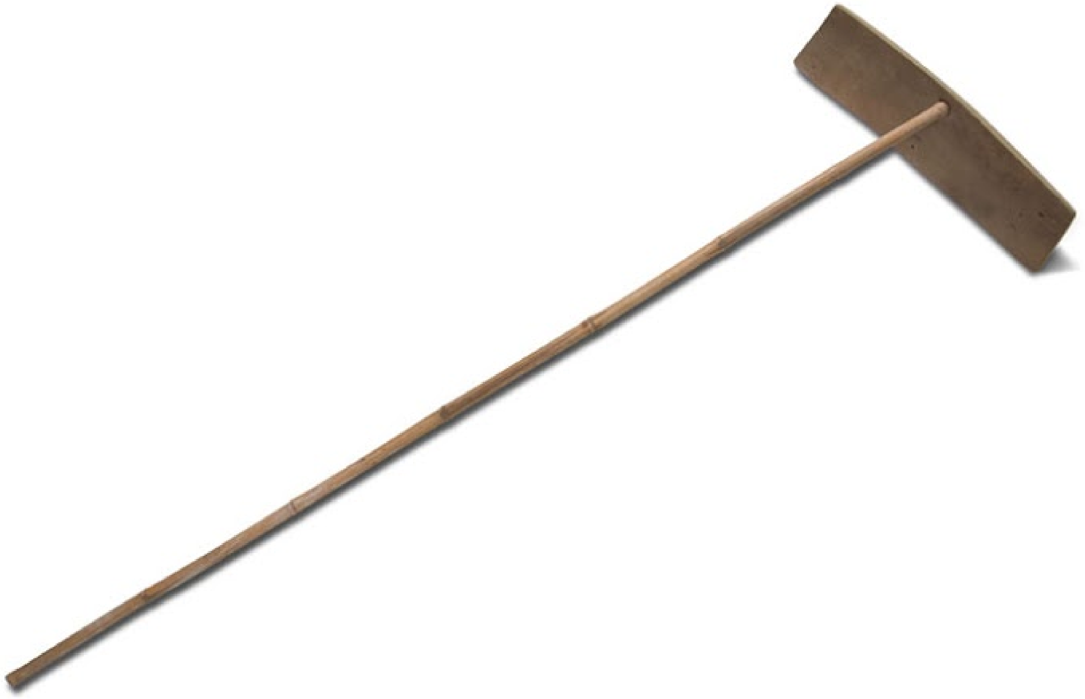

曬穀耙

- 非常不同意
- 不同意
- 有點不同意
- 普通
- 有點同意
- 同意
- 非常同意

55. 對於這張圖片有熟悉感。 *

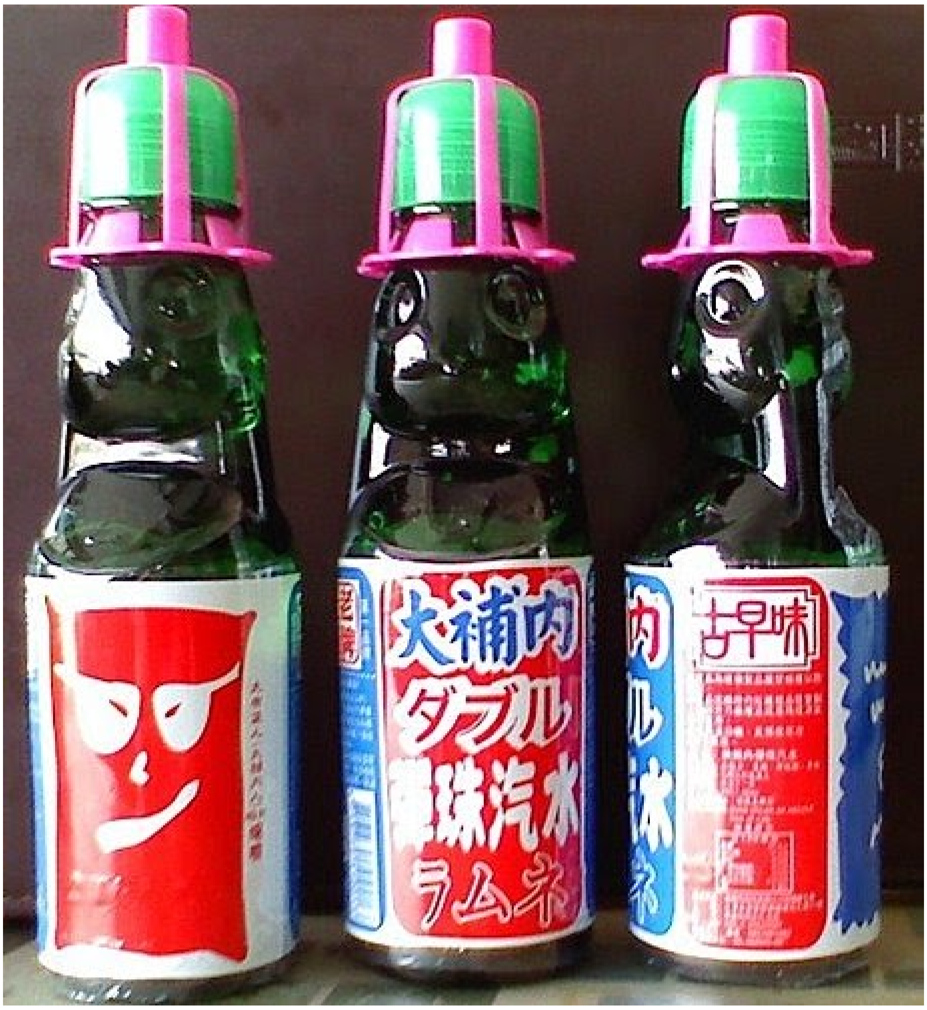

彈珠汽水

- 非常不同意
- 不同意
- 有點不同意
- 普通
- 有點同意
- 同意
- 非常同意

56. 對於這張圖片有熟悉感。 *

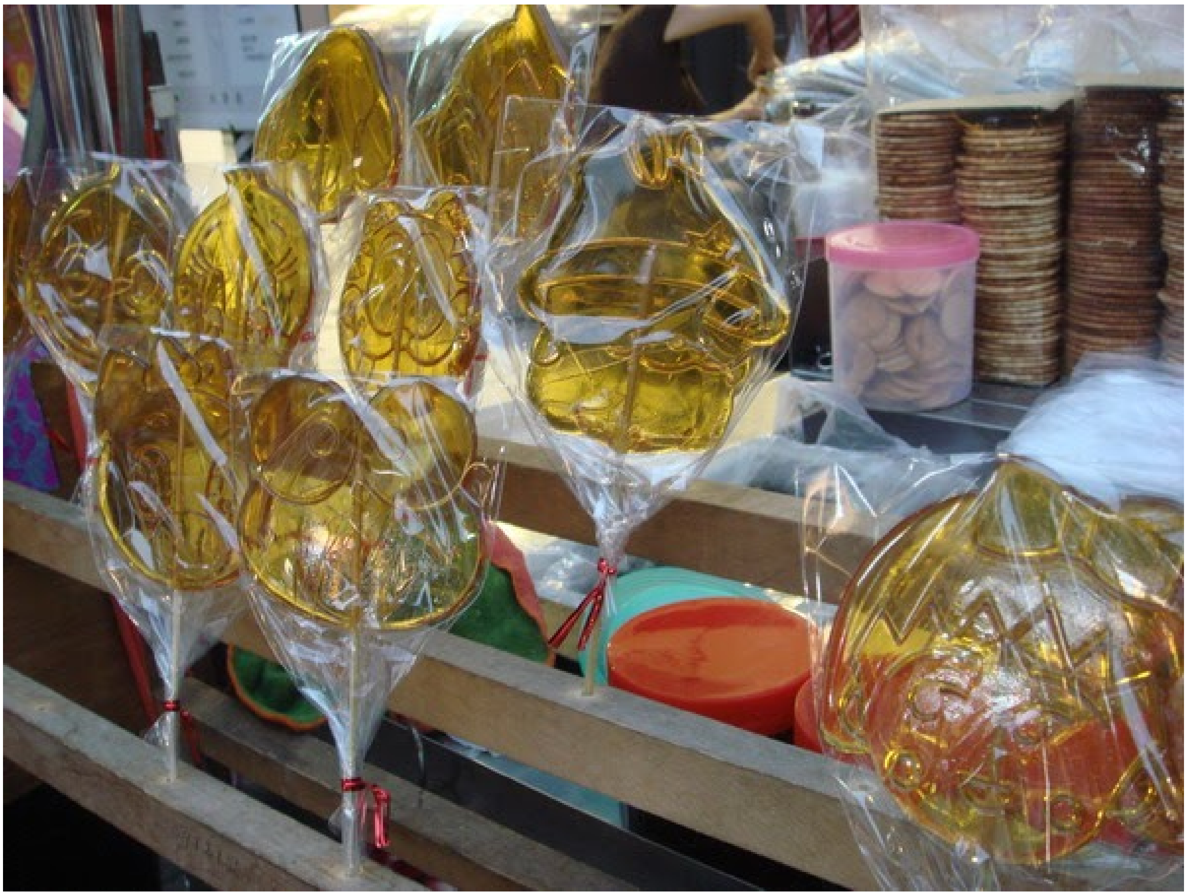

麥芽糖

- ◯ 非常不同意
- ◯ 不同意
- ◯ 有點不同意
- ◯ 普通
- ◯ 有點同意
- ◯ 同意
- ◯ 非常同意

57. 對於這張圖片有熟悉感。 *

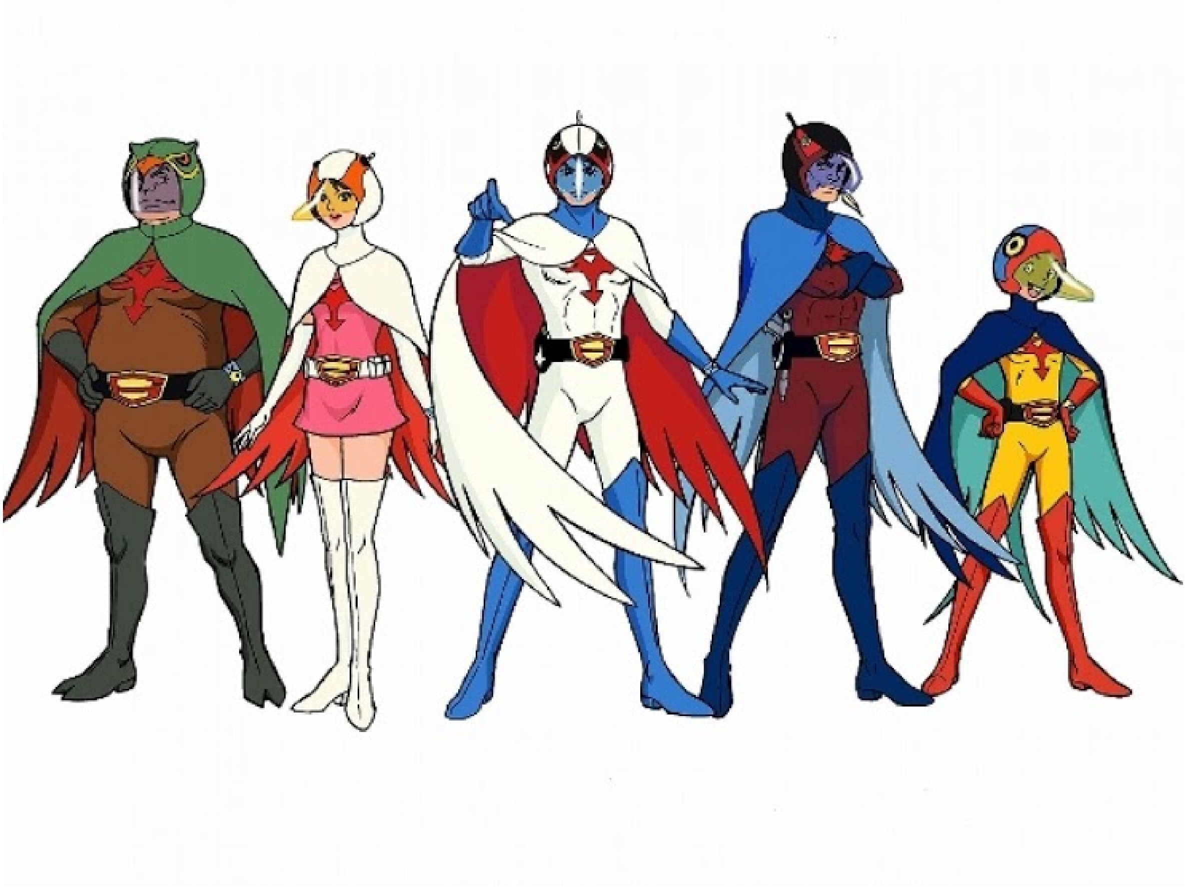

科學小飛俠

- ○ 非常不同意
- ○ 不同意
- ○ 有點不同意
- ○ 普通
- ○ 有點同意
- ○ 同意
- ○ 非常同意

58. 對於這張圖片有熟悉感。 *

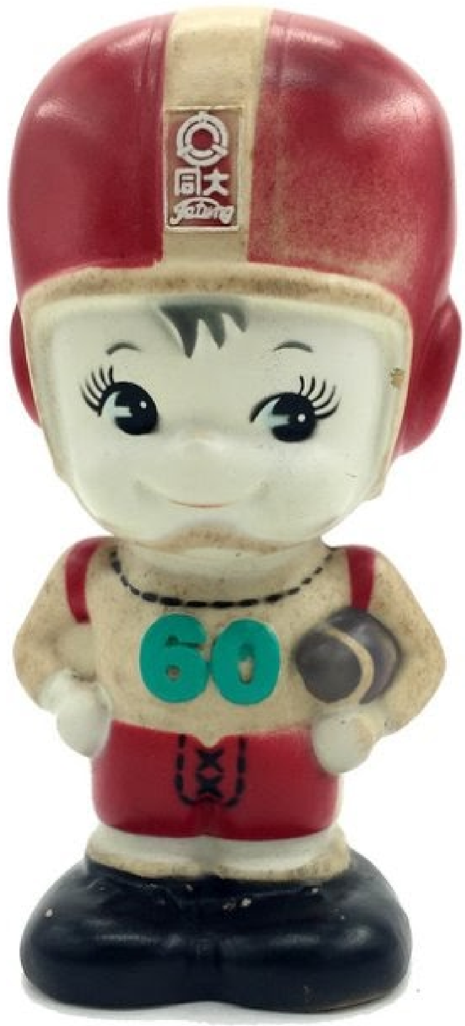

大同寶寶

- ◯ 非常不同意
- ◯ 不同意
- ◯ 有點不同意
- ◯ 普通
- ◯ 有點同意
- ◯ 同意
- ◯ 非常同意

59. 對於這張圖片有熟悉感。 *

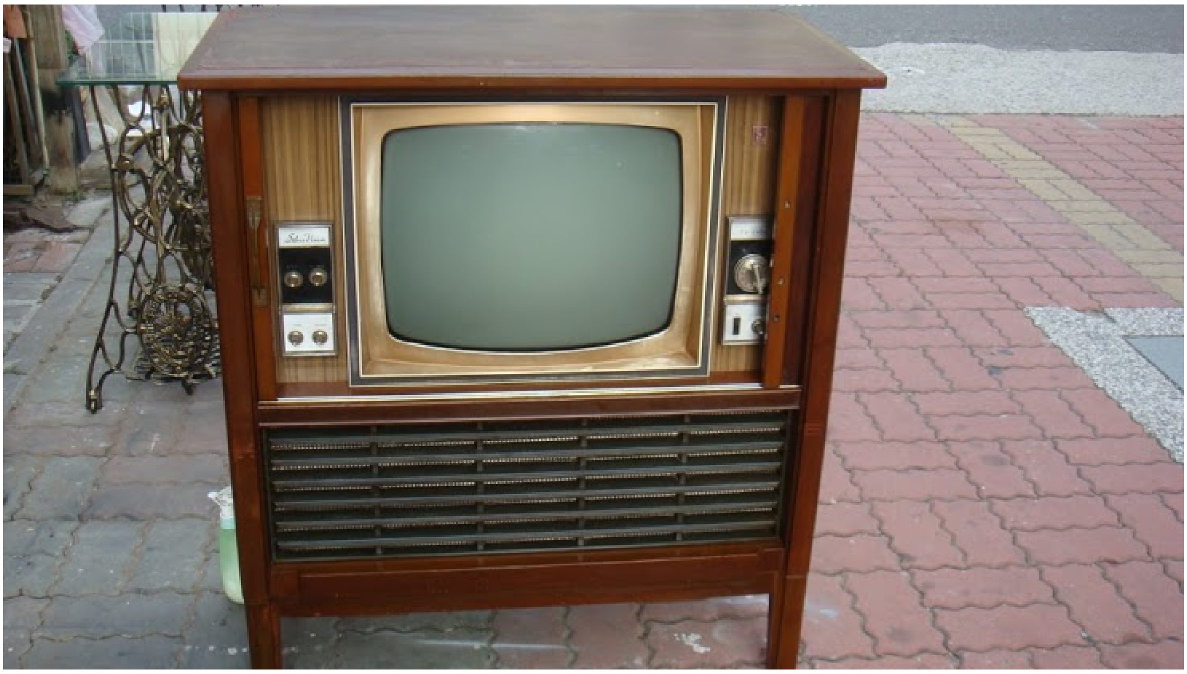

電視

- 非常不同意
- 不同意
- 有點不同意
- 普通
- 有點同意
- 同意
- 非常同意

60. 對於這張圖片有熟悉感。 *

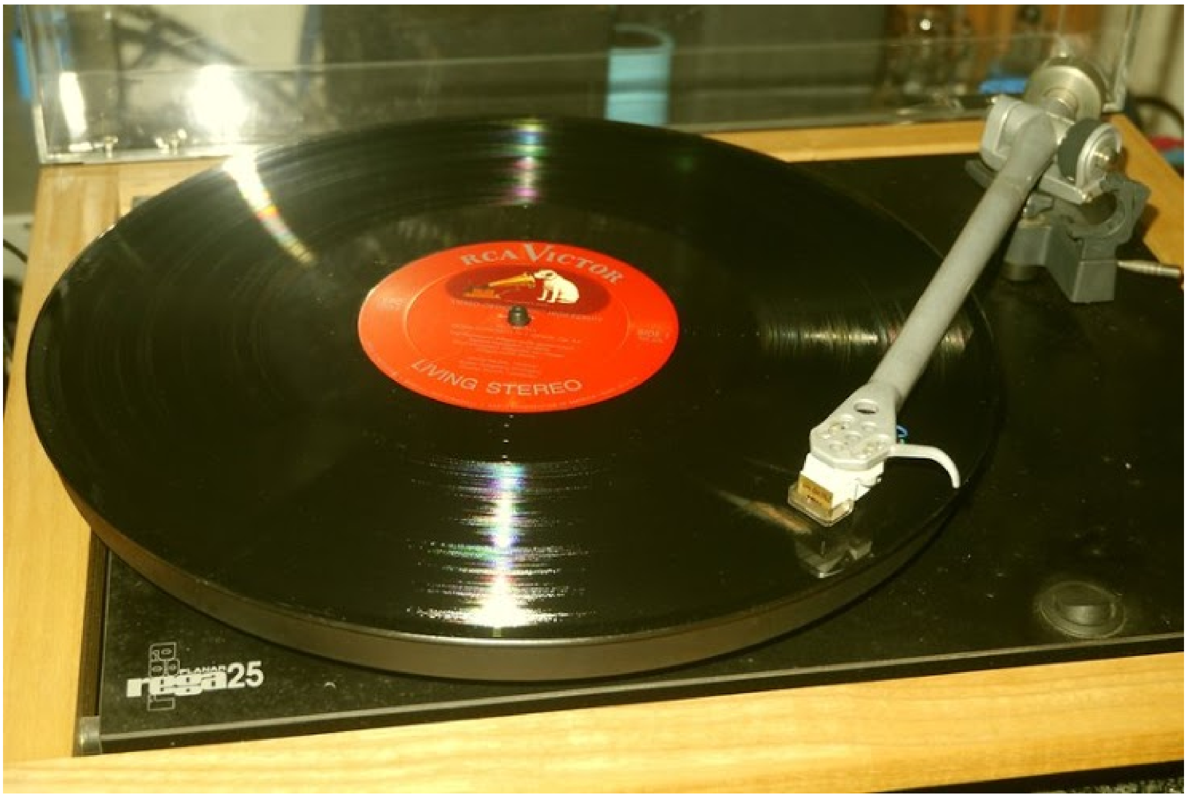

黑膠唱片

- ◯ 非常不同意
- ◯ 不同意
- ◯ 有點不同意
- ◯ 普通
- ◯ 有點同意
- ◯ 同意
- ◯ 非常同意

61. 對於這張圖片有熟悉感。 *

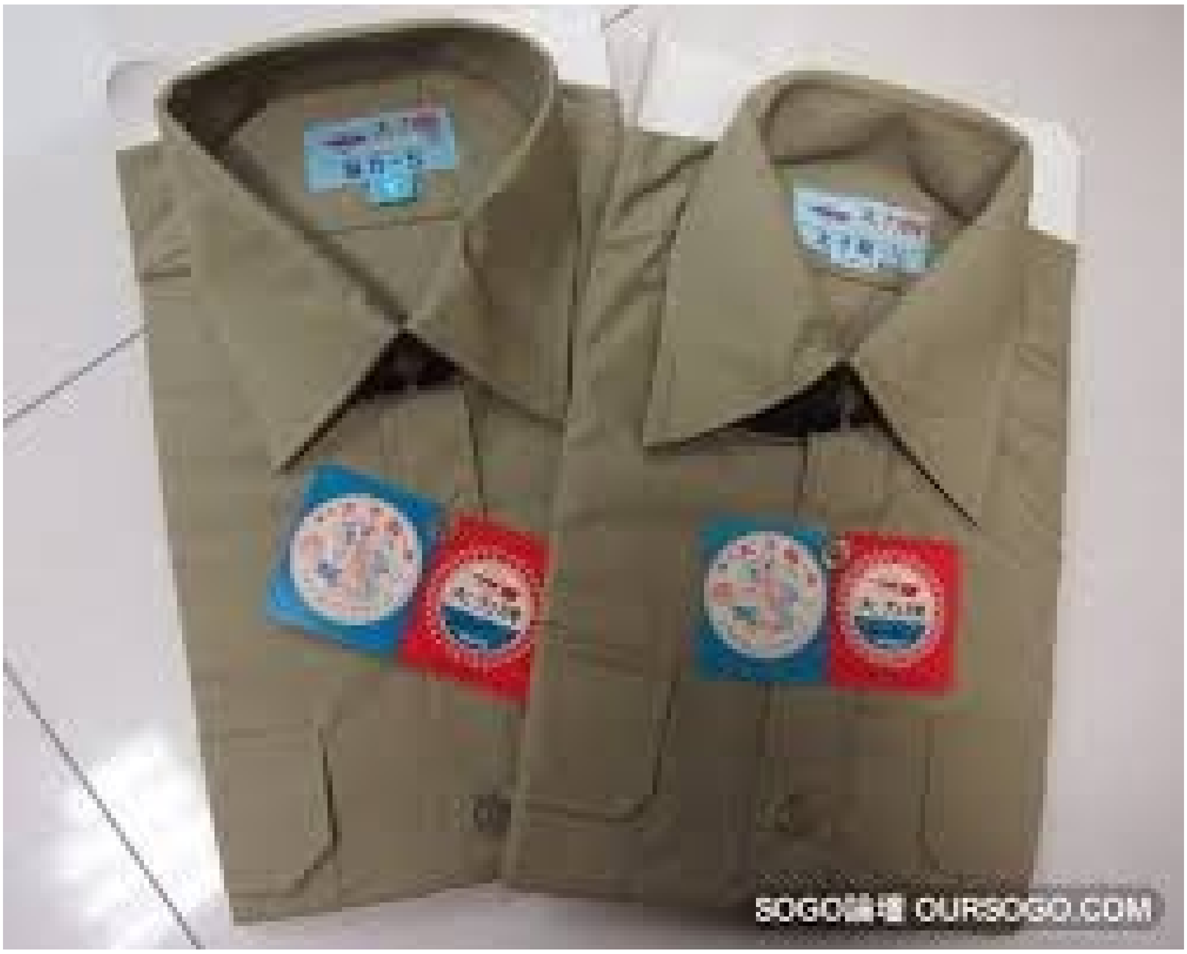

太子龍學生服

- 非常不同意
- 不同意
- 有點不同意
- 普通
- 有點同意
- 同意
- 非常同意

62. 對於這張圖片有熟悉感。 *

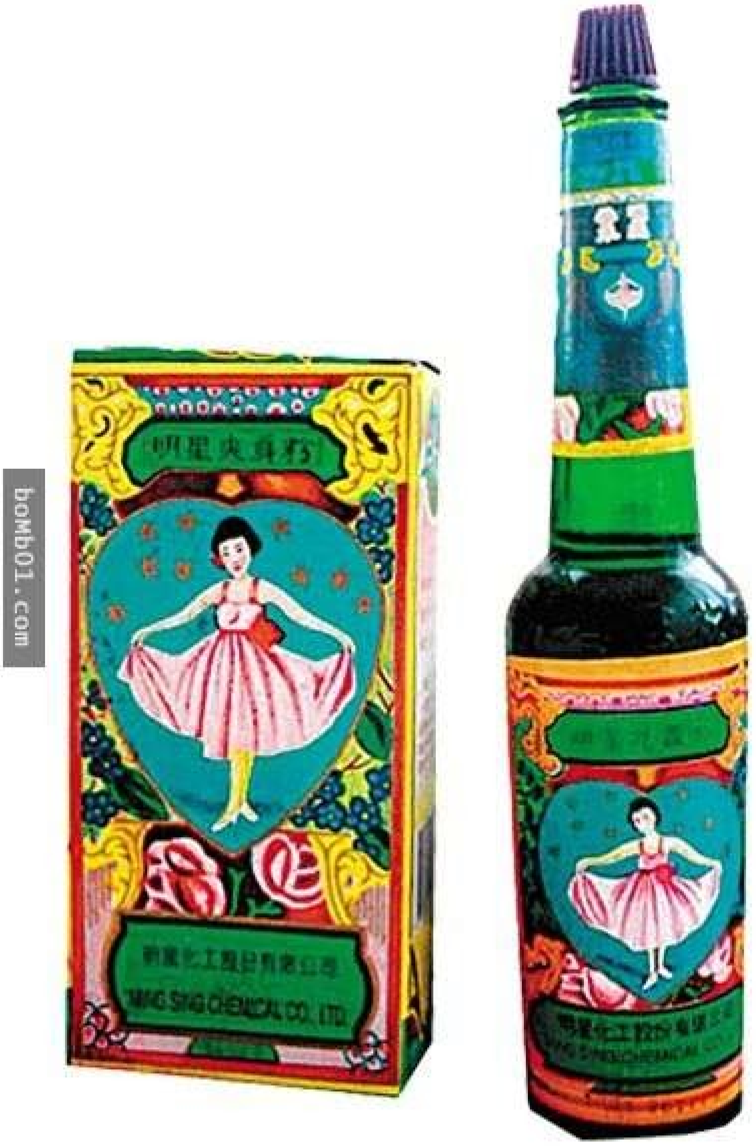

明星花露水

- 非常不同意
- 不同意
- 有點不同意
- 普通
- 有點同意
- 同意
- 非常同意

63. 對於這張圖片有熟悉感。 *

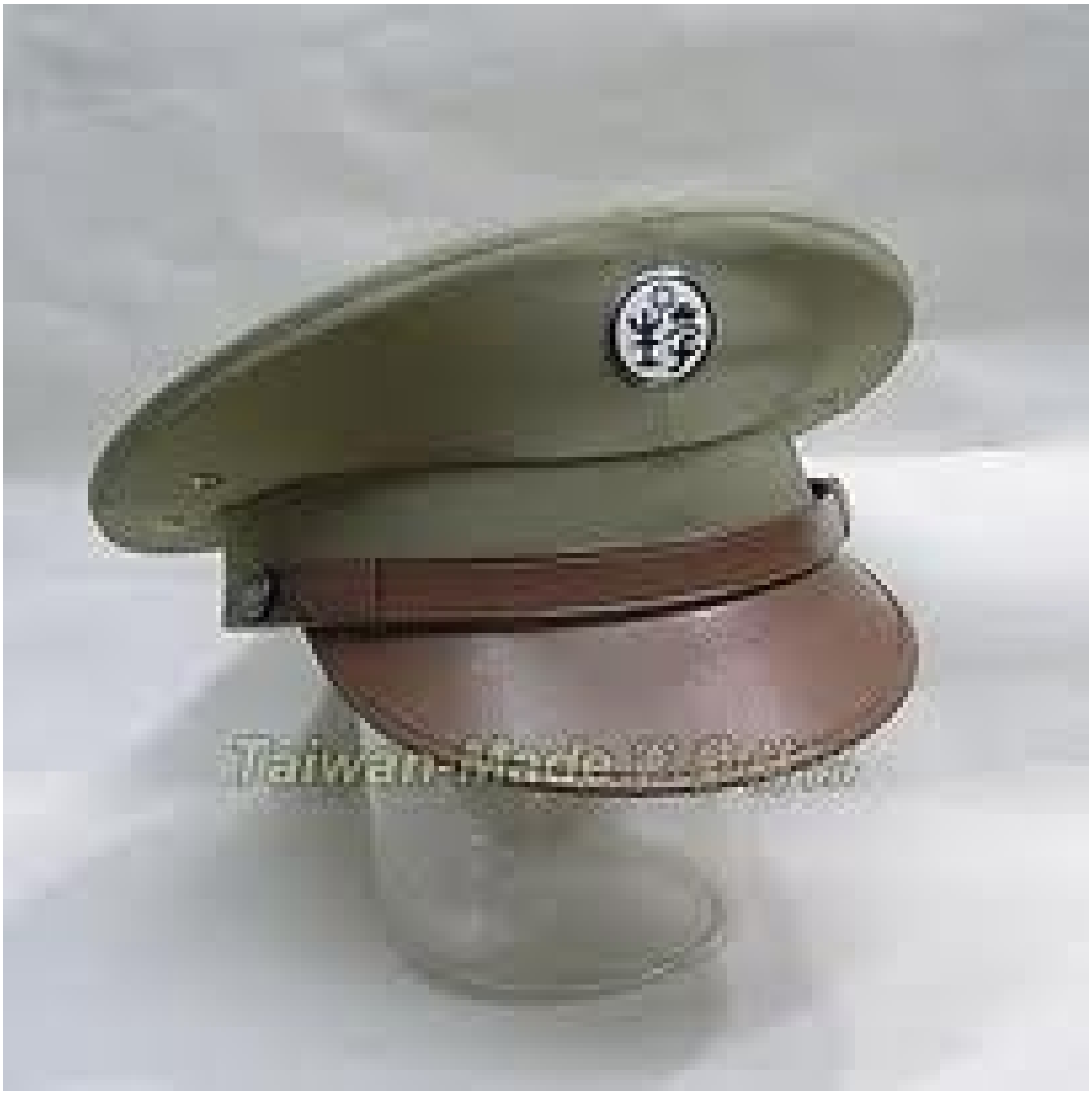

大盤帽

- 非常不同意
- 不同意
- 有點不同意
- 普通
- 有點同意
- 同意
- 非常同意

64. 對於這張圖片有熟悉感。 *

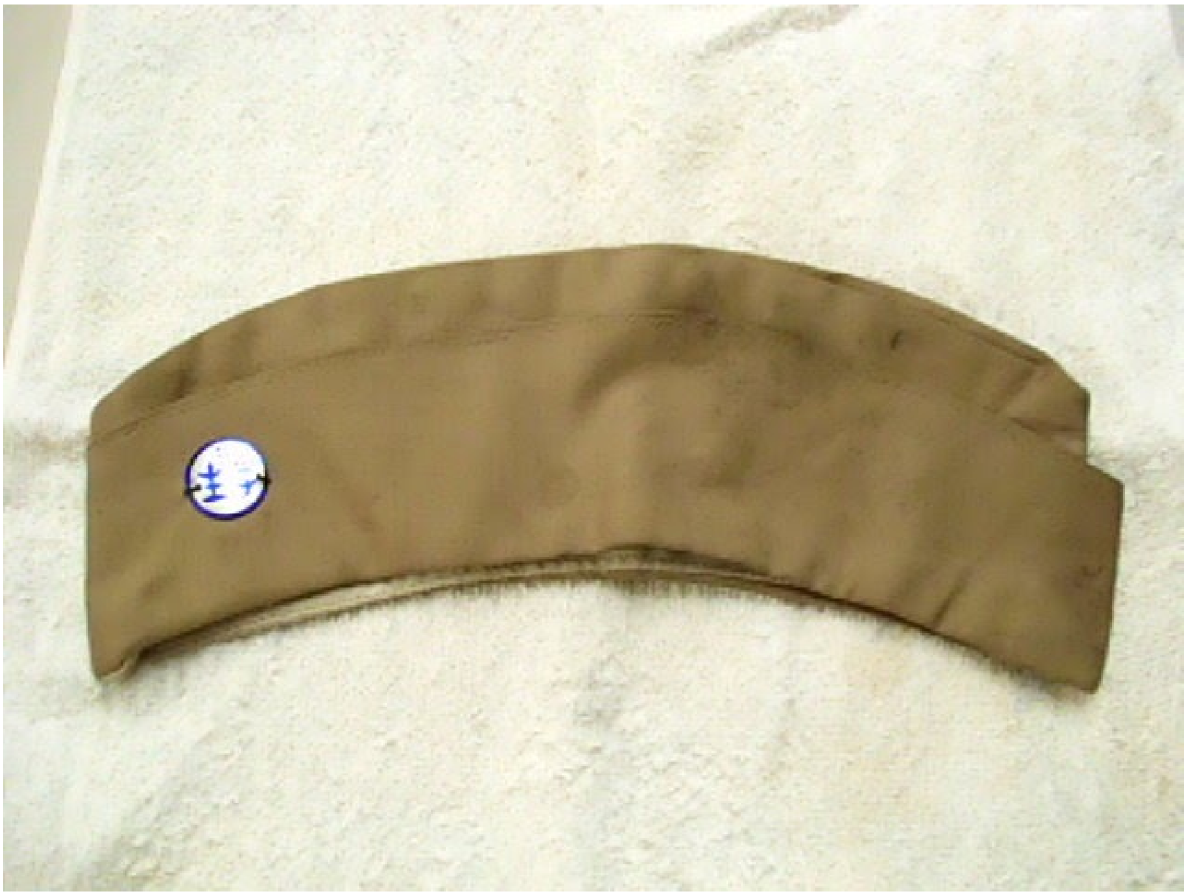

船型帽

- ◯ 非常不同意
- ◯ 不同意
- ◯ 有點不同意
- ◯ 普通
- ◯ 有點同意
- ◯ 同意
- ◯ 非常同意

65. 對於這張圖片有熟悉感。 *

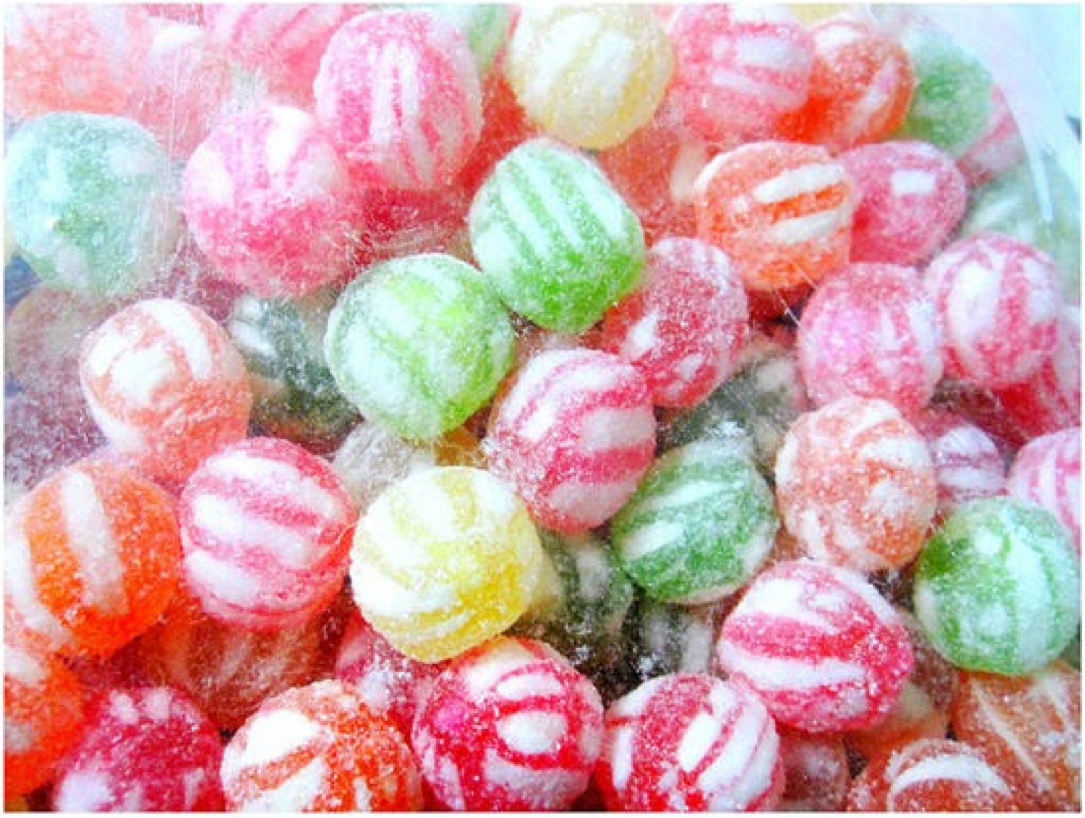

西瓜糖

- 非常不同意
- 不同意
- 有點不同意
- 普通
- 有點同意
- 同意
- 非常同意

66. 對於這張圖片有熟悉感。 *

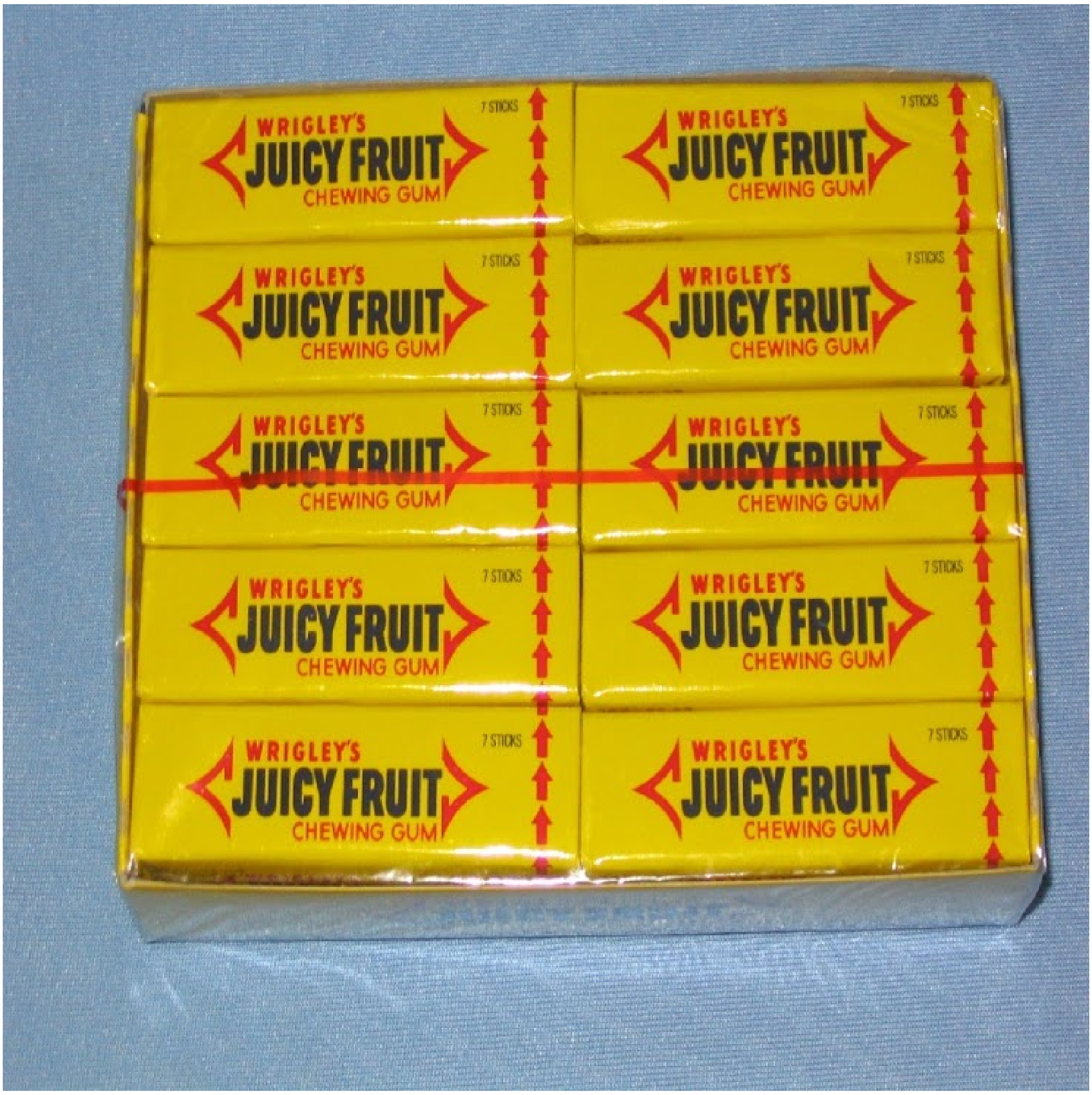

黃箭口香糖

- 非常不同意
- 不同意
- 有點不同意
- 普通
- 有點同意
- 同意
- 非常同意

67. 對於這張圖片有熟悉感。 *

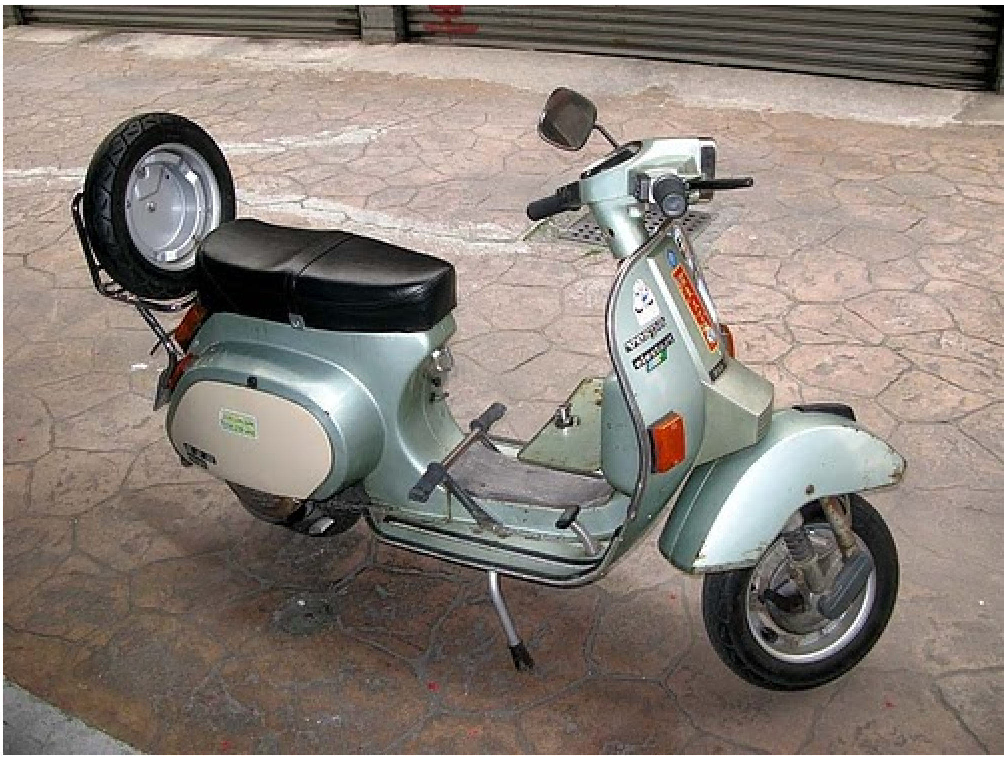

偉士牌機車

- 非常不同意
- 不同意
- 有點不同意
- 普通
- 有點同意
- 同意
- 非常同意

68. 對於這張圖片有熟悉感。 *

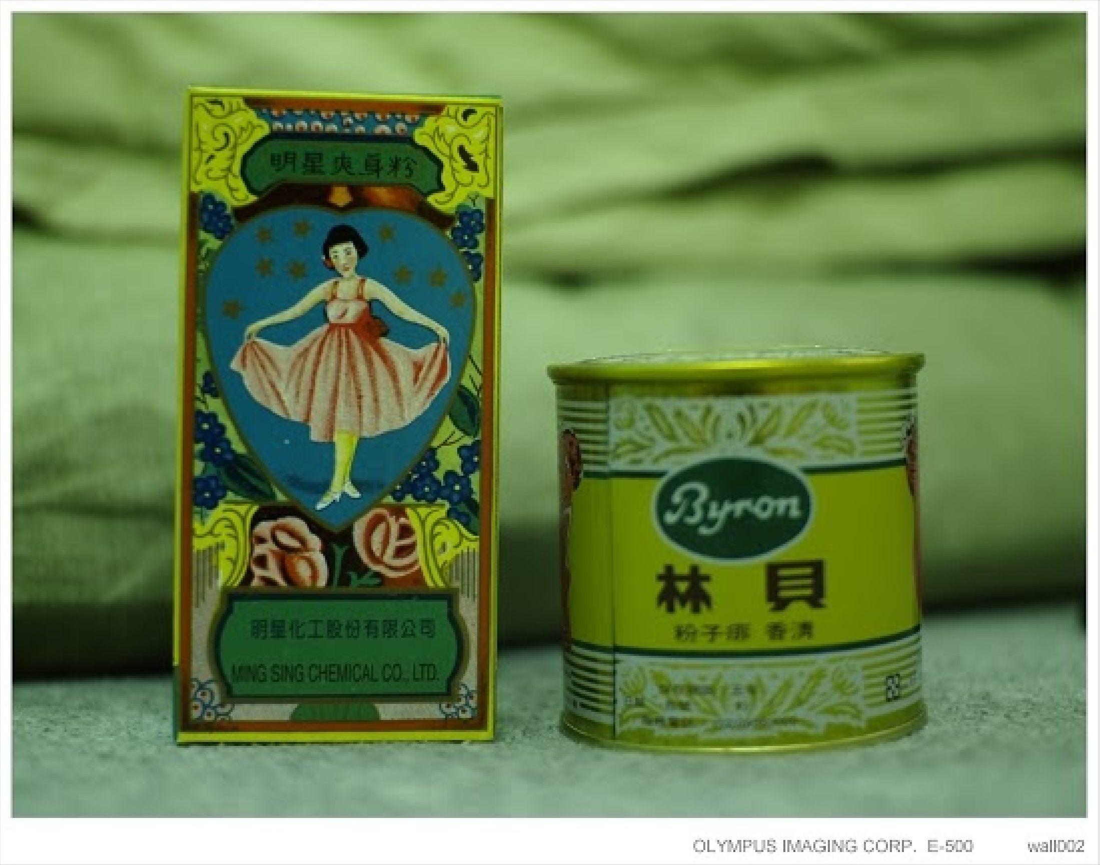

痱子粉

- ◯ 非常不同意
- ◯ 不同意
- ◯ 有點不同意
- ◯ 普通
- ◯ 有點同意
- ◯ 同意
- ◯ 非常同意

69. 對於這張圖片有熟悉感。 *

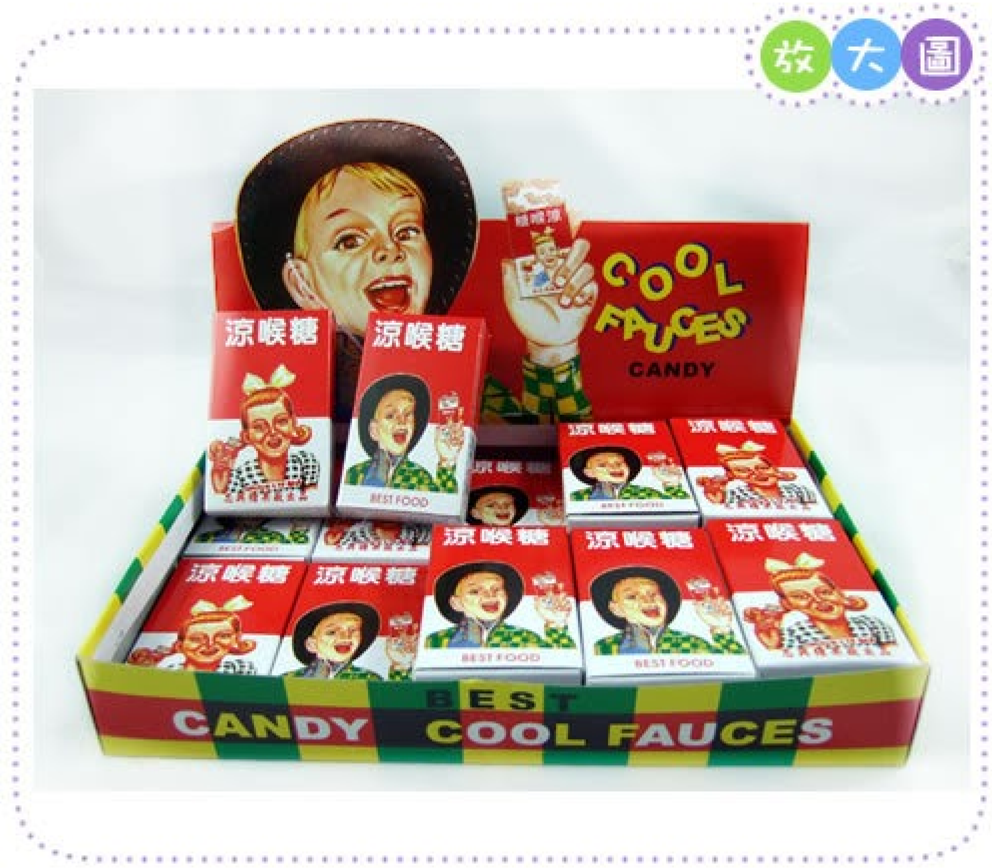

涼喉糖

- 非常不同意
- 不同意
- 有點不同意
- 普通
- 有點同意
- 同意
- 非常同意

70. 對於這張圖片有熟悉感。 *

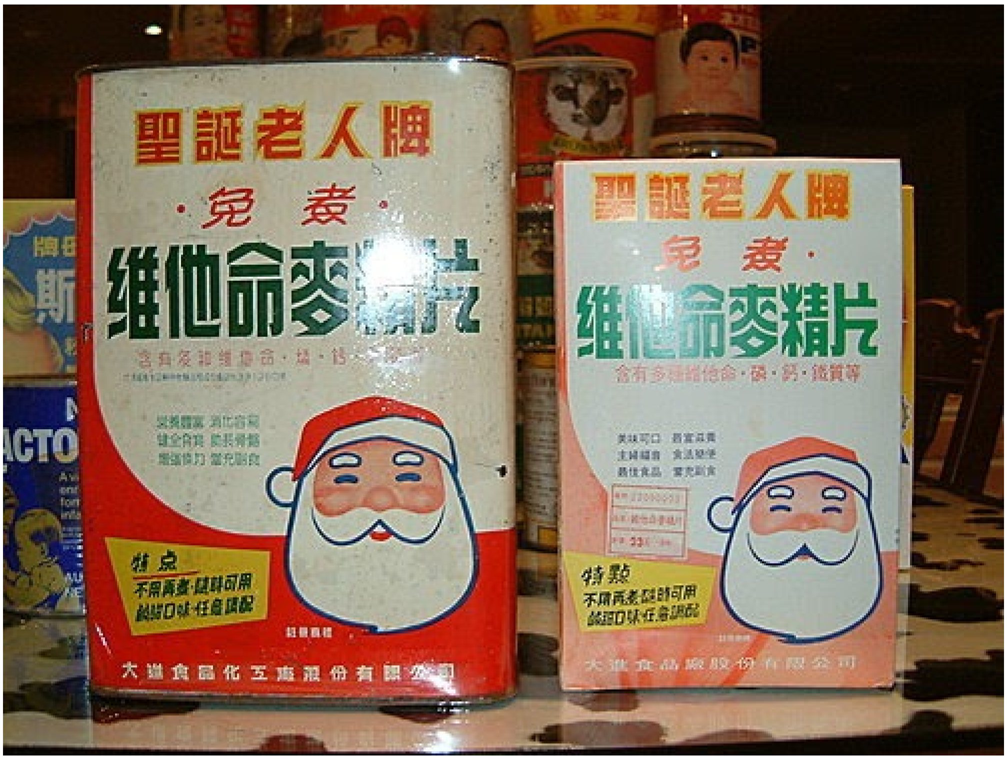

聖誕老人牌

- ◯ 非常不同意
- ◯ 不同意
- ◯ 有點不同意
- ◯ 普通
- ◯ 有點同意
- ◯ 同意
- ◯ 非常同意

71. 對於這張圖片有熟悉感。 *

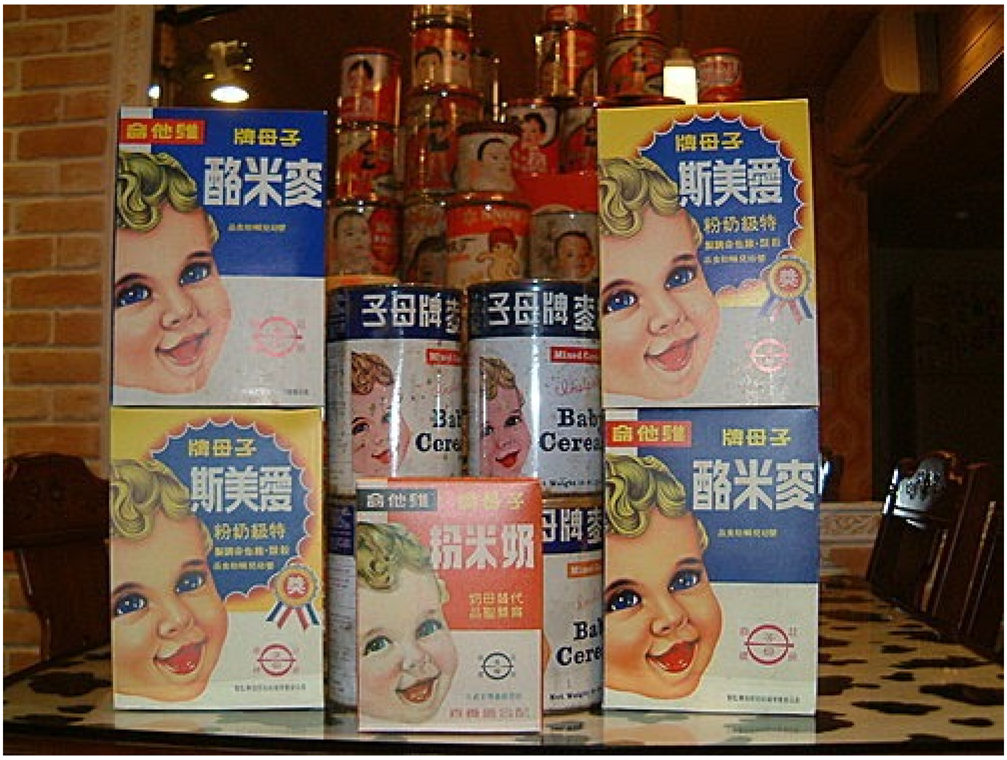

子母牌奶粉

- 非常不同意
- 不同意
- 有點不同意
- 普通
- 有點同意
- 同意
- 非常同意

72. 對於這張圖片有熟悉感。 *

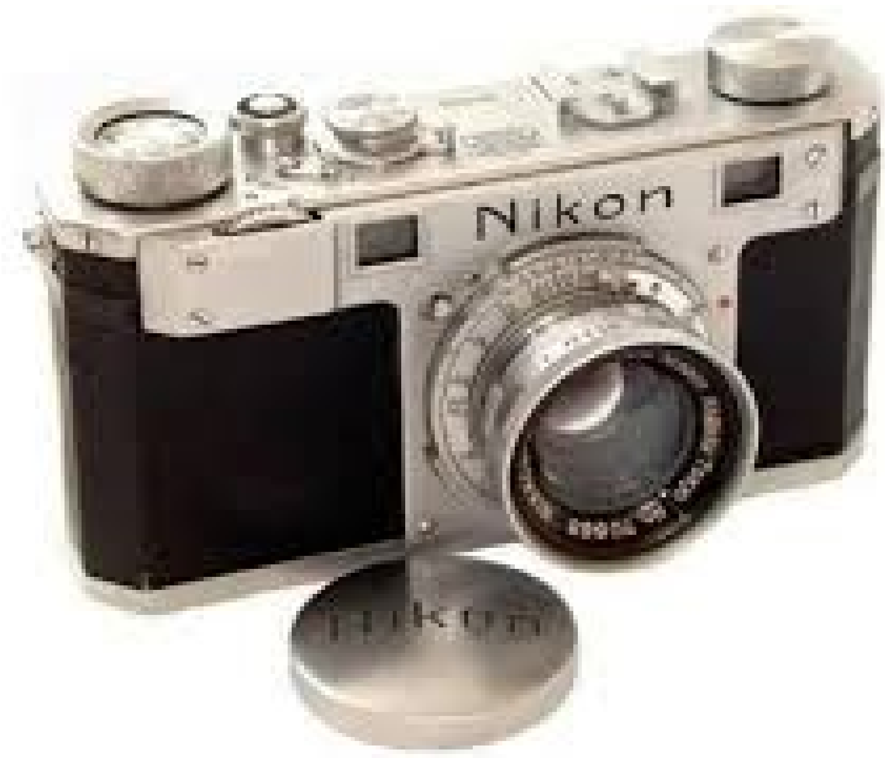

相機

- ◯ 非常不同意
- ◯ 不同意
- ◯ 有點不同意
- ◯ 普通
- ◯ 有點同意
- ◯ 同意
- ◯ 非常同意

73. 對於這張圖片有熟悉感。 *

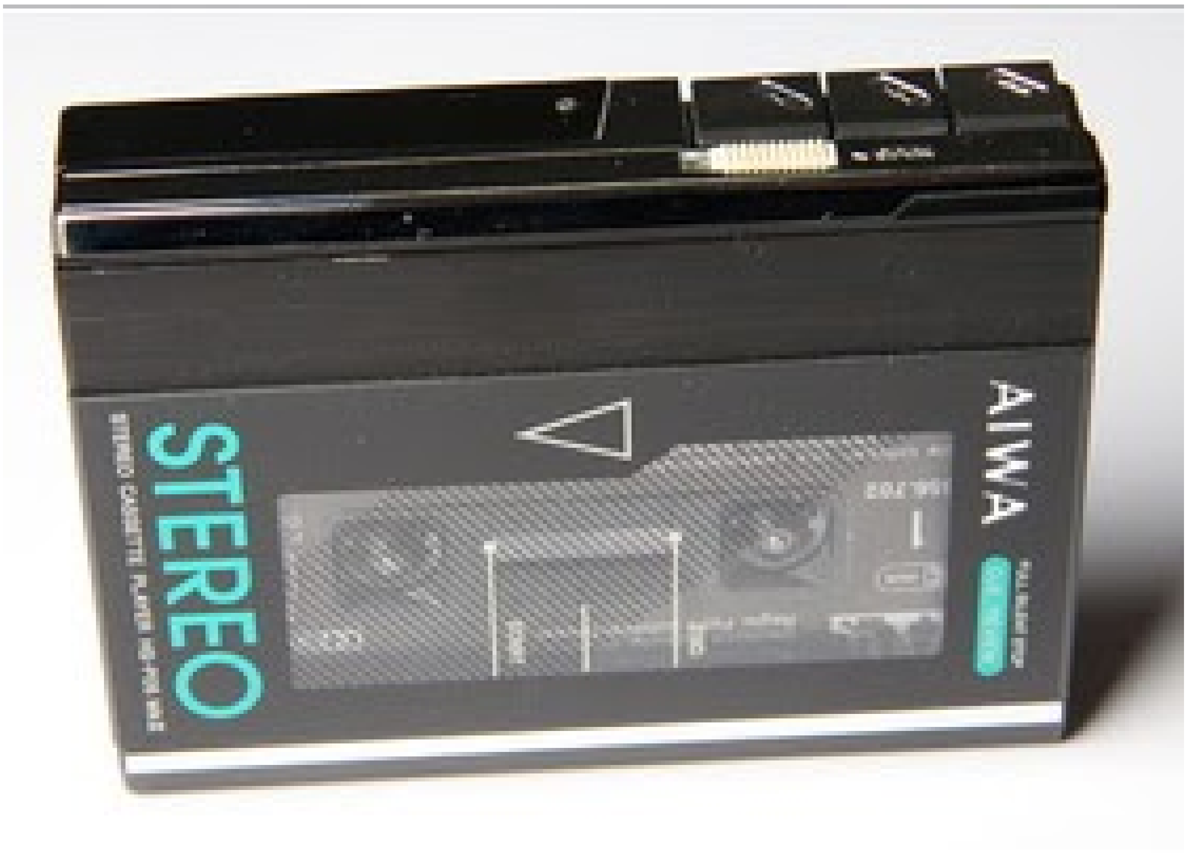

錄音帶

- ◯ 非常不同意
- ◯ 不同意
- ◯ 有點不同意
- ◯ 普通
- ◯ 有點同意
- ◯ 同意
- ◯ 非常同意

74. 對於這張圖片有熟悉感。 *

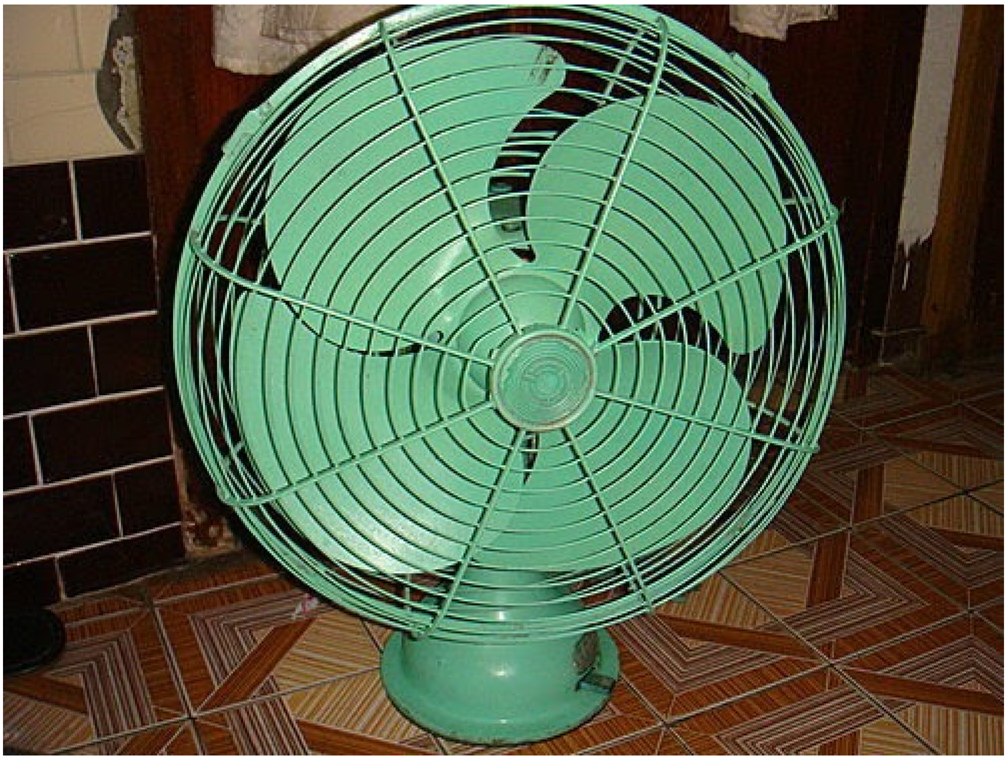

大同電扇

- 非常不同意
- 不同意
- 有點不同意
- 普通
- 有點同意
- 同意
- 非常同意

75. 對於這張圖片有熟悉感。 *

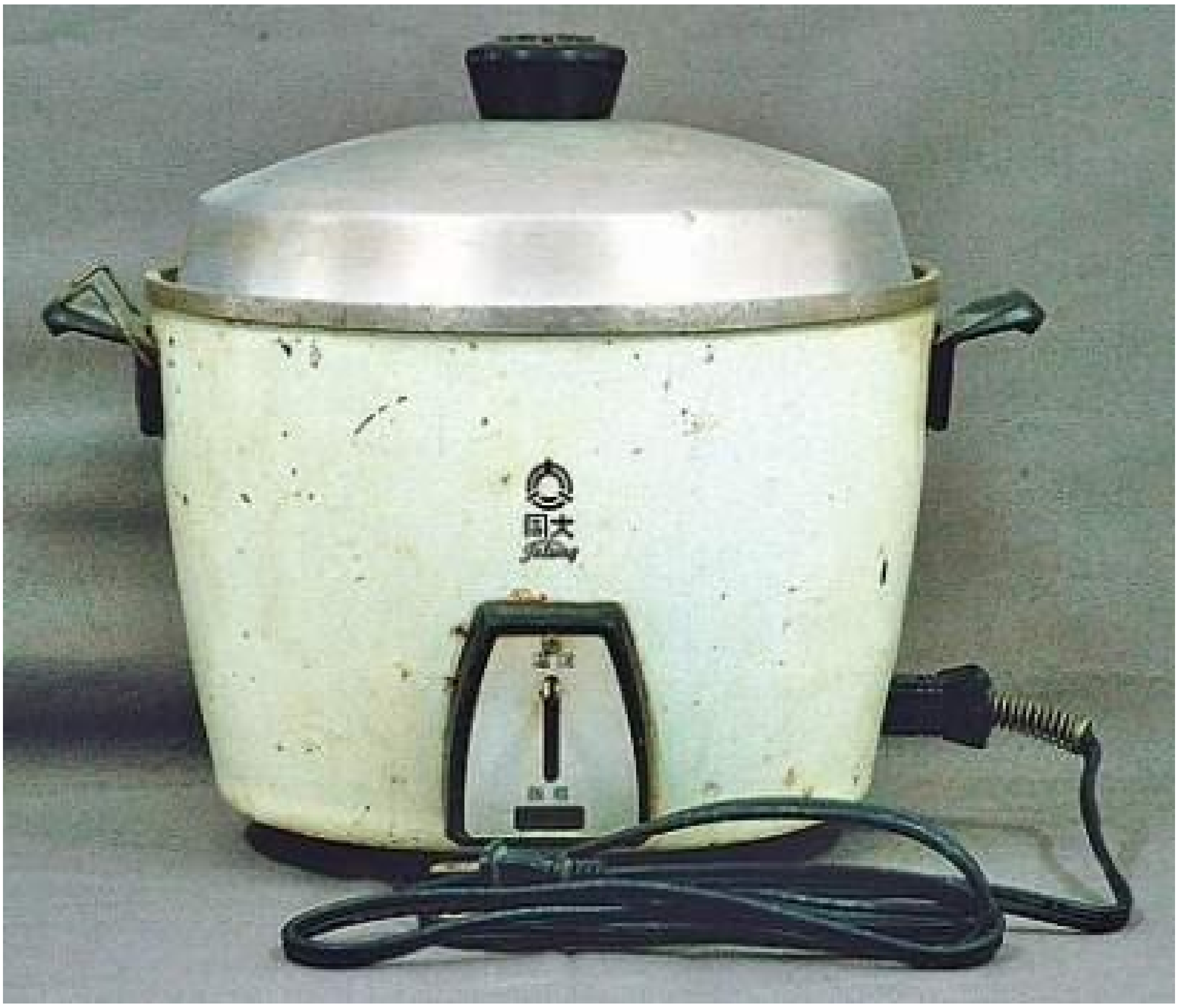

大同電鍋

- 非常不同意
- 不同意
- 有點不同意
- 普通
- 有點同意
- 同意
- 非常同意

76. 對於這張圖片有熟悉感。 *

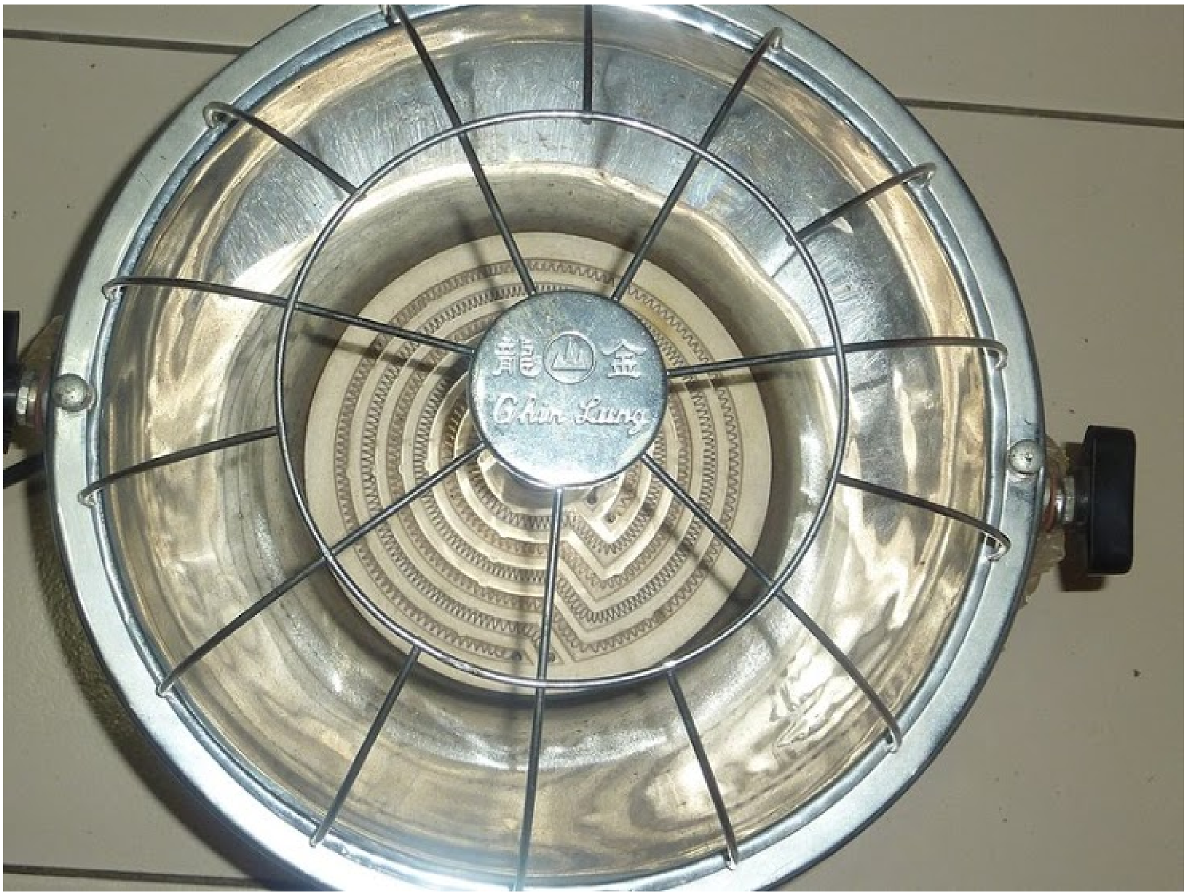

鎢絲電暖爐

- ◯ 非常不同意
- ◯ 不同意
- ◯ 有點不同意
- ◯ 普通
- ◯ 有點同意
- ◯ 同意
- ◯ 非常同意

77. 對於這張圖片有熟悉感。 *

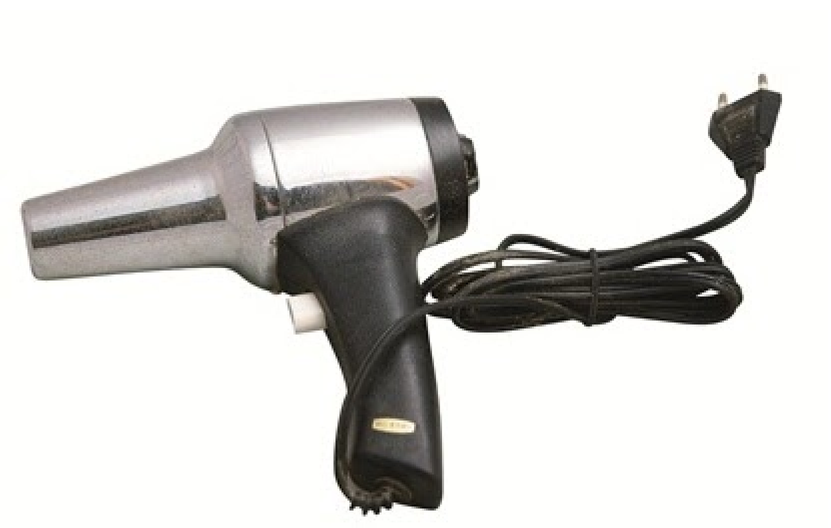

吹風機

- 非常不同意
- 不同意
- 有點不同意
- 普通
- 有點同意
- 同意
- 非常同意

78. 對於這張圖片有熟悉感。 *

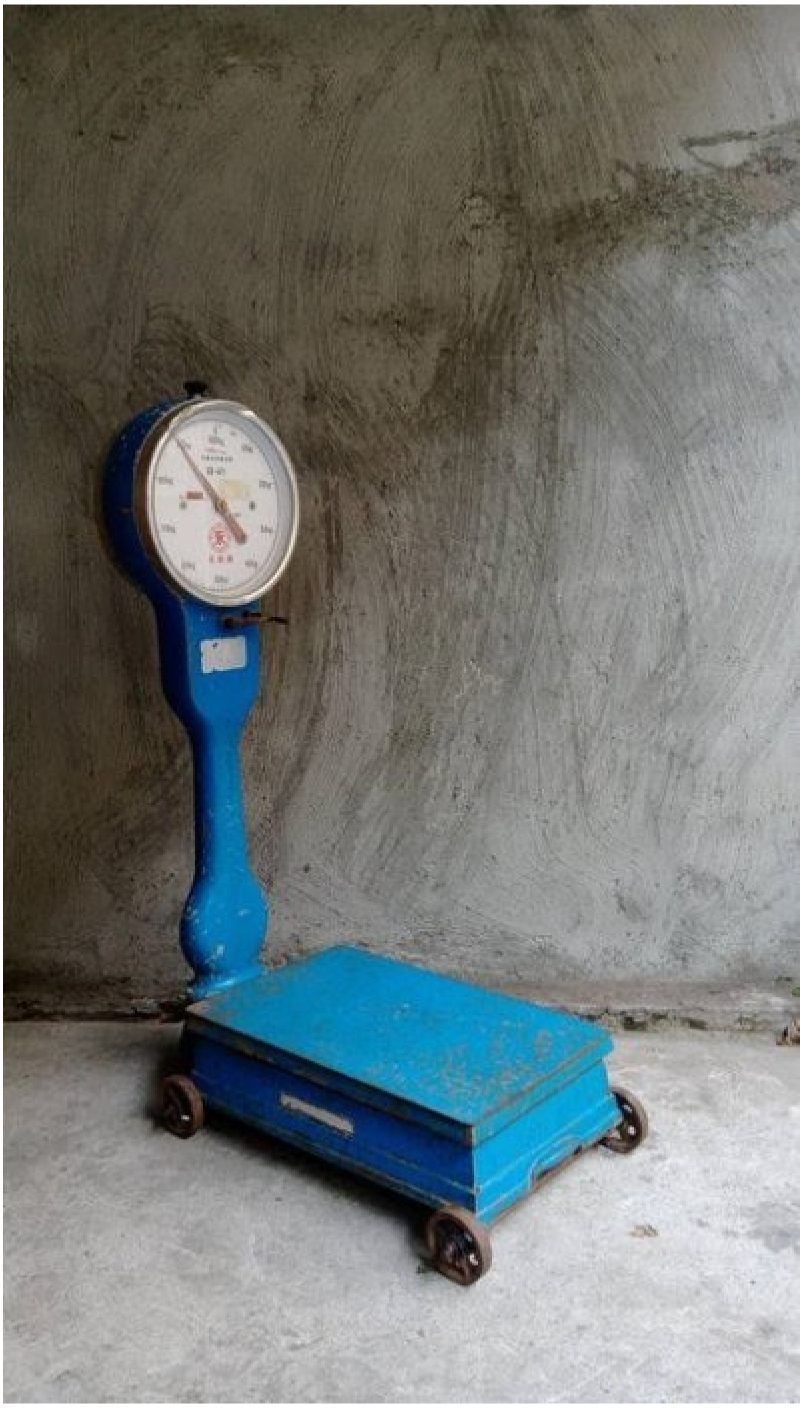

體重計

- 非常不同意
- 不同意
- 有點不同意
- 普通
- 有點同意
- 同意

○ 非常同意

79. 對於這張圖片有熟悉感。 *

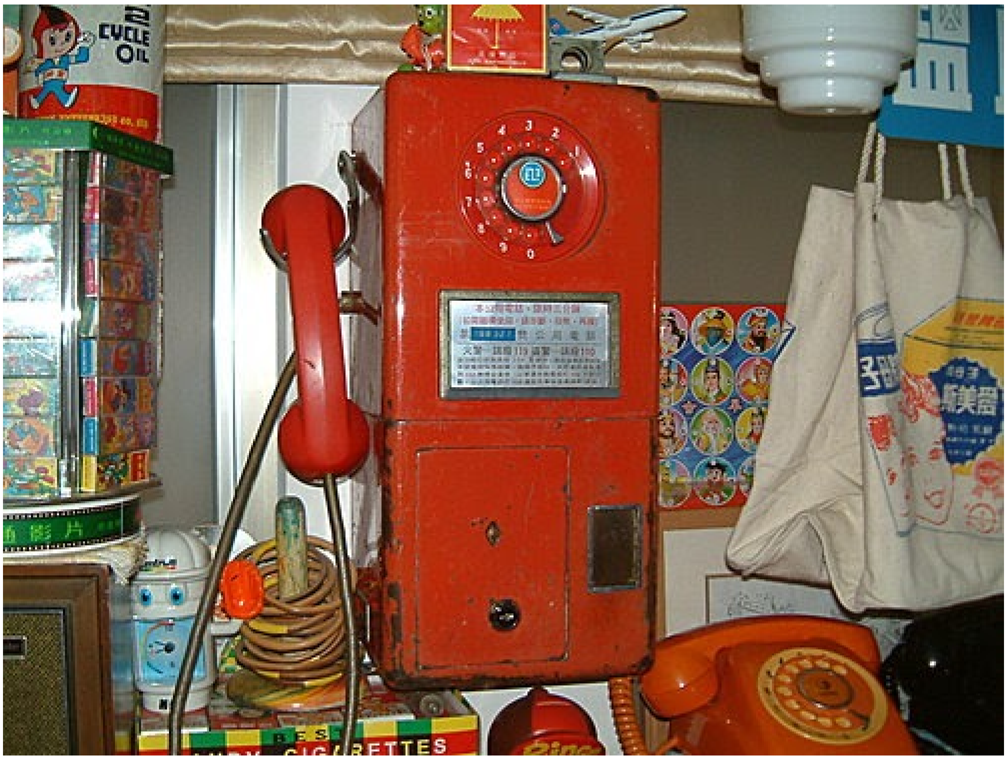

公用電話

○ 非常不同意

○ 不同意

○ 有點不同意

○ 普通

○ 有點同意

○ 同意

○ 非常同意

80. 對於這張圖片有熟悉感。 *

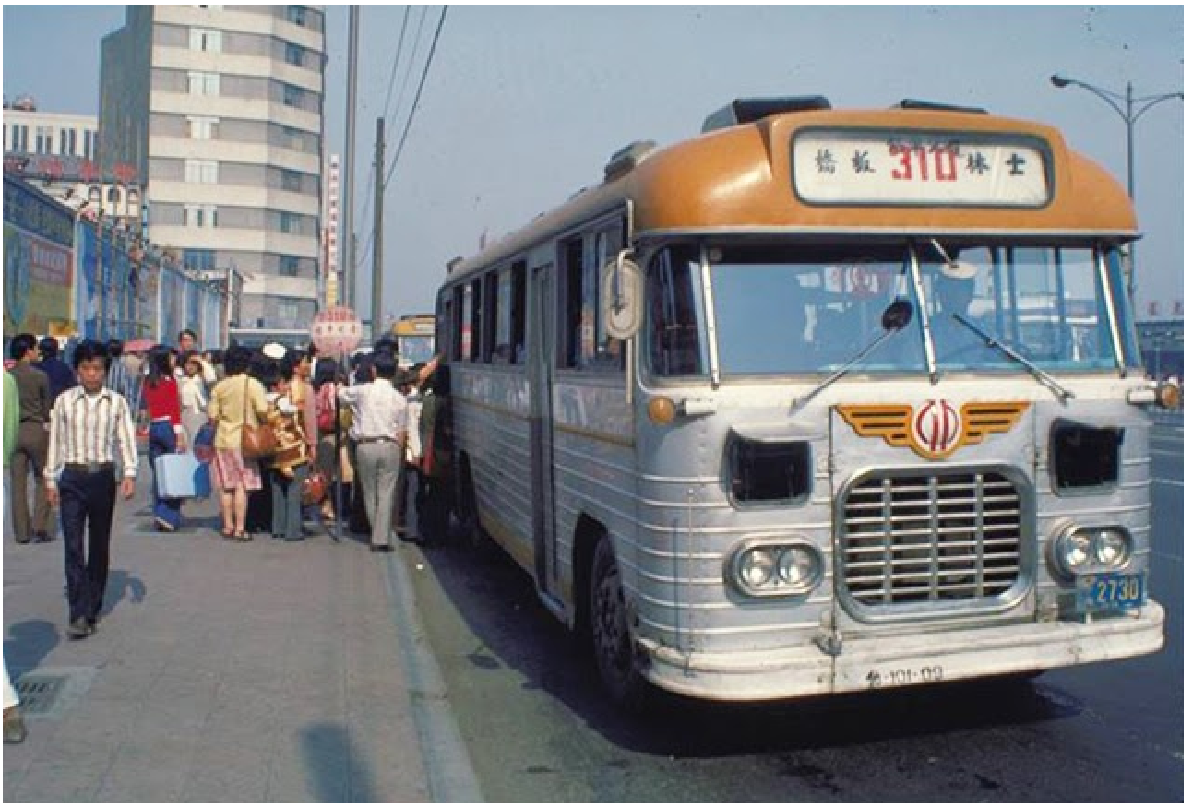

公車

- 非常不同意
- 不同意
- 有點不同意
- 普通
- 有點同意
- 同意
- 非常同意

81. 對於這張圖片有熟悉感。 *

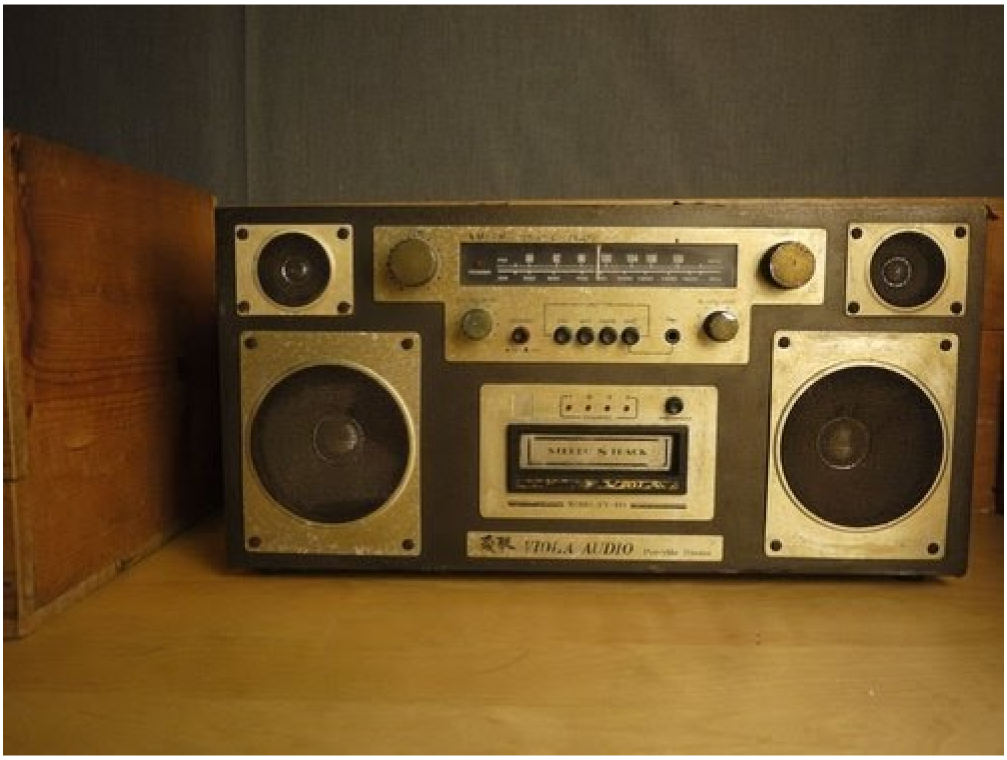

收音機

- 非常不同意
- 不同意
- 有點不同意
- 普通
- 有點同意
- 同意
- 非常同意

82. 對於這張圖片有熟悉感。 *

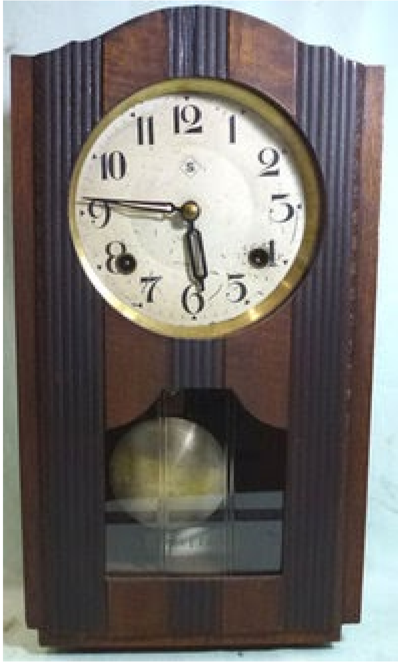

時鐘

- 非常不同意
- 不同意
- 有點不同意
- 普通
- 有點同意
- 同意
- 非常同意

83. 對於這張圖片有熟悉感。 *

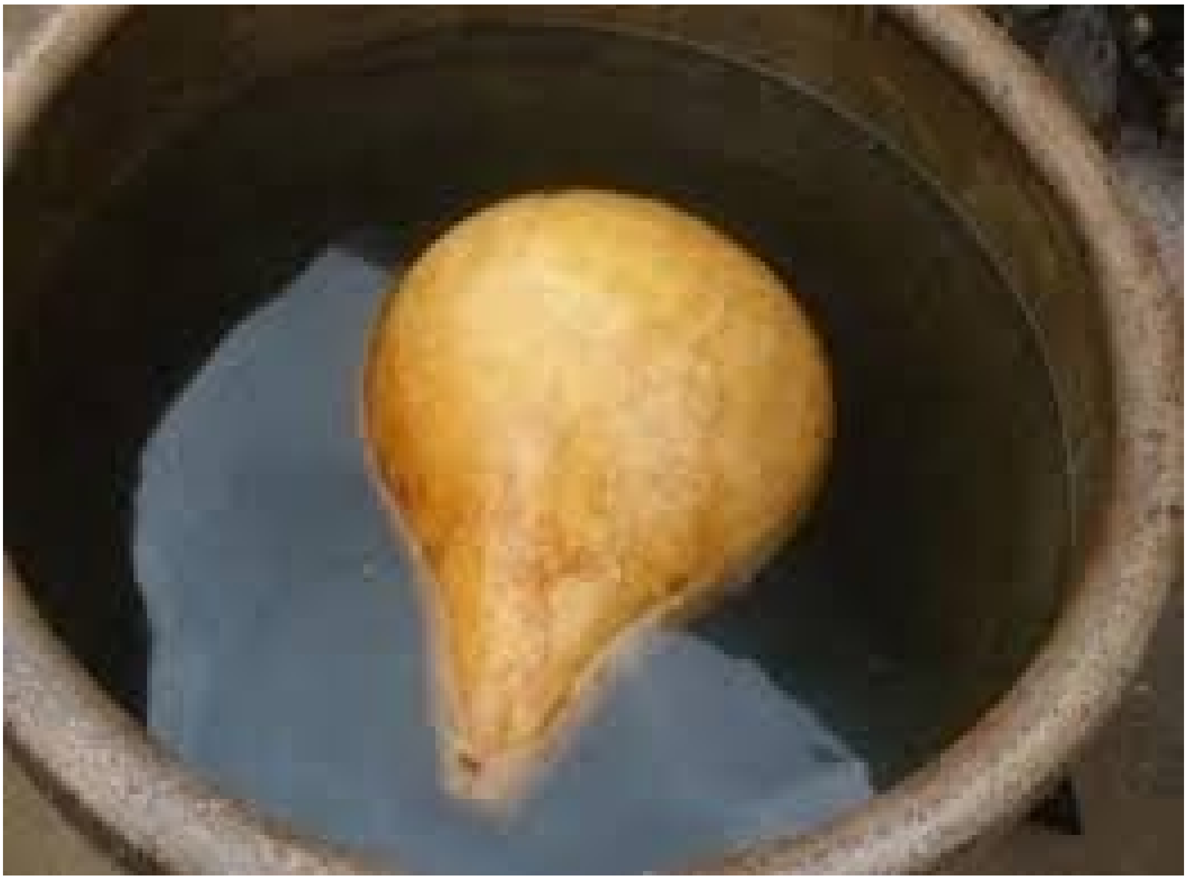

水瓢

- 非常不同意
- 不同意
- 有點不同意
- 普通
- 有點同意
- 同意
- 非常同意

84. 對於這張圖片有熟悉感。 *

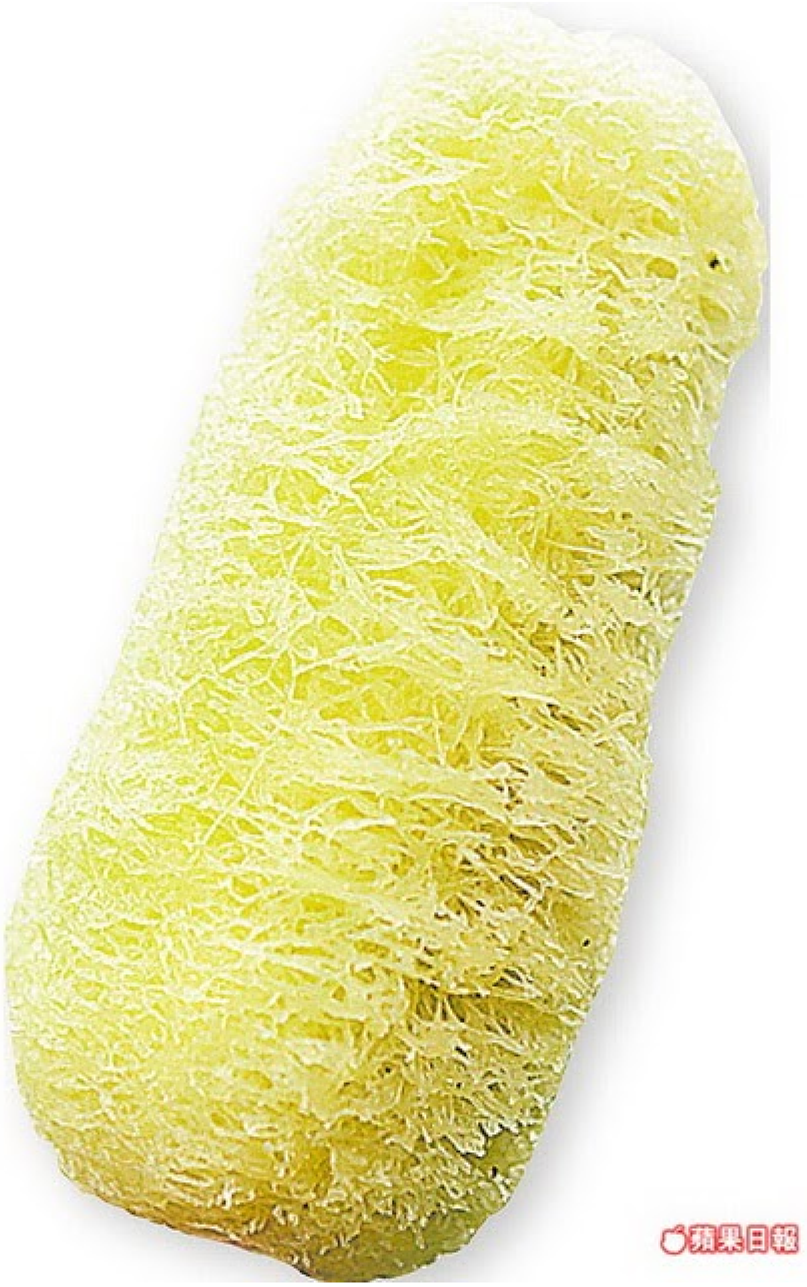

絲瓜布

- 非常不同意
- 不同意
- 有點不同意
- 普通
- 有點同意
- 同意
- 非常同意

85. 對於這張圖片有熟悉感。 *

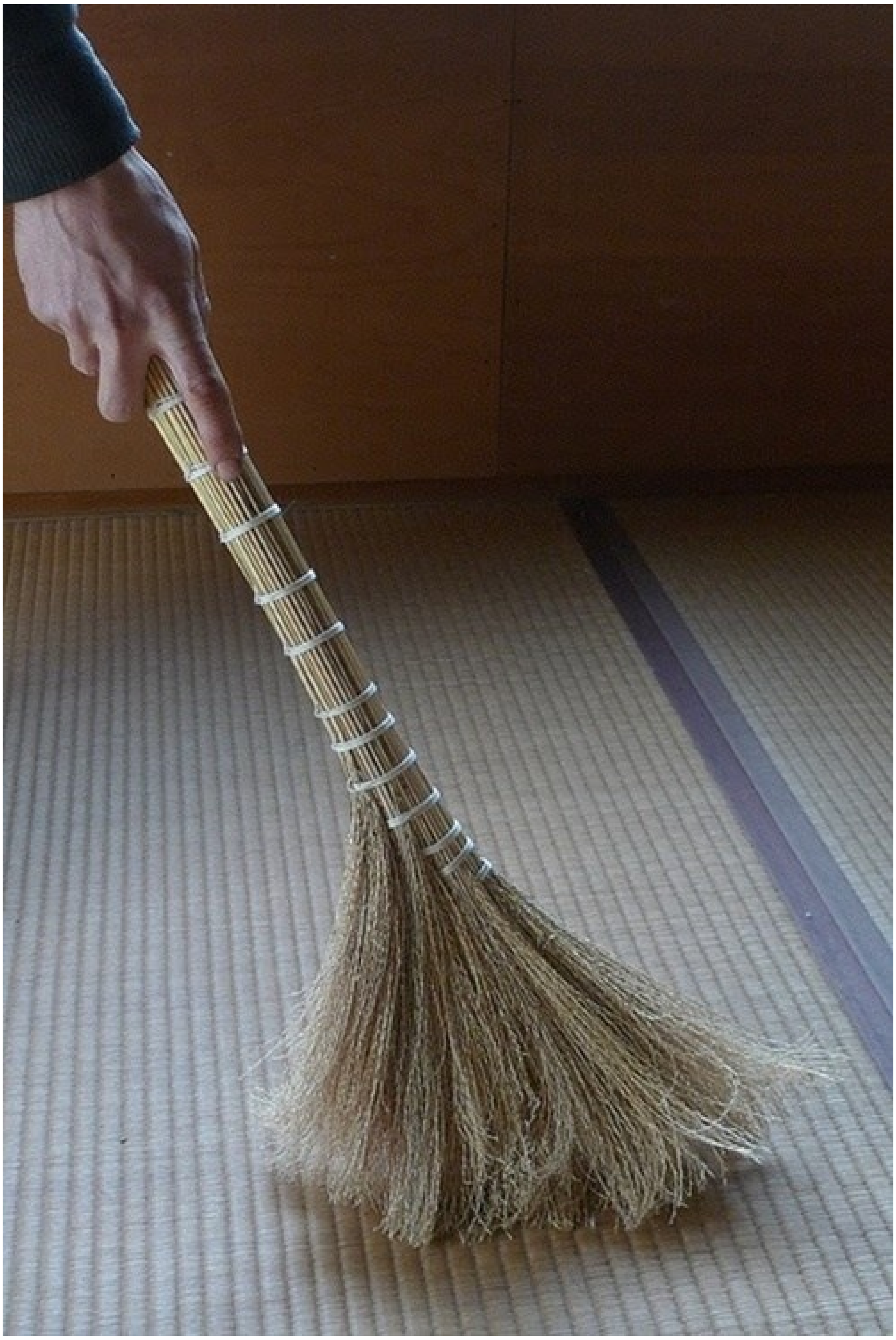

掃把

◯ 非常不同意

◯ 不同意

◯ 有點不同意

◯ 普通

◯ 有點同意

- 同意
- 非常同意

86. 對於這張圖片有熟悉感。 *

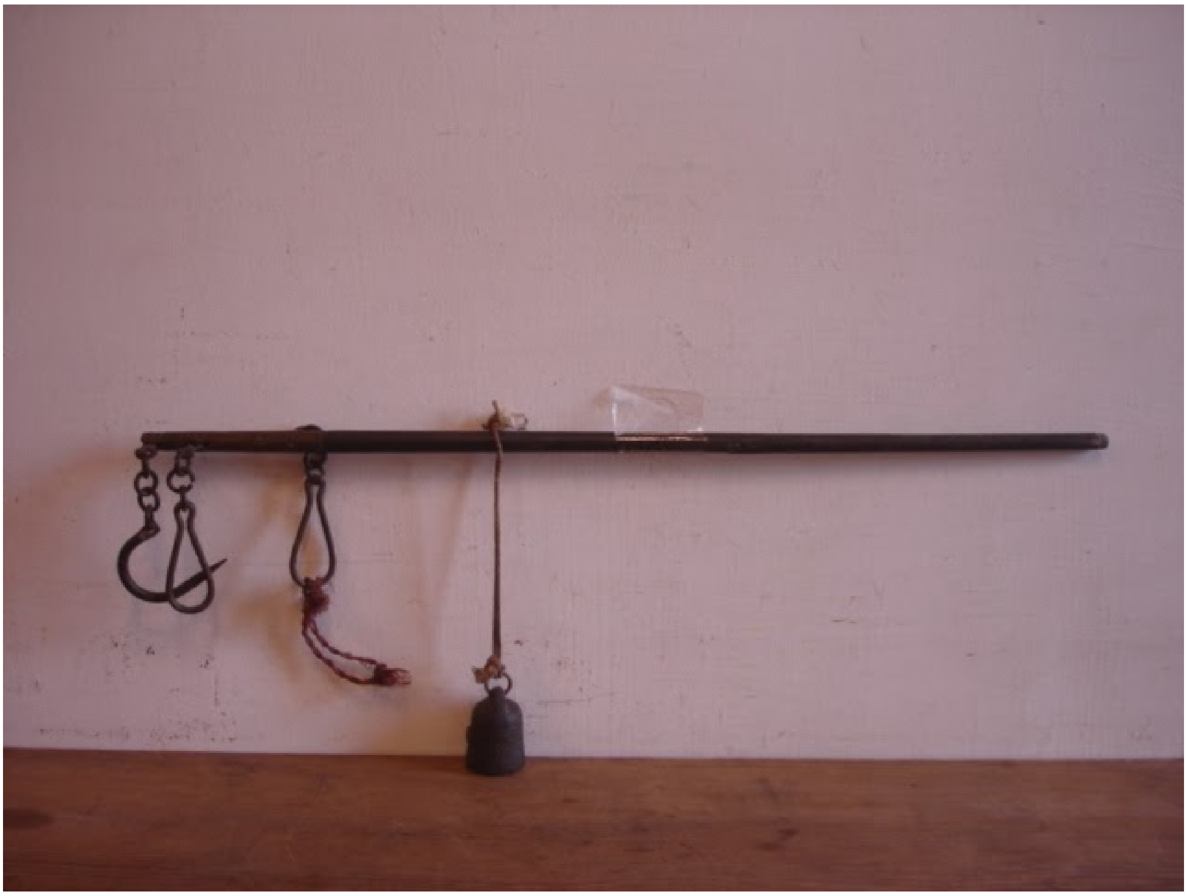

秤子

- 非常不同意
- 不同意
- 有點不同意
- 普通
- 有點同意
- 同意
- 非常同意

87. 對於這張圖片有熟悉感。 *

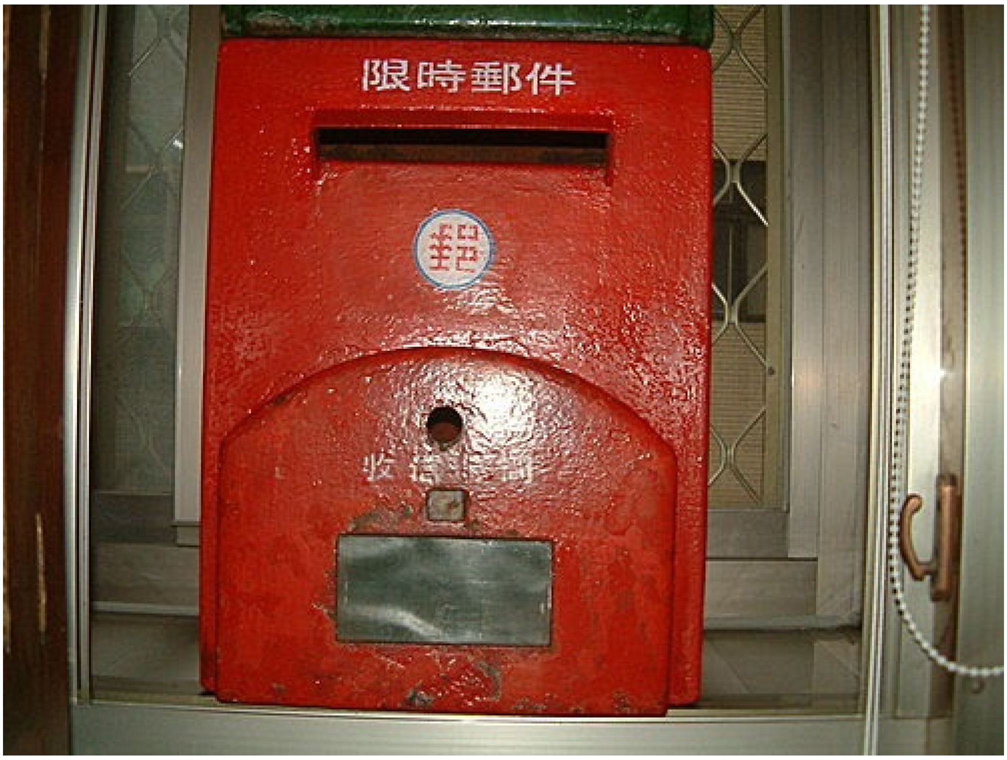

郵筒

- 非常不同意
- 不同意
- 有點不同意
- 普通
- 有點同意
- 同意
- 非常同意

88. 對於這張圖片有熟悉感。 *

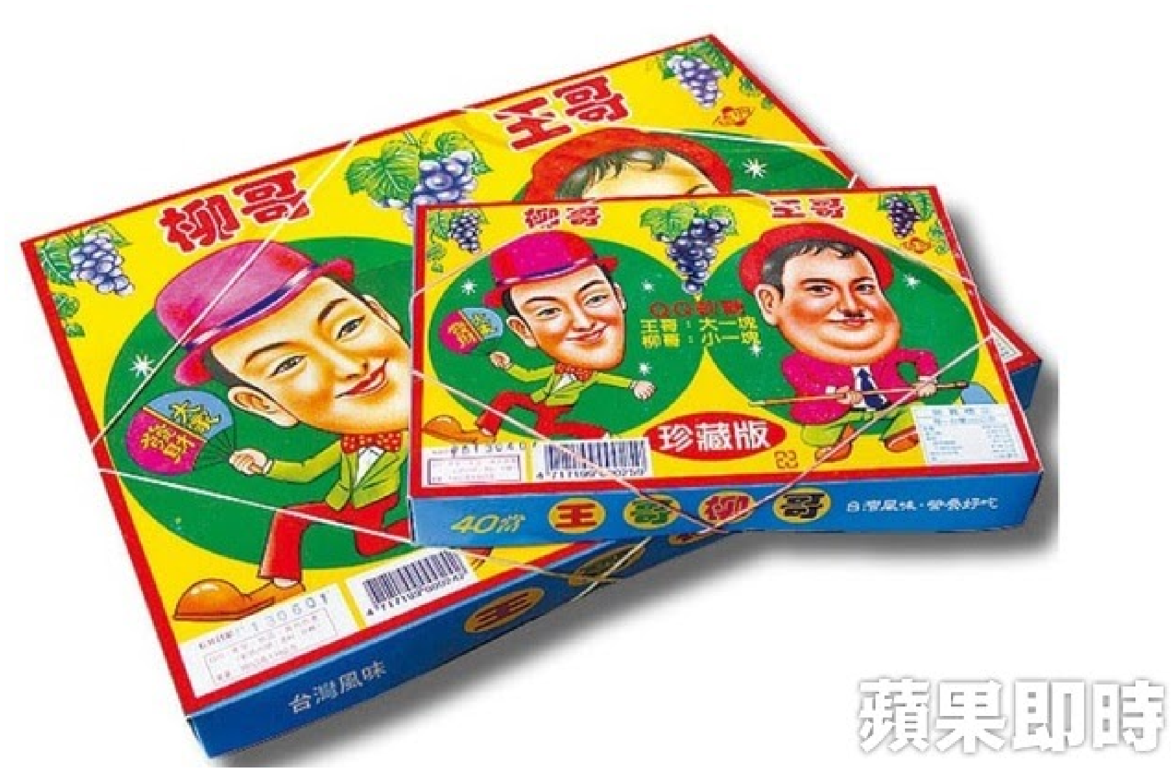

軟糖

- 非常不同意
- 不同意
- 有點不同意
- 普通
- 有點同意
- 同意
- 非常同意

89. 對於這張圖片有熟悉感。 *

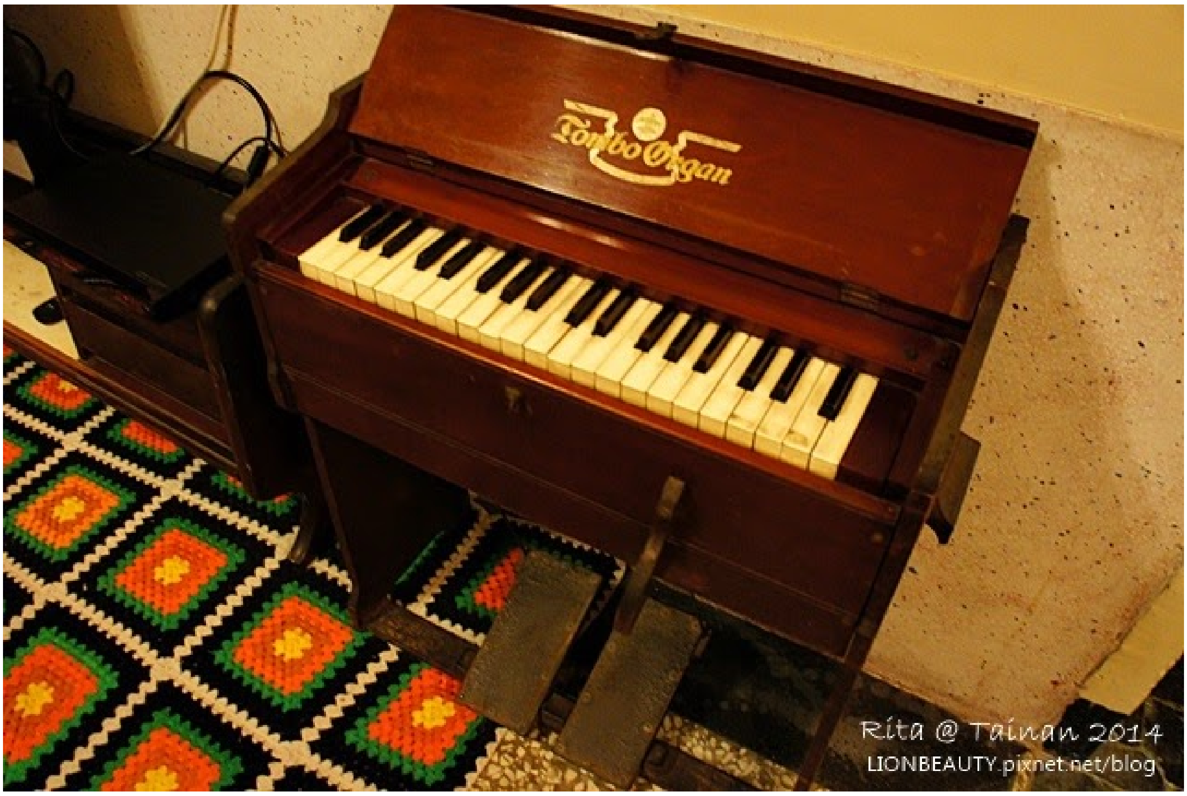

風琴

- ◯ 非常不同意
- ◯ 不同意
- ◯ 有點不同意
- ◯ 普通
- ◯ 有點同意
- ◯ 同意
- ◯ 非常同意

90. 對於這張圖片有熟悉感。 *

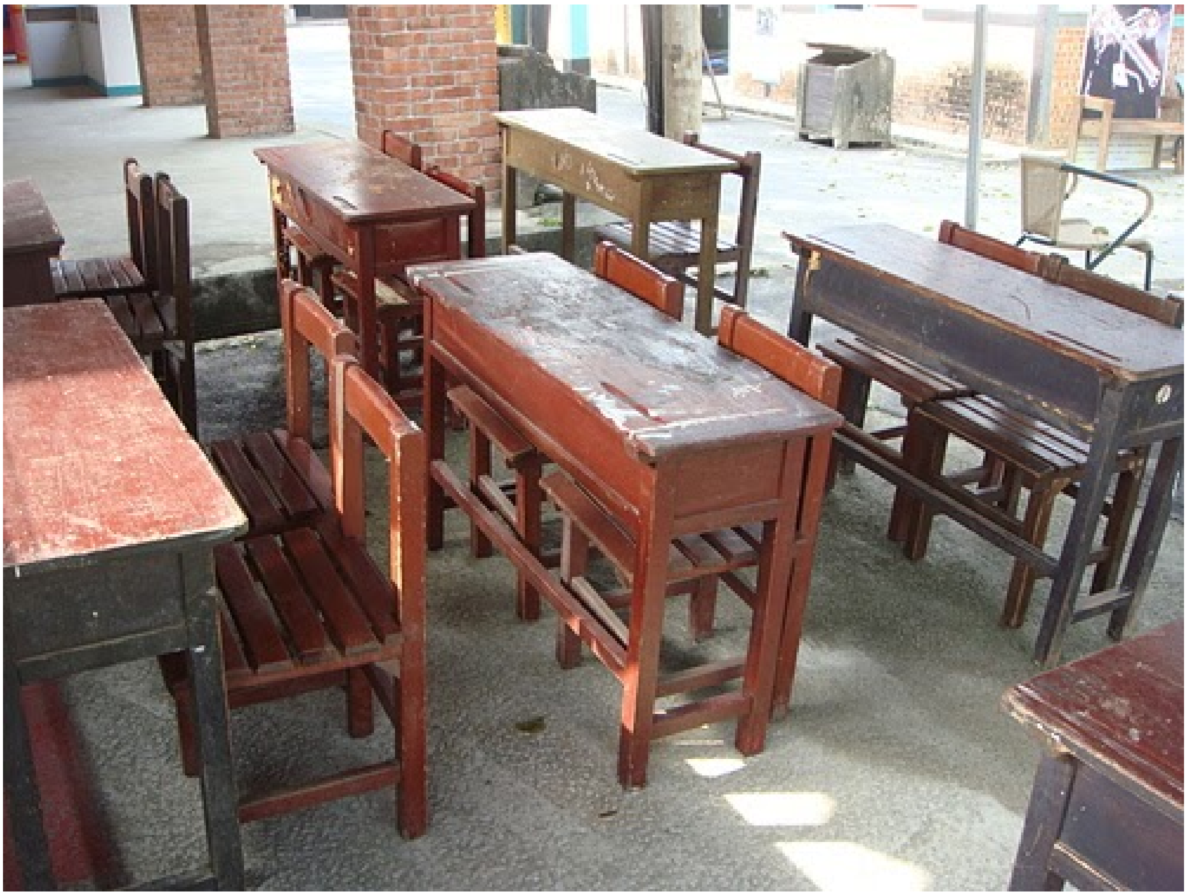

課桌椅

- 非常不同意
- 不同意
- 有點不同意
- 普通
- 有點同意
- 同意
- 非常同意

91. 對於這張圖片有熟悉感。 *

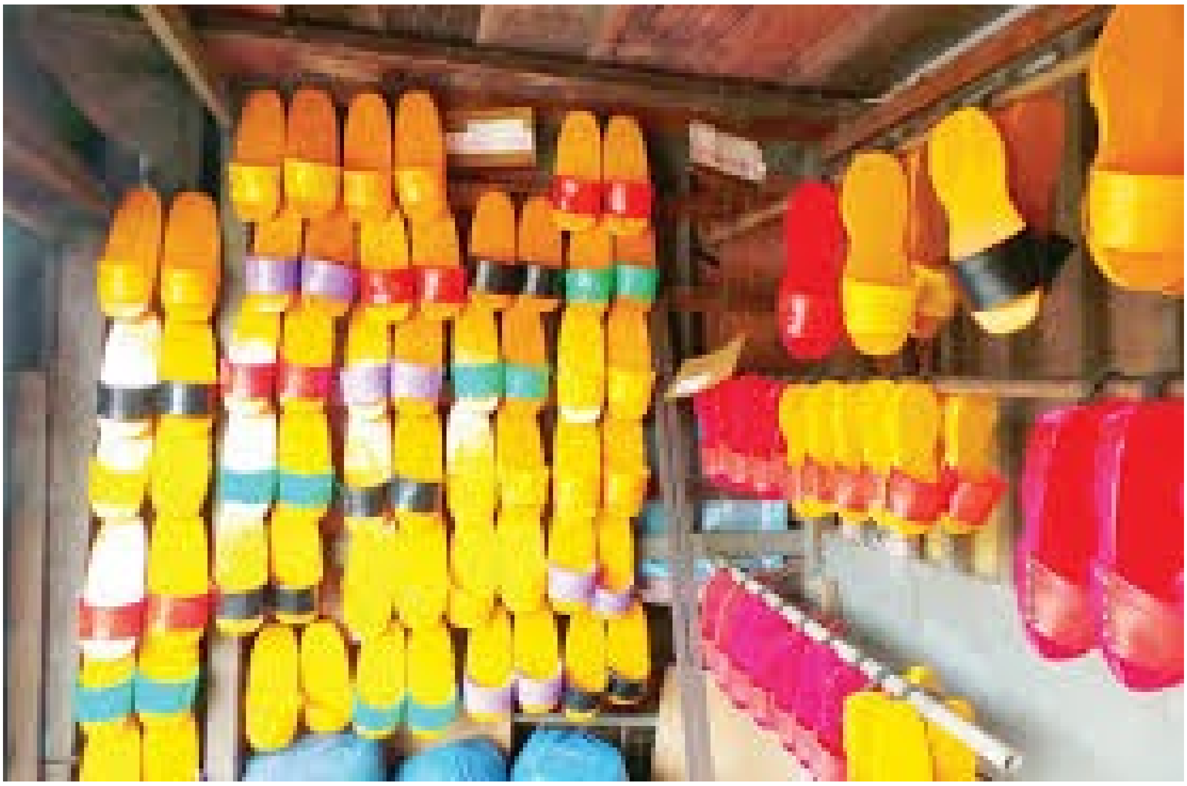

木屐

- 非常不同意
- 不同意
- 有點不同意
- 普通
- 有點同意
- 同意
- 非常同意

92. 對於這張圖片有熟悉感。 *

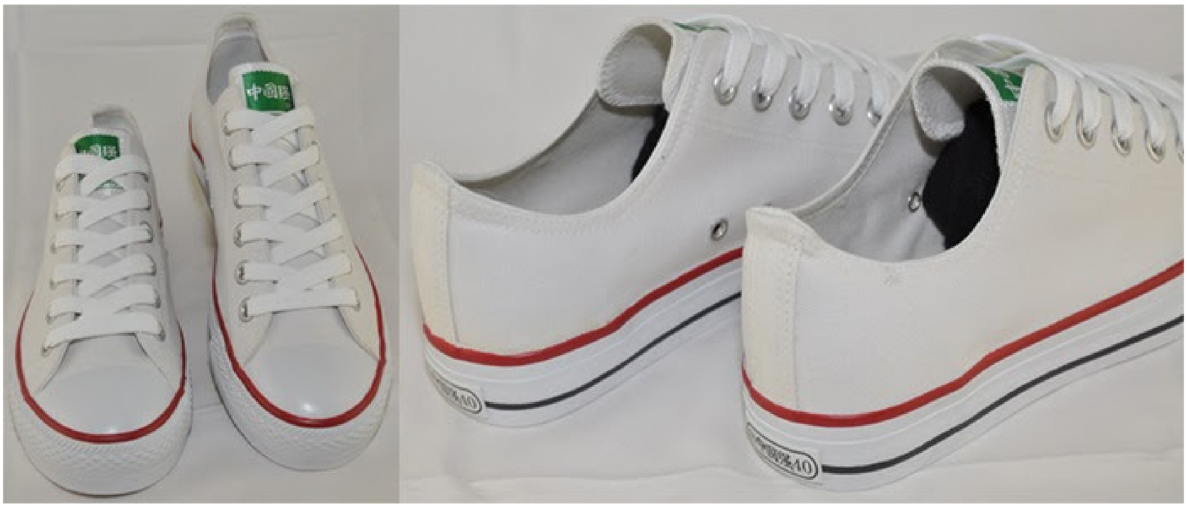

球鞋

- 非常不同意
- 不同意
- 有點不同意
- 普通
- 有點同意
- 同意
- 非常同意

93. 對於這張圖片有熟悉感。 *

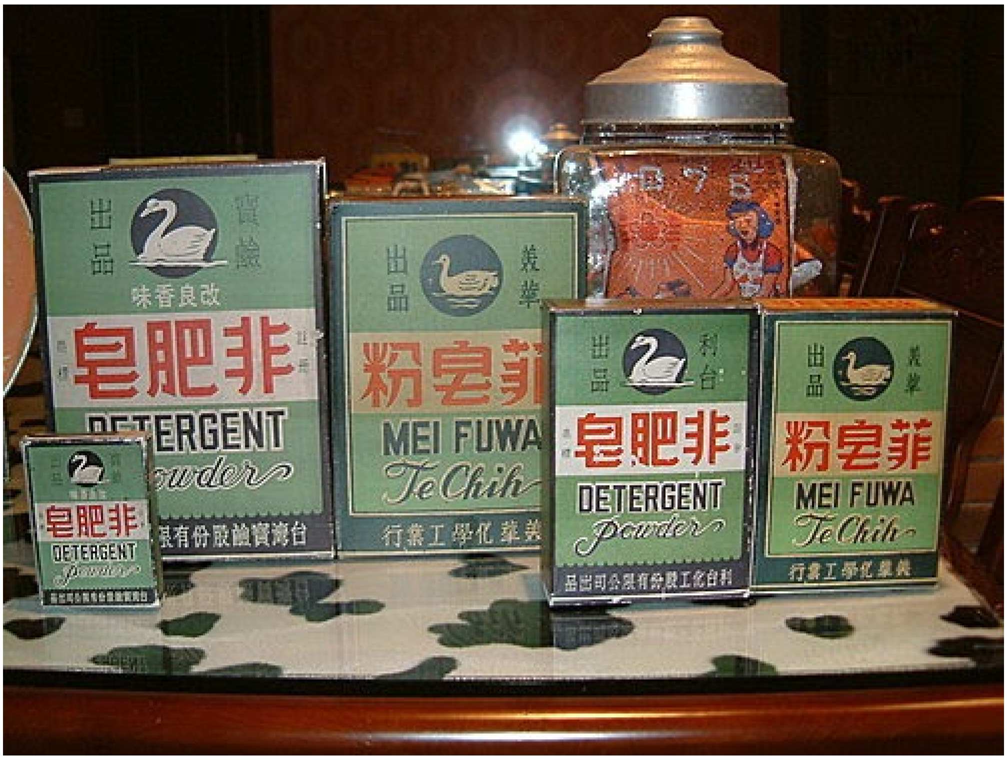

肥皂

- 非常不同意
- 不同意
- 有點不同意
- 普通
- 有點同意
- 同意
- 非常同意

其他對您來說有熟悉感的物品？(限60年代) *

您的回答

問卷到此結束！感謝您的耐心回答！敬祝　快樂如意！

提交

請勿利用 Google 表單送出密碼。

這份表單是在 大同大學 中建立。 檢舉濫用情形

Google 表單
