# Supplementary material for: A Cognitive-Based Board Game With Augmented Reality for Older Adults: Development and Usability Study
Source: JMIR Serious Games. 2020 Dec 14;8(4):e22007. doi: 10.2196/22007 (PMC7769693; doi:10.2196/22007)
Supplement: Multimedia Appendix 2 [file games_v8i4e22007_app2.pdf]

Survey on Impressions of the 1960s

Dear friends,
Thank you for taking the time to fill out this questionnaire. This survey is used to study the impressions of the 1960s in 50 to 59 year olds. The survey is anonymous. The information you provide will only be used for academic purposes, and will not be shared. We value your opinion and hope you can fill out the survey based on your honest opinions. Thank you for your support!

*必填

Basic Information

Sex *

- ◯ Male
- ◯ Female

Education Level *

- ◯ Less than Jr High School
- ◯ High School
- ◯ Bachelors or Associates Degree
- ◯ Graduate Degree

Age *

您的回答

Occupation *

您的回答

Your impressions of the 1960's

1. Please rate your familiarity with this image *

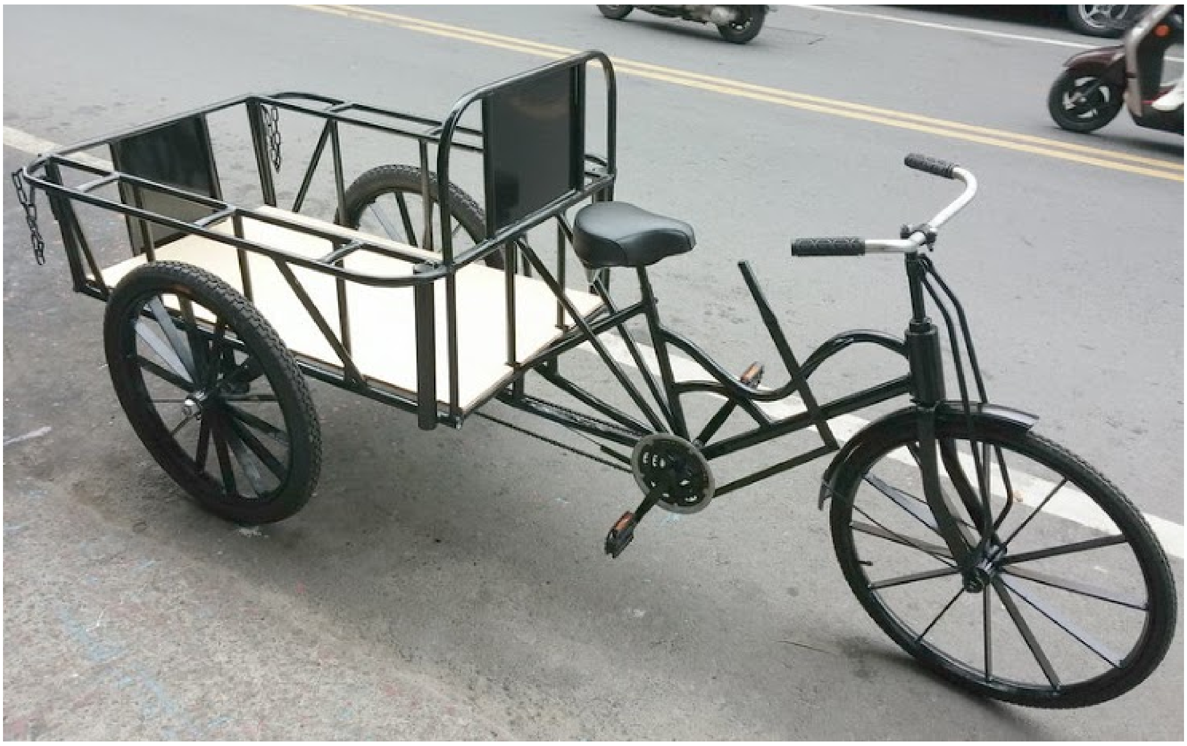

Tricycle

- Strongly disagree
- Disagree
- Slightly disagree
- Neutral
- Slightly agree
- agree
- Strongly agree

2. Please rate your familiarity with this image. *

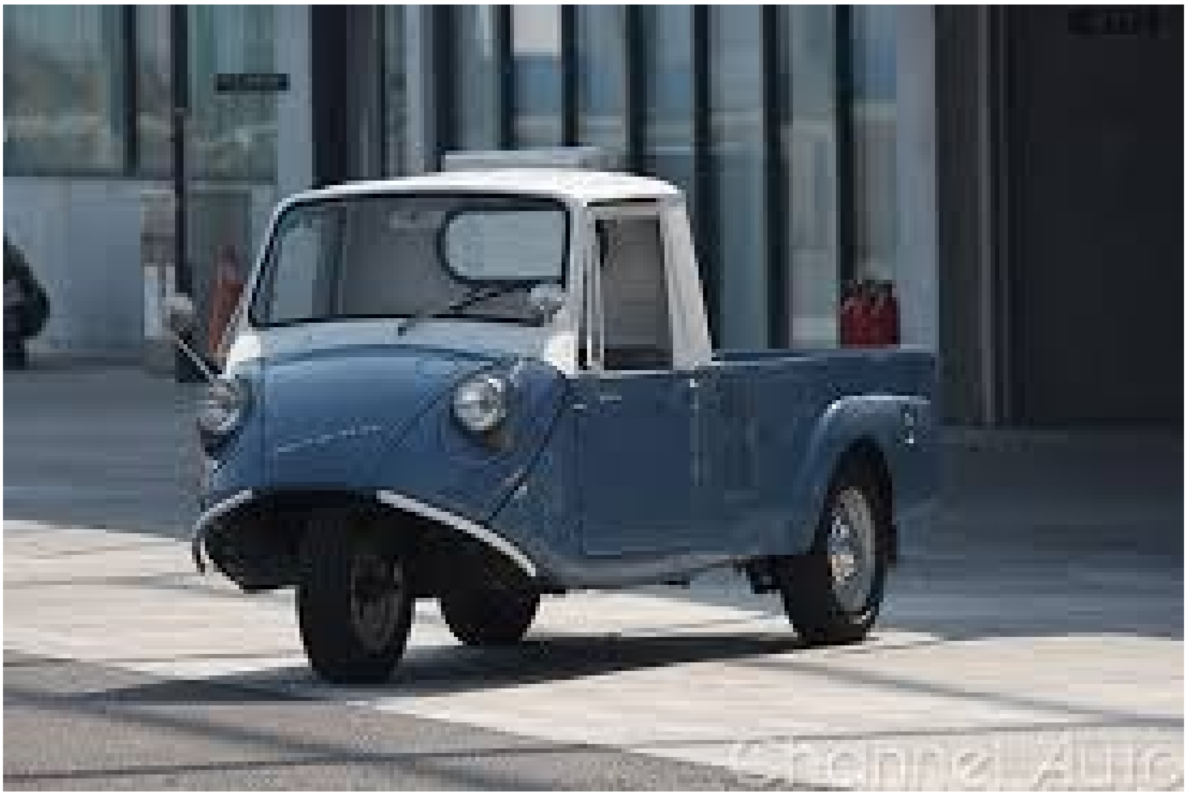

Three-wheeled truck

- Strongly disagree
- Slightly disagree
- Neutral
- Slightly agree
- Agree
- Strongly agree

3. Please rate your familiarity with this image. *

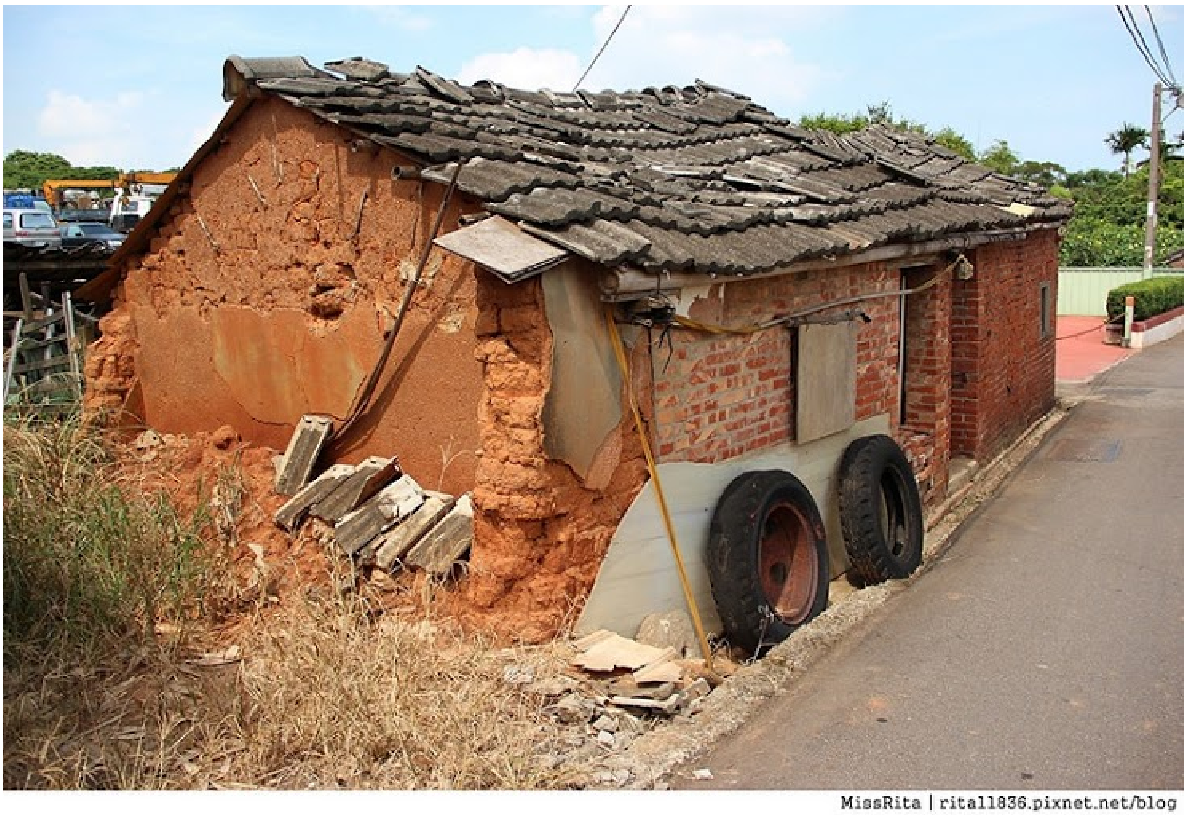

Traditional rammed-earth house

- Strongly disagree
- Disagree
- Slightly disagree
- Neutral
- Slightly agree
- Agree
- Strongly agree

4. Please rate your familiarity with this image. *

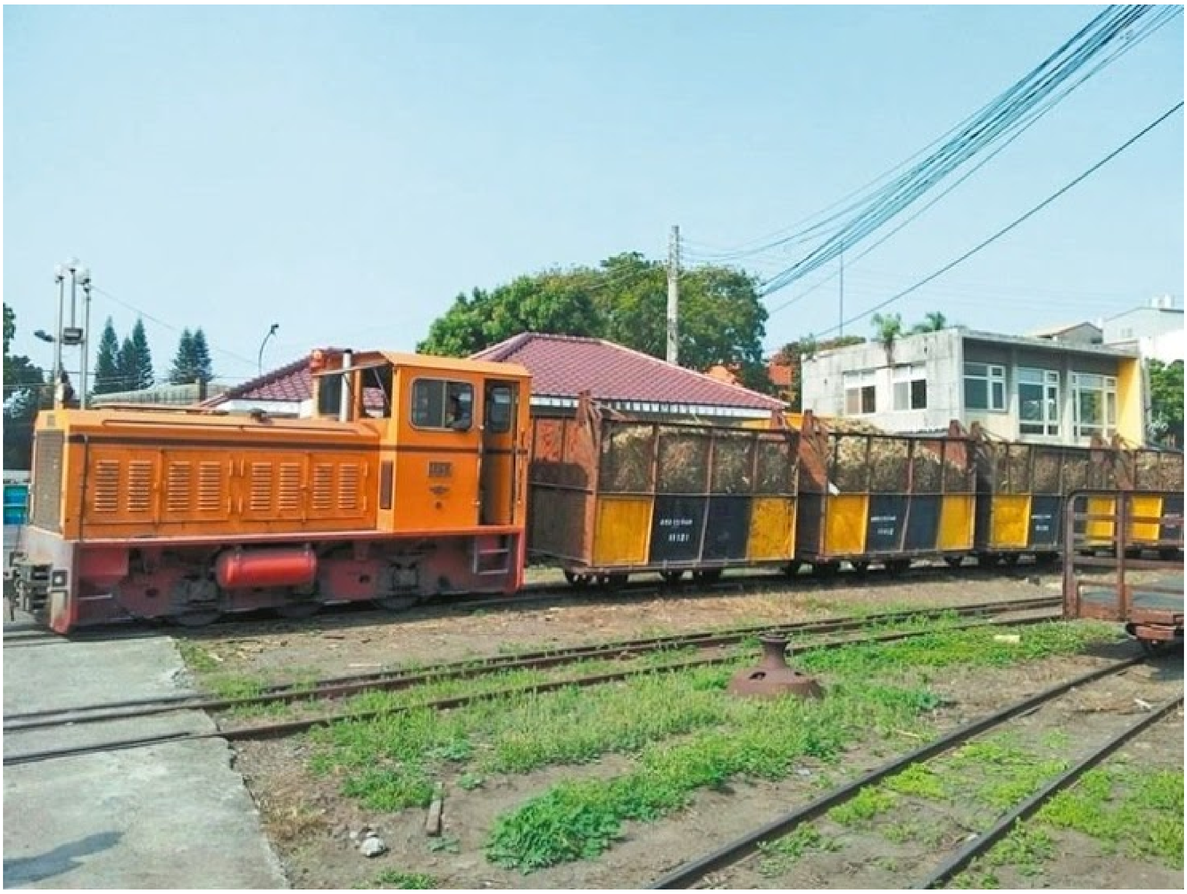

Taiwan Sugar Railways

- Strongly disagree
- Disagree
- Slightly disagree
- Neutral
- Slightly agree
- Agree
- Strongly agree

5. Please rate your familiarity with this image. *

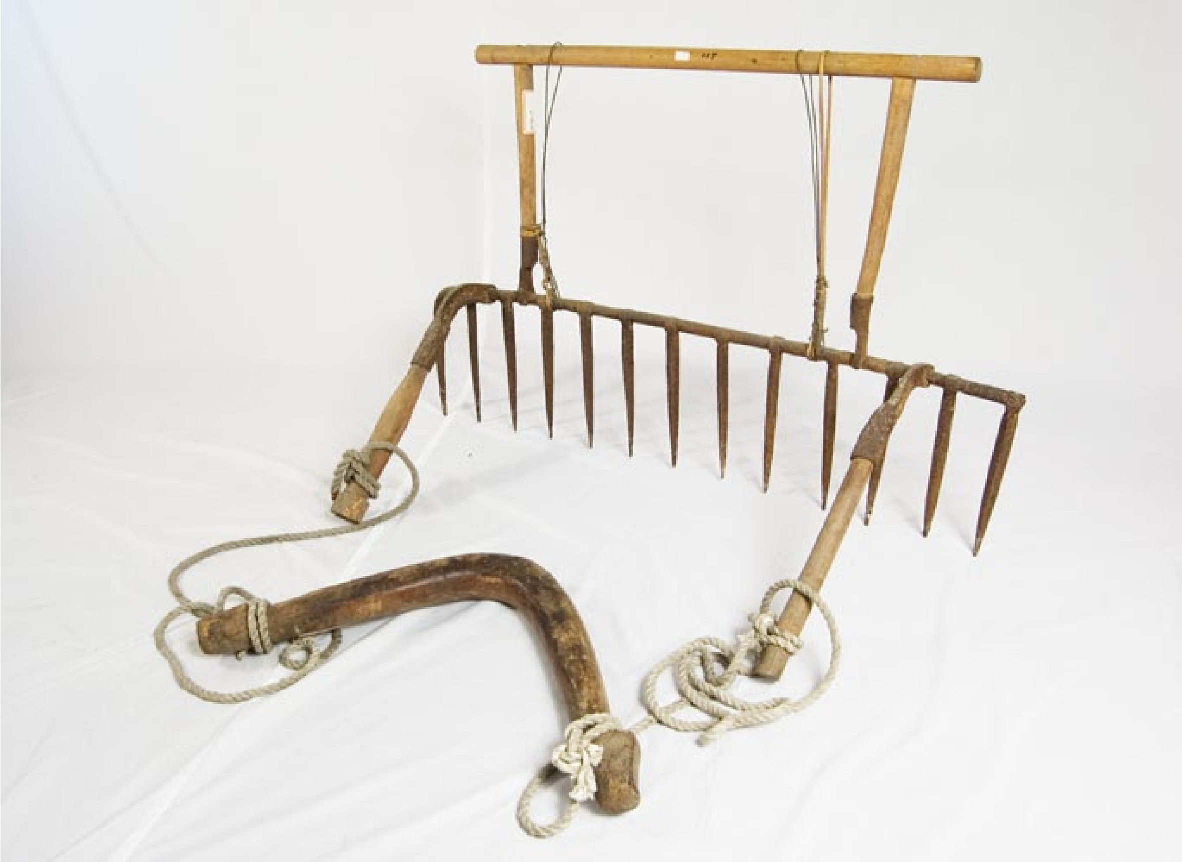

Hand rake

- Strongly disagree
- Disagree
- Slightly disagree
- Neutral
- Slightly agree
- Agree
- Strongly agree

6. Please rate your familiarity with this image. *

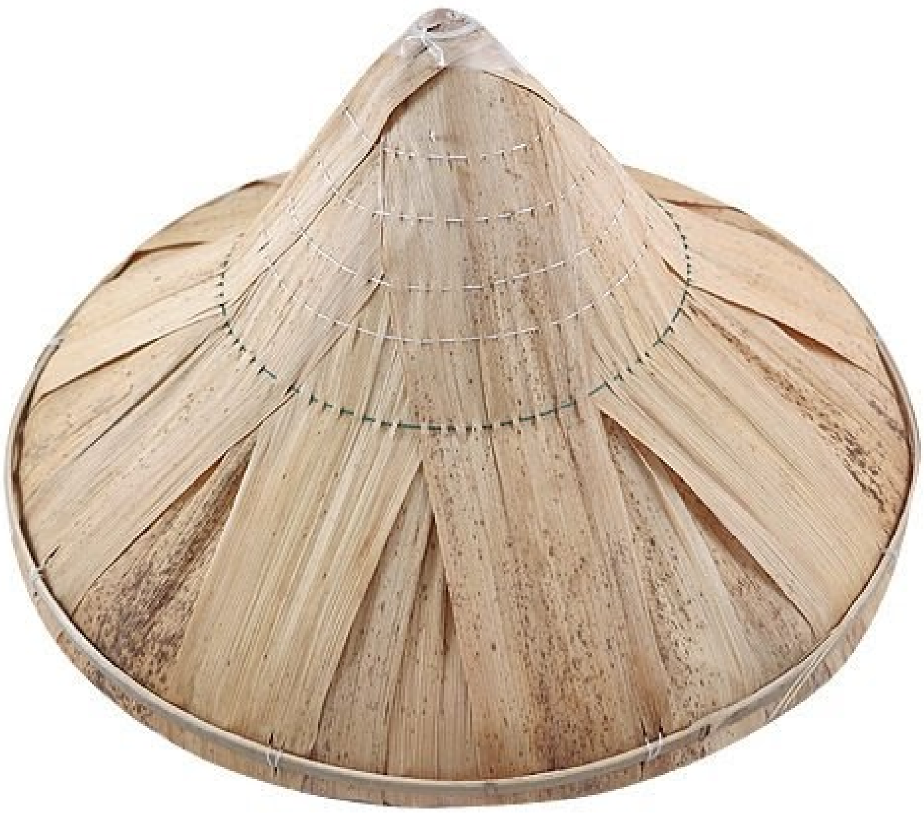

Asian conical hat

- ◯ Strongly disagree
- ◯ Disagree
- ◯ Slightly disagree
- ◯ Neutral
- ◯ Slightly agree
- ◯ Agree
- ◯ Strongly agree

7. Please rate your familiarity with this image. *

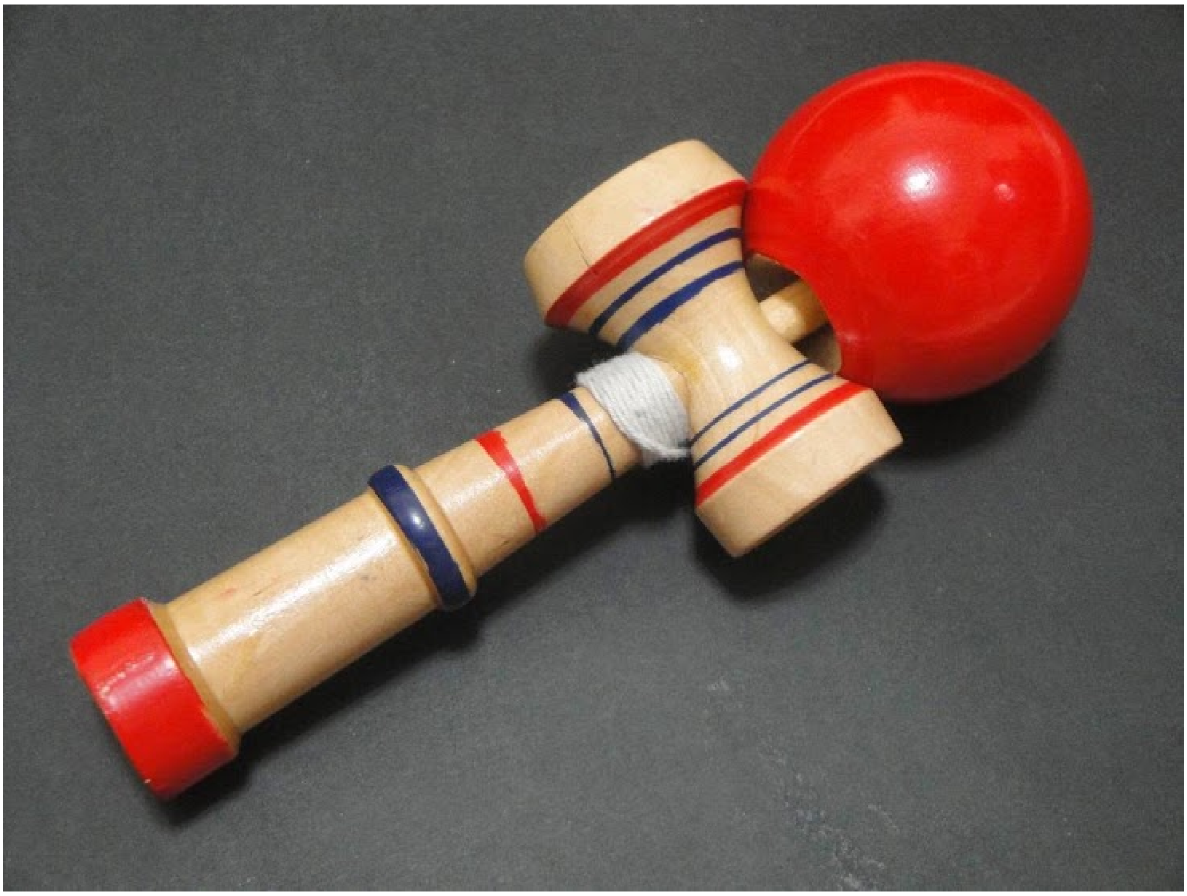

Kendama (Japanese cup-and-ball game)

- Strongly disagree
- Disagree
- Slightly disagree
- Neutral
- Slightly agree
- Agree
- Strongly agree

8. Please rate your familiarity with this image. *

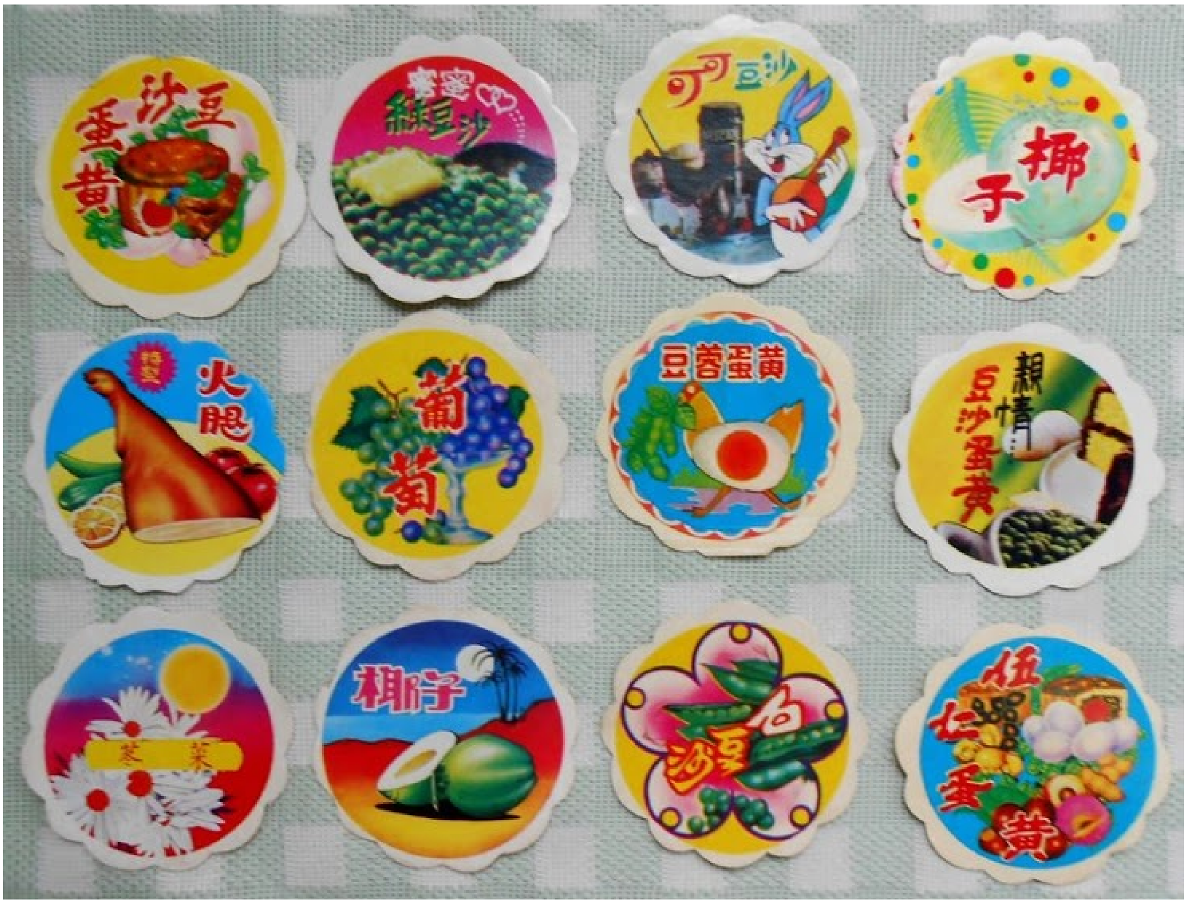

Mooncake labels

- Strongly disagree
- Disagree
- Slightly disagree
- Neutral
- Slightly agree
- Agree
- Strongly agree

9. Please rate your familiarity with this image. *

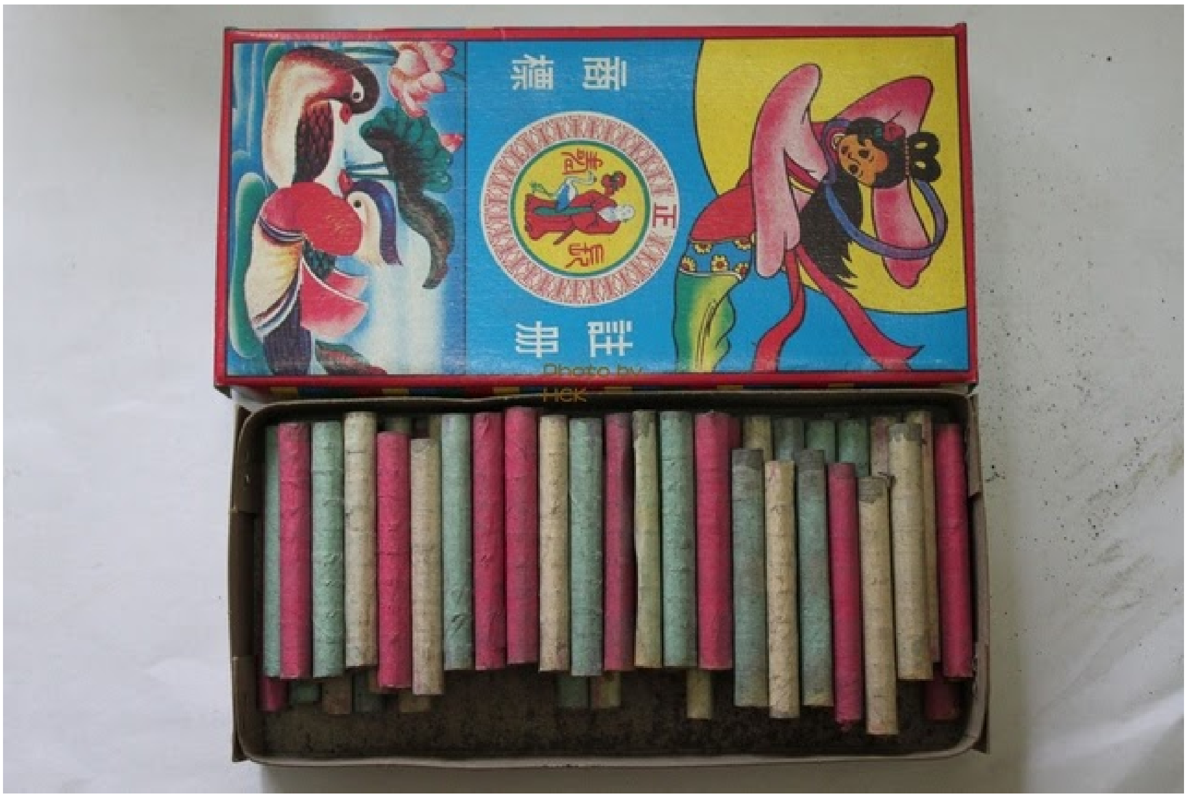

Firecracker

- Strongly disagree
- Disagree
- Slightly disagree
- Neutral
- Slightly agree
- Agree
- Strongly agree

10. Please rate your familiarity with this image. *

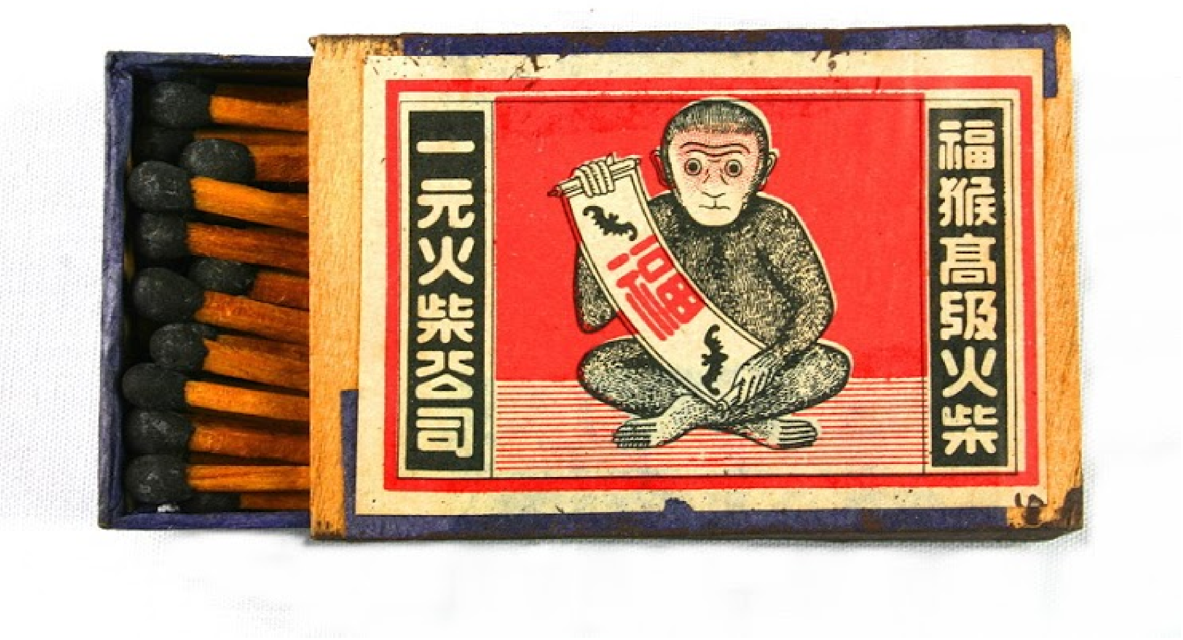

Matchbox

- Strongly disagree
- Disagree
- Slightly disagree
- Neutral
- Slightly agree
- Agree
- Strongly agree

11. Please rate your familiarity with this image. *

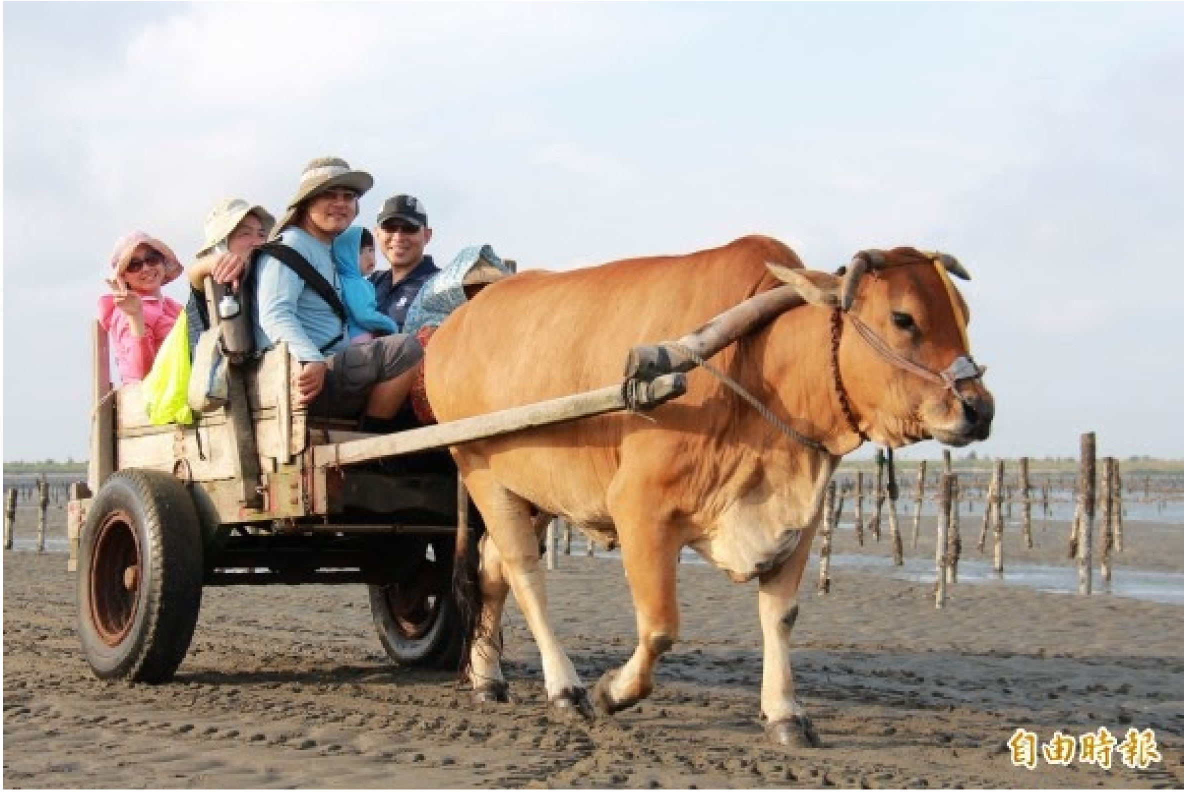

Ox cart

- Strongly disagree
- Disagree
- Slightly disagree
- Neutral
- Slightly agree
- Agree
- Strongly agree

12. Please rate your familiarity with this image. *

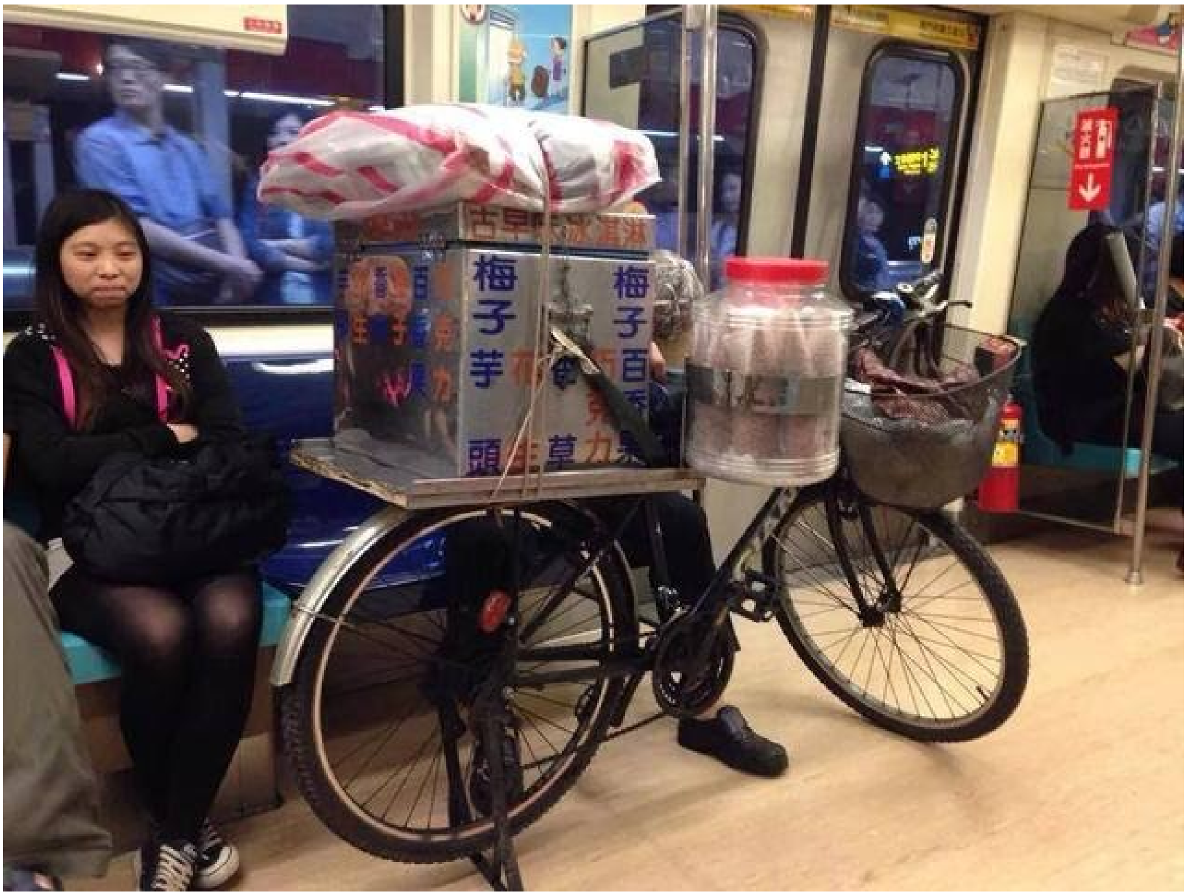

Ice cream cart

- ◯ Strongly disagree
- ◯ Disagree
- ◯ Slightly disagree
- ◯ Neutral
- ◯ Slightly agree
- ◯ Agree
- ◯ Strongly agree

13. Please rate your familiarity with this image. *

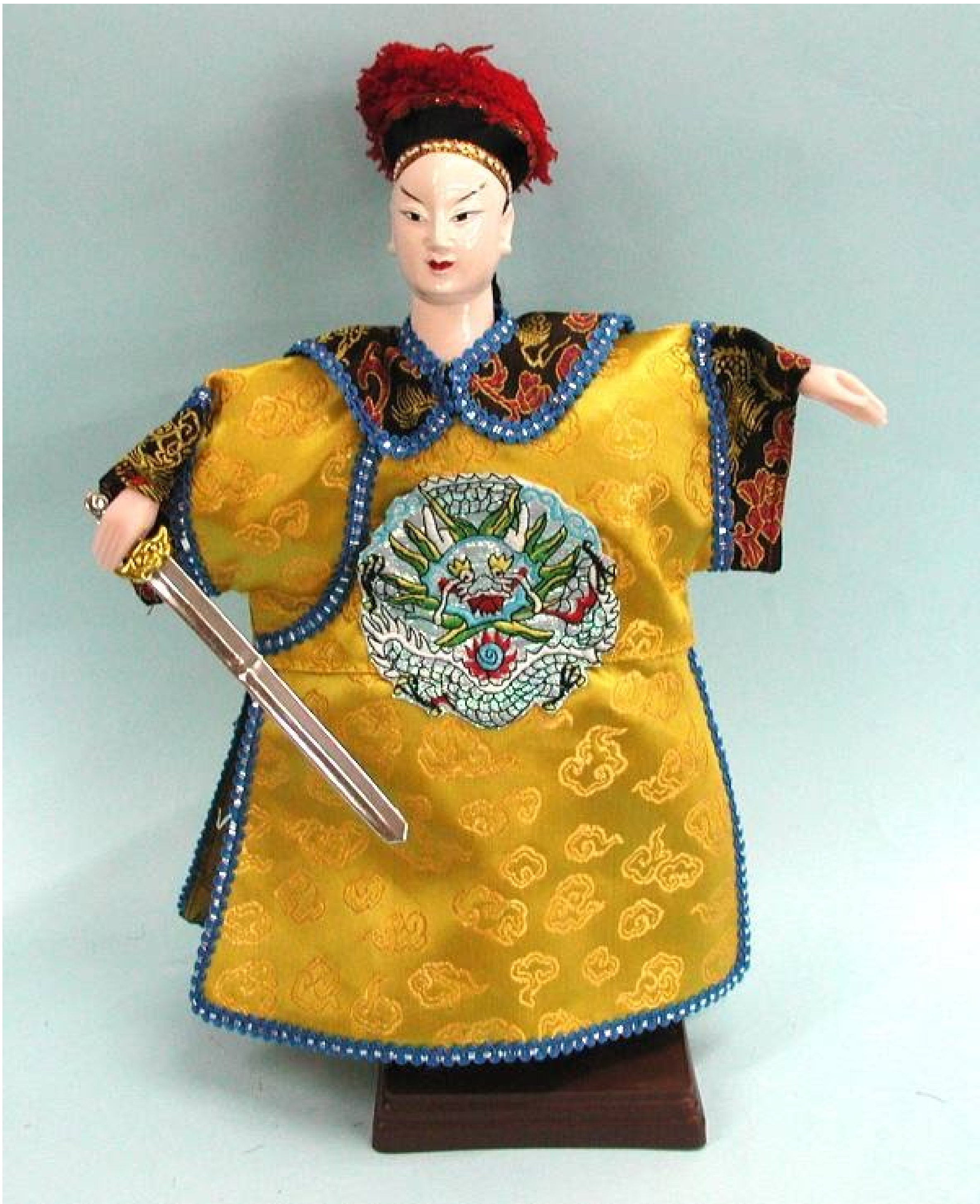

Glove puppetry puppet

- Strongly disagree
- Disagree
- Slightly disagree
- Neutral
- Slightly agree
- Agree

Agree

Strongly agree

14. Please rate your familiarity with this image. *

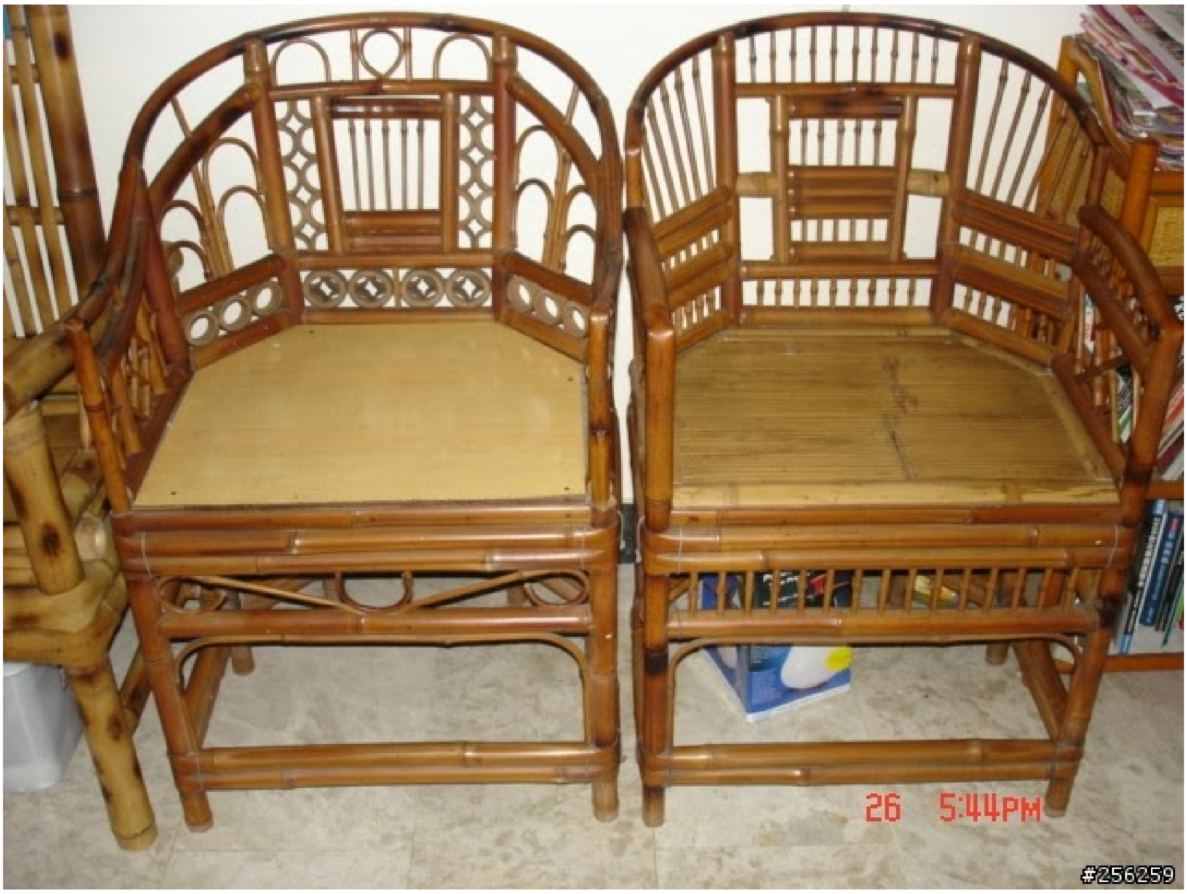

Bamboo chairs

Strongly disagree

Disagree

Slightly disagree

Neutral

Slightly agree

Agree

Strongly agree

15. Please rate your familiarity with this image. *

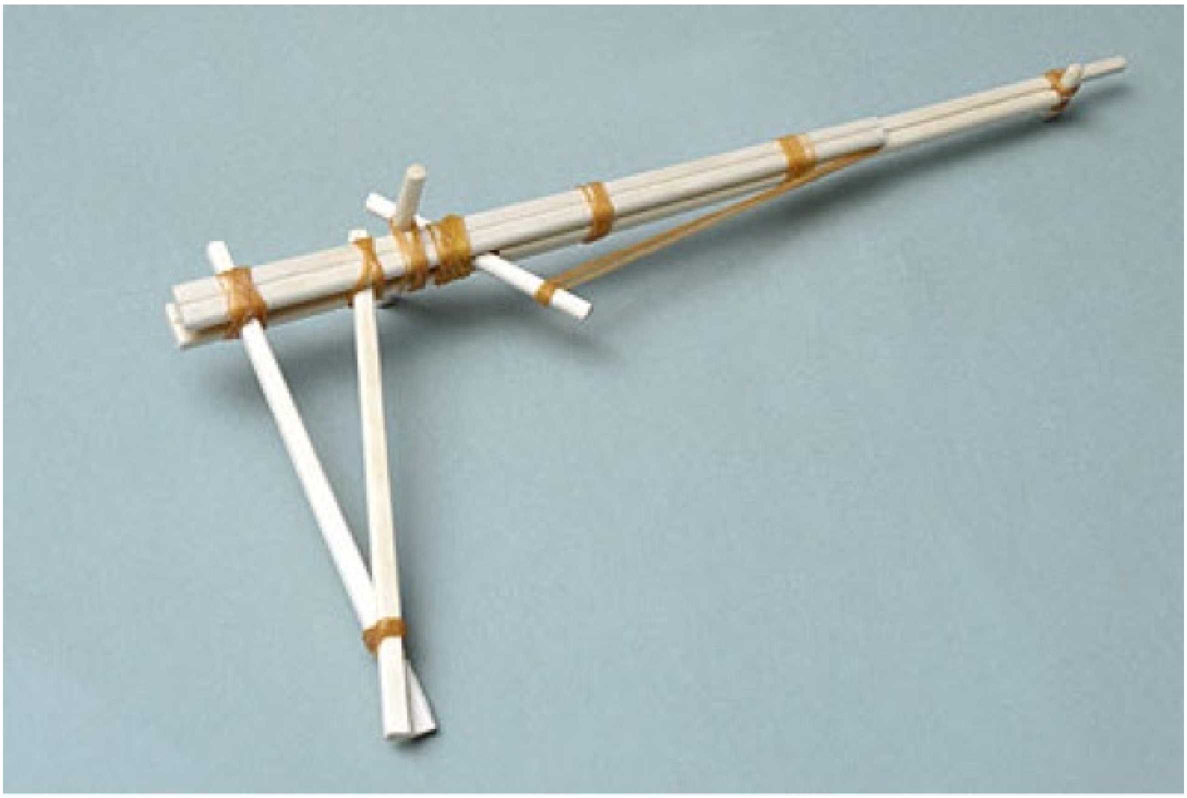

Bamboo toy gun

- Strongly disagree
- Disagree
- Slightly disagree
- Neutral
- Slightly agree
- Agree
- Strongly agree

16. 15. Please rate your familiarity with this image. *

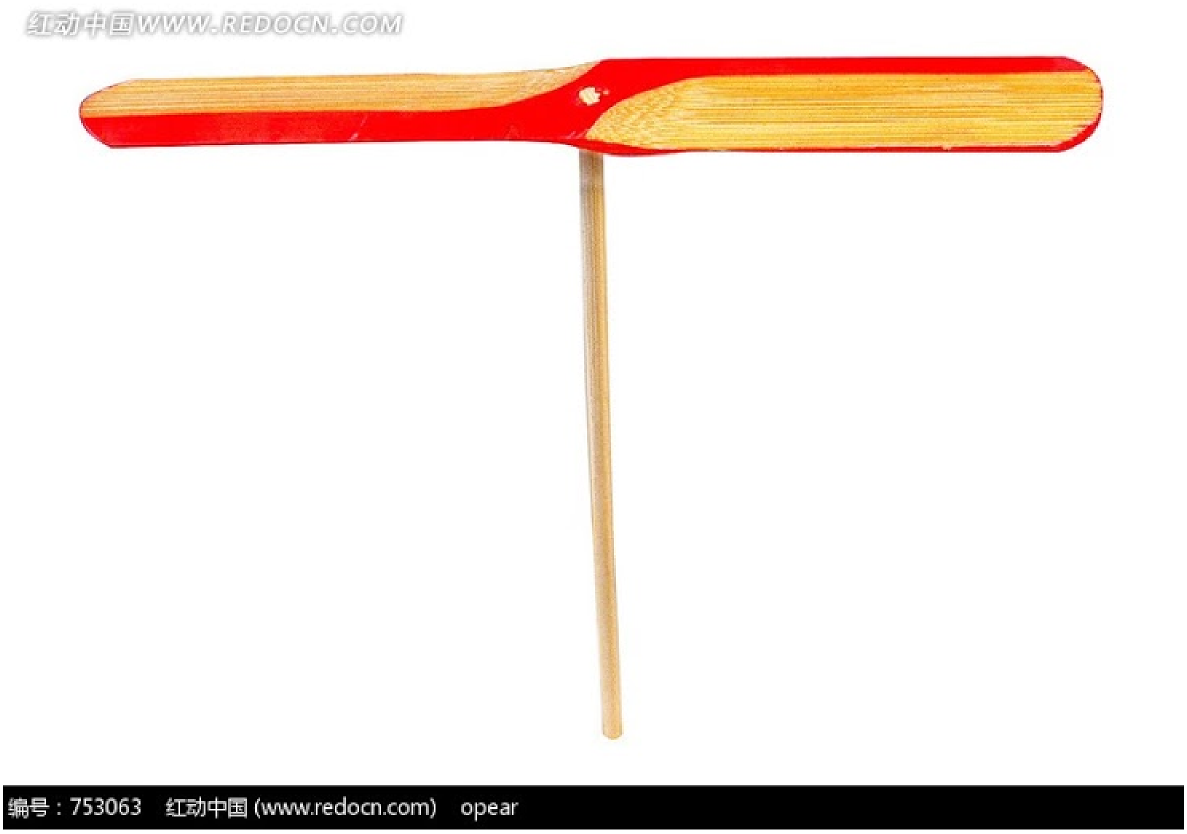

Bamboo-copter

- Strongly disagree
- Disagree
- Slightly disagree
- Neutral
- Slightly agree
- Agree
- Strongly agree

17. Please rate your familiarity with this image. *

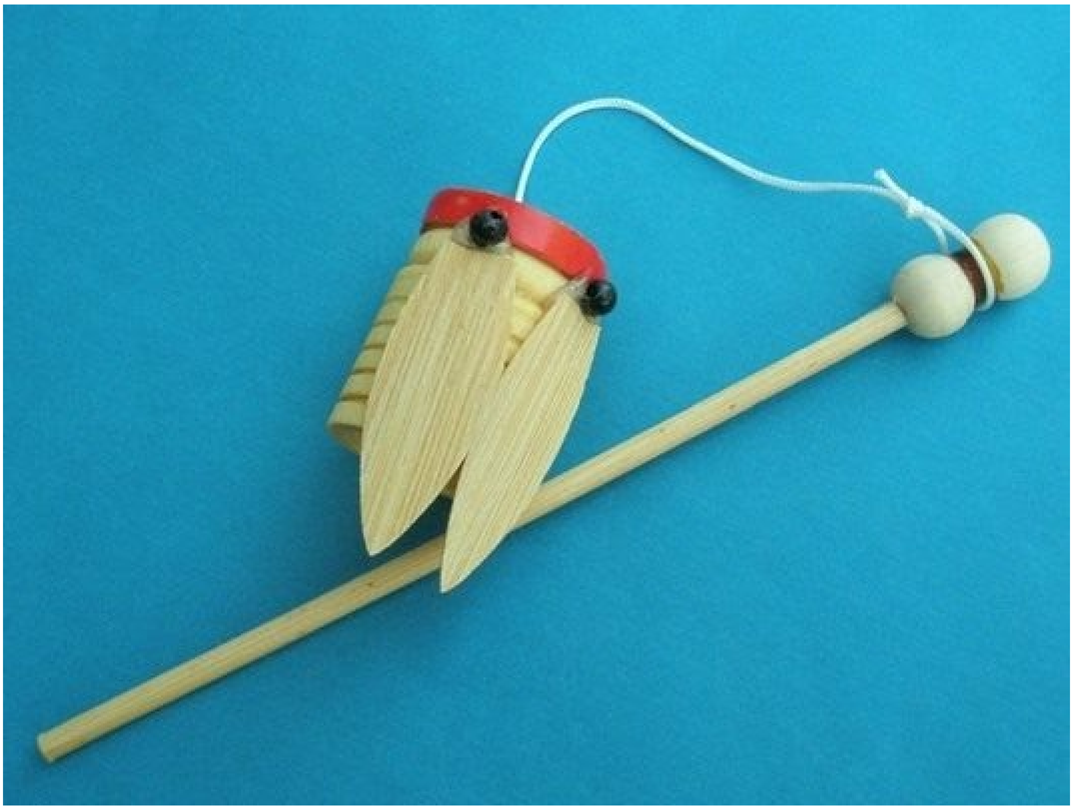

Bamboo ciacda toy

- Strongly disagree
- Disagree
- Slightly disagree
- Neutral
- Slightly agree
- Agree
- Strongly agree

18. Please rate your familiarity with this image. *

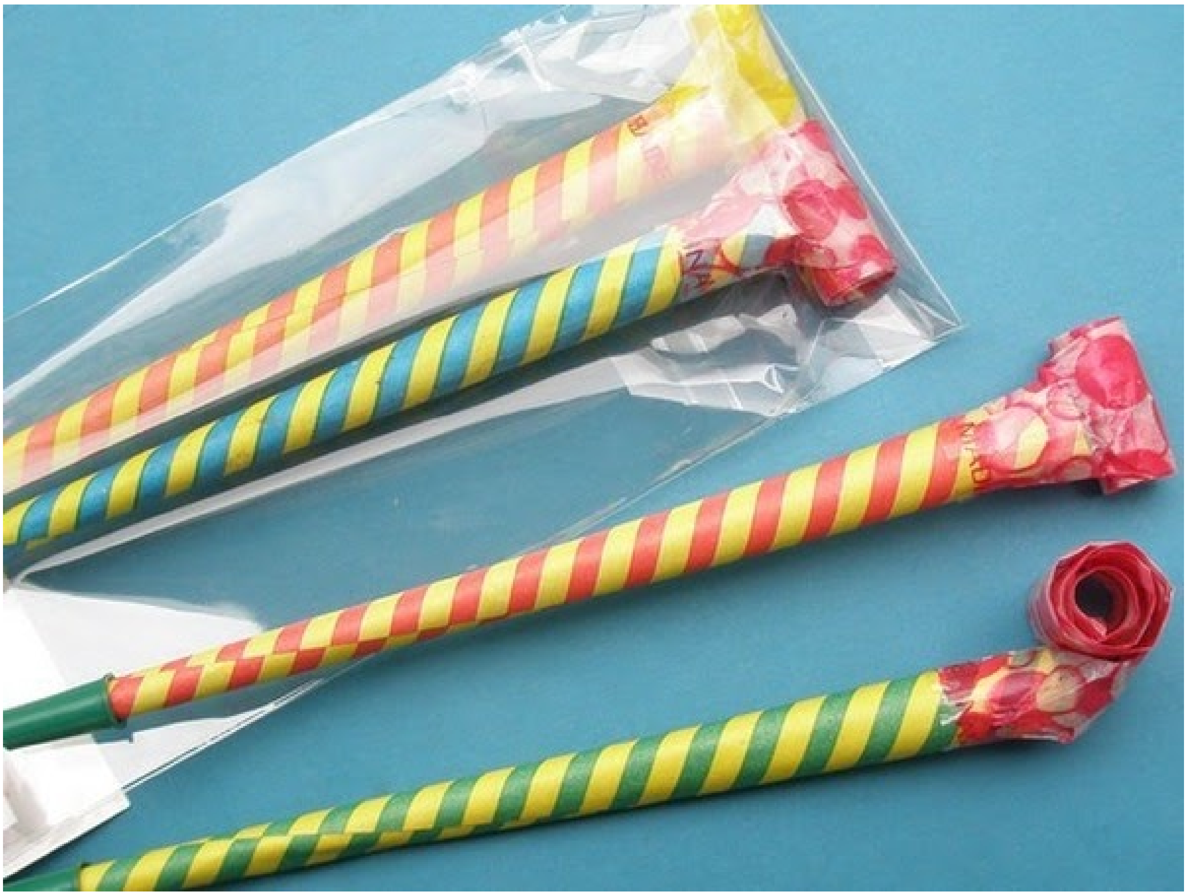

Party blowouts

- Strongly disagree
- Disagree
- Slightly disagree
- Neutral
- Slightly agree
- Agree
- Strongly agree

19. Please rate your familiarity with this image. *

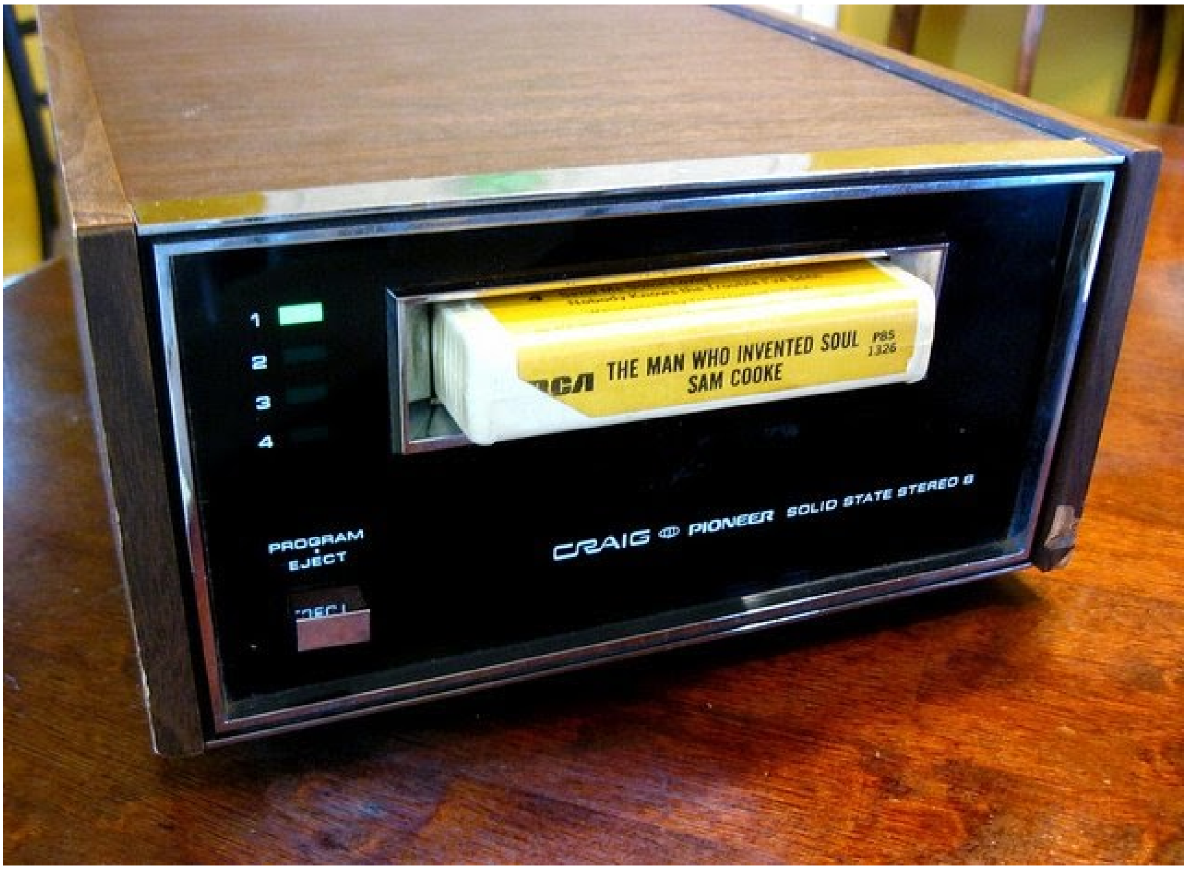

Cassette tapes

- Strongly disagree
- Disagree
- Slightly disagree
- Neutral
- Slightly agree
- Agree
- Strongly agree

20. Please rate your familiarity with this image. *

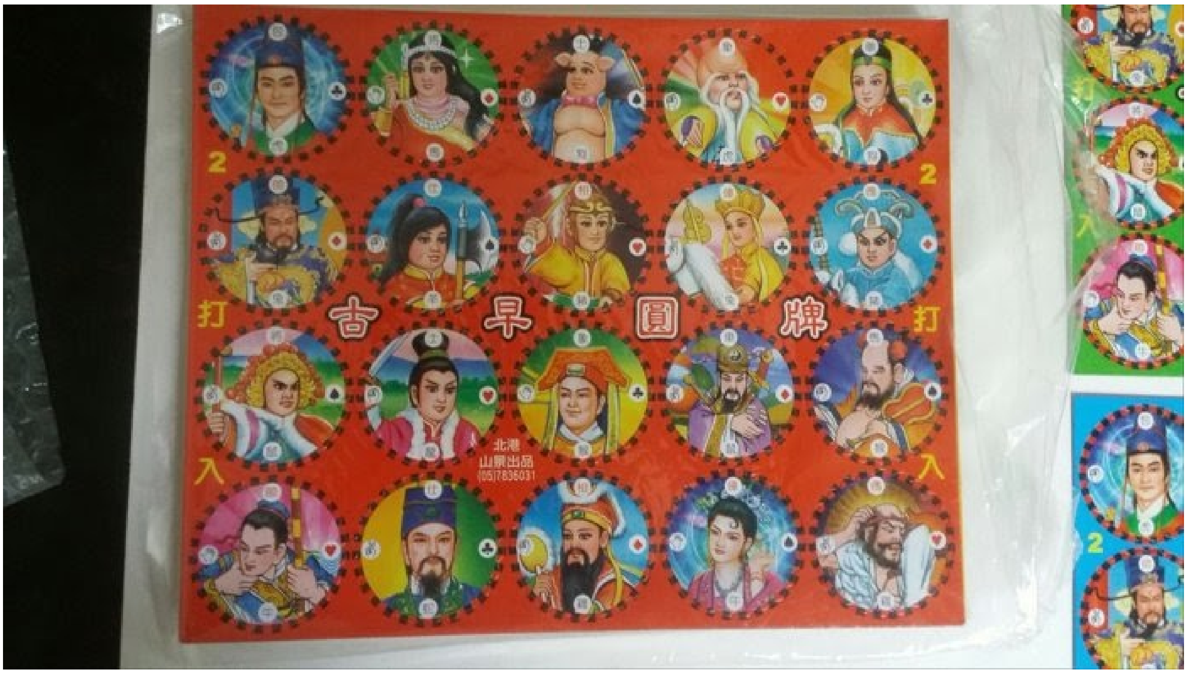

Milk caps (game)

- ◯ Strongly disagree
- ◯ Disagree
- ◯ Slightly disagree
- ◯ Neutral
- ◯ Slightly agree
- ◯ Agree
- ◯ Stronly agree

21. Please rate your familiarity with this image. *

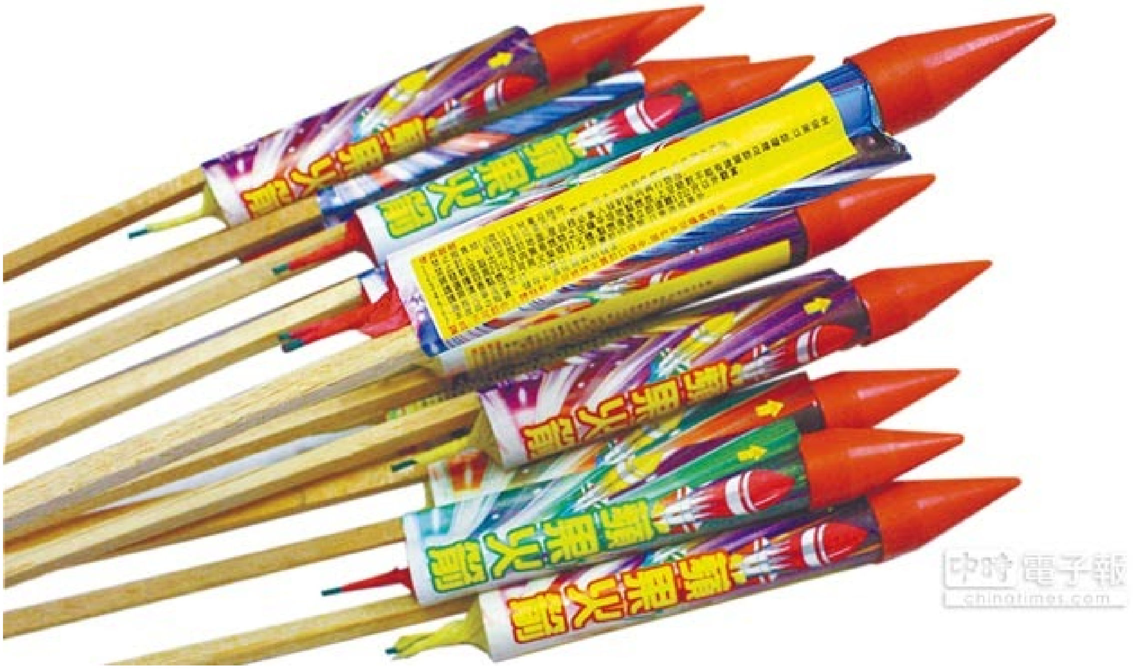

Skyrocket

- Strongly disagree
- Disagree
- Slightly disagree
- Neutral
- Slightly agree
- Agree
- Strongly agree

22. Please rate your familiarity with this image. *

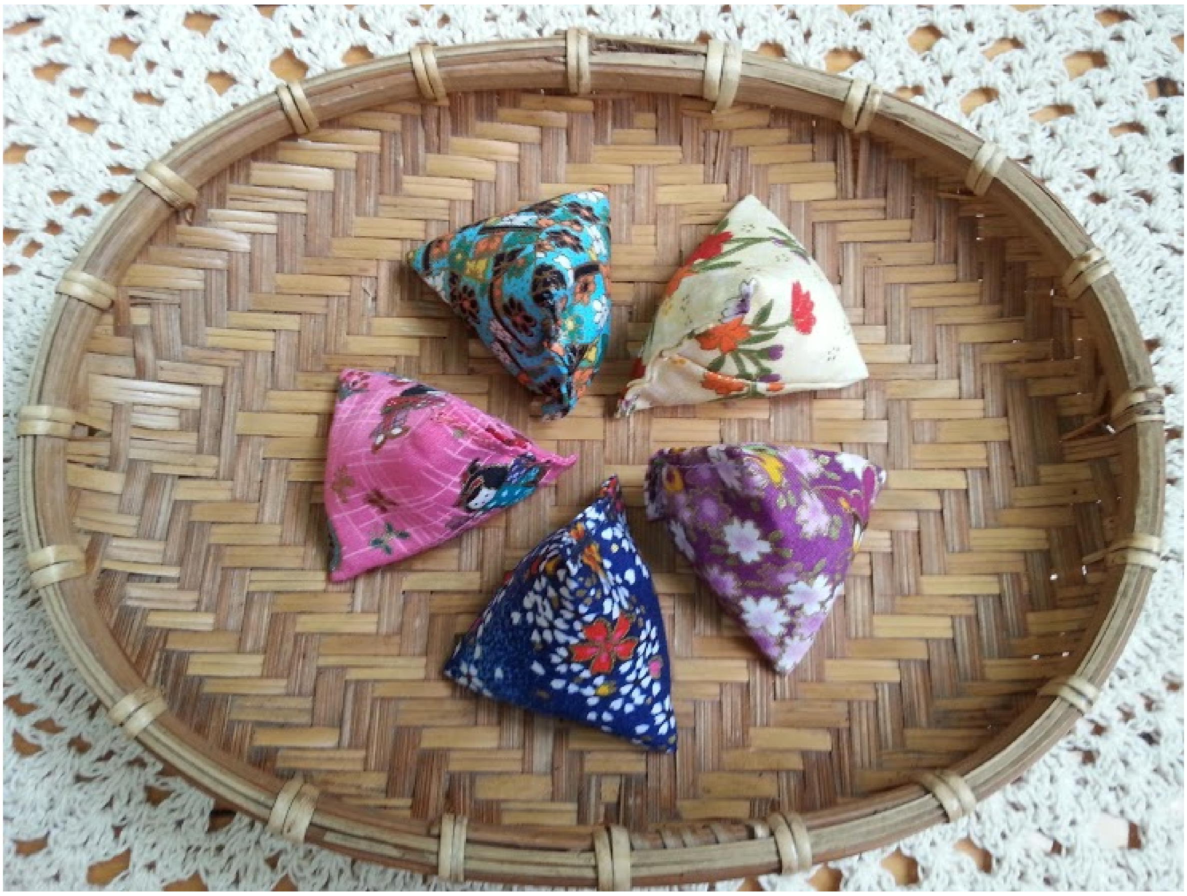

Toy sandbags

- Strongly disagree
- Disagree
- Slightly disagree
- Neutral
- Slightly agree
- Agree
- Strongly agree

23. Please rate your familiarity with this image. *

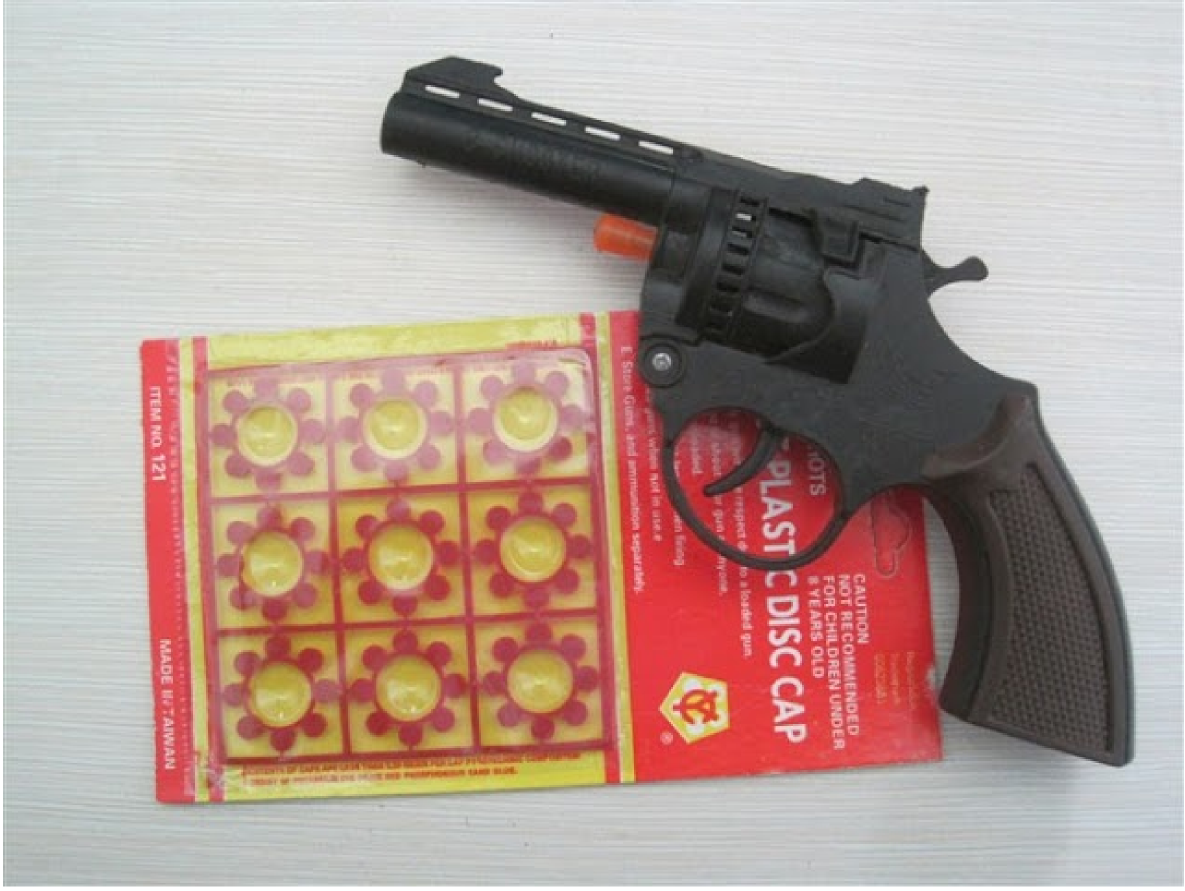

BB guns

- Strongly disagree
- Disagree
- Slightly disagree
- Neutral
- Slightly agree
- Agree
- Strongly agree

24. Please rate your familiarity with this image. *

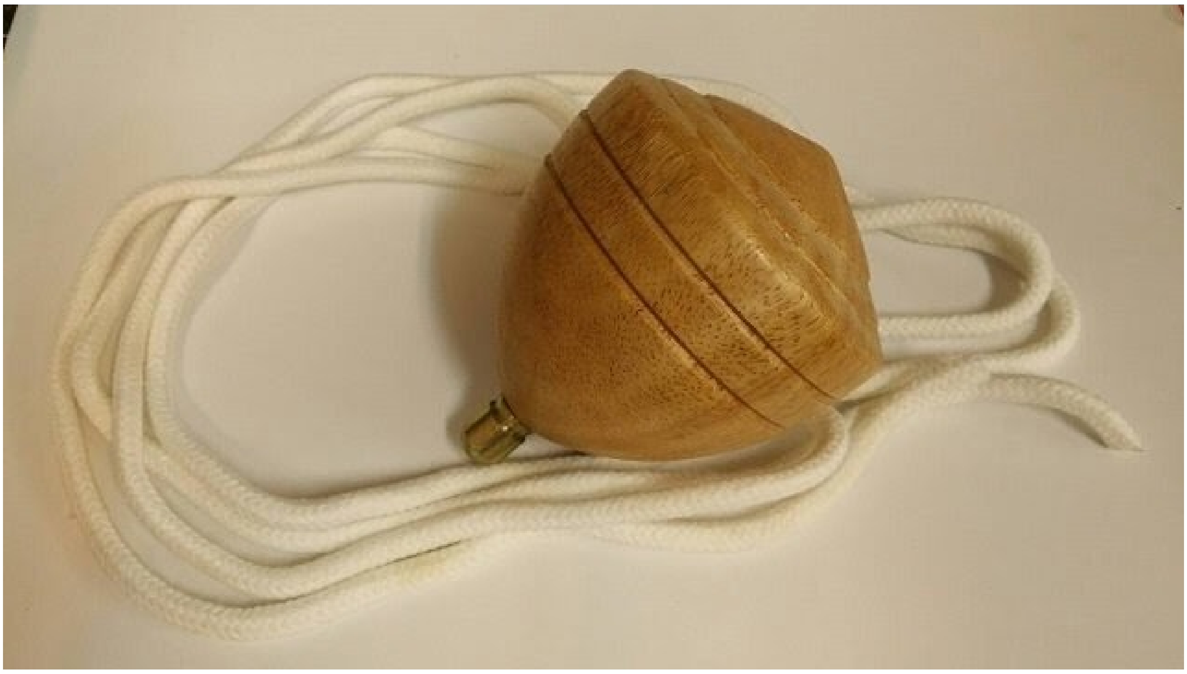

Spinning top

- Strongly disagree
- Disagree
- Slightly disagree
- Neutral
- Slightly agree
- Agree
- Strongly agree

25. Please rate your familiarity with this image. *

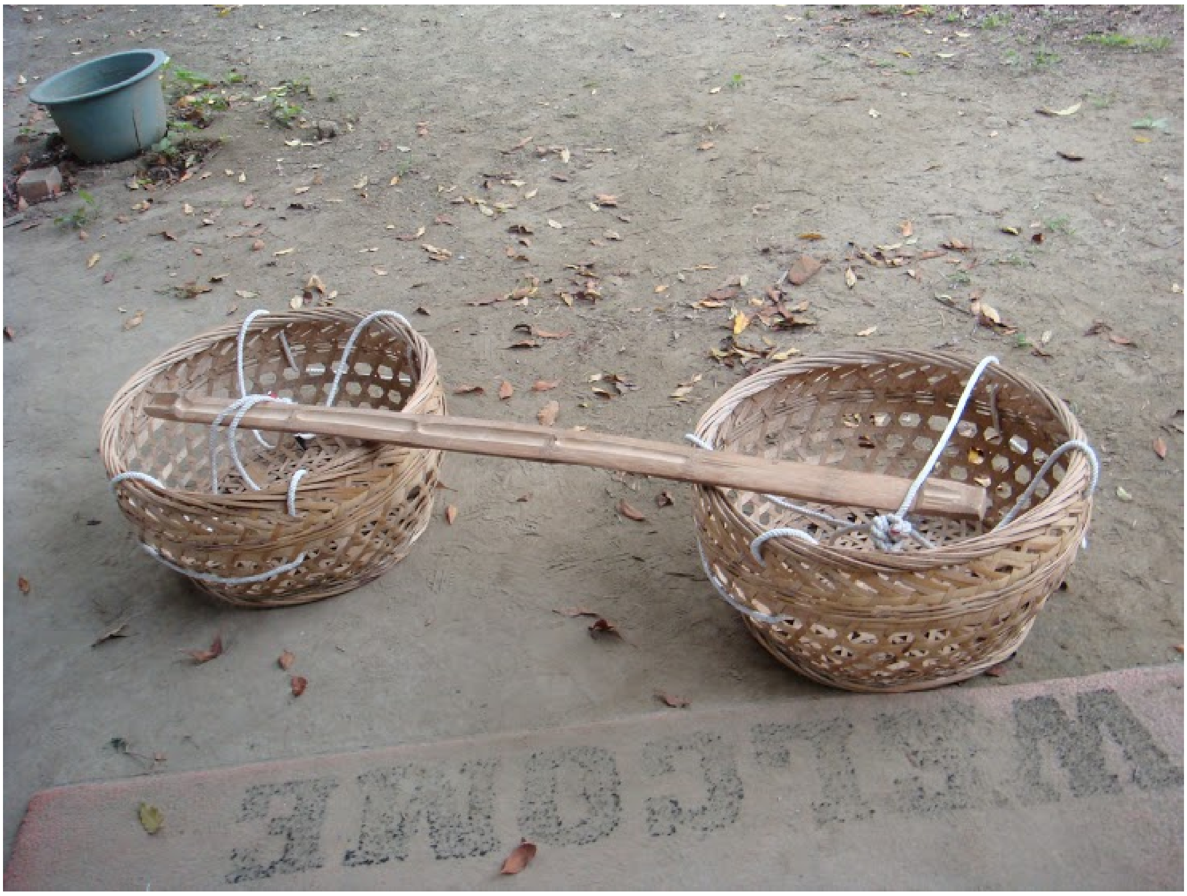

Shoulder pole

- ◯ Strongly disagree
- ◯ Disagree
- ◯ Slightly disagree
- ◯ Neutral
- ◯ Slightly agree
- ◯ Agree
- ◯ Strongly agree

26. Please rate your familiarity with this image. *

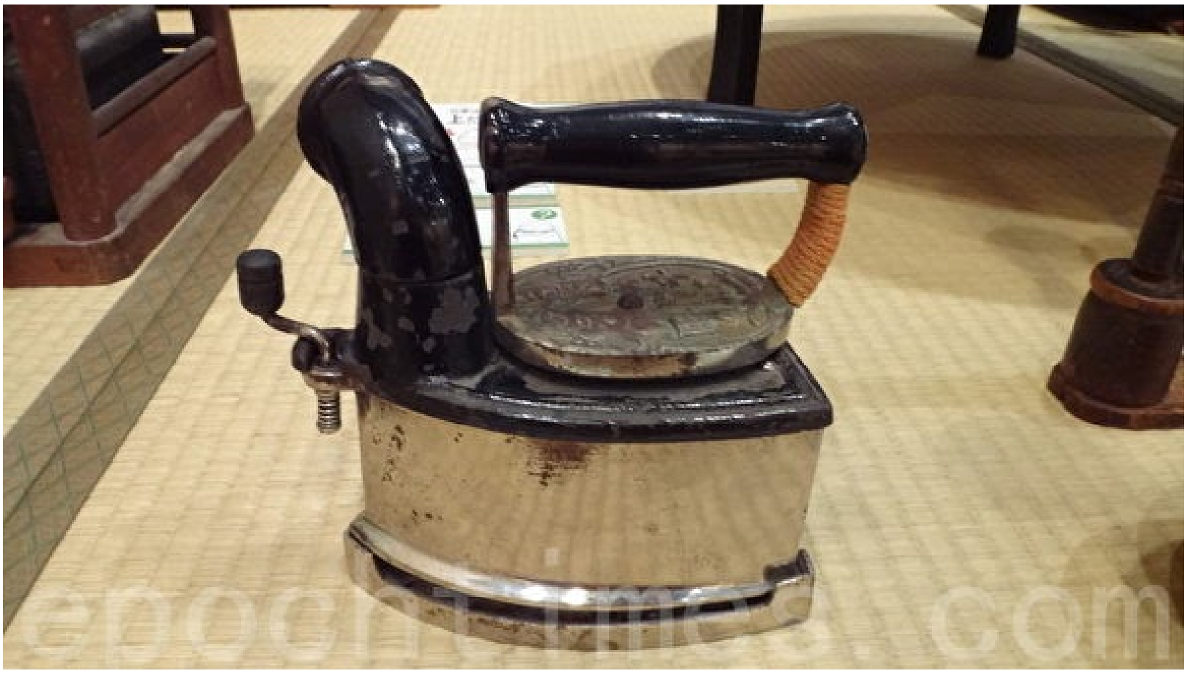

Charcoal iron

- Strongly disagree
- Disagree
- Slightly disagree
- Neutral
- Slightly agree
- Agree
- Strongly agree

27. Please rate your familiarity with this image. *

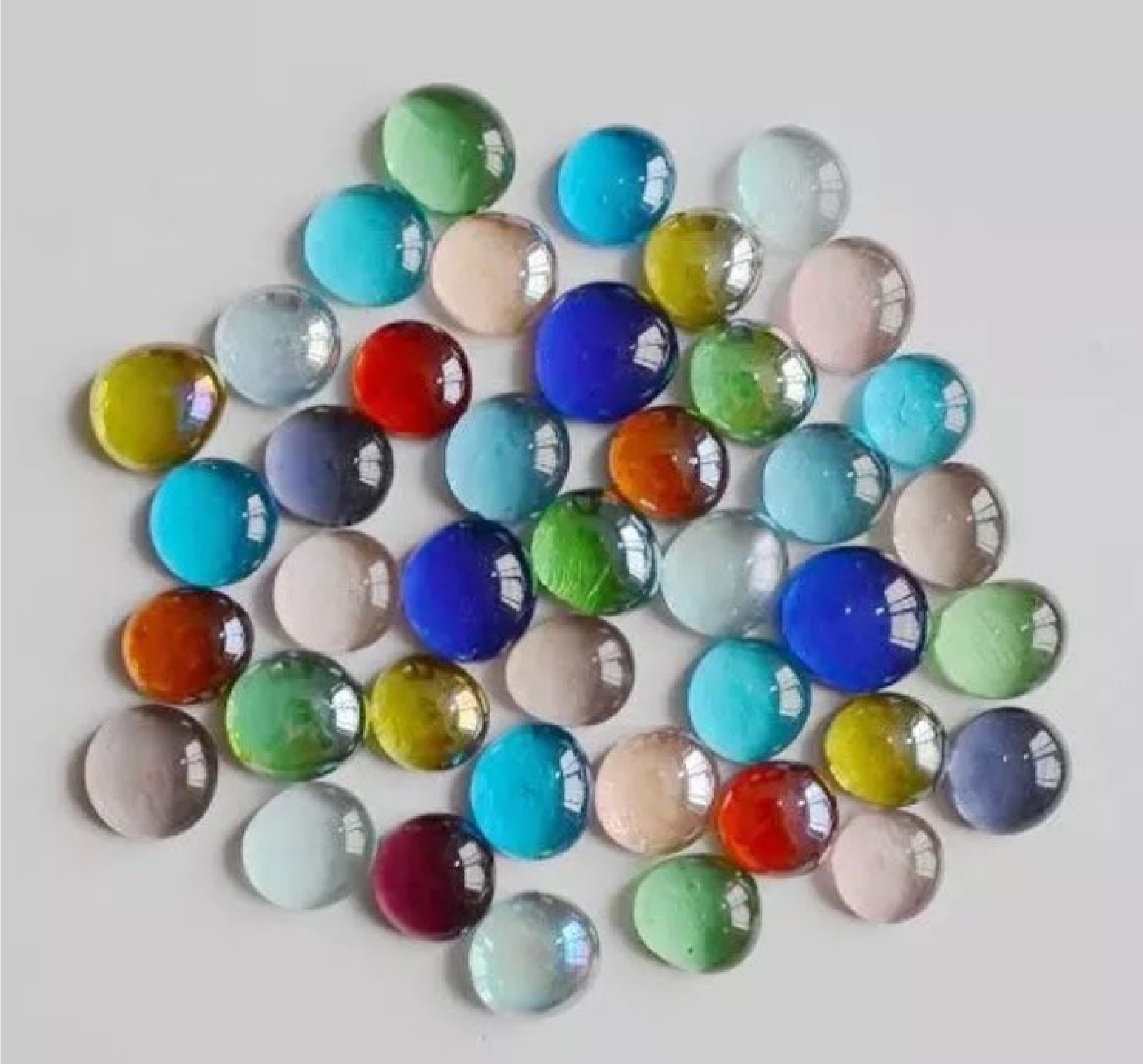

Glass gems

- Strongly disagree
- Disagree
- Slightly disagree
- Neutral
- Slightly agree
- Agree
- Strongly agree

28. Please rate your familiarity with this image. *

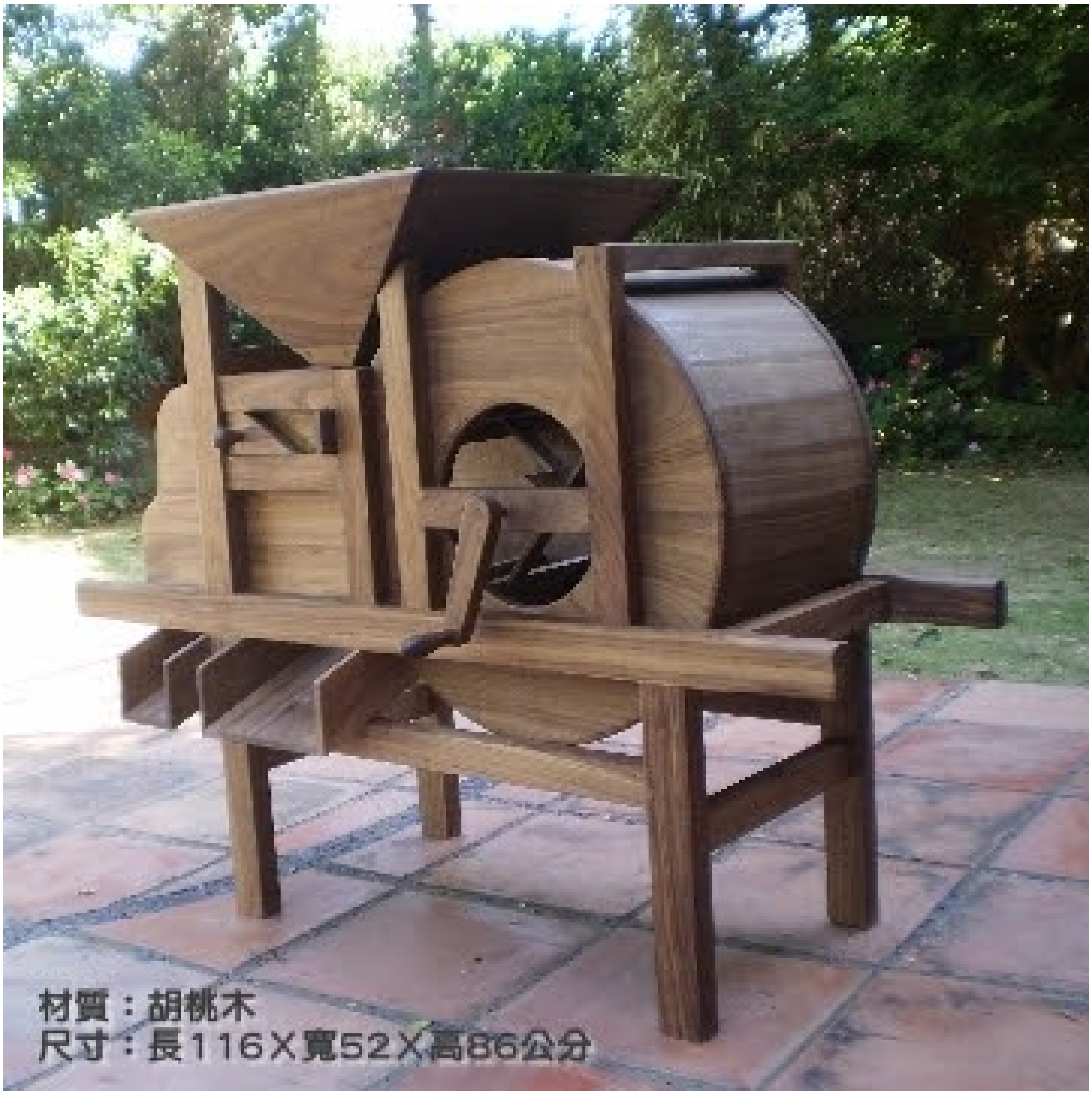

Winnowing machine

- ◯ Strongly disagree
- ◯ Disagree
- ◯ Slightly disagree
- ◯ Neutral
- ◯ Slightly agree
- ◯ Agree
- ◯ Strongly agree

29. Please rate your familiarity with this image. *

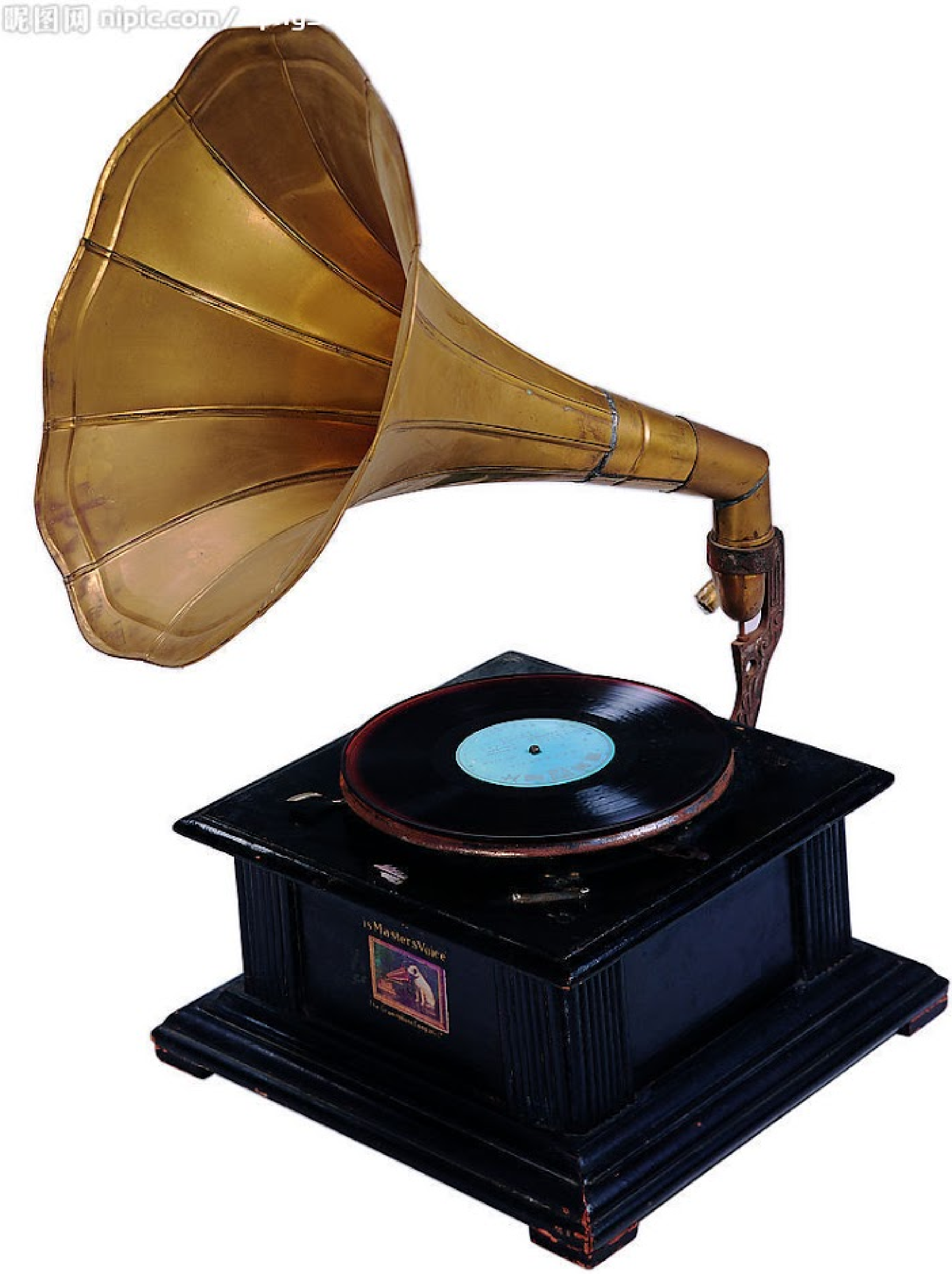

gramophone

- Strongly disagree
- Disagree
- Slightly disagree
- Neutral

Slightly agree

Agree

Strongly agree

30. Please rate your familiarity with this image. *

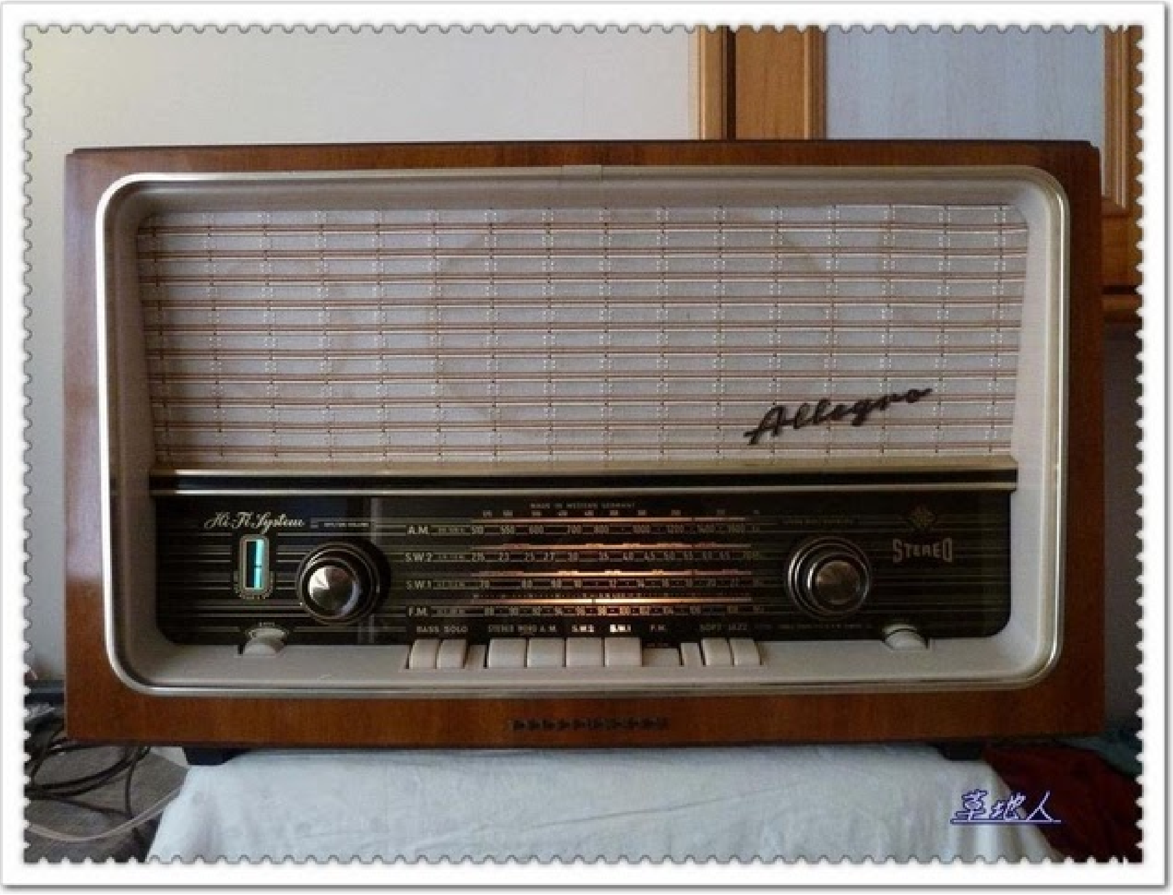

Vacuum tube radio

Strongly disagree

Disagree

Slightly disagree

Neutral

Slightly agree

Agree

Strongly agree

31. Please rate your familiarity with this image. *

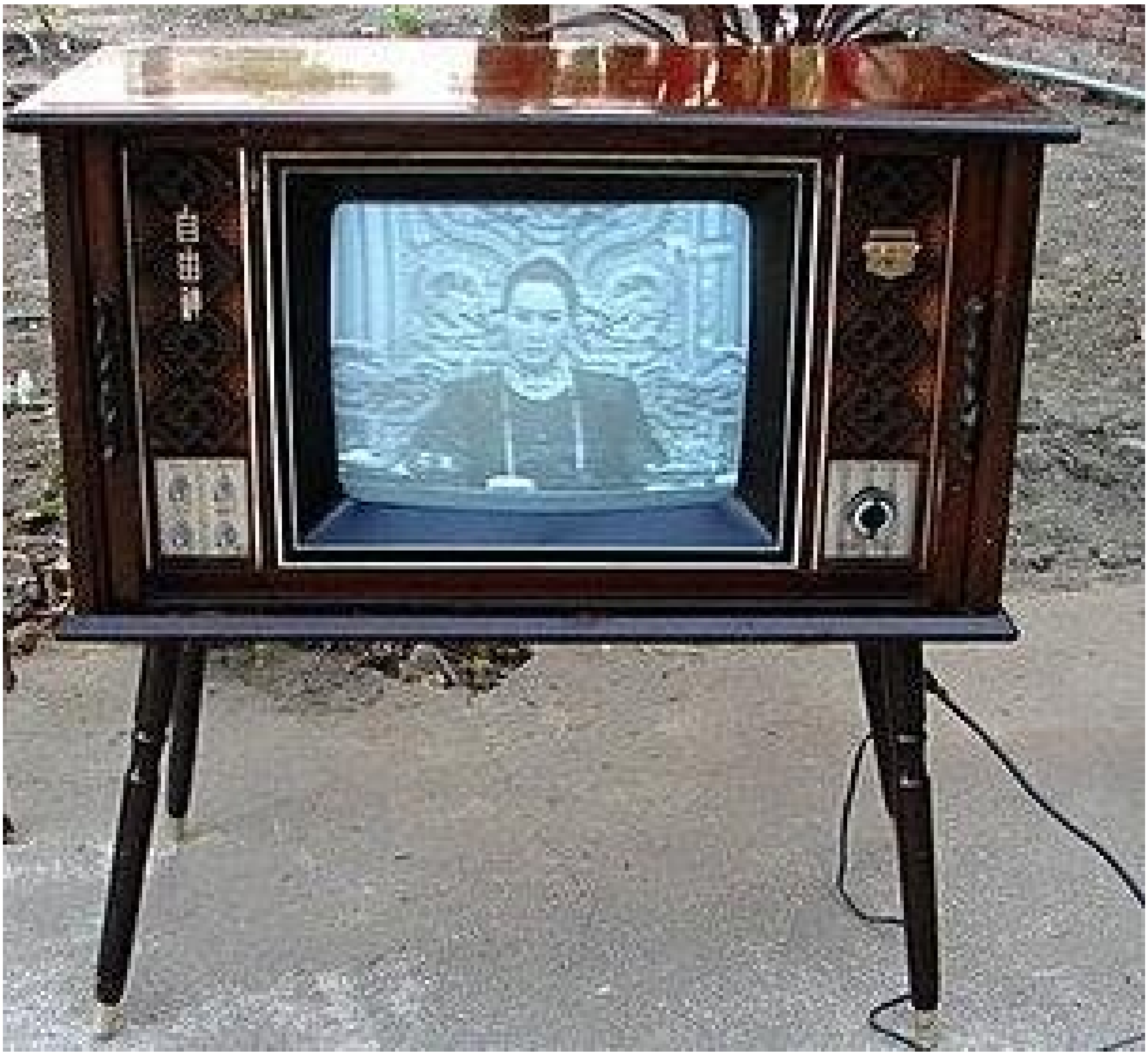

Vacuum tube TV

- Strongly disagree
- Disagree
- Slightly disagree
- Neutral
- Slightly agree
- Agree
- Strongly agree

32. Please rate your familiarity with this image. *

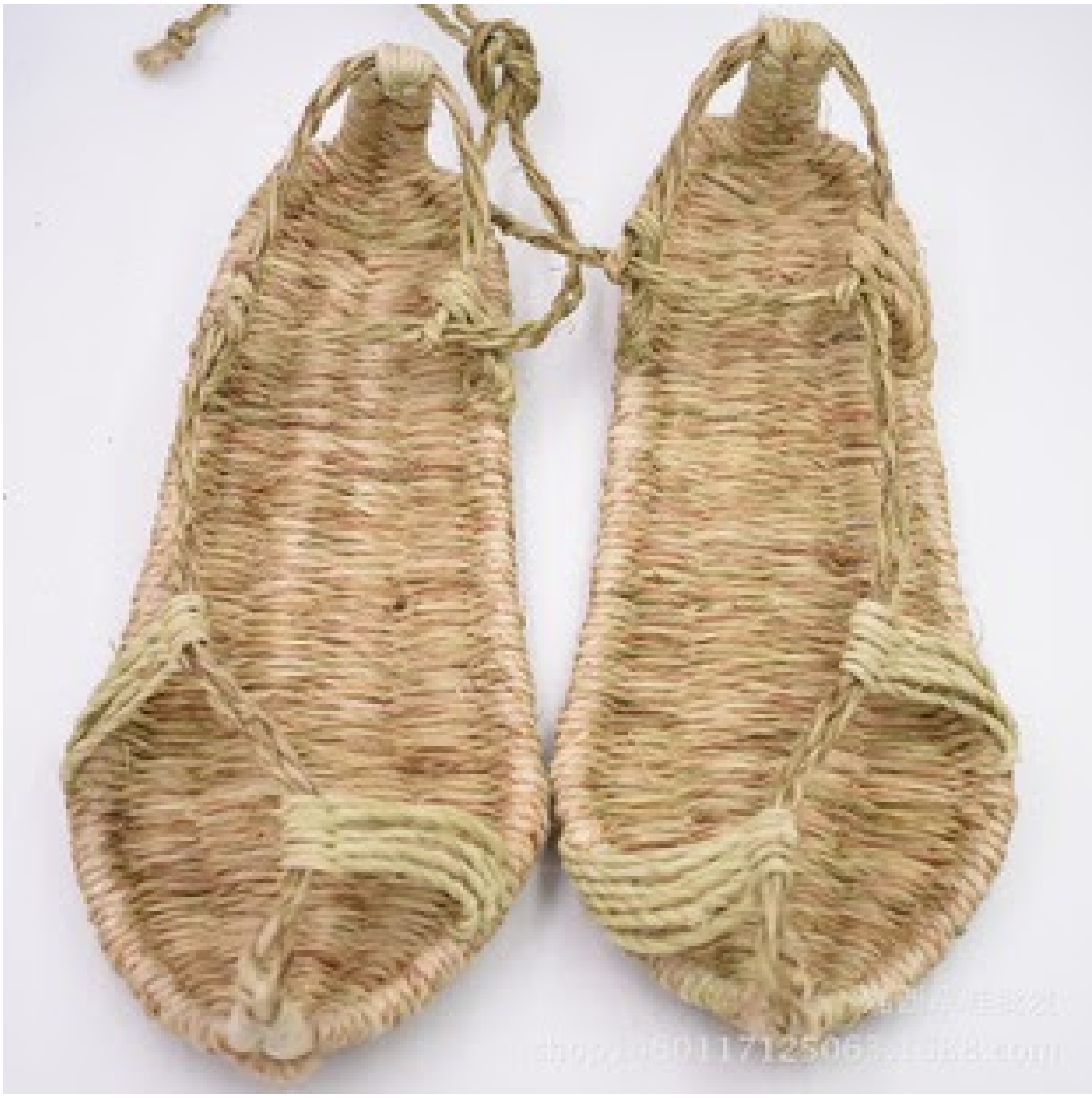

Traditional straw sandals

- Strongly disagree
- Disagree
- Slightly disagree
- Neutral
- Slightly agree
- Agree
- Strongly agree

33. Please rate your familiarity with this image. *

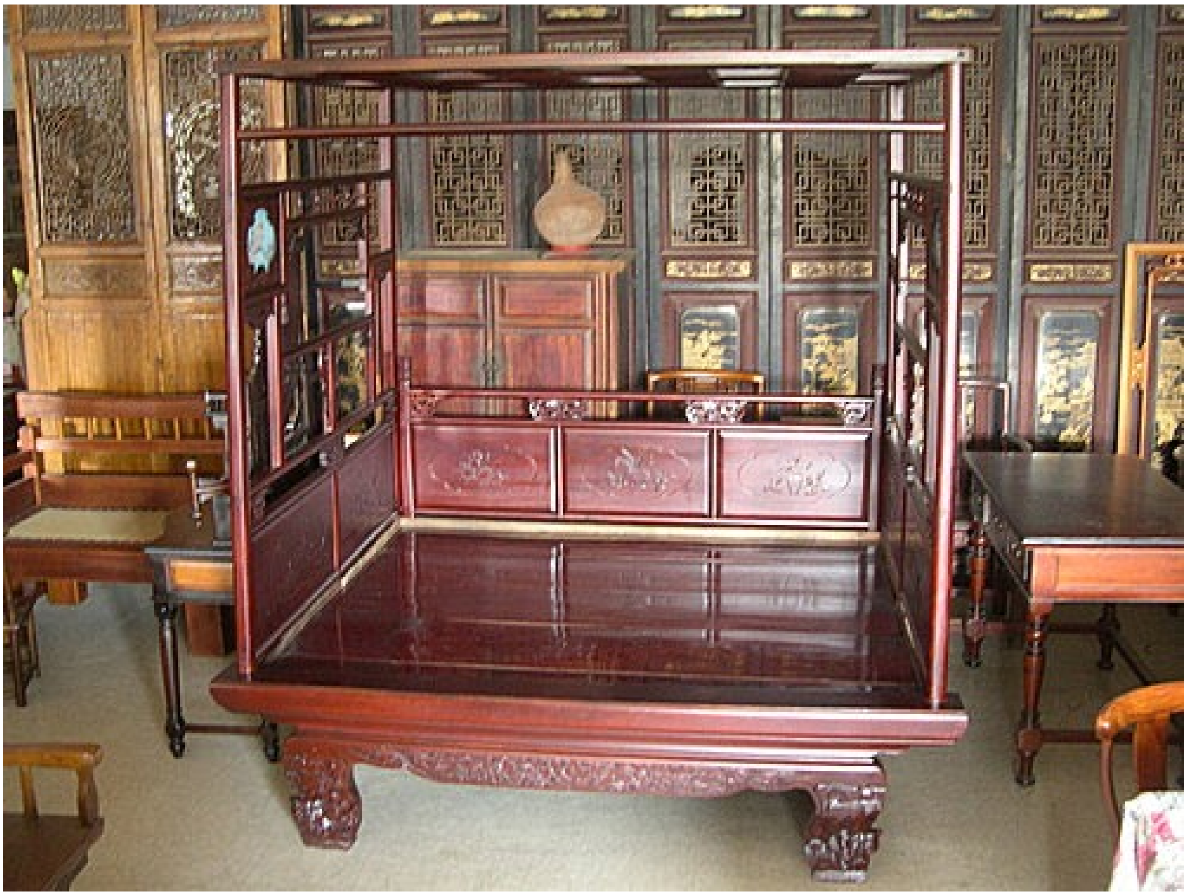

Elevated wood bed frame

- Strongly disagree
- Disagree
- Slightly disagree
- Neutral
- Slightly agree
- Agree
- Strongly agree

34. Please rate your familiarity with this image. *

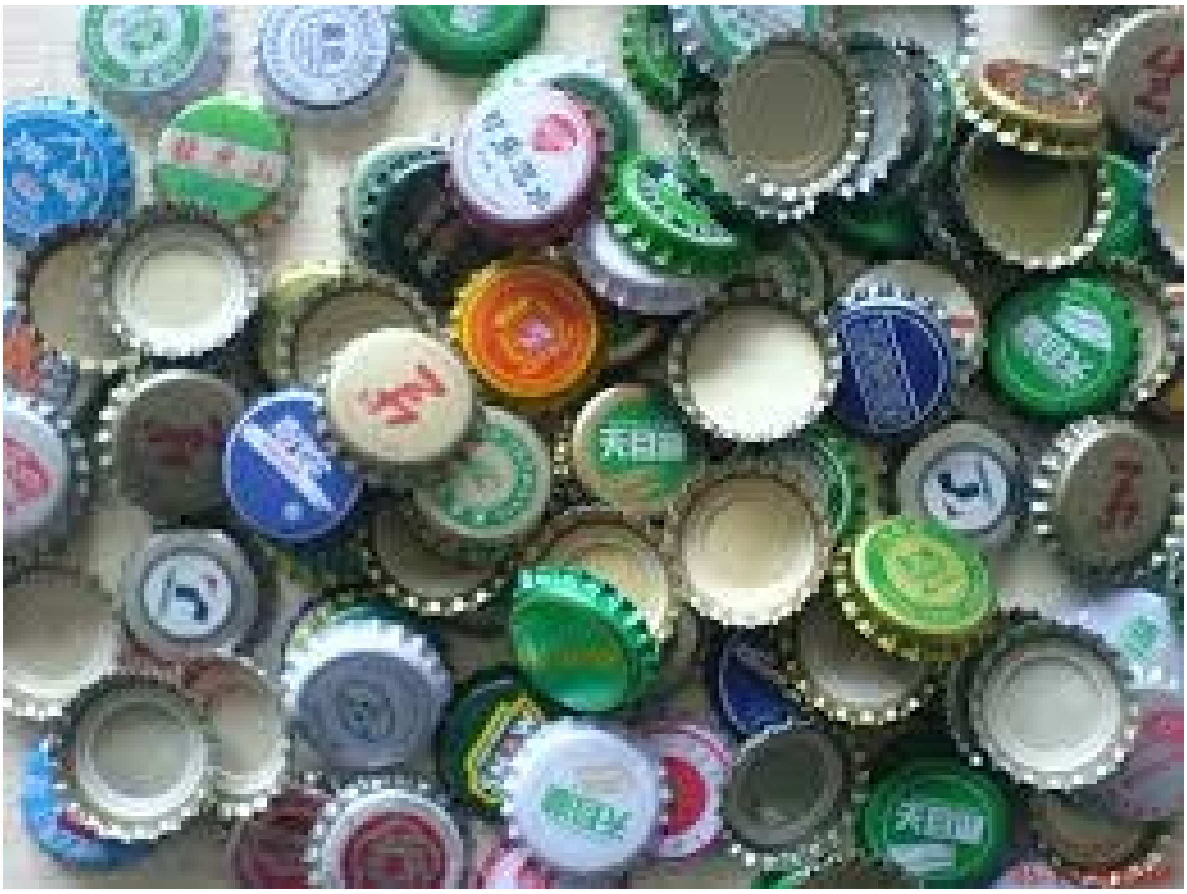

Bottle caps

- Strongly disagree
- Disagree
- Slightly disagree
- Neutral
- Slightly agree
- Agree
- Strongly agree

35. Please rate your familiarity with this image. *

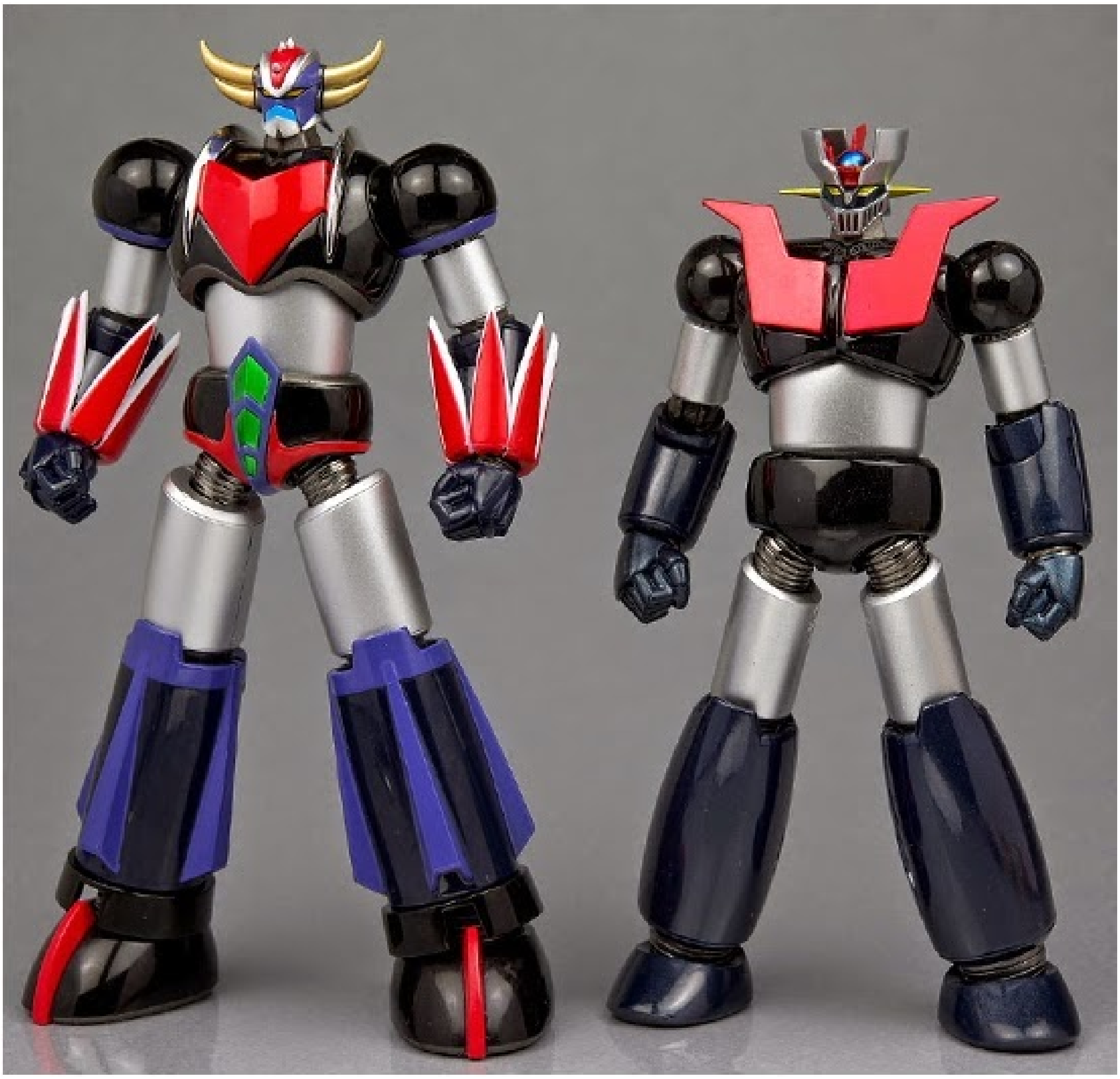

Mazinger Z figurine

- Strongly disagree
- Disagree
- Slightly disagree
- Neutral
- Slightly agree
- Agree
- Strongly agree

36. Please rate your familiarity with this image. *

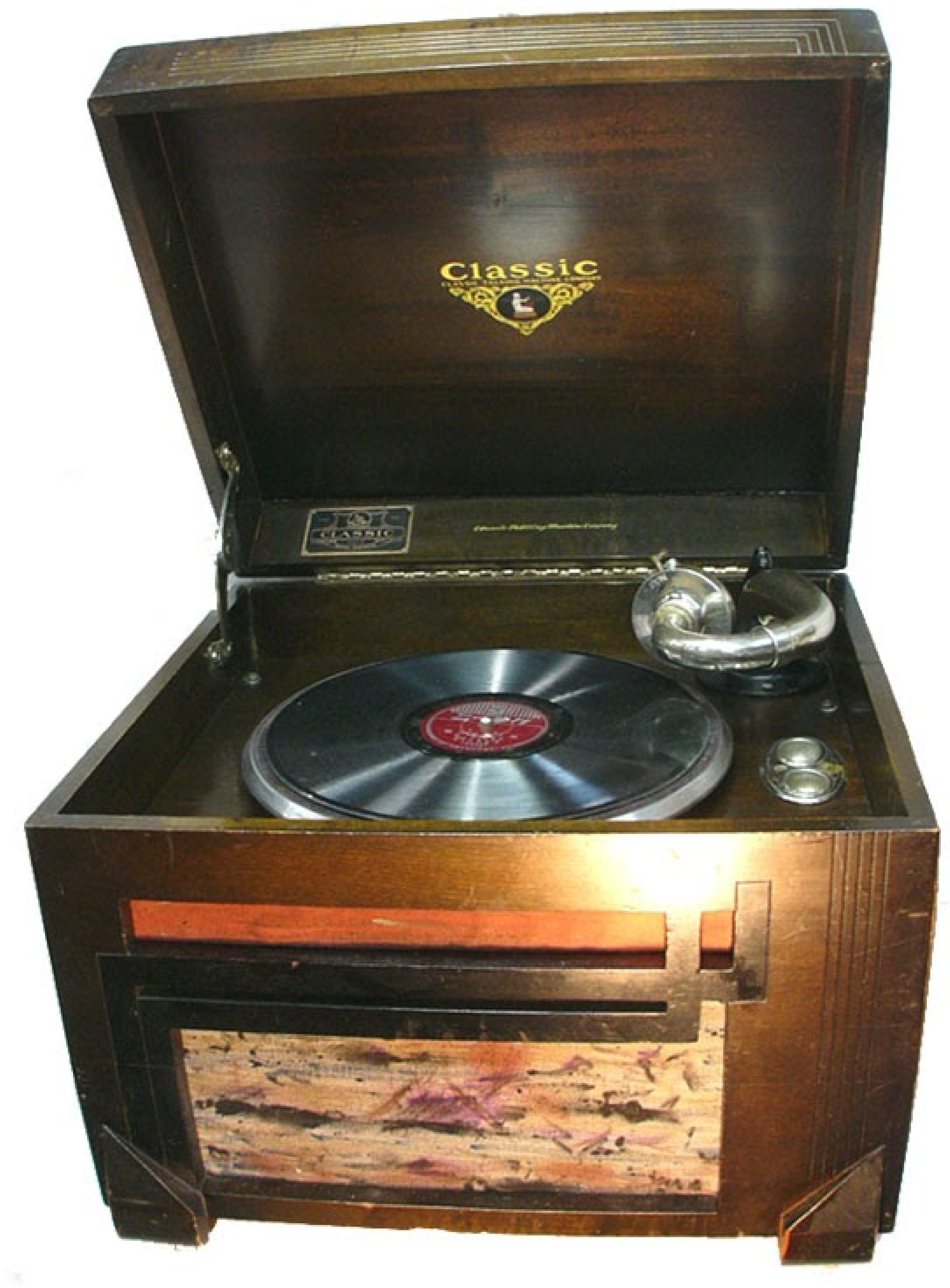

Turntable

- Strongly disagree
- Disagree
- Slightly disagree
- Neutral
- Slightly agree

○ Agree

○ Strongly agree

37. Please rate your familiarity with this image. *

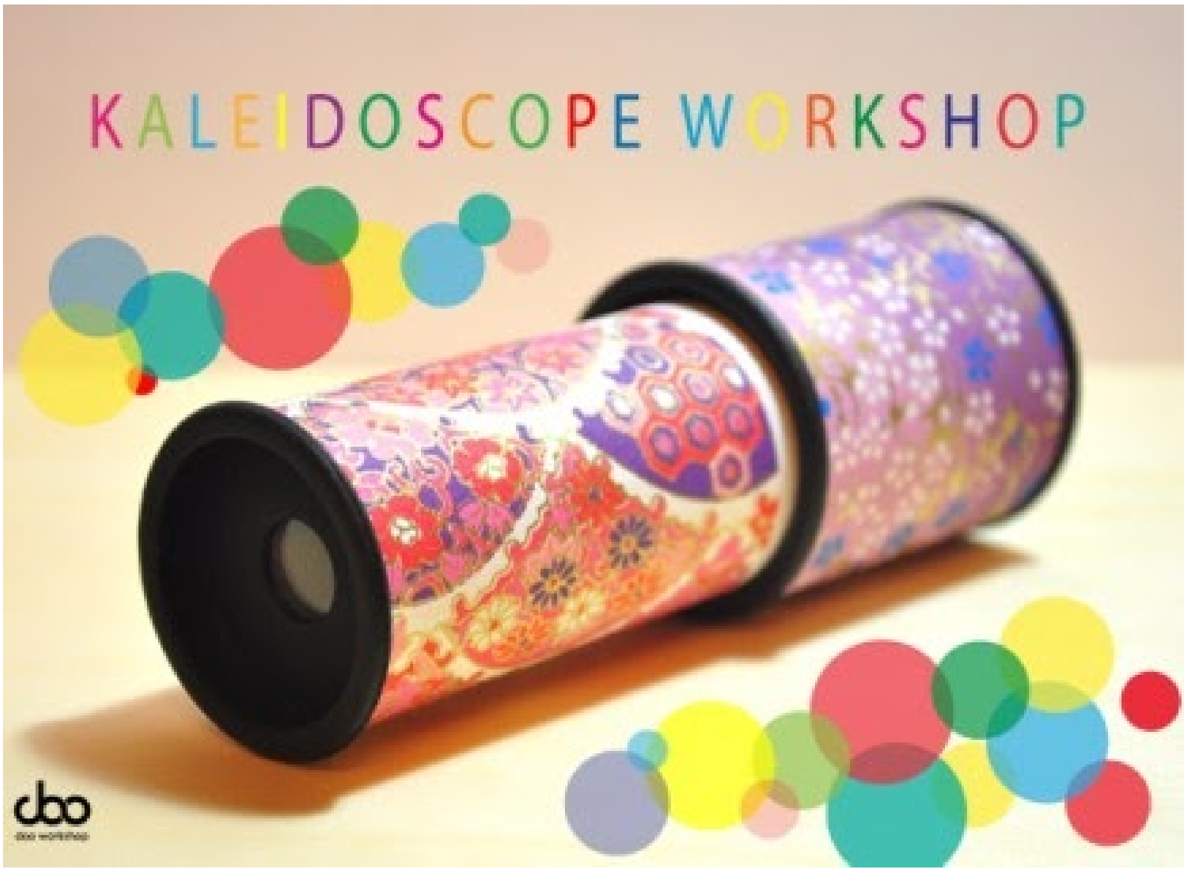

Kaleidoscope

○ Strongly disagree

○ Disagree

○ Slightly disagree

○ Neutral

○ Slightly agree

○ Agree

○ Strongly agree

38. Please rate your familiarity with this image. *

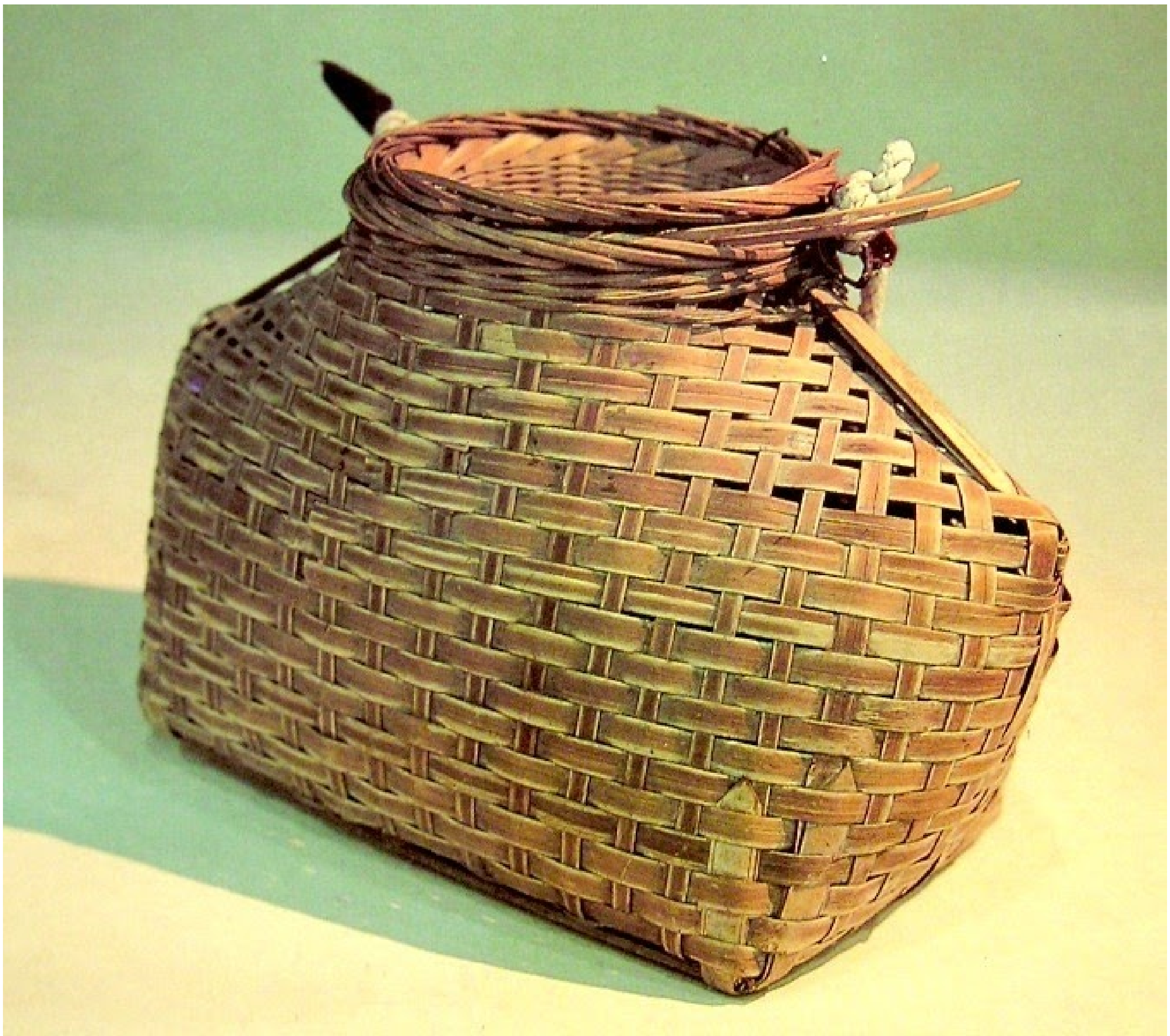

Bamboo fish basket

- Strongly disagree
- Disagree
- Slightly disagree
- Neutral
- Slightly agree
- Agree
- Strongly agree

39. Please rate your familiarity with this image. *

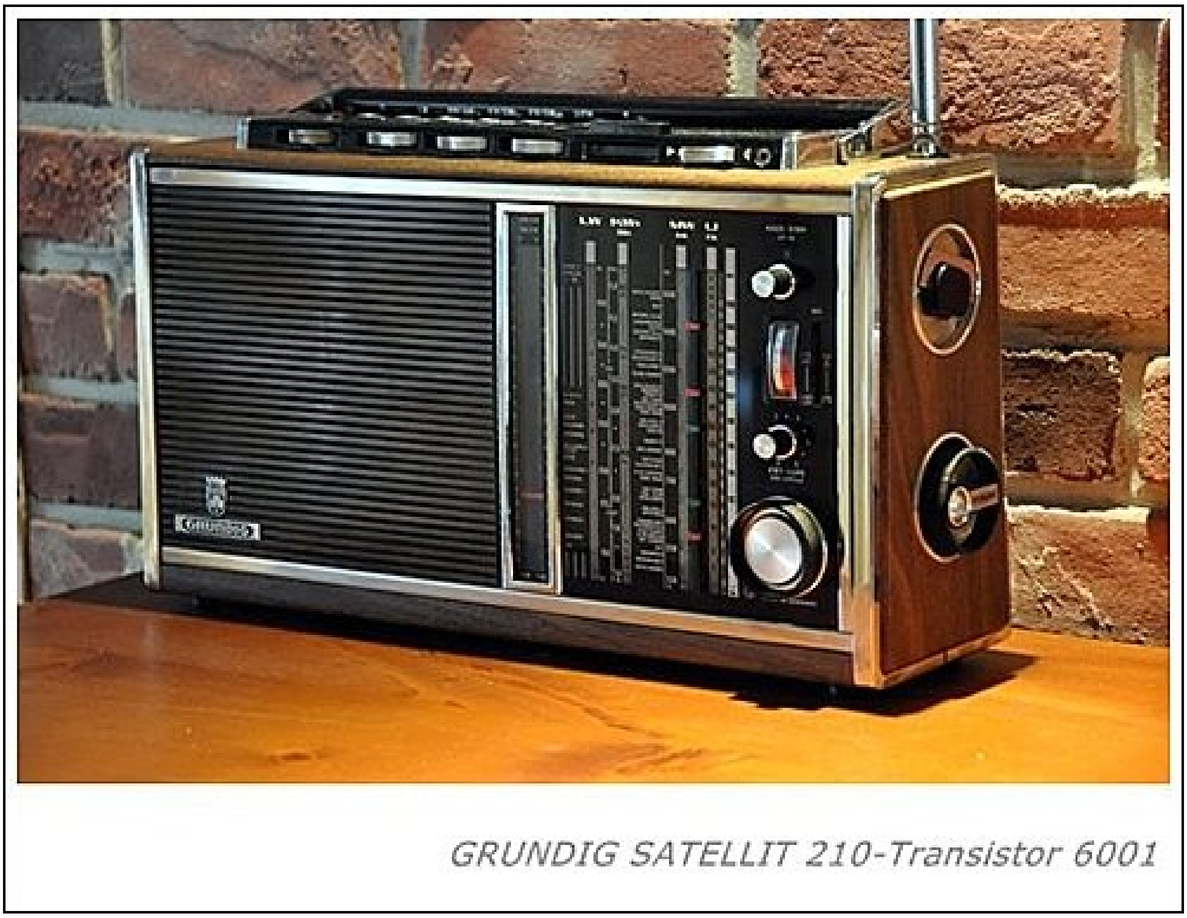

Transistor radio

- Strongly disagree
- Disagree
- Slightly disagree
- Neutral
- Slightly agree
- Agree
- Strongly agree

40. Please rate your familiarity with this image. *

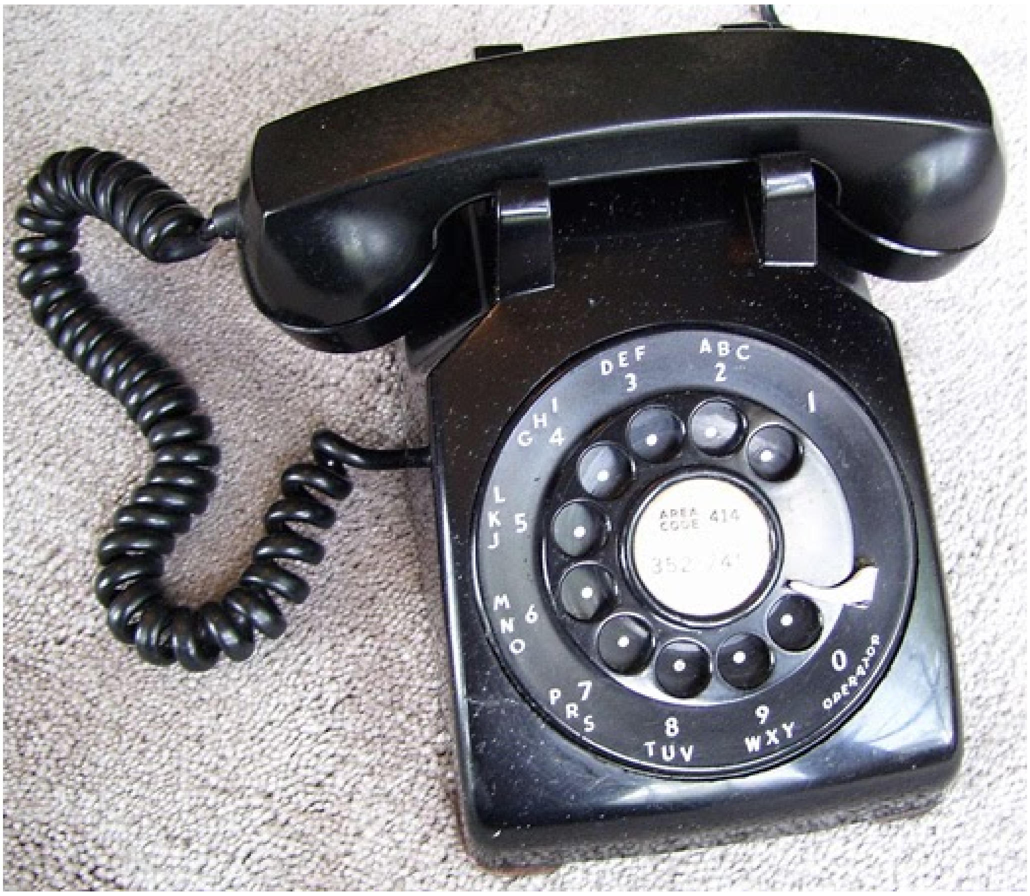

Rotary phone

- Strongly disagree
- Disagree
- Slightly disagree
- Neutral
- Slightly agree
- Agree
- Strongly agree

41. Please rate your familiarity with this image. *

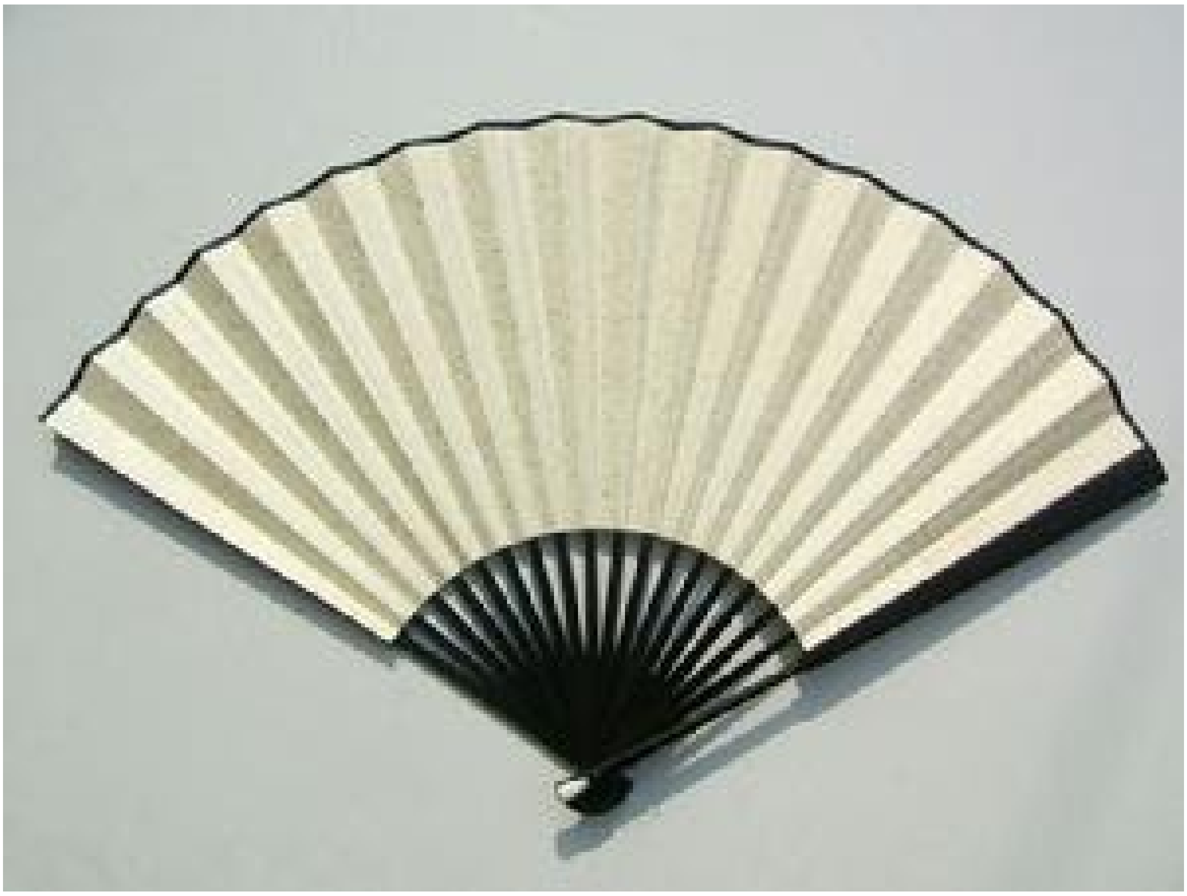

Hand fan

- Strongly disagree
- Disagree
- Slightly disagree
- Neutral
- Slightly agree
- Agree
- Strongly agree

42. Please rate your familiarity with this image. *

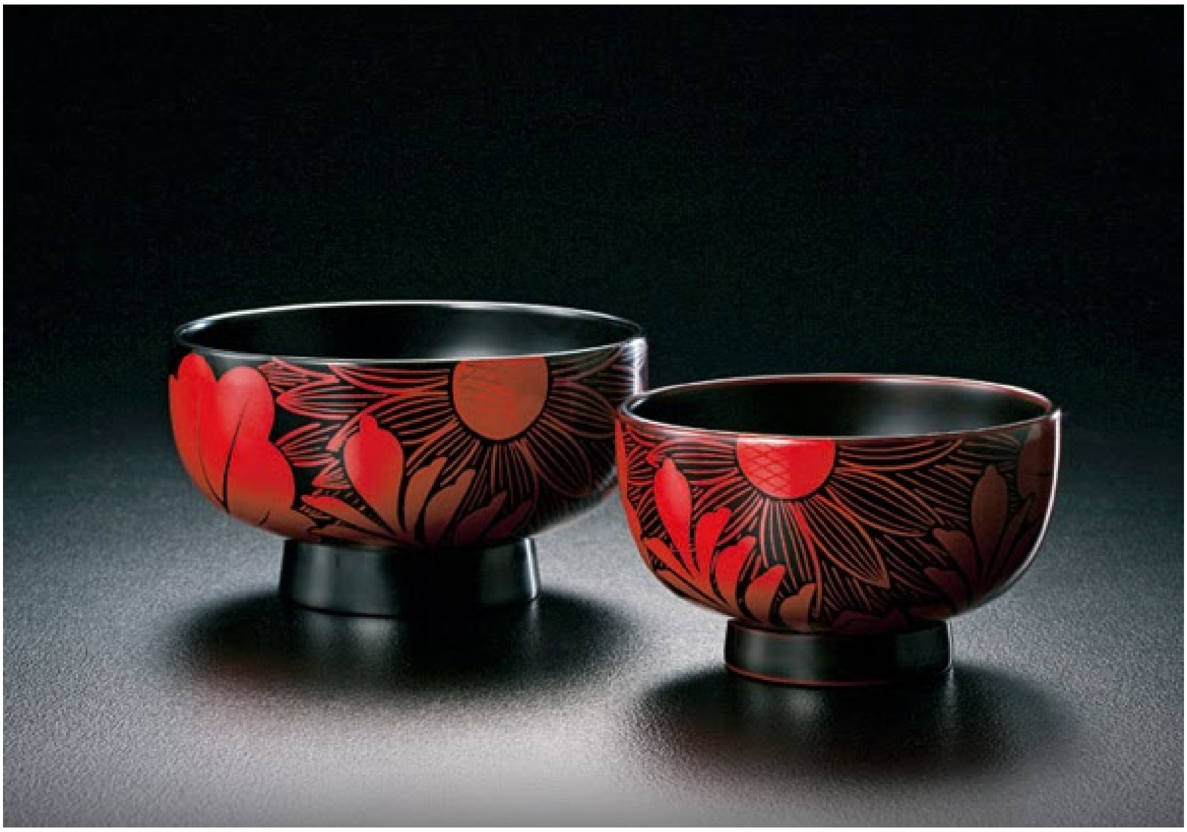

Lacquerware

- Strongly disagree
- Disagree
- Slightly disagree
- Neutral
- Slightly agree
- Agree
- Strongly agree

43. Please rate your familiarity with this image. *

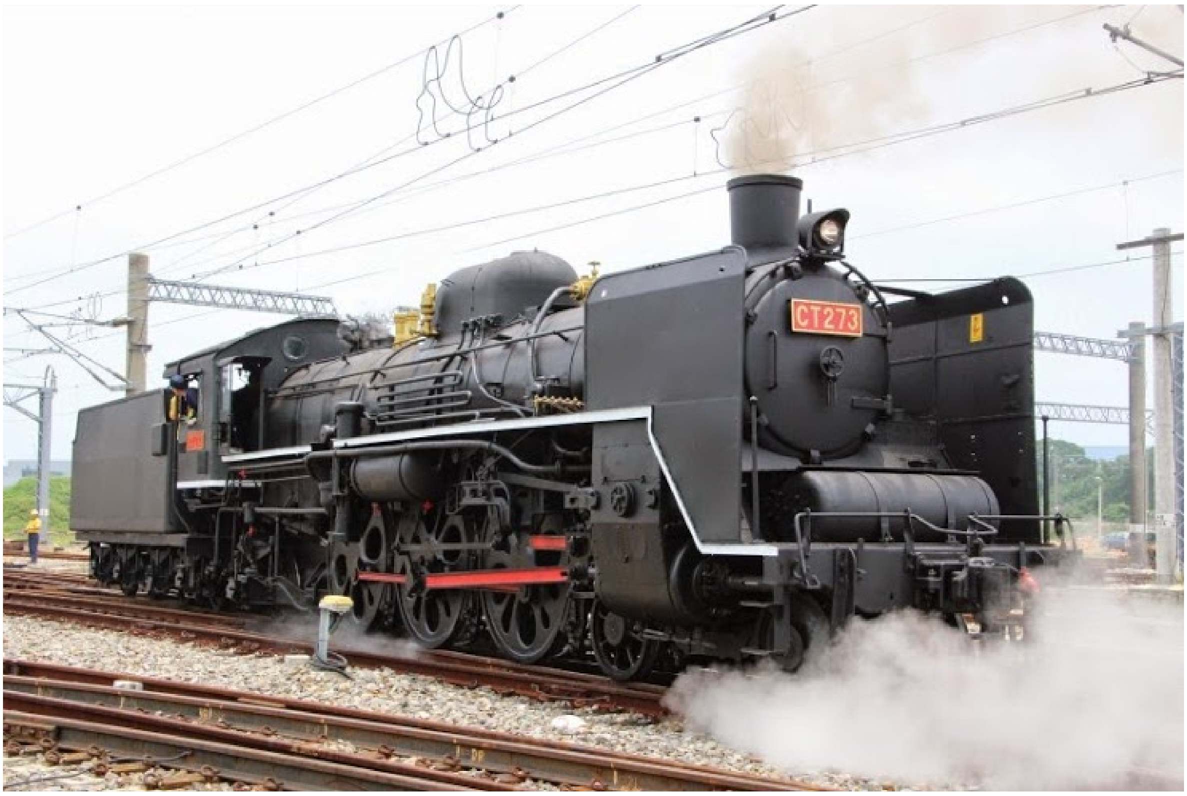

Steam trains

- Strongly disagree
- Disagree
- Slightly disagree
- Neutral
- Slightly agree
- Agree
- Strongly agree

44. Please rate your familiarity with this image. *

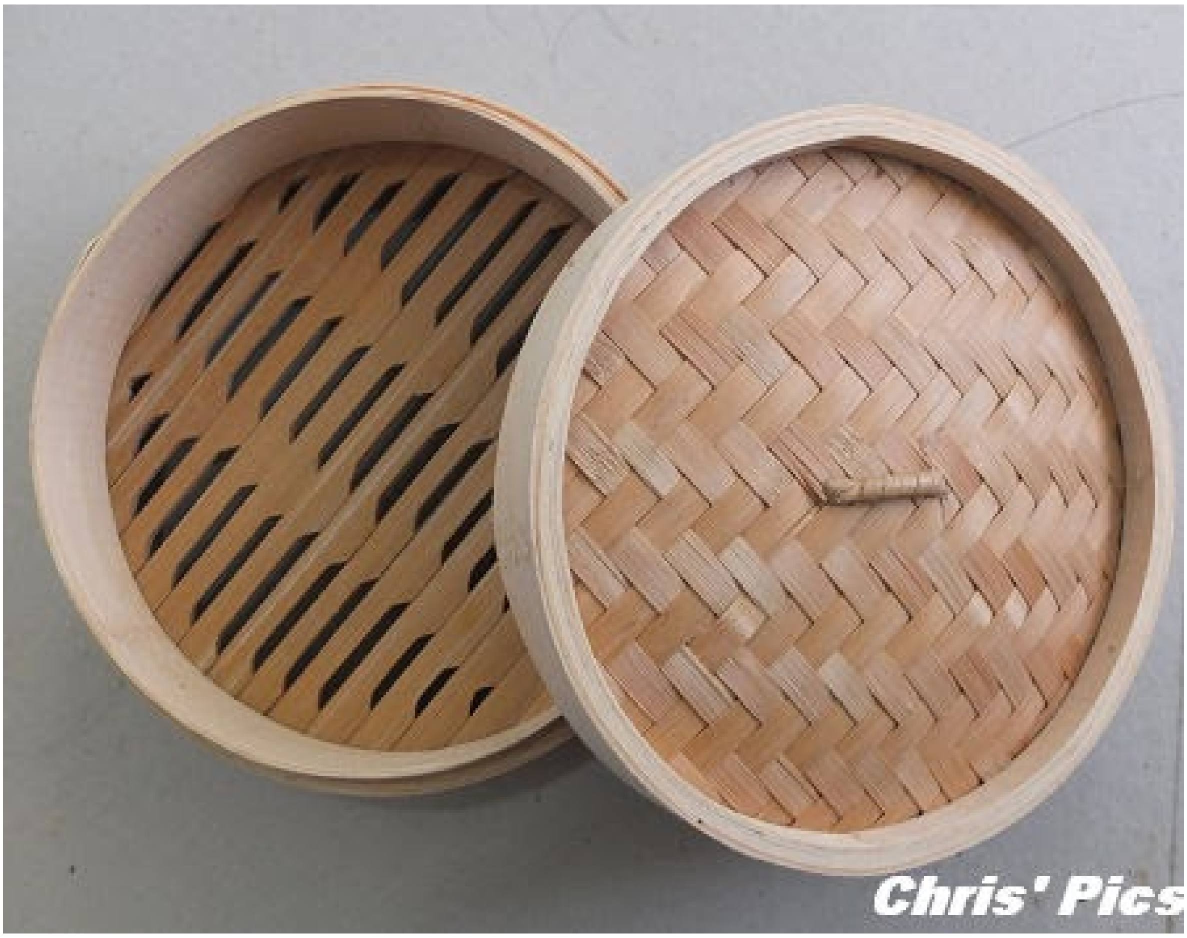

Bamboo steamer

- Strongly disagree
- Disagree
- Slightly disagree
- Neutral
- Slightly agree
- Agree
- Strongly agree

45. Please rate your familiarity with this image. *

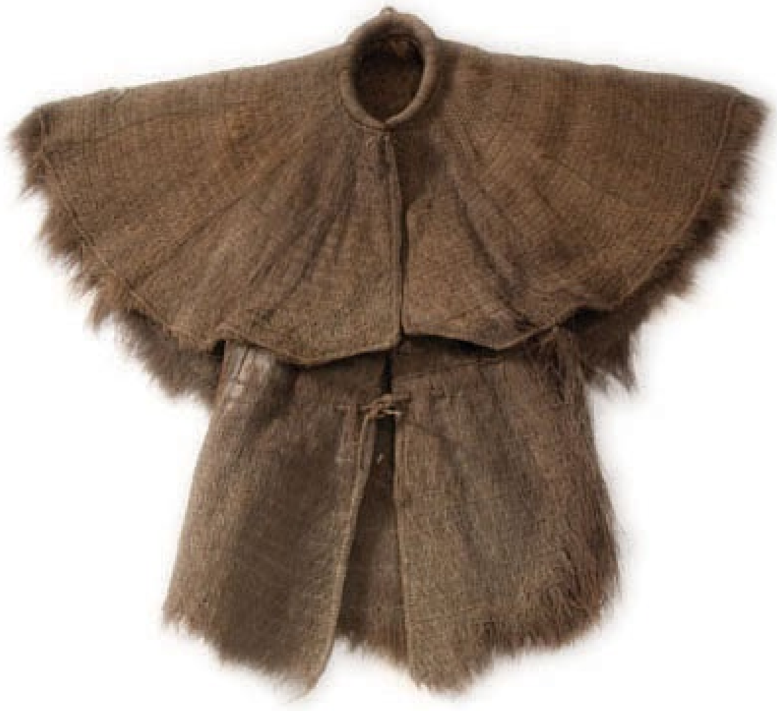

Straw cape

- Strongly disagree
- Disagree
- Slightly disagree
- Neutral
- Slightly agree
- Agree
- Strongly agree

46. Please rate your familiarity with this image. *

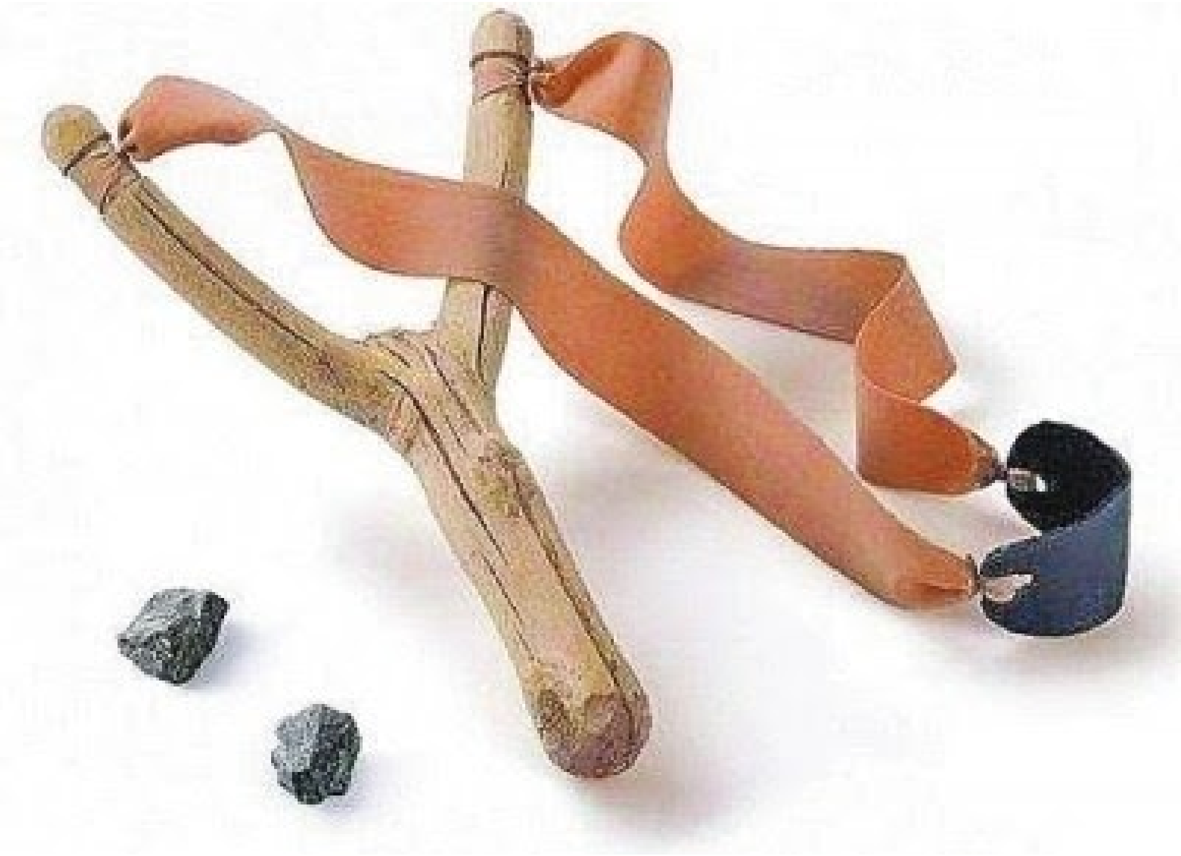

Slingshot

- Strongly disagree
- Disagree
- Slightly disagree
- Neutral
- Slightly agree
- Agree
- Strongly agree

47. Please rate your familiarity with this image. *

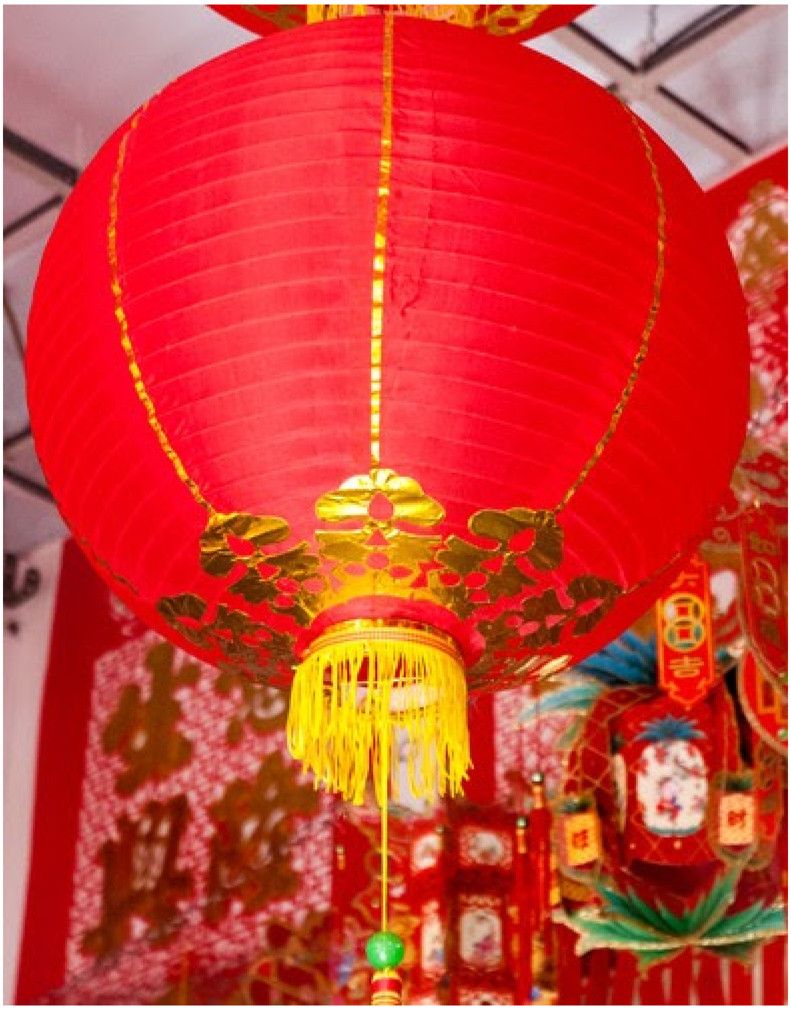

Lantern

- Strongly disagree
- Disagree
- Slightly disagree
- Neutral
- Slightly agree
- Agree
- Strongly agree

48. Please rate your familiarity with this image. *

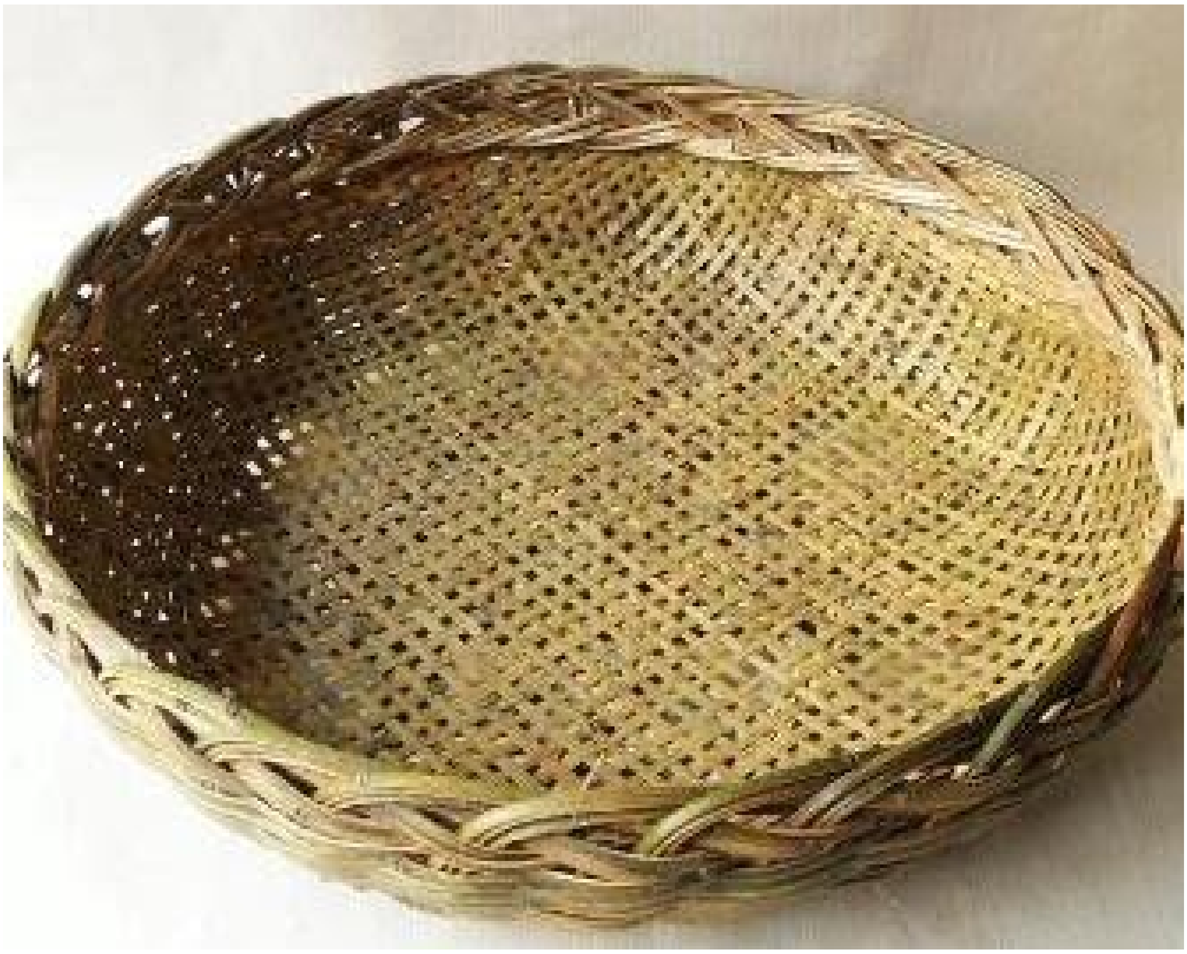

Bamboo sieve

- Strongly disagree
- Disagree
- Slightly disagree
- Neutral
- Slightly agree
- Agree
- Strongly agree

49. Please rate your familiarity with this image. *

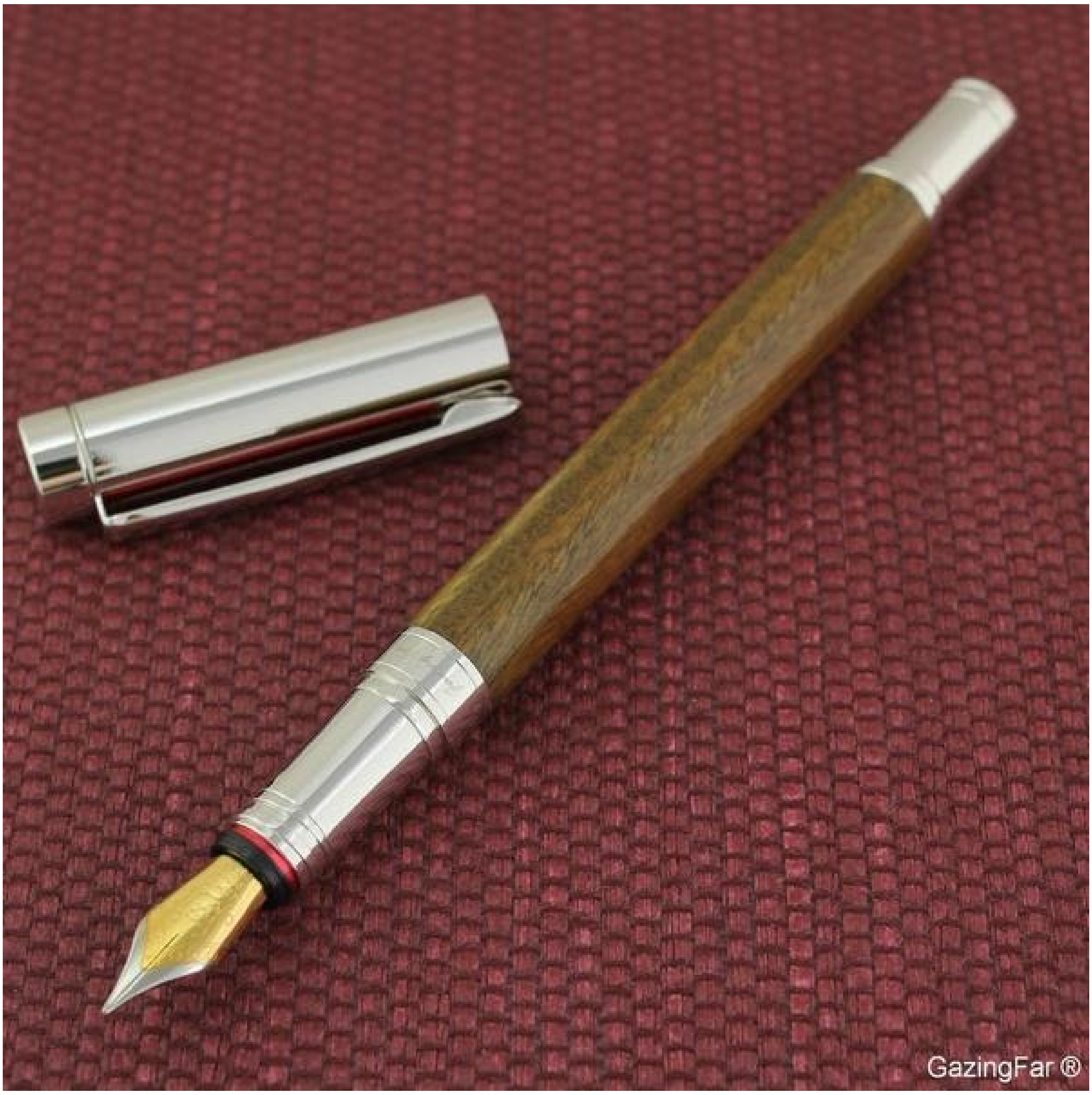

Fountain pen

- Strongly disagree
- Disagree
- Slightly disagree
- Neutral
- Slightly agree
- Agree
- Strongly agree

50. Please rate your familiarity with this image. *

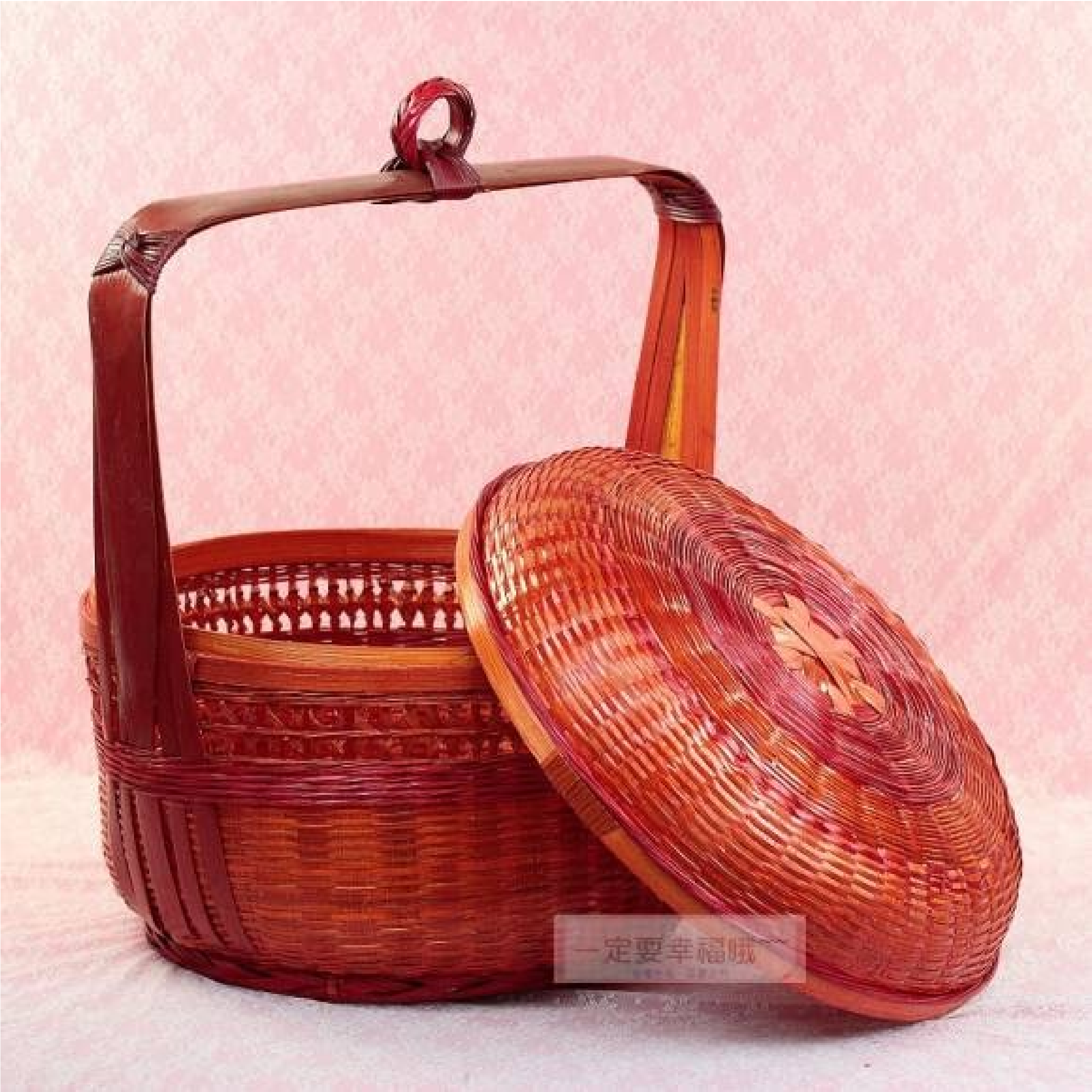

Wedding gift basket

- Strongy disagree
- Disagree
- Slightly disagree
- Neutral
- Slightly agree
- Agree
- Strongly agree

51. Please rate your familiarity with this image. *

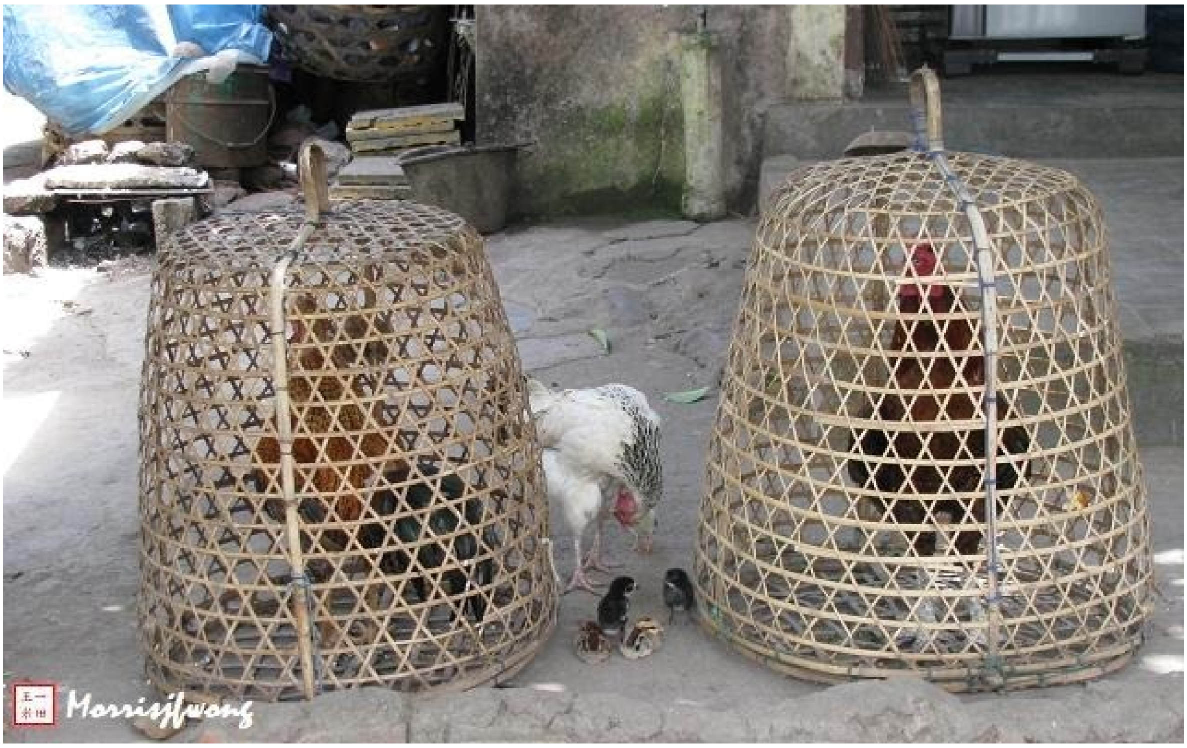

Bamboo chicken coop

- Strongly disagree
- Disagree
- Slightly disagree
- Neutral
- Slightly agree
- Agree
- Strongly agree

52. Please rate your familiarity with this image. *

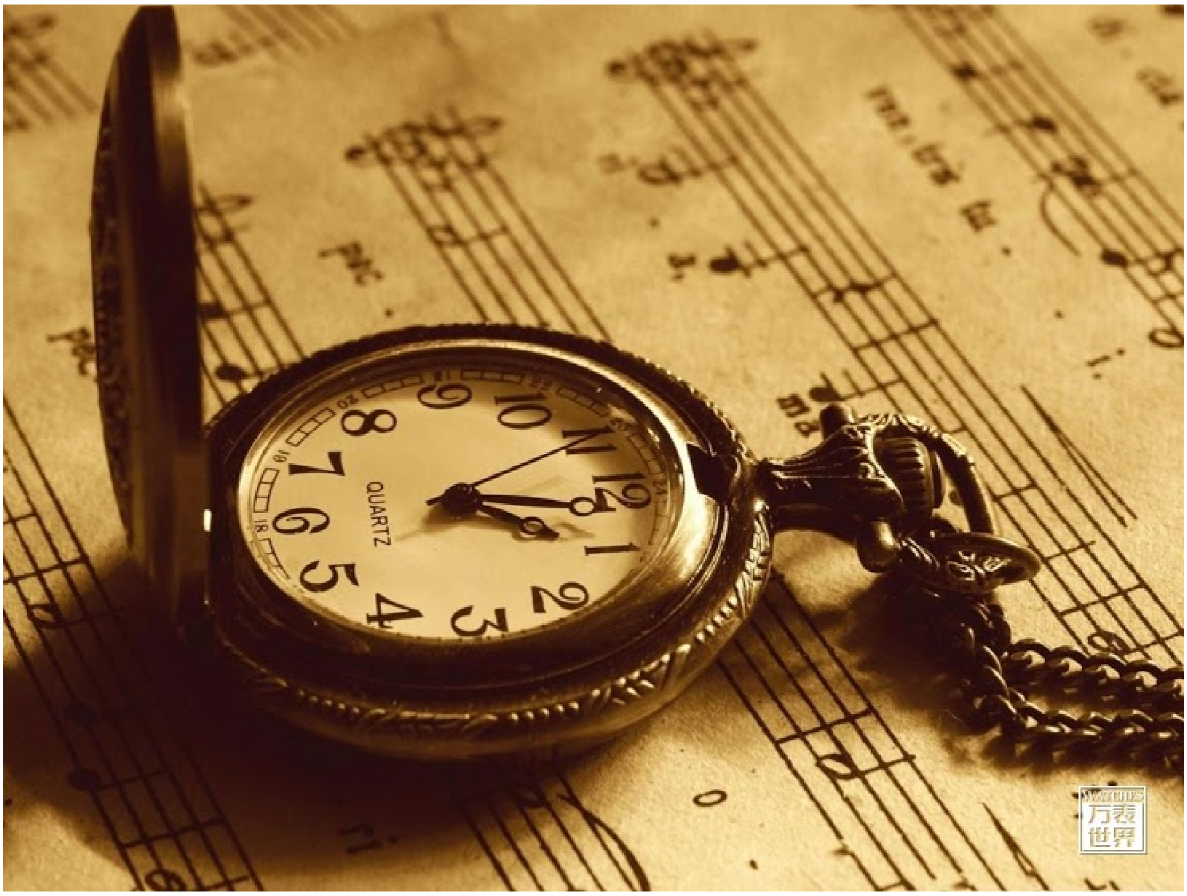

Pocket watch

- Strongly disagree
- Disagree
- Slightly disagree
- Neutral
- Slightly agree
- Agree
- Strongly agree

53. Please rate your familiarity with this image. *

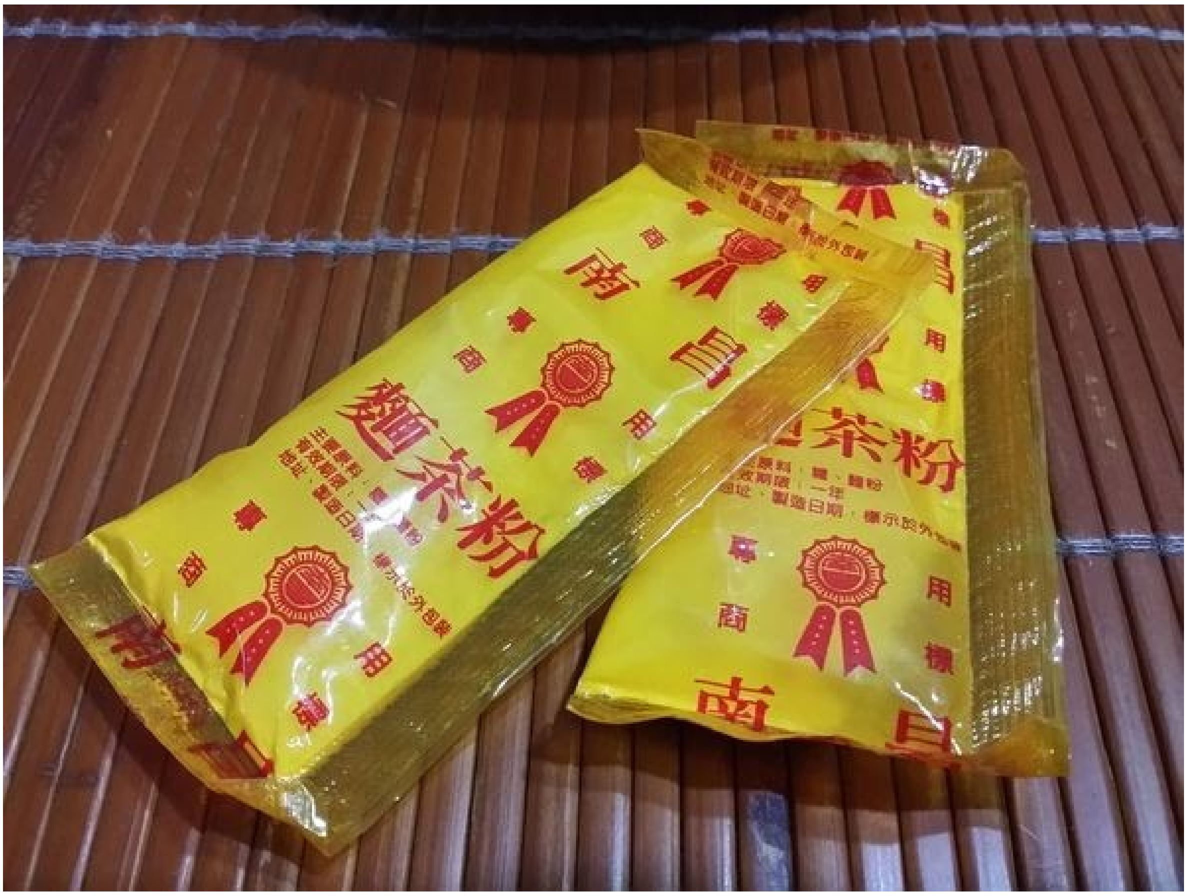

Roasted wheat flour

- Strongly disagree
- Disagree
- Slightly disagree
- Neutral
- Slightly agree
- Agree
- Strongly agree

54. Please rate your familiarity with this image. *

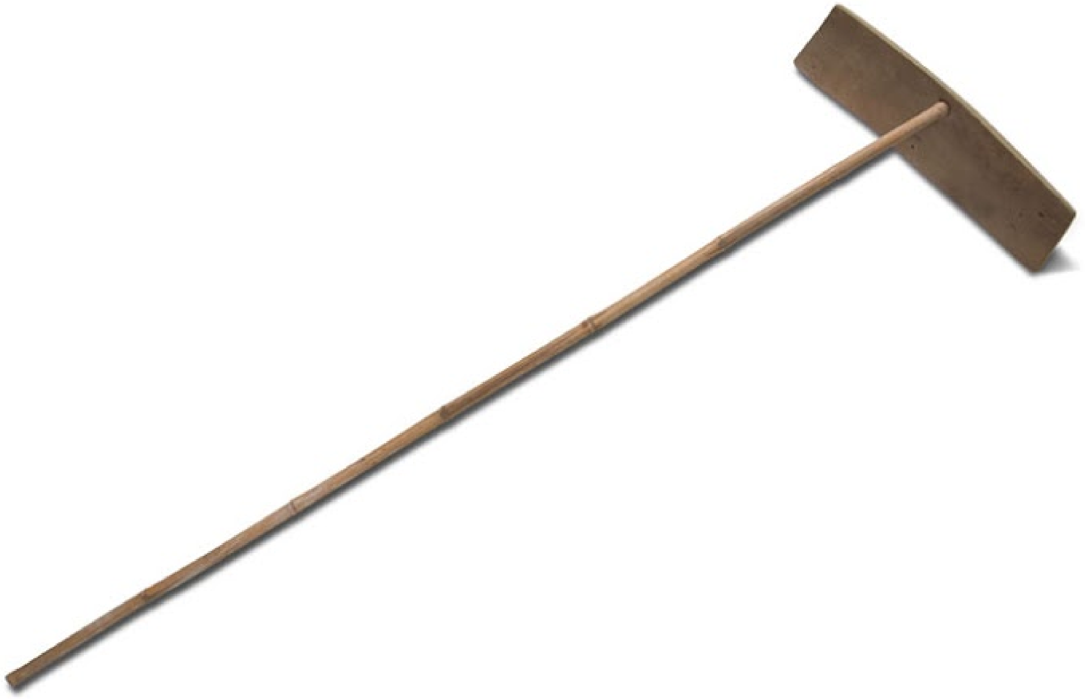

Harrow

- Strongly disagree
- Disagree
- Slightly disagree
- Neutral
- Slightly agree
- Agree
- Strongly agree

55. Please rate your familiarity with this image. *

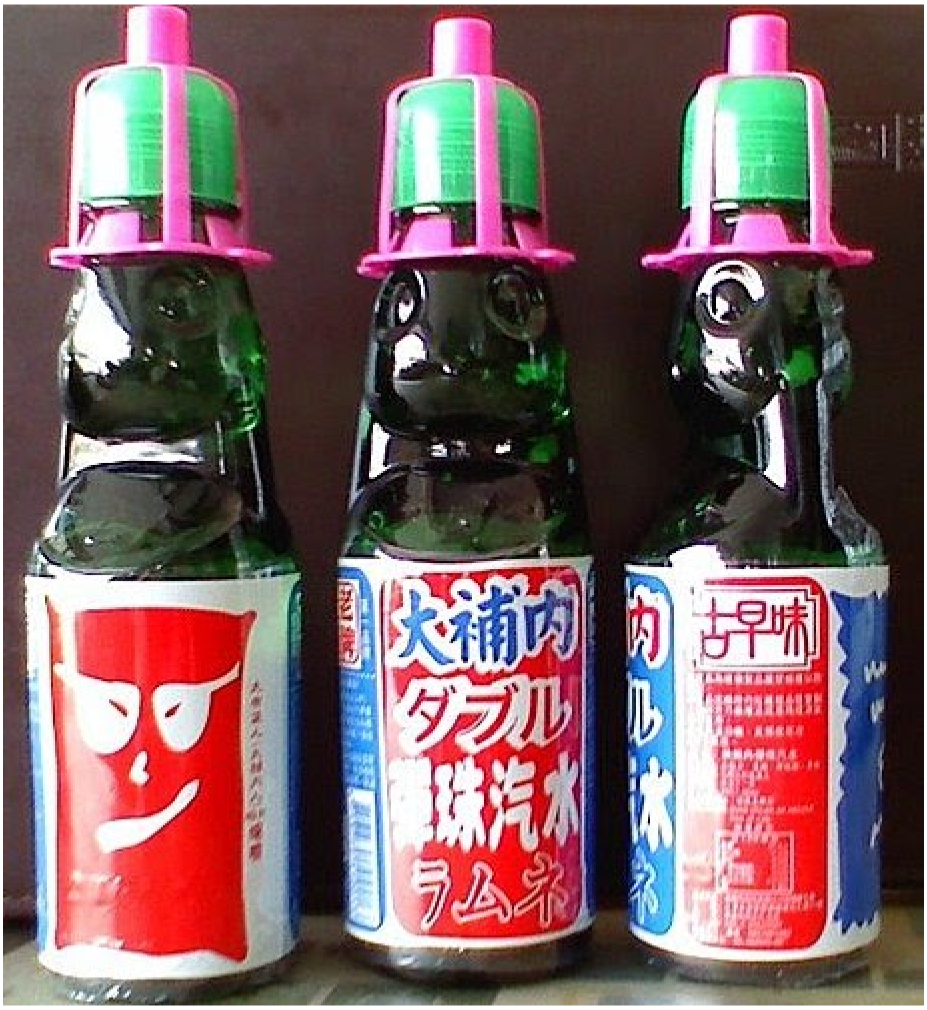

Marble soda

- ◯ Strongly disagree
- ◯ Disagree
- ◯ Slightly disagree
- ◯ Neutral
- ◯ Slightly agree
- ◯ Agree
- ◯ Strongly agree

56. Please rate your familiarity with this image. *

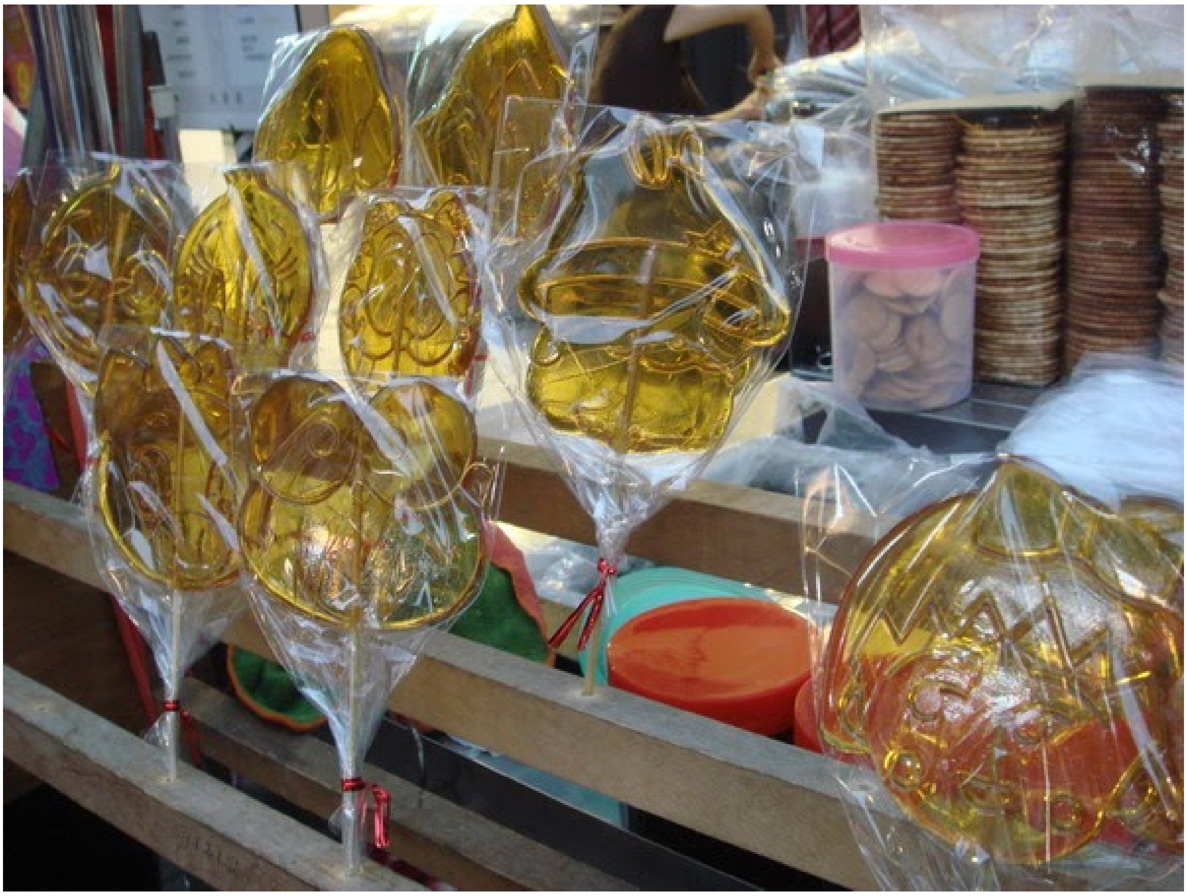

Maltose candy

- Strongly disagree
- Disagree
- Slightly disagree
- Neutral
- Slightly agree
- Agree
- Strongly agree

57. Please rate your familiarity with this image. *

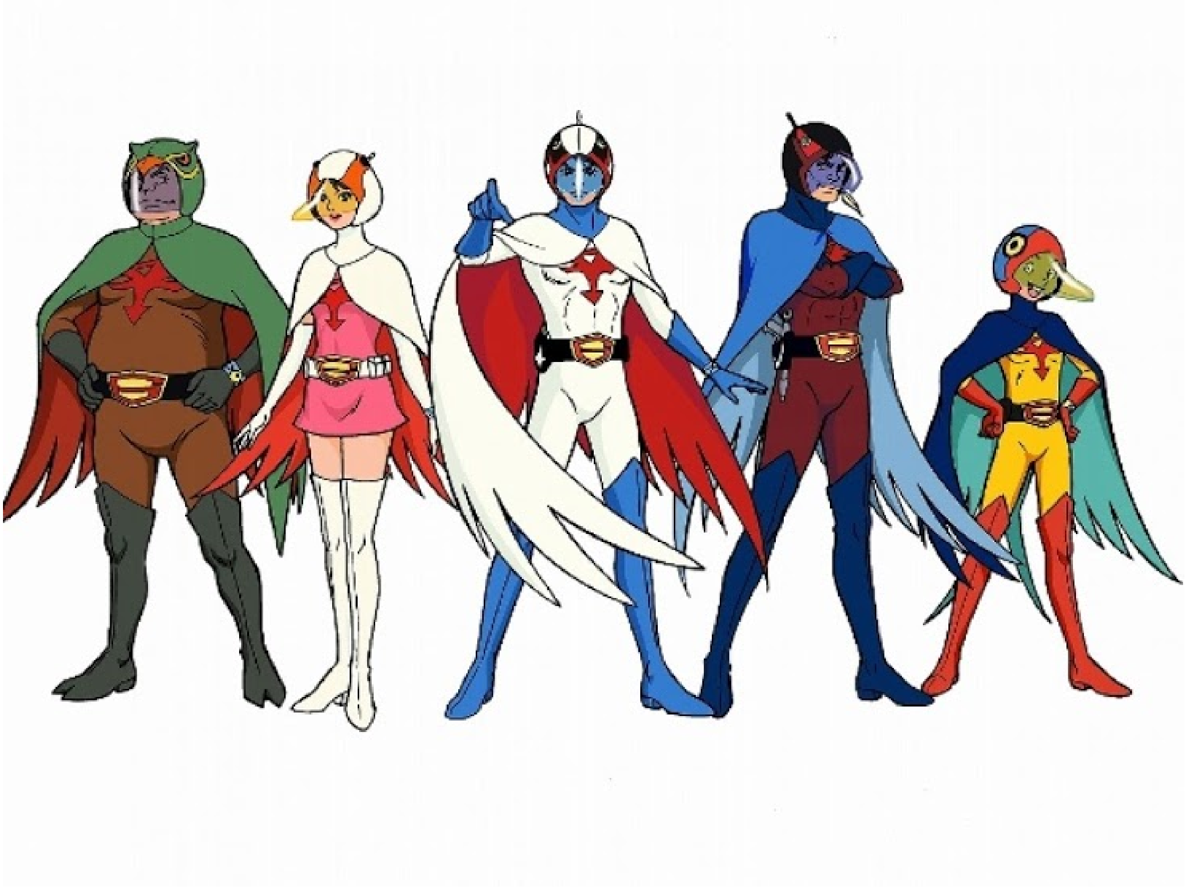

Science Ninja Team Gatchaman

- Strongly disagree
- Disagree
- Slightly disagree
- Neutral
- Slightly agree
- Agree
- Strongly agree

58. Please rate your familiarity with this image. *

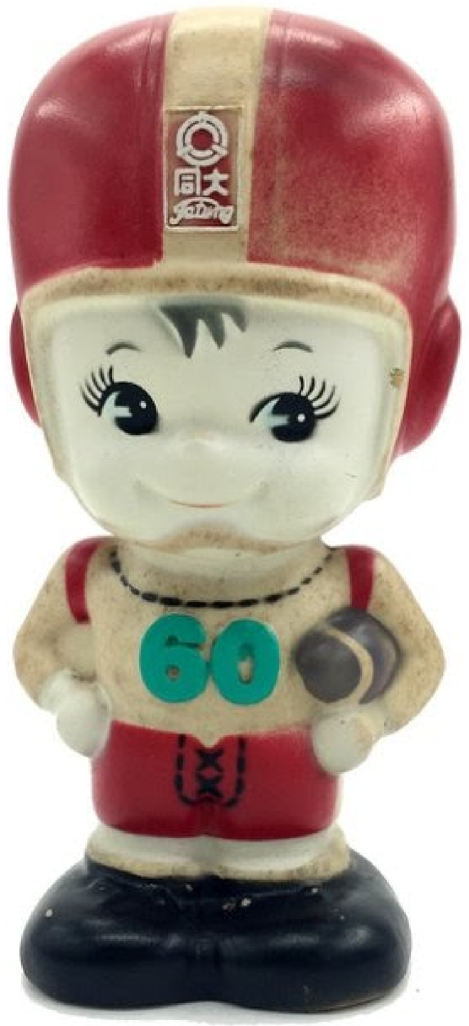

Toy figurine "Tatung Boy"

- ◯ Strongly disagree
- ◯ Disagree
- ◯ Slightly disagree
- ◯ Neutral
- ◯ Slightly agree
- ◯ Agree
- ◯ Strongly agree

59. Please rate your familiarity with this image. *

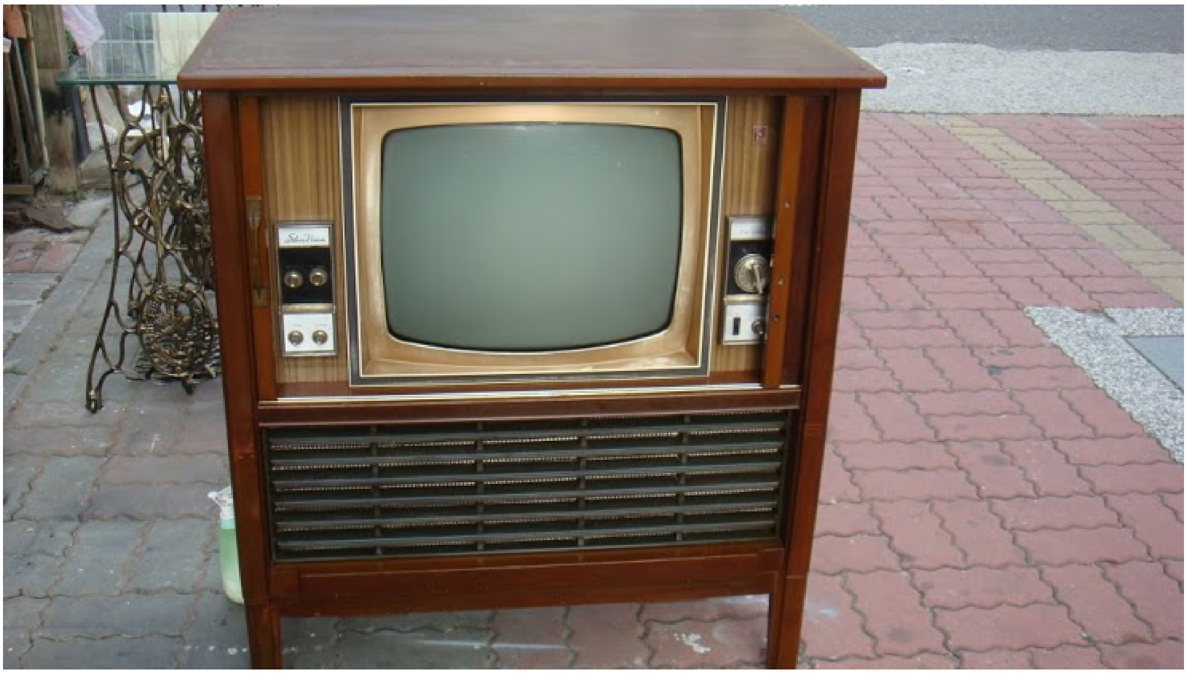

Television

- Strongly disagree
- Disagree
- Slightly disagree
- Neutral
- Slightly agree
- Agree
- Strongly agree

60. Please rate your familiarity with this image. *

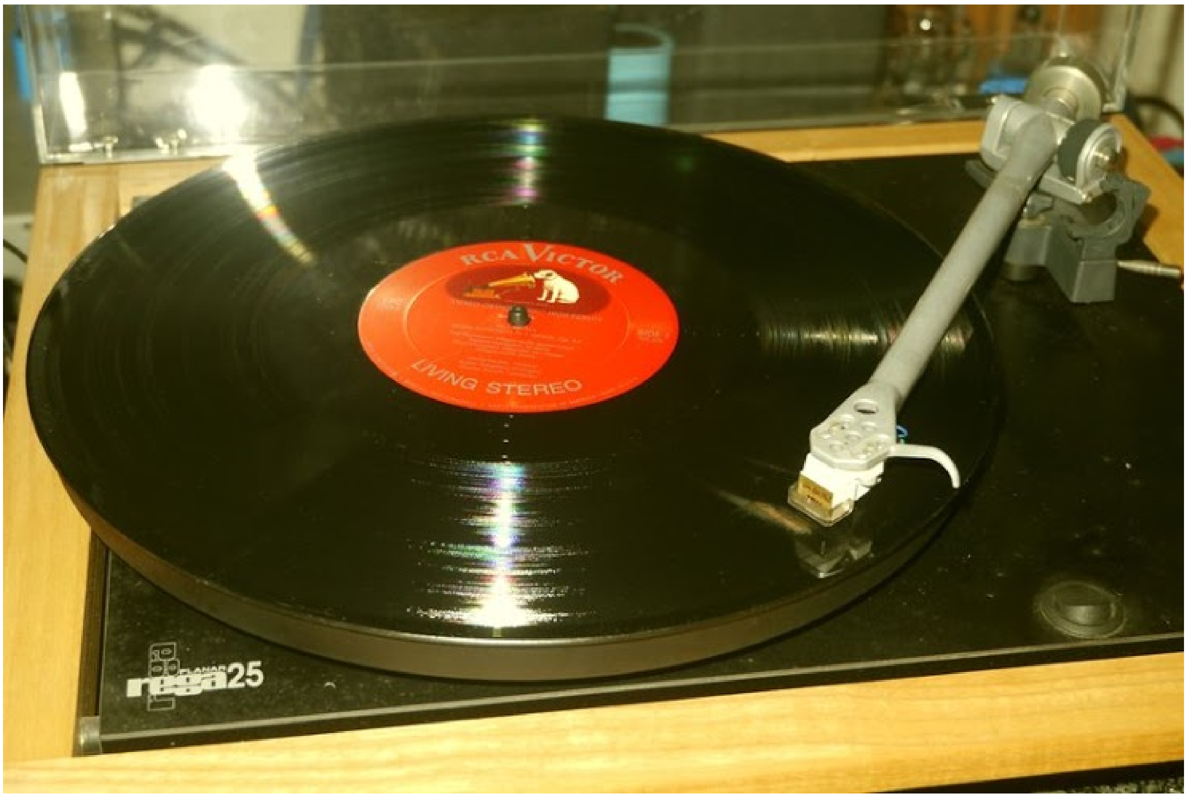

Vinyl records

- Strongly disagree
- Disagree
- Slightly disagree
- Neutral
- Slightly agree
- Agree
- Strongly agree

61. Please rate your familiarity with this image. *

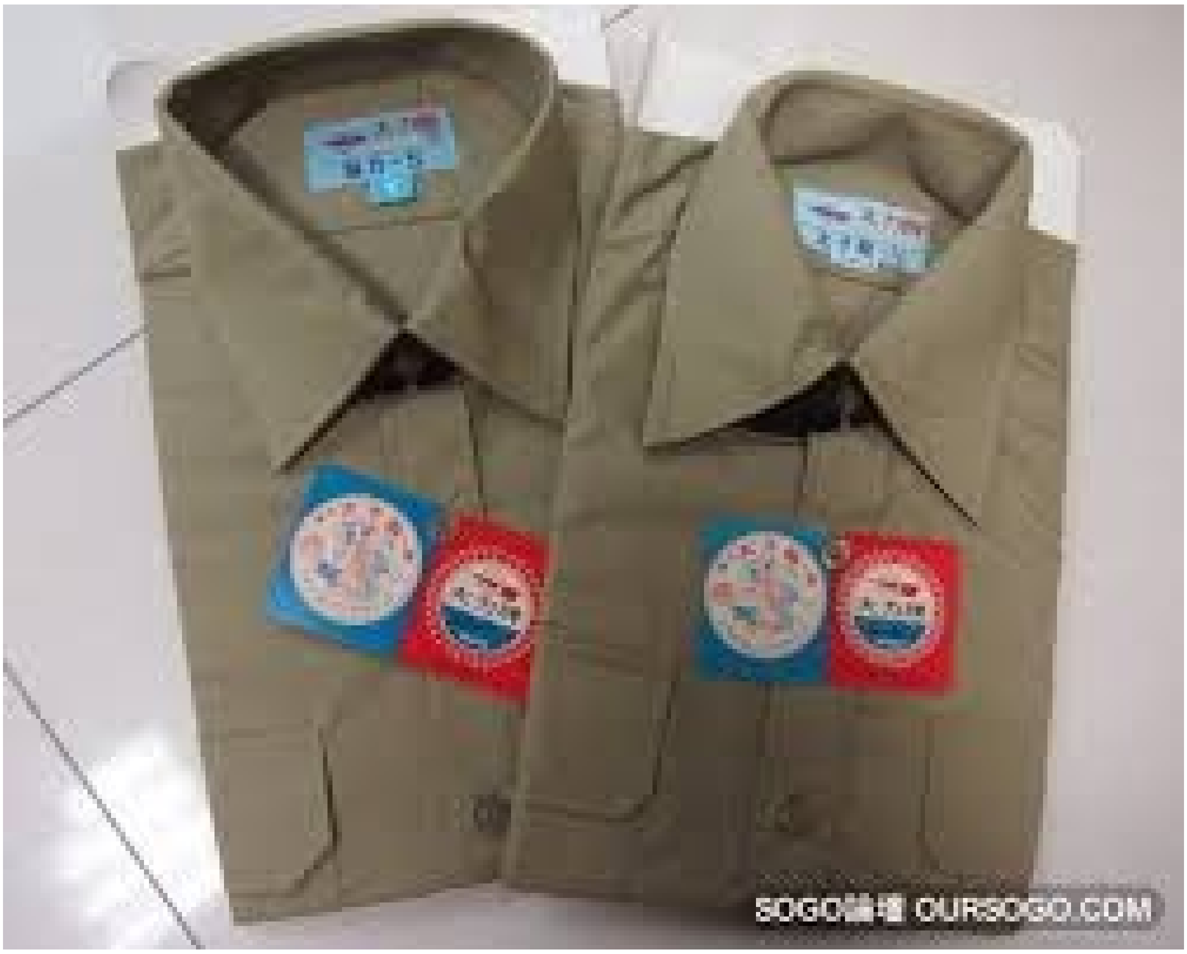

Student uniform

- Strongly disagree
- Disagree
- Slightly disagree
- Neutral
- Slightly agree
- Agree
- Strongly agree

62. Please rate your familiarity with this image. *

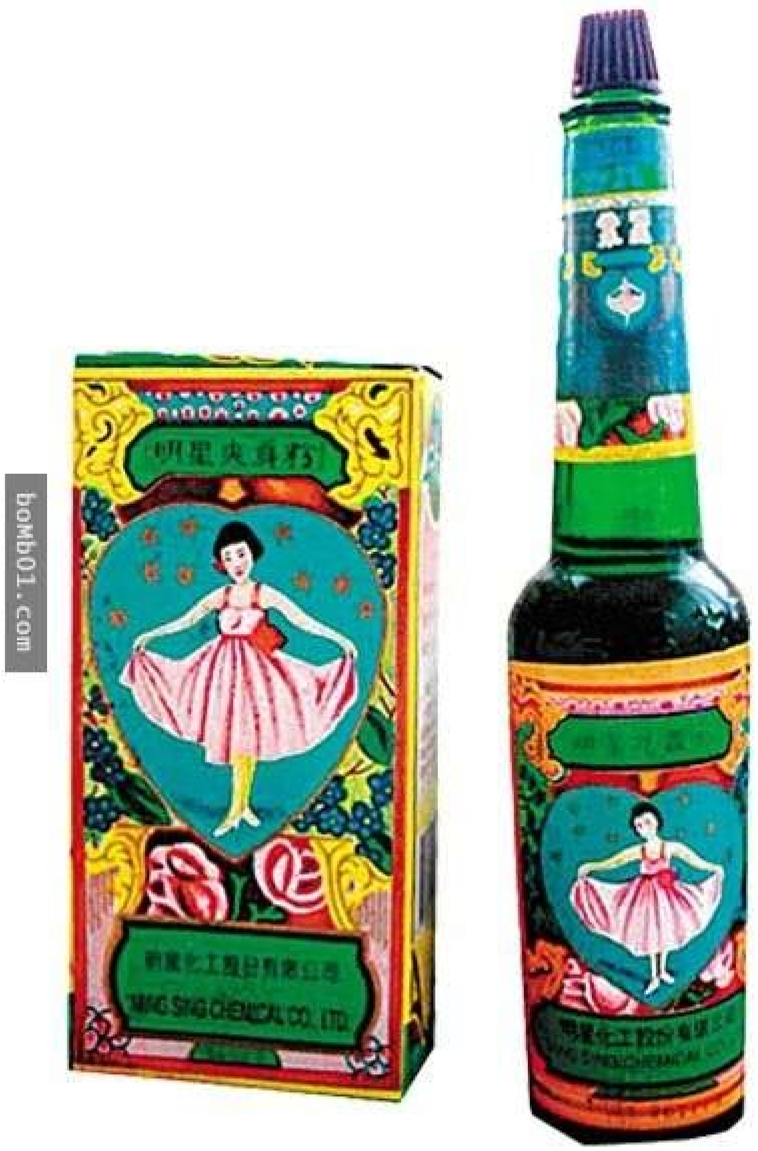

Traditional Taiwanese perfume

- ◯ Strongly disagree
- ◯ Disagree
- ◯ Slightly disagree
- ◯ Neutral
- ◯ Slightly agree
- ◯ Agree
- ◯ Strongly agree

63. Please rate your familiarity with this image. *

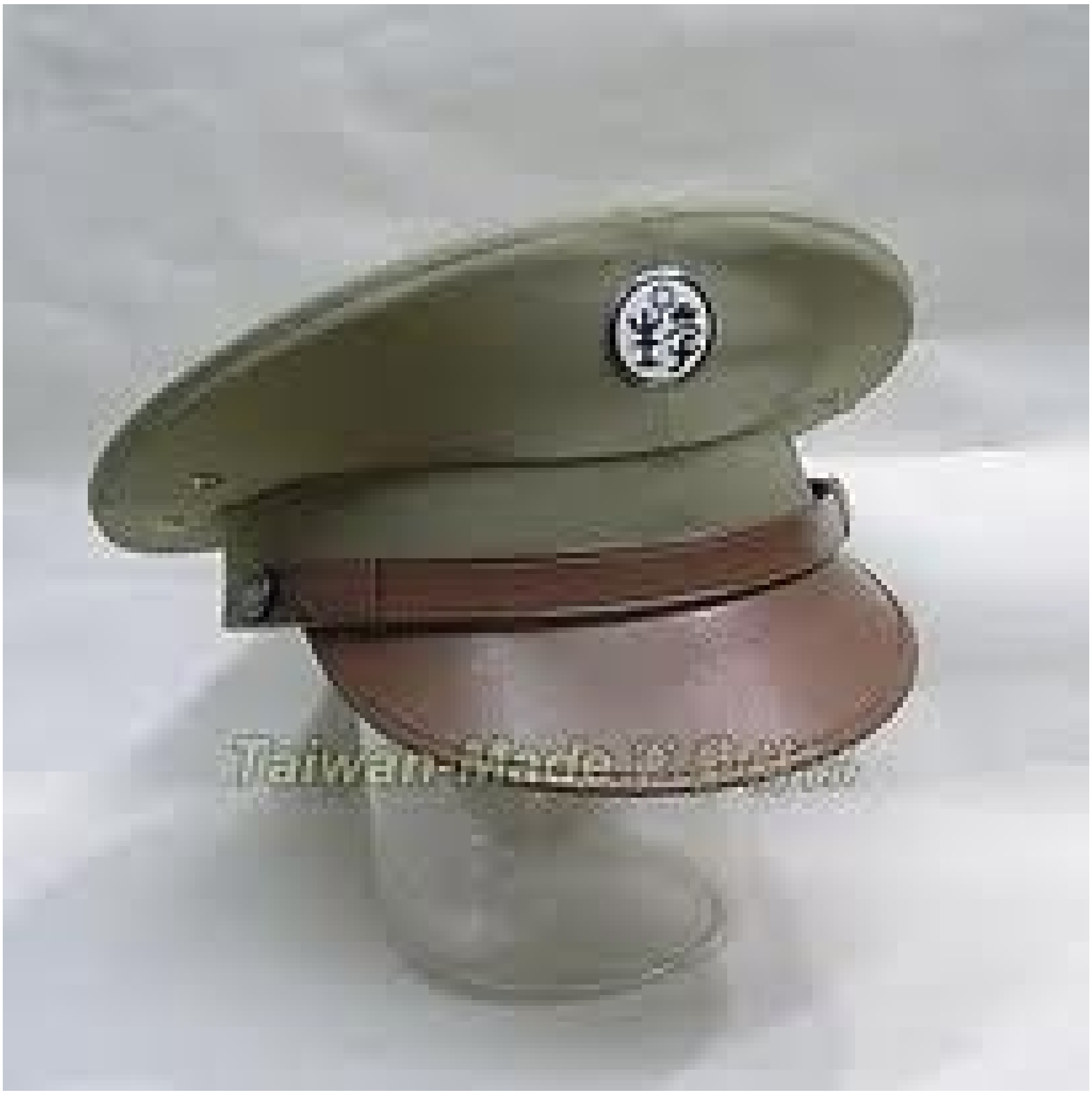

Peaked cap

- Strongly disagree
- Disagree
- Slightly disagree
- Neutral
- Slightly agree
- Agree
- Strongly agree

64. Please rate your familiarity with this image. *

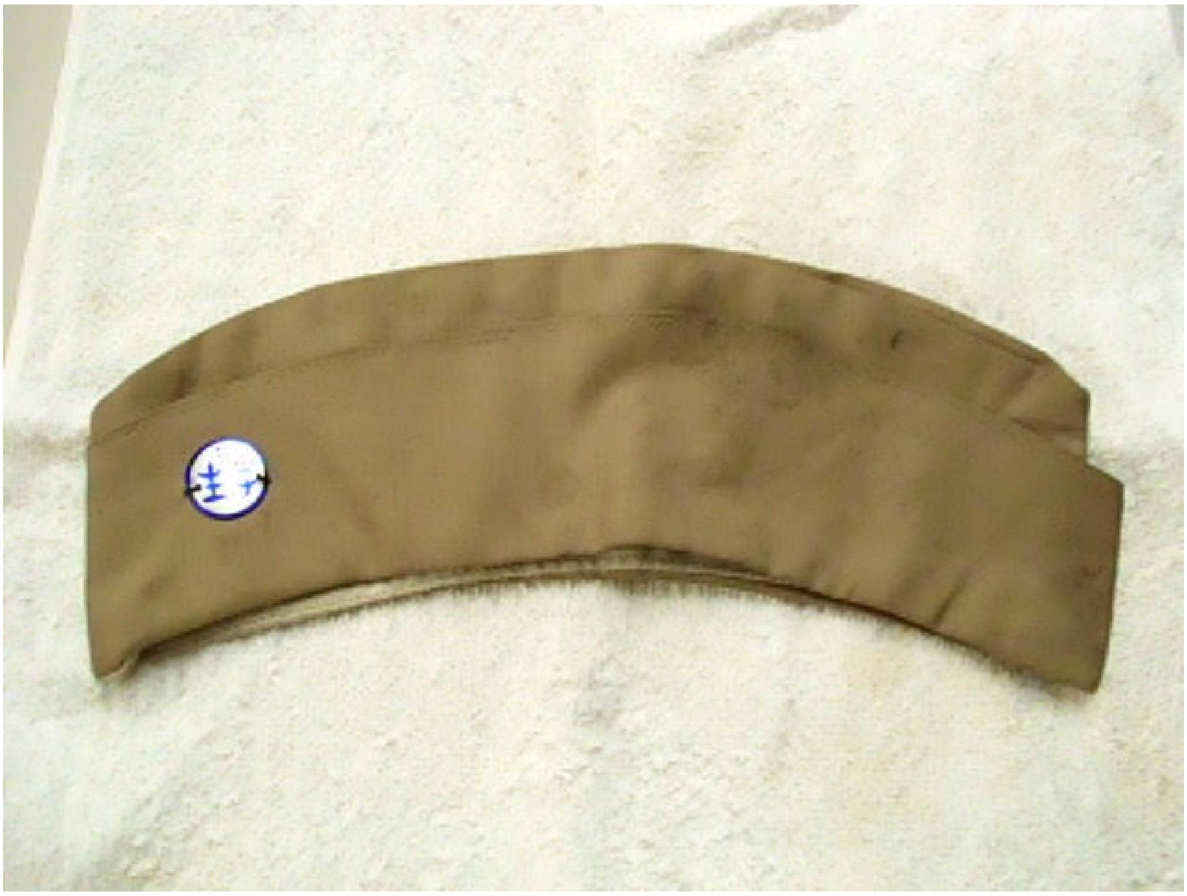

Boat cap

- Strongly disagree
- Disagree
- Slightly disagree
- Neutral
- Slightly agree
- Agree
- Strongly agree

65. Please rate your familiarity with this image. *

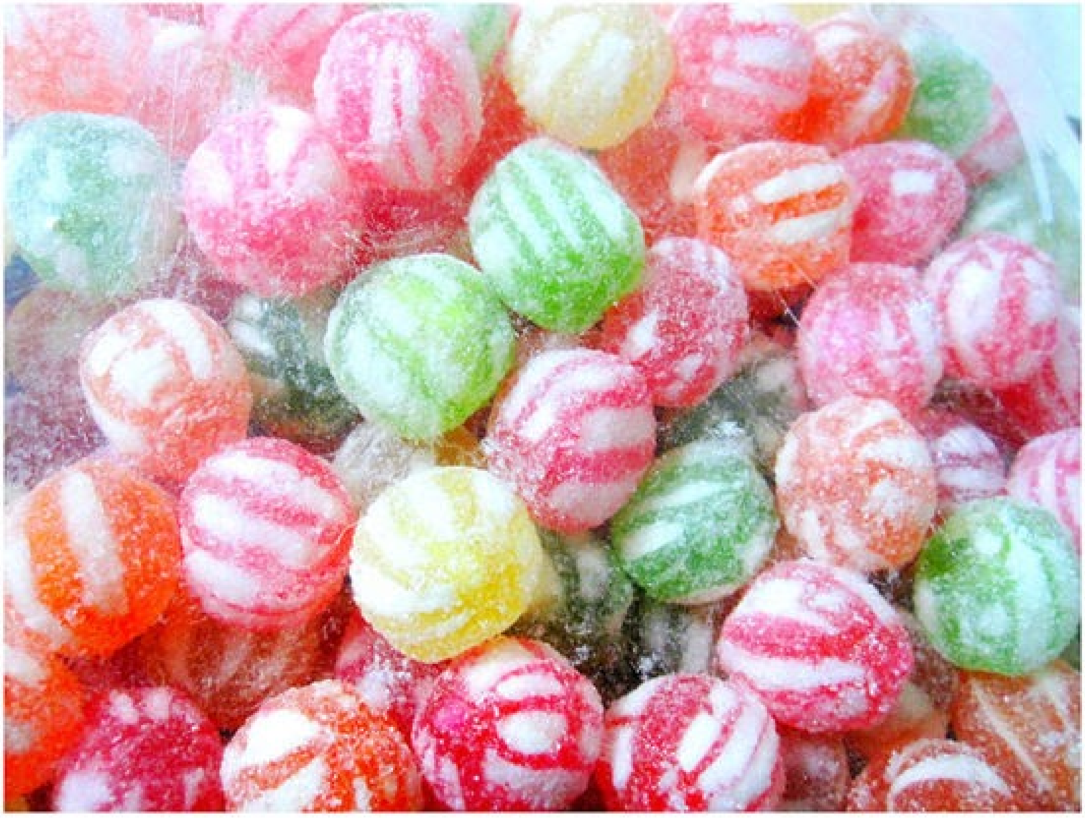

Japanese candy ‘Konpetio’

- Strongly disagree
- Disagree
- Slightly disagree
- Neutral
- Slightly agree
- Agree
- Strongly agree

66. Please rate your familiarity with this image. *

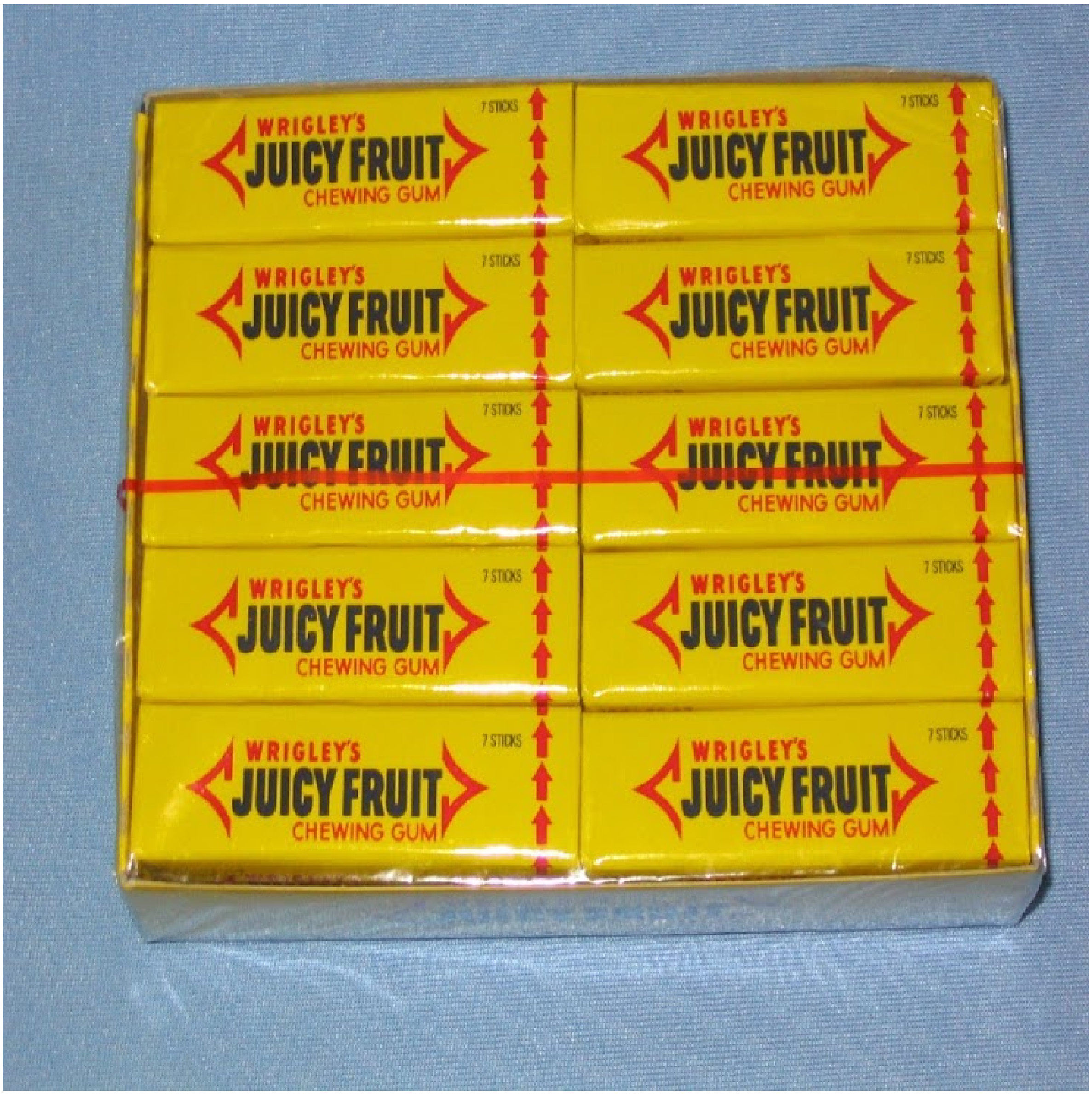

Juicy Fruit chewing gum

- Strongly disagree
- Disagree
- Slightly disagree
- Neutral
- Slightly agree
- Agree
- Strongly agree

67. Please rate your familiarity with this image. *

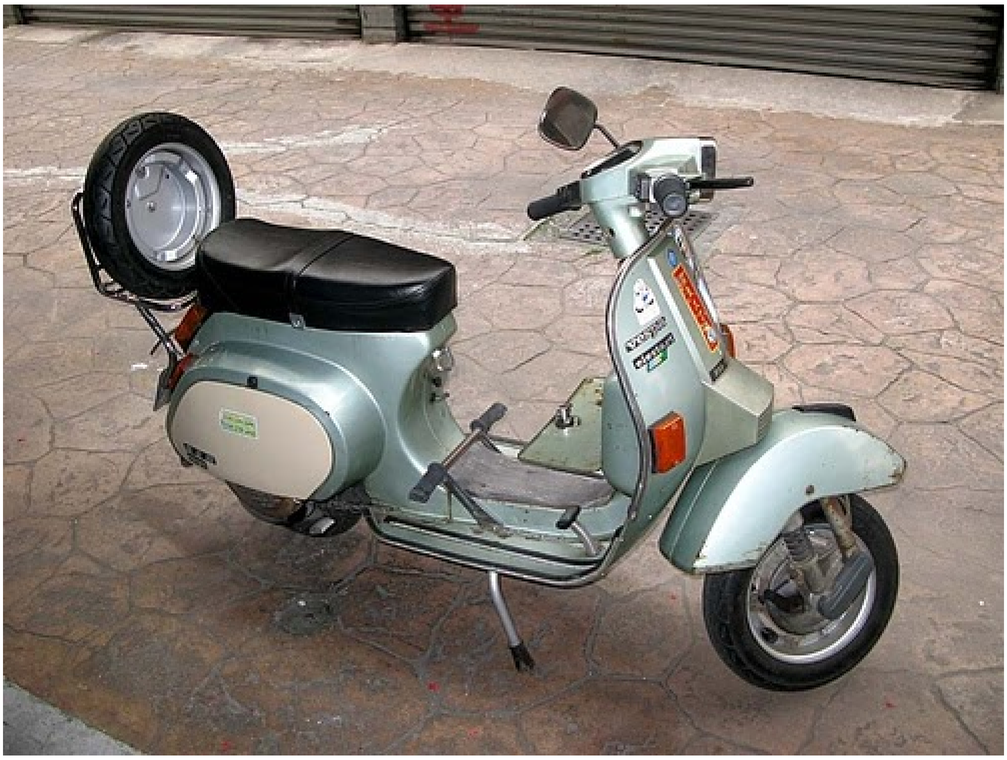

Vespa scooter

- Strongly disagree
- Disagree
- Slightly disagree
- Neutral
- Slightly agree
- Agree
- Strongly agree

68. Please rate your familiarity with this image. *

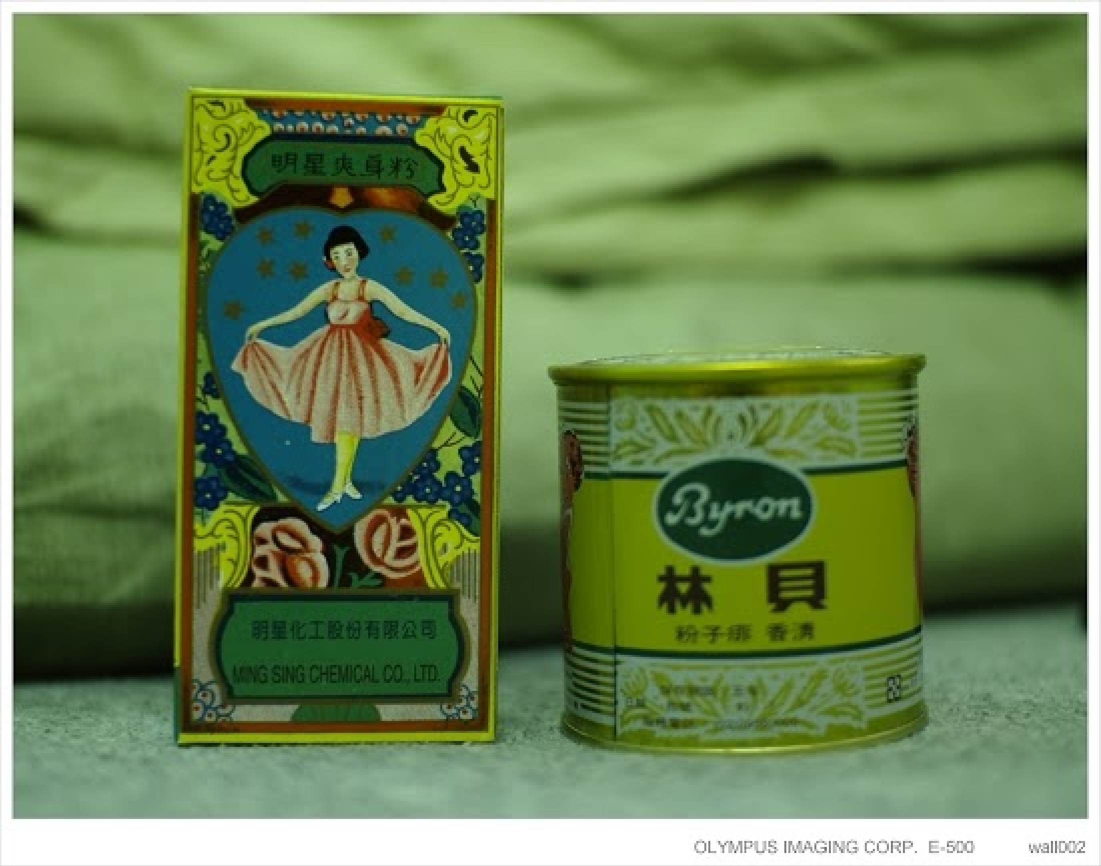

Baby powder

- ◯ Strongly disagree
- ◯ Disagree
- ◯ Slightly disagree
- ◯ Neutral
- ◯ Slightly agree
- ◯ Agree
- ◯ Strongly agree

69. Please rate your familiarity with this image. *

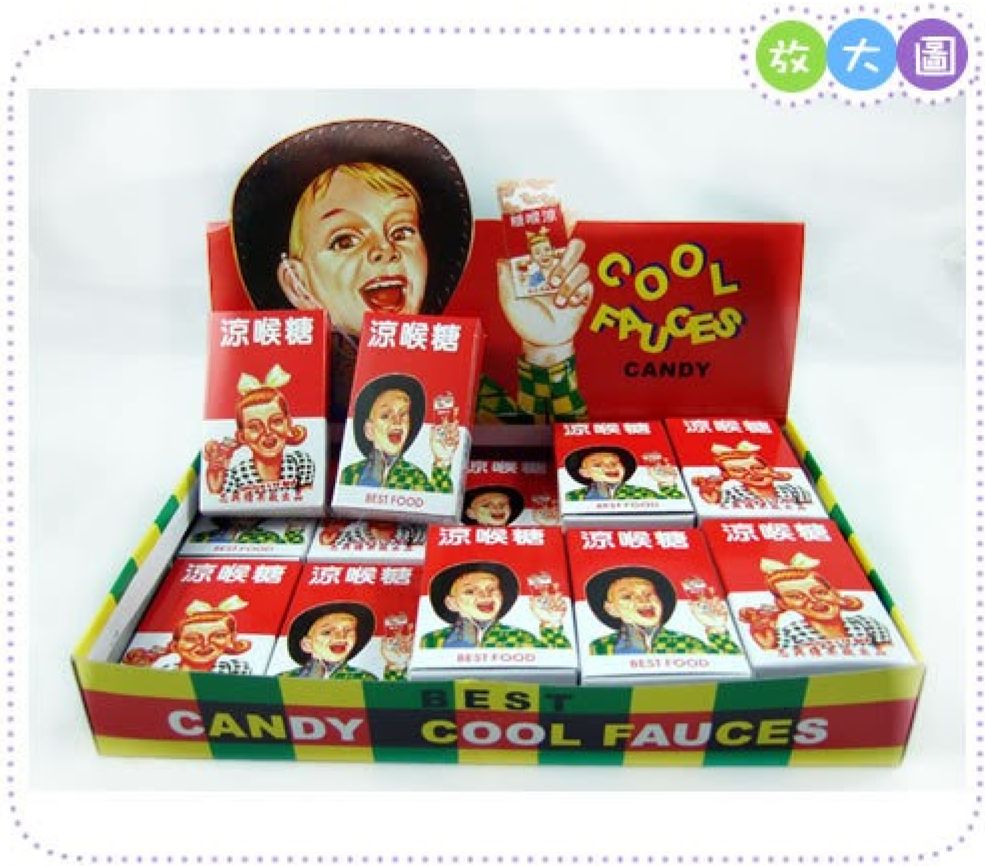

Cool Fauces Candy

- Strongly disagree
- Disagree
- Slightly disagree
- Neutral
- Slightly agree
- Agree
- Strongly agree

70. Please rate your familiarity with this image. *

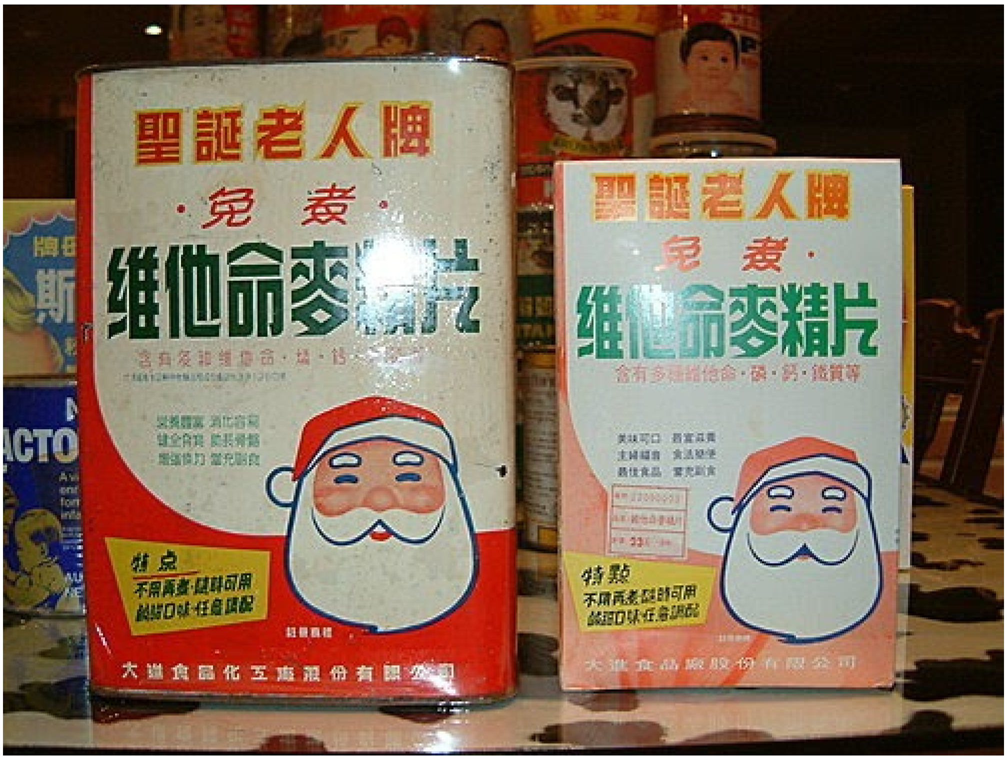

Santa Clause brand oatmeal

- ◯ Strongly disagree
- ◯ Disagree
- ◯ Slightly disagree
- ◯ Neutral
- ◯ Slightly agree
- ◯ Agree
- ◯ Strongly agree

71. Please rate your familiarity with this image. *

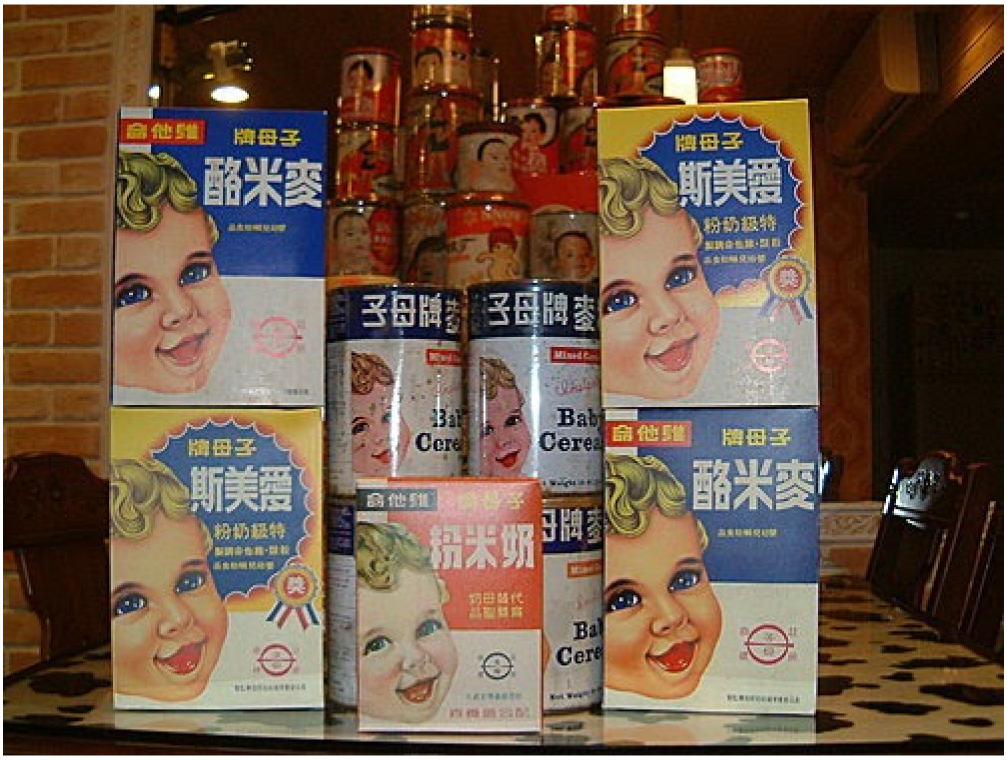

Mother-Son brand milk powder

- ◯ Strongly disagree
- ◯ Disagree
- ◯ Slightly disagree
- ◯ Neutral
- ◯ Slightly agree
- ◯ Agree
- ◯ Strongly agree

72. Please rate your familiarity with this image. *

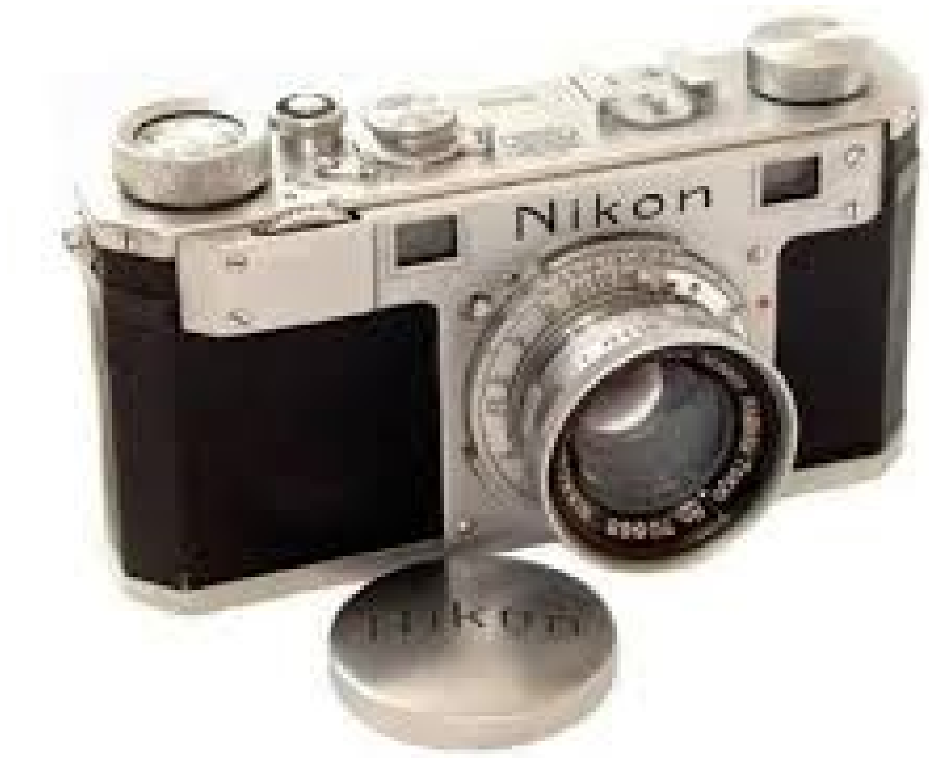

Film camera

- Strongly disagree
- Disagree
- Slightly disagree
- Neutral
- Slightly agree
- Agree
- Strongly agree

73. Please rate your familiarity with this image. *

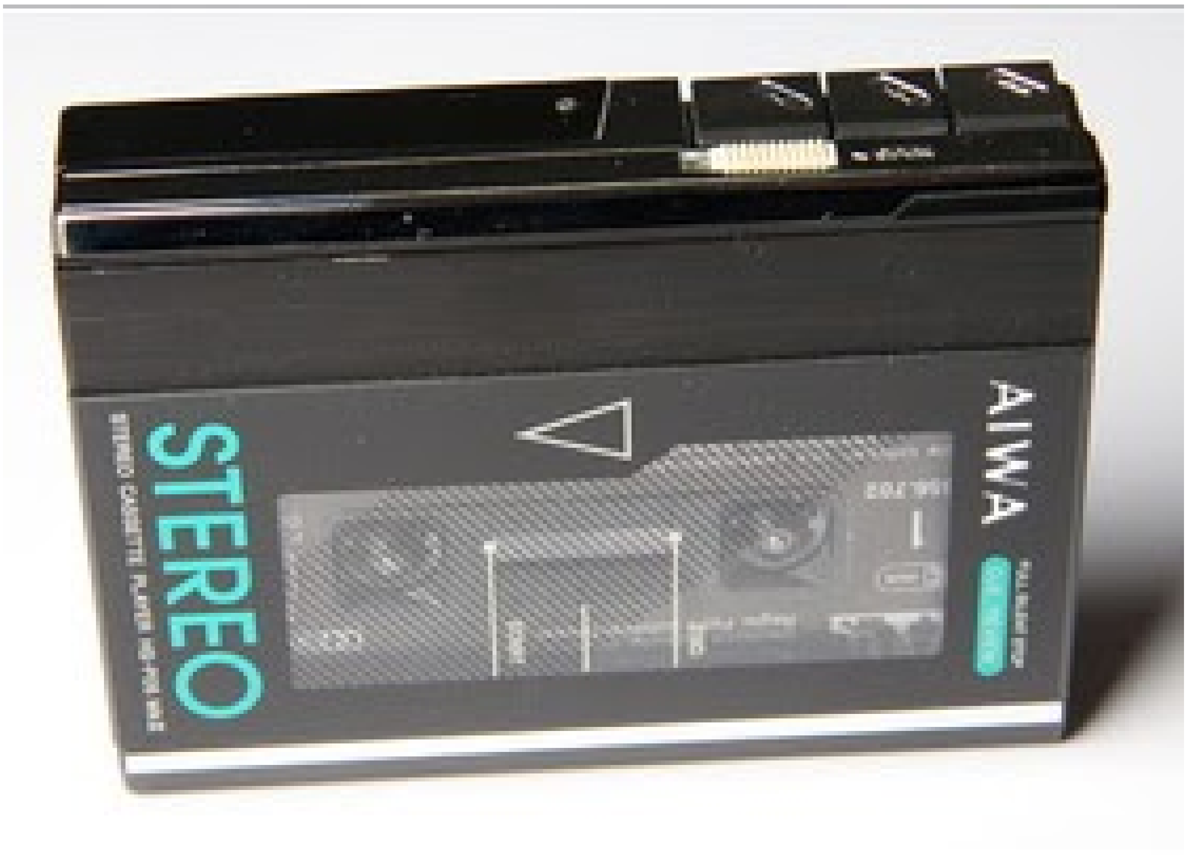

Audio cassette

- Strongly disagree
- Disagree
- Slightly disagree
- Neutral
- Slightly agree
- Agree
- Strongly agree

74. Please rate your familiarity with this image. *

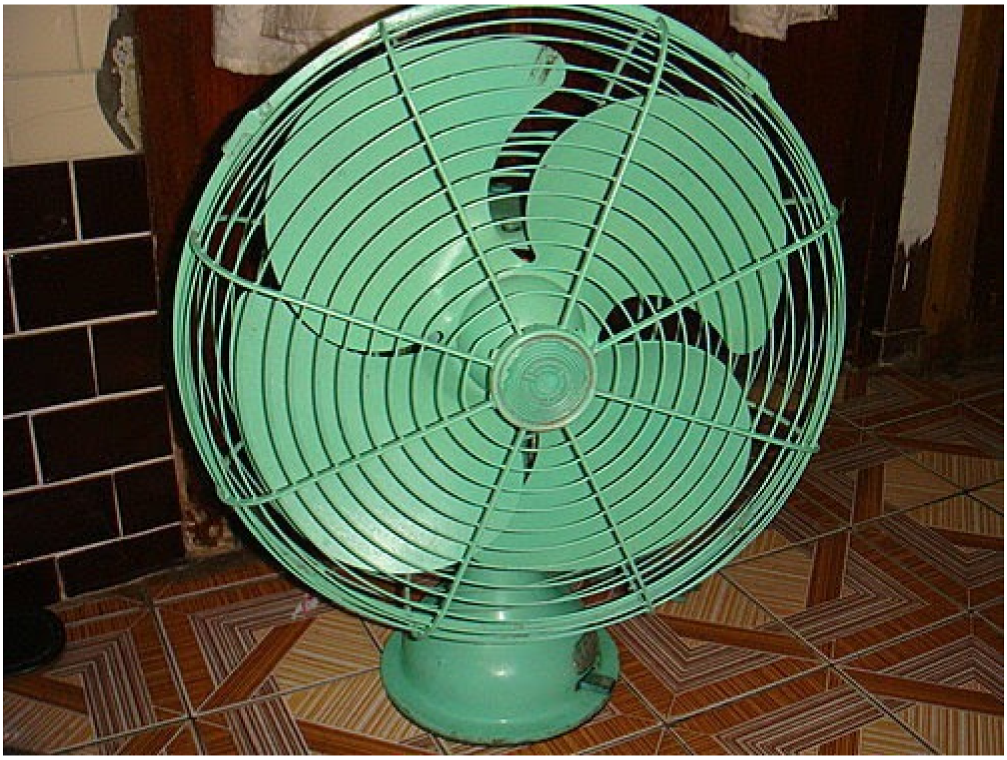

Tatung brand electric fan

- Strongly disagree
- Disagree
- Slightly disagree
- Neutral
- Slightly agree
- Agree
- Strongly agree

75. Please rate your familiarity with this image. *

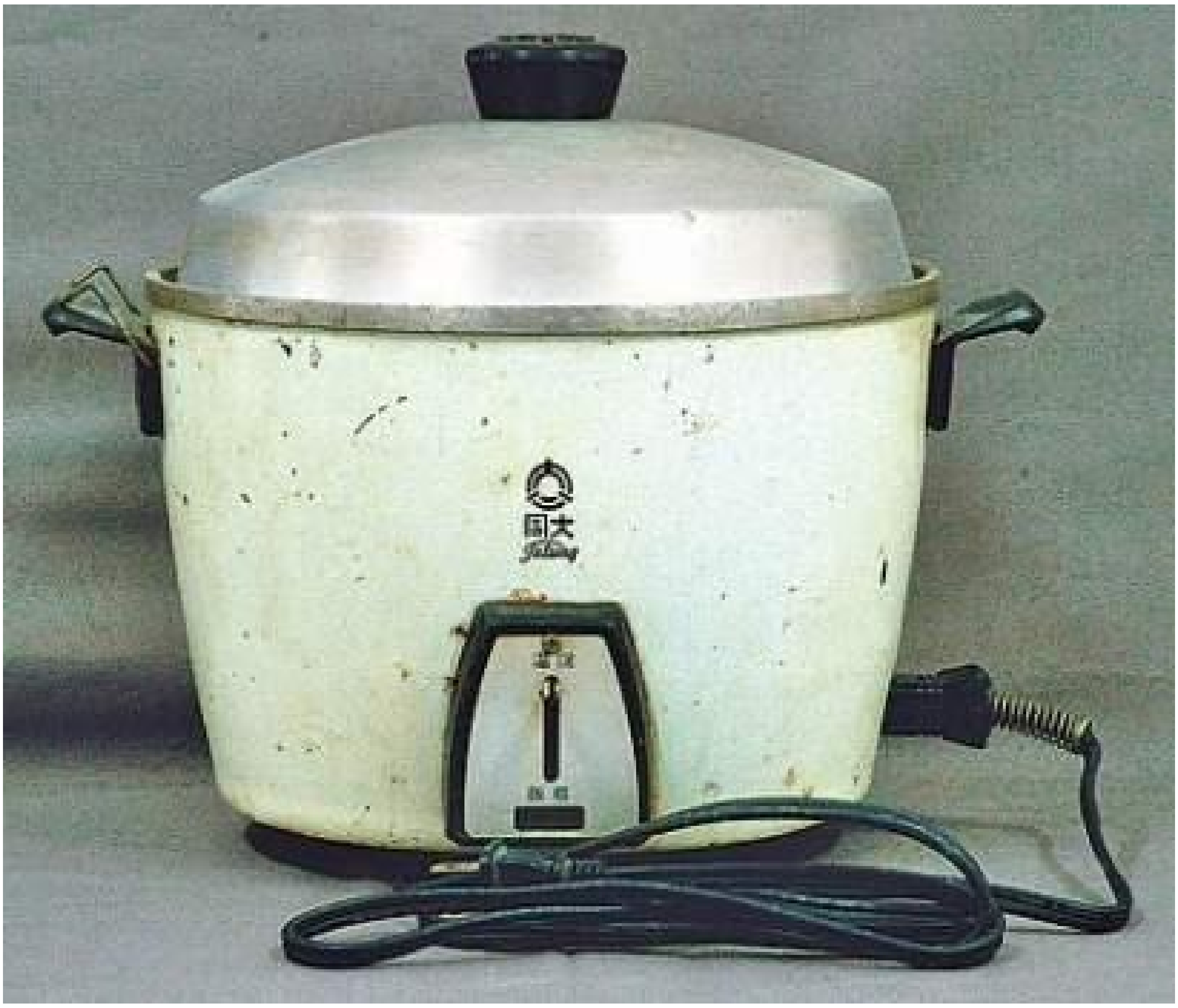

Automatic cooker and steamer

- ◯ Strongly disagree
- ◯ Disagree
- ◯ Slightly disagree
- ◯ Neutral
- ◯ Slightly agree
- ◯ Agree
- ◯ Strongly agree

76. Please rate your familiarity with this image. *

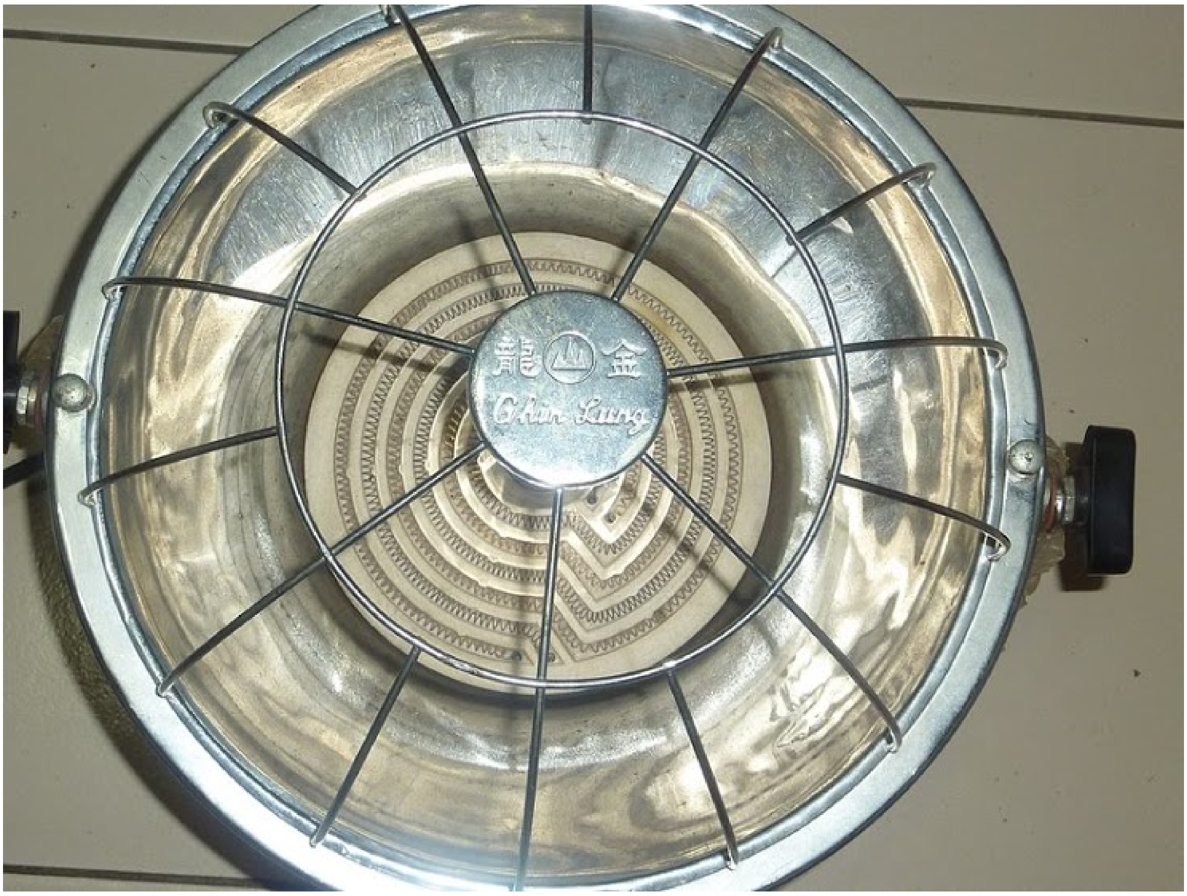

Tungsten electric heater

- Strongly disagree
- Disagree
- Slightly disagree
- Neutral
- Slightly agree
- Agree
- Strongly agree

77. Please rate your familiarity with this image. *

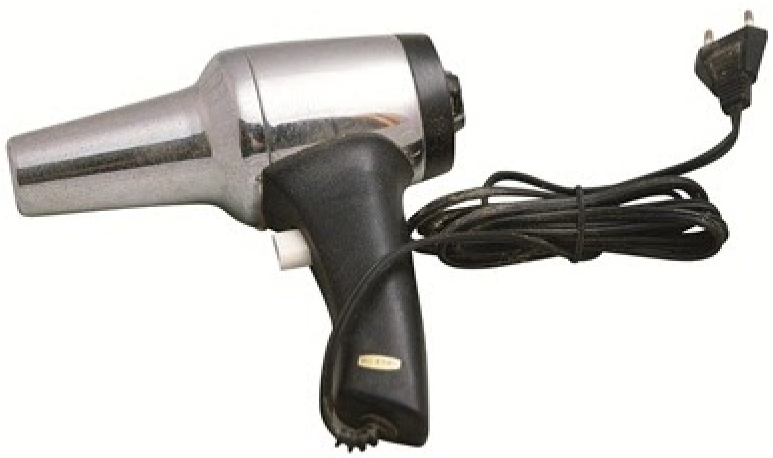

Hairdryer

- Strongly disagree
- Disagree
- Slightly disagree
- Neutral
- Slightly agree
- Agree
- Strongly agree

78. Please rate your familiarity with this image. *

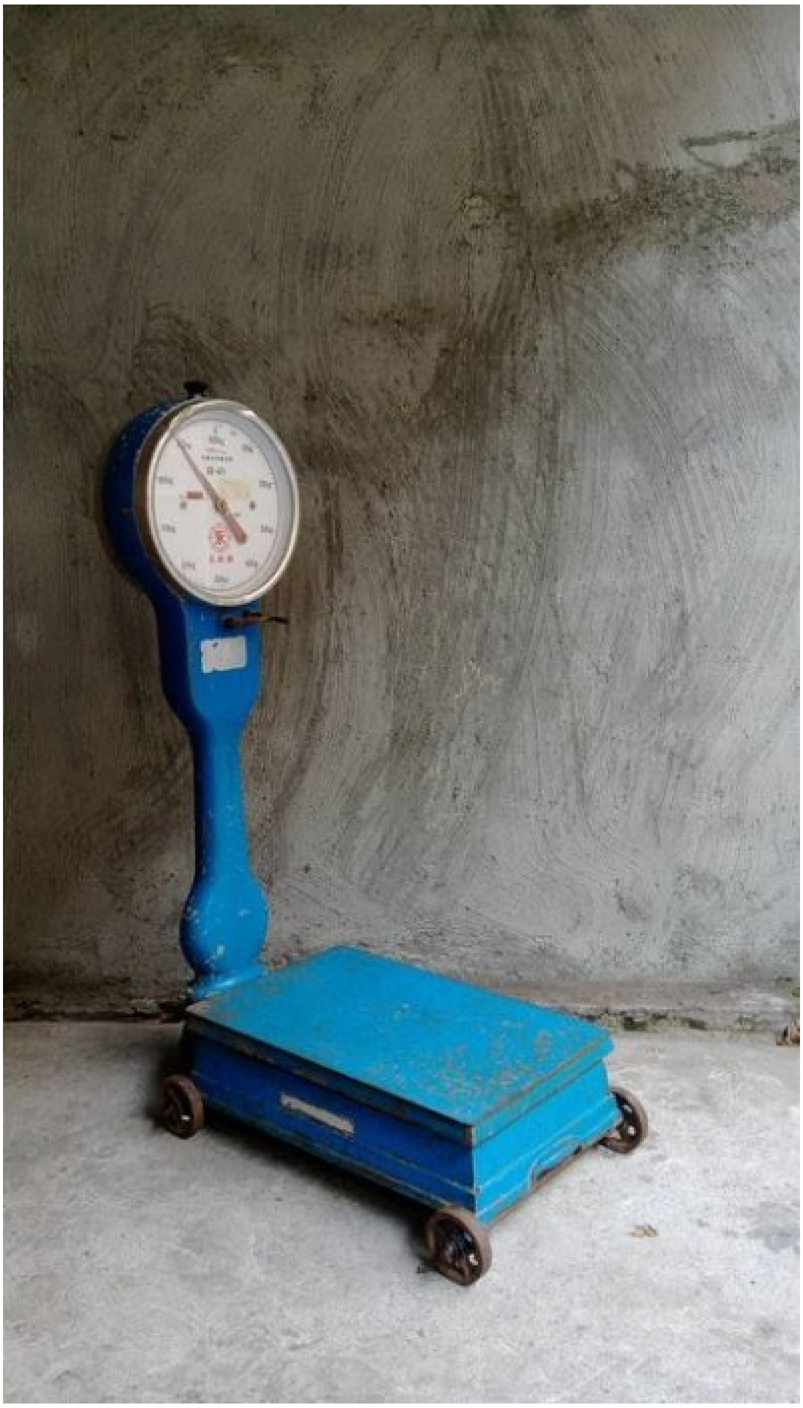

Weight scale

- Strongly disagree
- Disagree
- Slightly disagree
- Neutral
- Slightly agree
- Agree

Strongly agree

79. Please rate your familiarity with this image. *

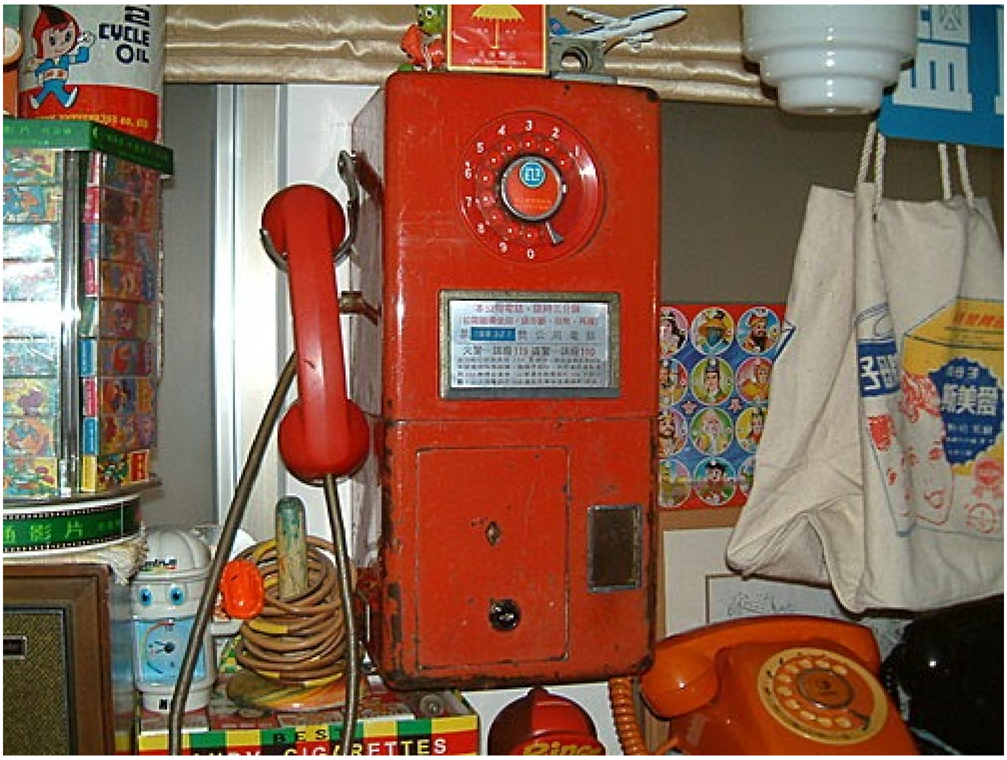

Rotary pay phone

- Strongly disagree
- Disagree
- Slightly disagree
- Neutral
- Slightly agree
- Agree
- Strongly agree

80. Please rate your familiarity with this image. *

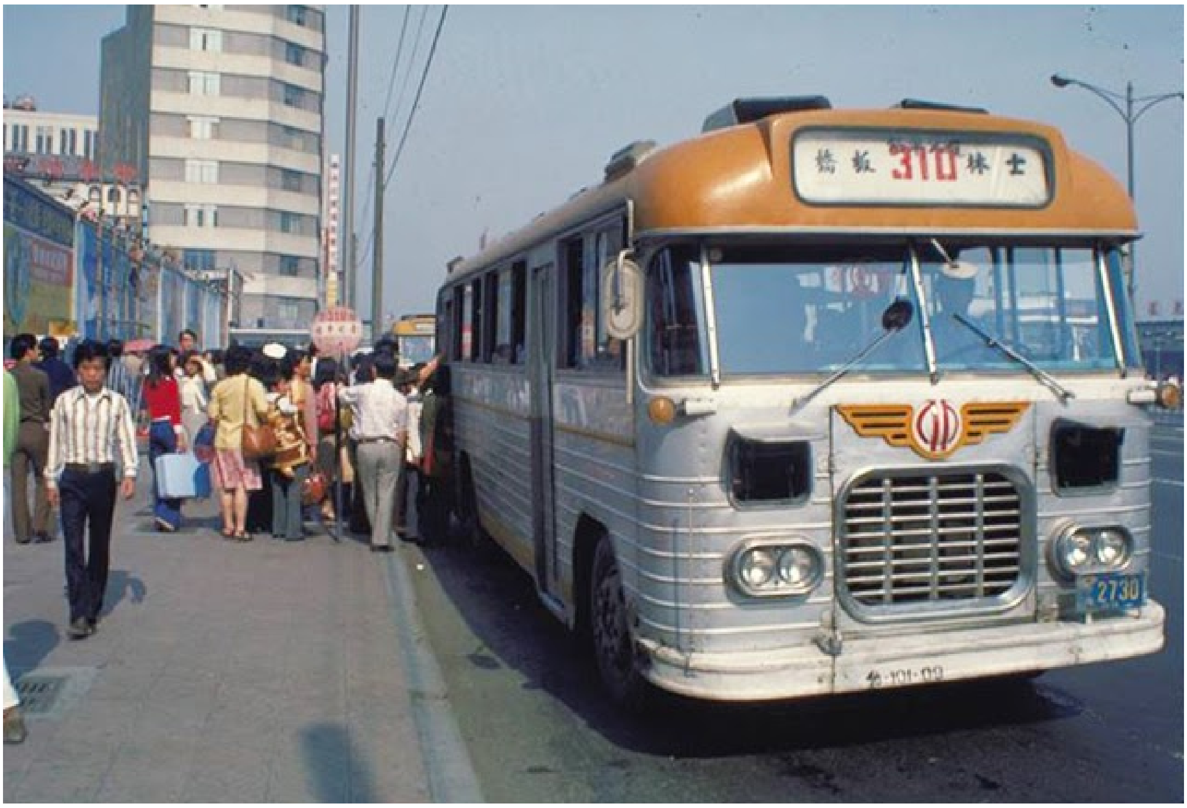

Public bus

- Strongly disagree
- Disagree
- Slightly disagree
- Neutral
- Slightly agree
- Agree
- Strongly agree

81. Please rate your familiarity with this image. *

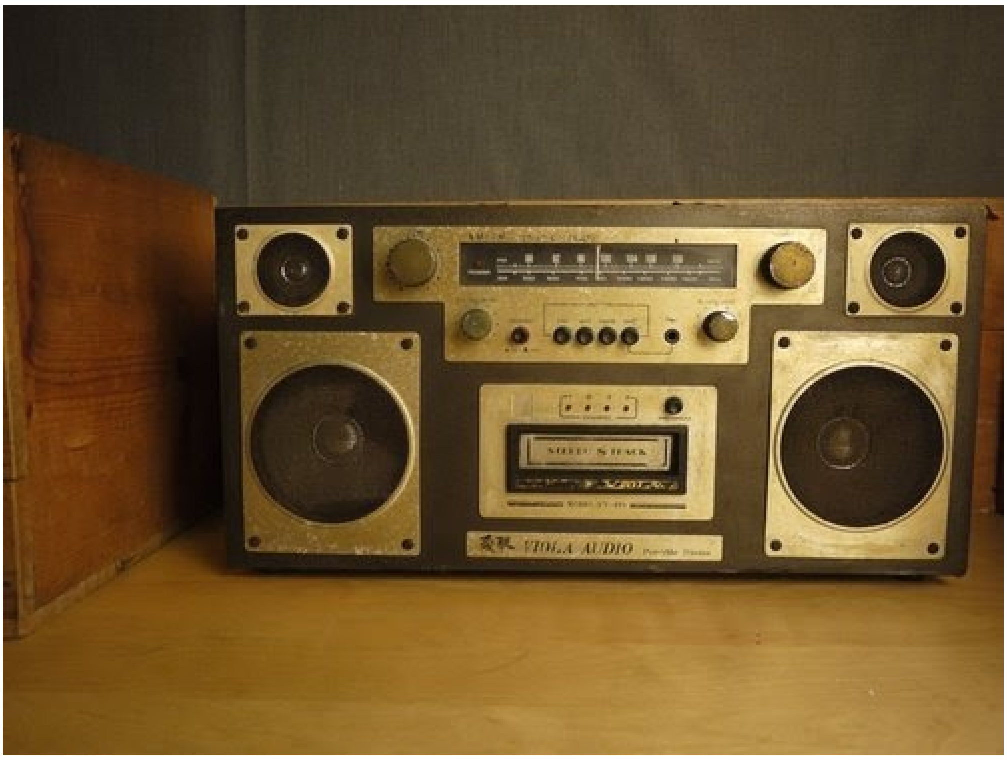

Radio

- Strongly disagree
- Disagree
- Slightly disagree
- Neutral
- Slightly agree
- Agree
- Strongly agree

82. Please rate your familiarity with this image. *

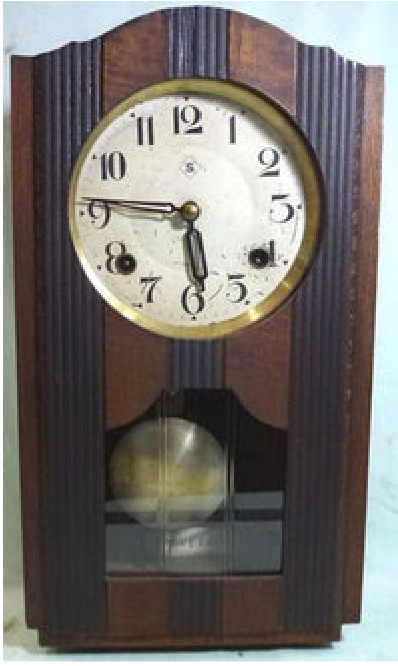

Pendulum clock

- Strongly disagree
- Disagree
- Slightly disagree
- Neutral
- Slightly agree
- Agree
- Strongly agree

83. Please rate your familiarity with this image. *

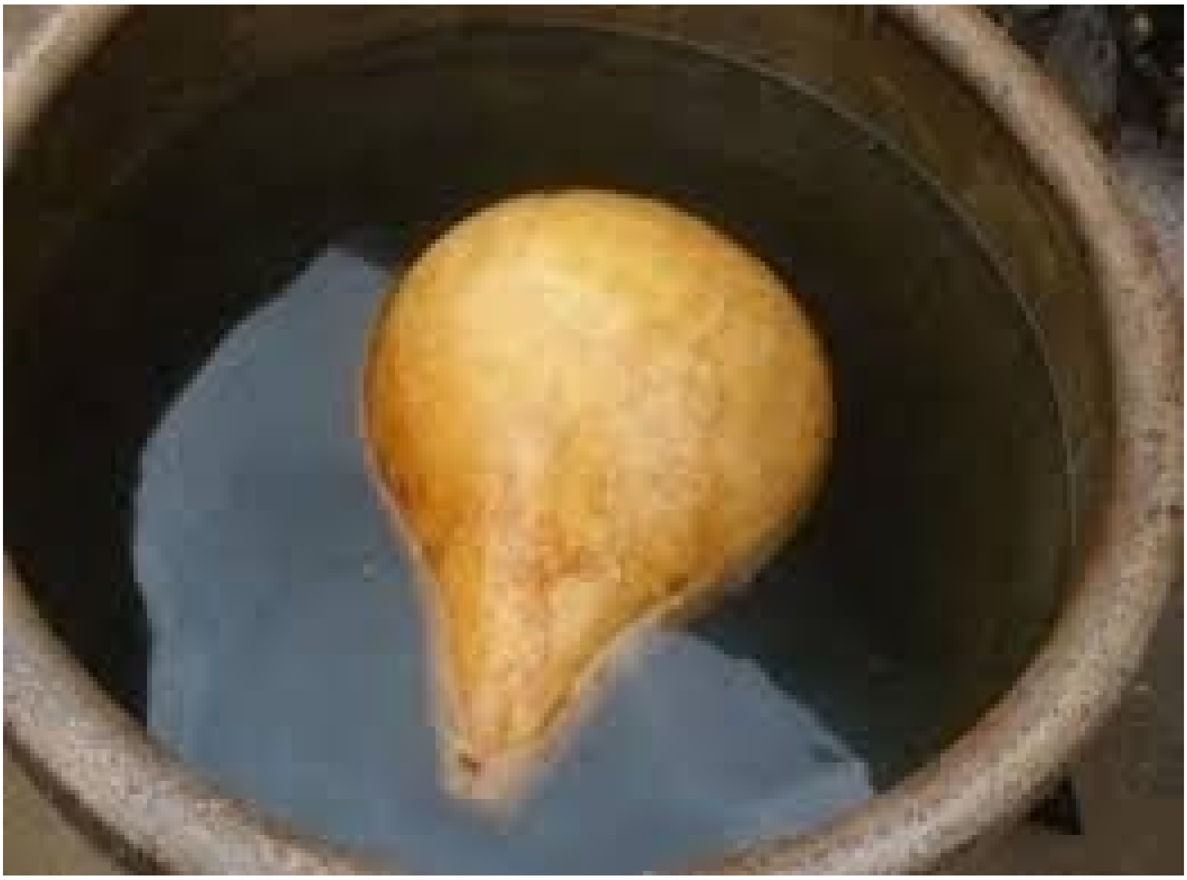

Wood water scoop

- Strongly disagree
- Disagree
- Slightly disagree
- Neutral
- Slightly agree
- Agree
- Strongly agree

84. Please rate your familiarity with this image. *

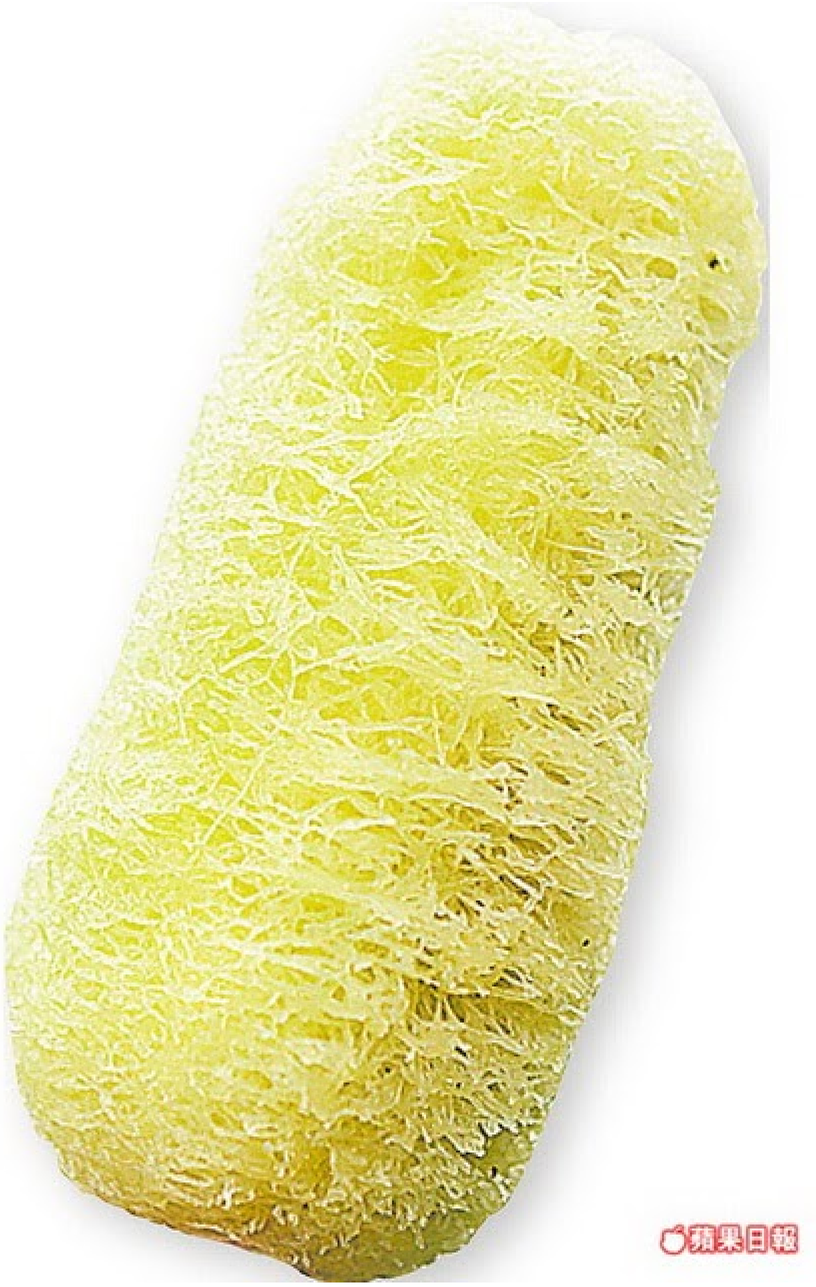

Loofah cloth

- Strongly disargee
- Disagree
- Slightly disagree
- Neutral
- Slightly agree
- Agree
- Strongly agree

85. Please rate your familiarity with this image. *

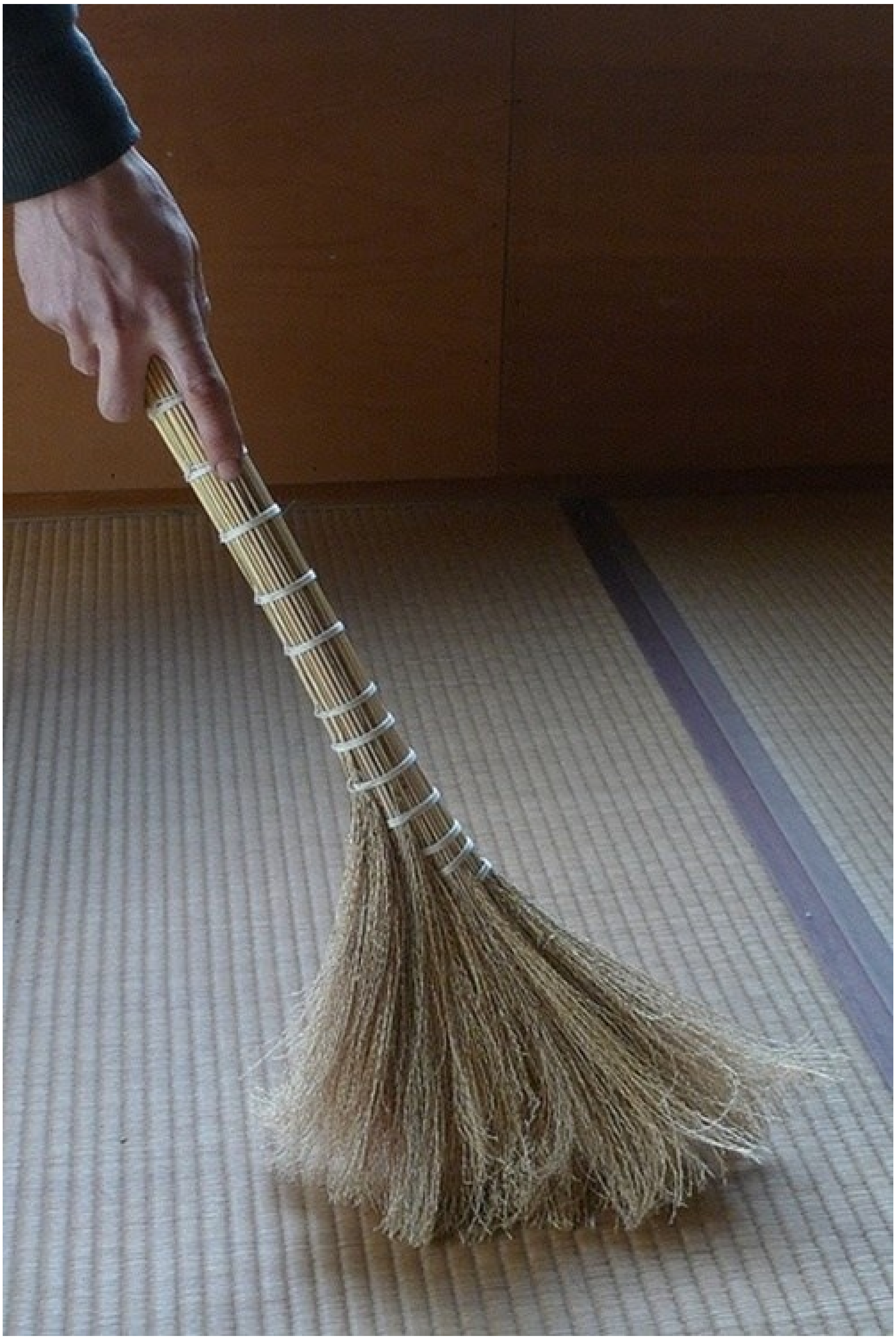

Straw broom

- Strongly disagree
- Disagree
- Slightly disagree
- Neutral
- Slightly agree

Agree

Strongly agree

86. Please rate your familiarity with this image. *

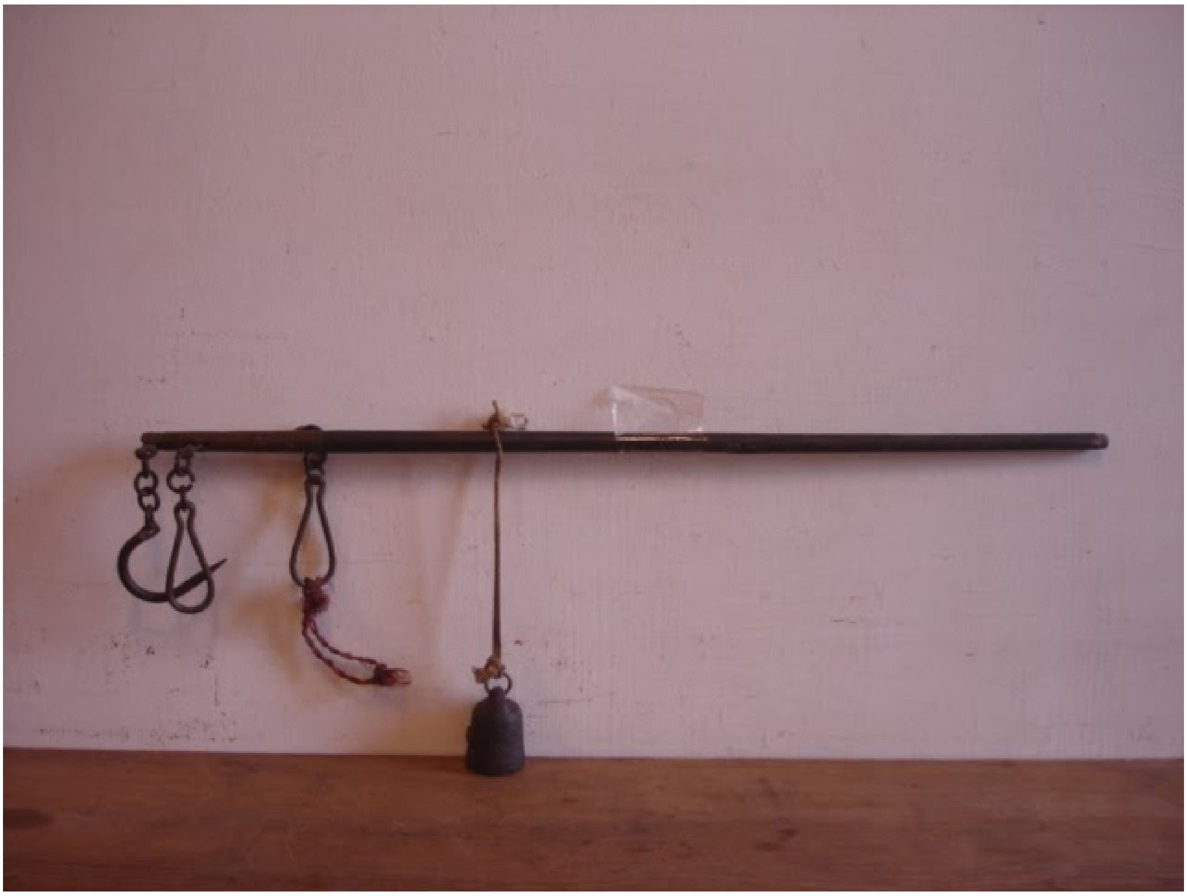

Steelyard

Strongly disagree

Disagree

Slightly disagree

Neutral

Slightly agree

Agree

Strongly agree

87. Please rate your familiarity with this image. *

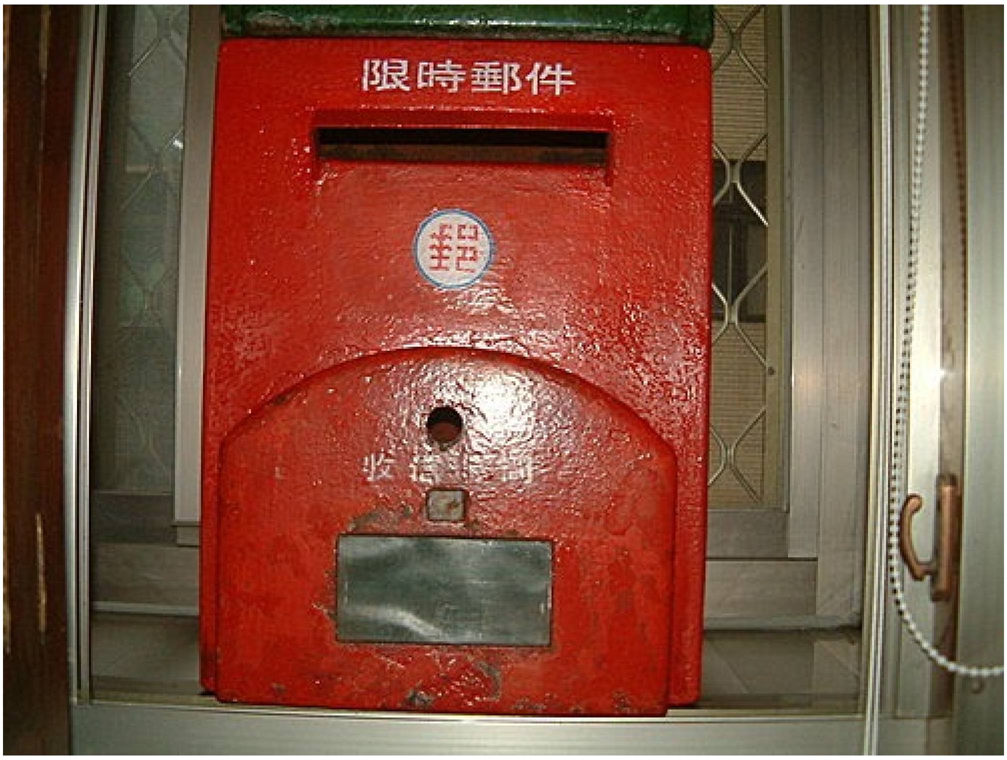

Postbox

- Strongly disagree
- Disagree
- Slightly disagree
- Neutral
- Slightly agree
- Agree
- Strongly agree

88. Please rate your familiarity with this image. *

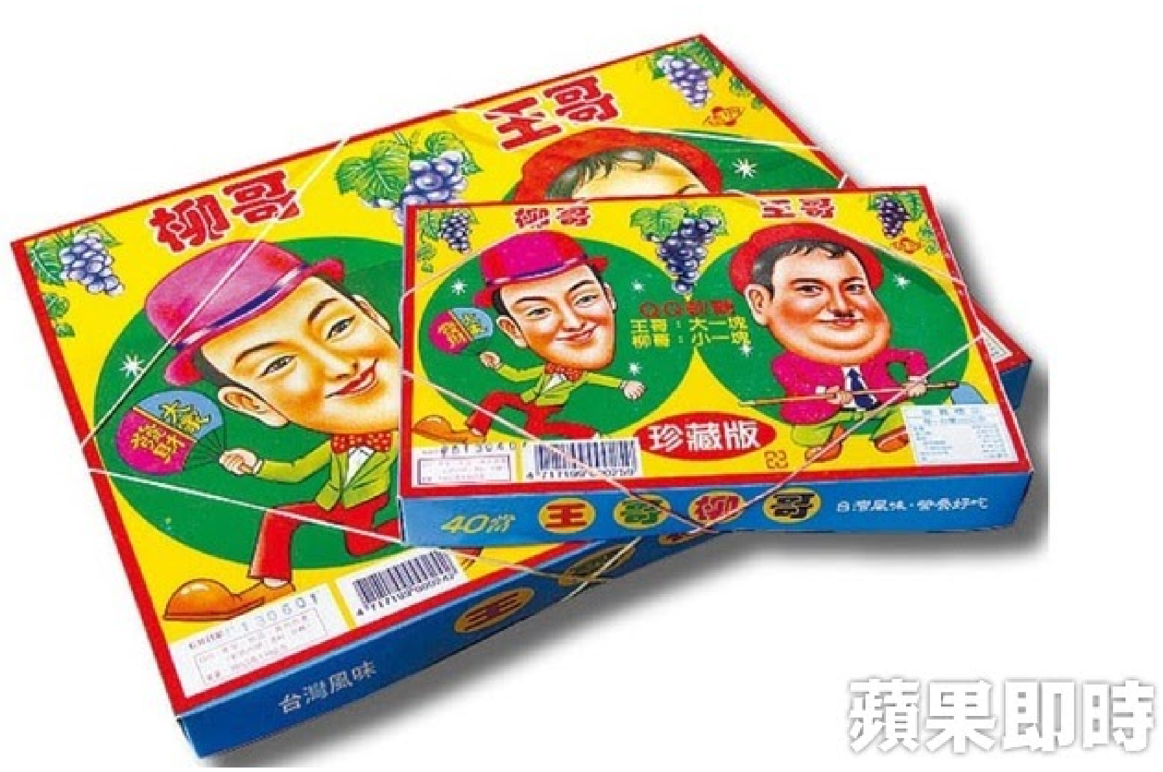

Soft candy

- Strongly disagree
- Disagree
- Slightly disagree
- Neutral
- Slightly agree
- Agree
- Strongly agree

89. Please rate your familiarity with this image. *

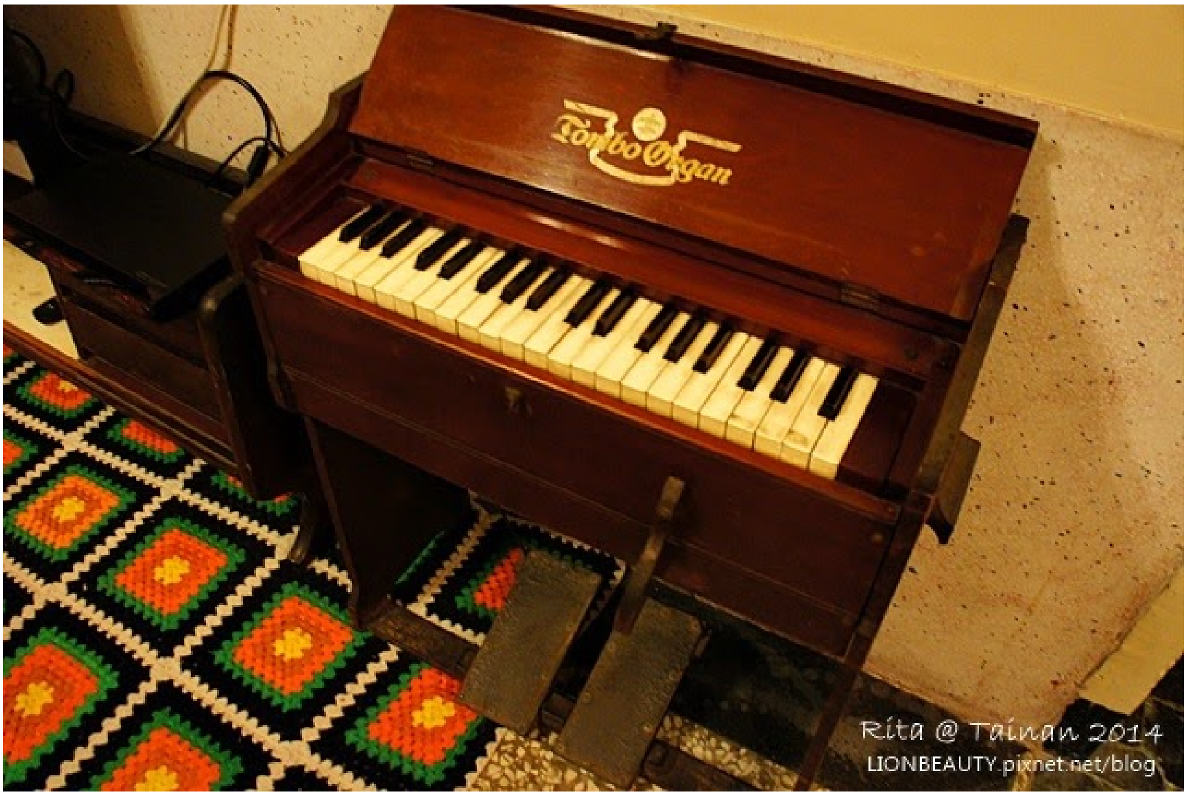

Reed organ

- Strongly disagree
- Disagree
- Slightly disagree
- Neutral
- Slightly agree
- Agree
- Strongly agree

90. Please rate your familiarity with this image. *

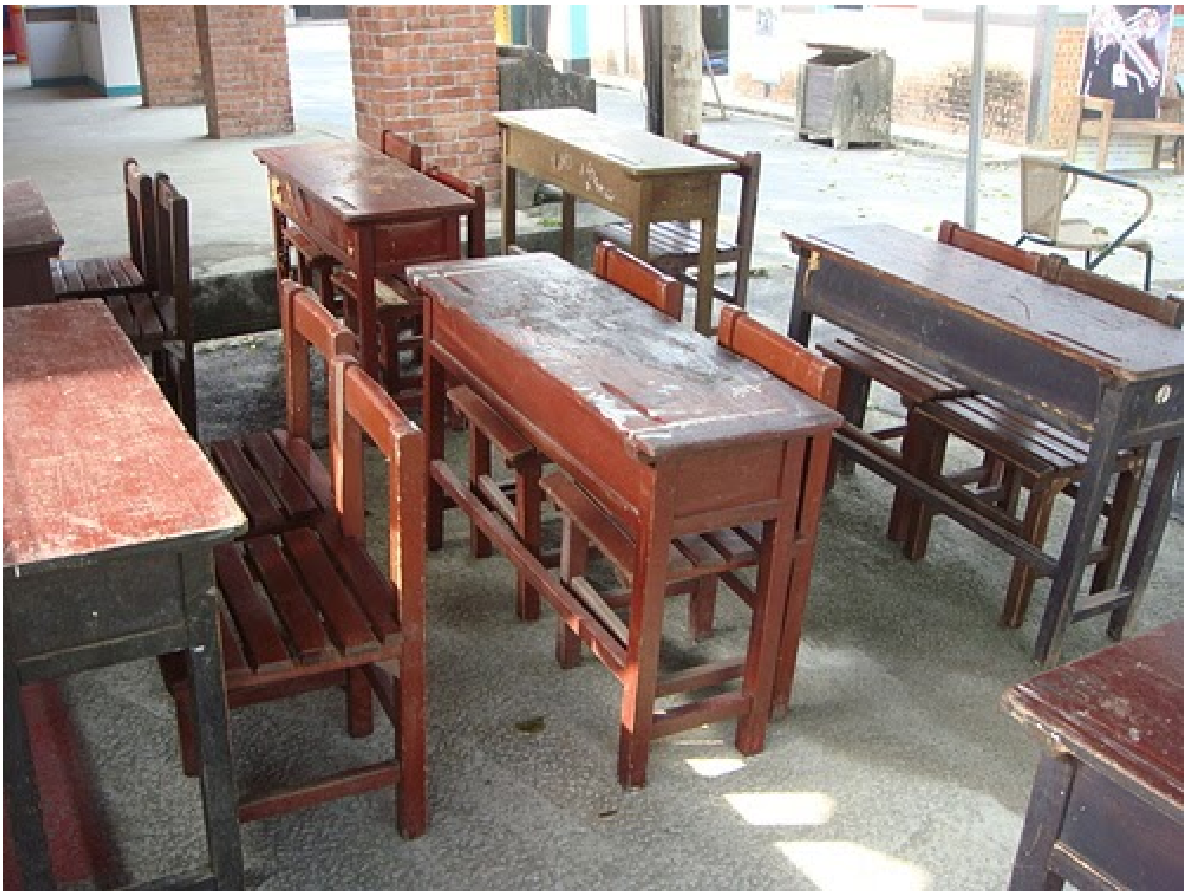

School desks and chairs

- Strongly disagree
- Disagree
- Slightly disagree
- Neutral
- Slightly agree
- Agree
- Strongly agree

91. Please rate your familiarity with this image. *

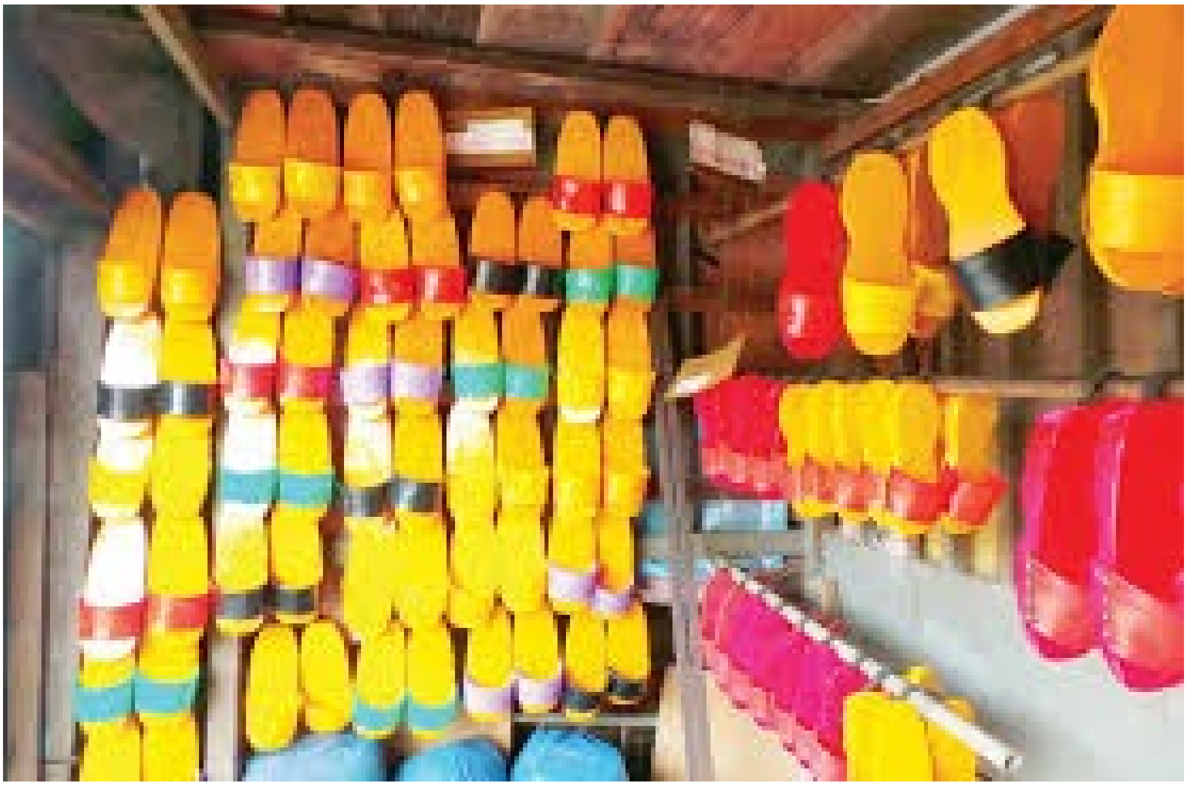

Wood clogs

- Strongly disagree
- Disagree
- Slightly disagree
- Neutral
- Slightly agree
- Agree
- Strongly agree

92. Please rate your familiarity with this image. *

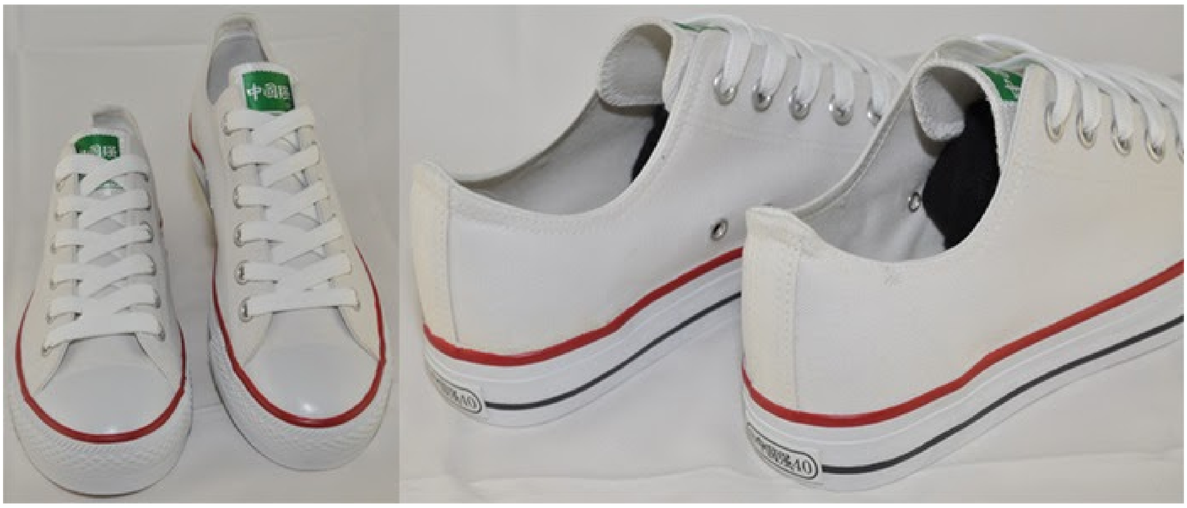

Sneakers

- Strongly disagree
- Disagree
- Slightly disagree
- Neutral
- Slightly agree
- Agree
- Strongly agree

93. Please rate your familiarity with this image. *

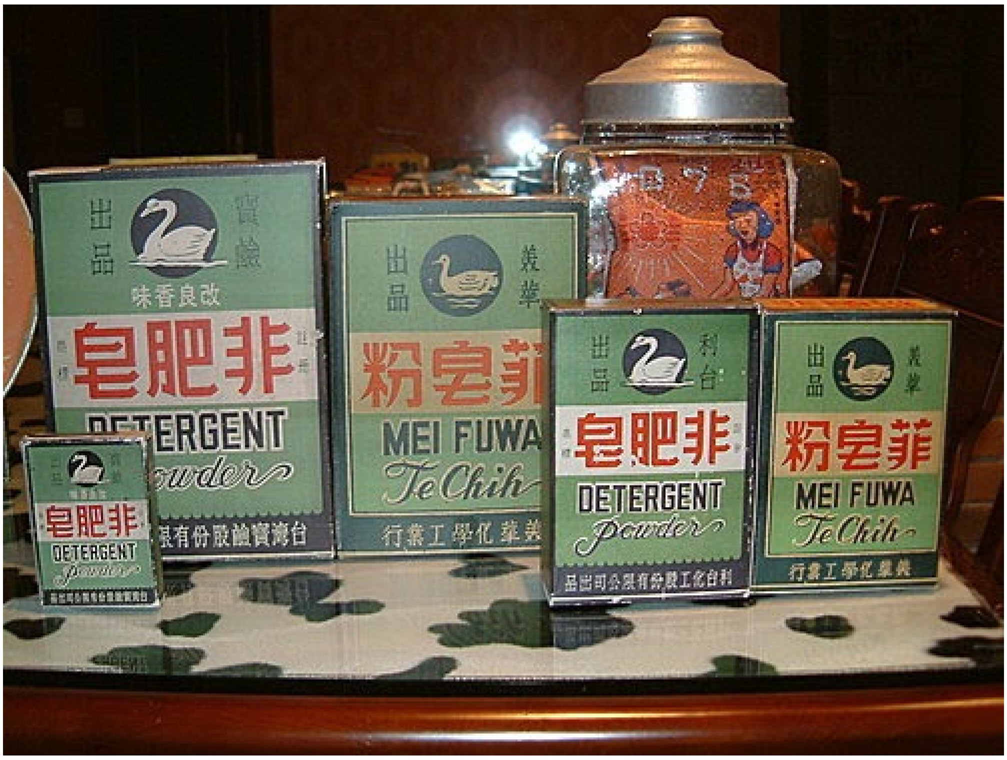

Soap/detergent

- ◯ Strongly disagree
- ◯ Distragree
- ◯ Slightly disagree
- ◯ Neutral
- ◯ Slightly agree
- ◯ Agree
- ◯ Strongly agree

Are there other objects from the 1960's that bring you a familiar feeling? *

您的回答

This survey ends here! Thank you for your patient participation.

提交

請勿利用 Google 表單送出密碼。

Google 並未認可或建立這項內容。 檢舉濫用情形 - 服務條款 - 隱私權政策

Google 表單
